# Metabolites in Cherry Buds to Detect Winter Dormancy

**DOI:** 10.3390/metabo12030247

**Published:** 2022-03-16

**Authors:** Frank-M. Chmielewski, Klaus-P. Götz

**Affiliations:** Agricultural Climatology, Faculty of Life Sciences, Humboldt-University of Berlin, Albrecht-Thaer-Weg 5, 14195 Berlin, Germany

**Keywords:** *Prunus avium* L., cv. ‘Summit’, sweet cherry, global metabolite profiling, plant metabolites, winter dormancy, endodormancy release, beginning of ontogenetic development, phenological modelling

## Abstract

Winter dormancy is still a “black box” in phenological models, because it evades simple observation. This study presents the first step in the identification of suitable metabolites which could indicate the timing and length of dormancy phases for the sweet cherry cultivar ‘Summit’. Global metabolite profiling detected 445 named metabolites in flower buds, which can be assigned to different substance groups such as amino acids, carbohydrates, phytohormones, lipids, nucleotides, peptides and some secondary metabolites. During the phases of endo- and ecodormancy, the energy metabolism in the form of glycolysis and the tricarboxylic acid (TCA) cycle was shut down to a minimum. However, the beginning of ontogenetic development was closely related to the up-regulation of the carbohydrate metabolism and thus to the generation of energy for the growth and development of the sweet cherry buds. From the 445 metabolites found in cherry buds, seven were selected which could be suitable markers for the ecodormancy phase, whose duration is limited by the date of endodormancy release (t_1_) and the beginning of ontogenetic development (t_1_*). With the exception of abscisic acid (ABA), which has been proven to control bud dormancy, all of these metabolites show nearly constant intensity during this phase.

## 1. Introduction

Phenological stages of plants and environmental conditions are important factors which regulate growth and development, and therefore changes at the transcriptional level, to respond to intra- and extracellular signals. This in turn controls the metabolic state of the plant. The mechanisms behind the expression of genes and the resulting metabolic phenotype of plants and their organs are very complex [1]. Current research results highlight that the organogenesis of cherry buds during paradormancy, endodormancy and ecodormancy is defined by the expression of genes involved in specific pathways [2]. For example, *DORMANCY ASSICIATED MADS*-box (*DAM*), floral identity and organogenesis genes are up-regulated until the start of endodormancy, while endodormancy itself shows a more complex array of signaling pathways. These include genes that react to cold, are involved in abscisic acid (ABA) synthesis and in oxidation-reduction processes. Thus, they are related to the transition between different phases of bud dormancy. Metabolites, as well as their intermediates, are classified as either primary or secondary, based on their intrinsic functions within the organism. Primary metabolites are different types of organic compounds that are involved in growth and development, the synthesis of hormones and proteins, photosynthesis, and respiration. They are present in almost every part of the plant species [3]. Secondary metabolites are biologically active substances to protect plants from diseases, microbes, and herbivore elicitors. 

Studies focused on the metabolic characterization of bud dormancy in *Prunus avium* L. represent an interesting framework to anticipate adaptation to weather and climate [4]. However, physiological and molecular studies are currently receiving increasing attention, as research on a broad range of metabolites and genes in plant species, such as cherries [5,6,7,8,9,10], grapevine [11] and rhubarb [12] and other deciduous trees [13,14,15], shows. It was shown that ABA and the representatives of gibberellins, GA_3_ and GA_4_, have significant dormancy-alleviating effects in sweet cherry cultivars, similar to observations in Japanese apricot and peach [9]. In cherry buds, the main phenolic compounds, caffeoylquinic acids, coumaroylquinic acids and catechins, as well as quercetin and kaempferol derivatives, were identified and demonstrate that the content of phenolic compounds decreases until the end of endodormancy [10]. Furthermore, data regarding ascorbate and glutathione support the hypothesis of their protective role during the para- and endodormancy phases. A comprehensive overview of the current knowledge about the winter dormancy of deciduous trees is given by Fadón et al. [15]. In this study, findings on the dormancy of woody plants are summarised, considering the dynamics of phytohormones and genetic and epigenetic regulations.

**Figure 1:**
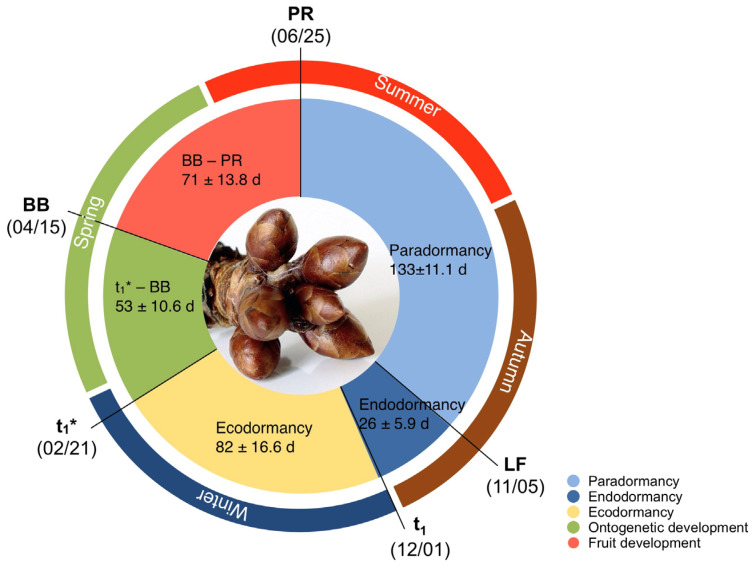
Average timing of phenological stages and duration of phenological phases of the sweet cherry cv. ‘Summit’ for 9 years (2011/12–2019/20) at the experimental orchard in Berlin-Dahlem. ‘Picking ripeness’ (PR), ‘total leaf fall’ (LF), endodormancy release (t_1_), beginning of ontogenetic development (t_1_*), and ‘beginning of blossom’ (BB, BBCH 61). Numbers in the inner circle give the length of phases ± standard deviation in days (d). Extended data from [17].

In order to detect relevant metabolites, which are related to the induction, maintenance and release of dormancy phases [16], it is necessary to know the timing of the phenological stages, which limit them (Figure 1). A major challenge here is that the date of endodormancy release (t_1_) and the beginning of ontogenetic development (t_1_*), the latter of which must start a few weeks before any visible changes in the buds are detectable, cannot be recorded by phenological observations. For this reason, it was necessary to detect the timing of t_1_ and t_1_* before we were able to identify any metabolites that might be related to the induction or release of winter dormancy. For nine years, we analysed the timing of t_1_ by means of the sampling of twigs, which were placed in a climate chamber and t_1_* was analysed according to changes in the flower bud’s water content [17].

Figure 1 shows the four relevant phases of flower bud development from bud initiation to blossom, as well as the time until fruit ripeness. Shortly after picking ripeness in summer, new buds appear on the tree (bud set), which grow and develop until leaf fall. Thus, in temperate climates, LF marks the end of paradormancy (phase 1), which for ‘Summit’ lasts 133 ± 11.1 days in NE Germany. During this phase, bud development can be controlled by the growth of plant organs (apical dominance), other than substances in the bud itself [18]. The transition from paradormancy to endodormancy (phase 2) begins with the cessation of growth due to declining temperature and photoperiod [19,20], while for *Prunus* species, the influence of photoperiod probably depends on air temperature [21]. For ‘Summit’, the endodormancy phase (true winter rest) at our site lasts 26 ± 5.9 days, and ends very consistently at the end of November or beginning of December. It is assumed that the state of endodormancy can be released by the accumulation of chill units, until the cultivar specific chilling requirement is fulfilled [22]. A visible sign of endodormancy release is the ability of buds to develop and finally to bloom, which was studied in a climate chamber experiment, to detect t_1_. Ecodormancy keeps the reproductive organs biologically silent until spring, when air temperature rises and the water content in the buds increases. For the climate conditions in NE Germany, this third phase lasts on average 82 ± 16.6 days and ends with the beginning of ontogenetic development (t_1_*, beginning of phase 4). The continuously rising water content after t_1_* was a clear sign of the resumption of biological activity and was related to rising temperatures in early spring [17]. On average, 53 ± 10.6 days after t_1_*, the buds started to bloom. Finally, after pollination and fruit set, cherries developed in 71 ± 13.8 days until picking ripeness at the end of June. 

Here, this untargeted approach (global metabolite profiling) was the first step in an exploratory study to identify metabolites, which could later be used as suitable biomarkers, indicating the transition between phenological phases, such as endo- and ecodormancy, and the following stage of ontogenetic development. 

This study aims to identify biochemical changes that accompany developmental transitions of cherry buds (*Prunus avium* L., cv. ‘Summit’). Since not all phase transitions are accompanied by visual changes in the tree, prediction of its timing relies on mathematical models. Thus, identifying biomarkers that can reliably indicate the transition between phenological phases will improve phenological modelling substantially. This would allow for developing physiologically justified model approaches, instead of statistical ones. In addition, the course of the metabolites provides information about the metabolism in the buds during different development phases (Figure 1).

## 2. Results

### 2.1. Metabolites in Cherry Buds, Identified by Global Profiling

As a result of the global metabolite profiling, 445 named metabolites were found in sweet cherry buds which can be assigned to amino acids, carbohydrates, phytohormones, lipids, nucleotides, peptides, and some secondary metabolites (Table S1, Figure S1). 

The number of metabolites which were markedly differently (*p* ≤ 0.05) between the phenological phases (1/2), (2/3) and (3/4) were 217, 208, 390, respectively, where 114, 119, 338 metabolites increased and 103, 89, 52 metabolites decreased, indicating anabolic as well as catabolic activity over the observed phases in the buds (Table 1). Between the individual dormancy phases (1/2, 2/3, 1/3), the smallest number of metabolites significantly changed, whereby the lowest number (*n* = 208) was found in the transition from endo- to ecodormancy (phase 2/3). Among the three dormancy phases and the beginning of ontogenetic development (phases 1/4, 2/4, 3/4), always more than 300 metabolites significantly increased (305, 323, 338), while a distinctly lower number of metabolites decreased (57, 46, 52). This clearly supports the resumption of bud development after t_1_*, which can be seen in Figure S1 in the predominantly red colours (high intensity) after t_1_*. 

A differentiated picture emerged for the change in metabolites between phenological stages (Table 2). The number of metabolites which differ significantly (*p* ≤ 0.05) was 85 and 94 between ‘total leaf fall’ (LF) and endodormancy release (t_1_), and between t_1_ and beginning of ontogenetic development (t_1_*), respectively. In this context, between LF and t_1_, 19% of the metabolites increased, and 81% of them decreased, whereas the opposite took place between t_1_ and t_1_*, with 89% and 11%. This indicates the cessation of growth and bud development between the two stages, LF and t_1_, marking the endodormancy phase, and its resumption with the beginning of ontogenetic development (t_1_*). Thus, the number of significantly changing metabolites further increased between t_1_* and ‘swollen bud’ (SB) to 158 metabolites, of which 95% increased and only 5% decreased. During this phase, the water content markedly rose from 54% during ecodormancy (t_1_-t_1_*), and 55% at t_1_*, to 66% at SB in the 2015/16 study season. This rising water content, starting at t_1_*, was also found in the same magnitude for other study years [17], and confirms the start of biological activity in the buds. For the development stages after SB, the number of metabolites that increased in intensity (76–97%) was clearly higher than those that decreased. For the very short phases ‘swollen bud’/’side green’ (SB/SG) and ‘tight cluster’/’open cluster’ (TC/OC), only a few metabolites showed marked changes. In this case, the time was too short to cause any significant changes in the metabolite intensity.

### 2.2. Metabolites of Glycolysis and TCA Cycle

Glycolysis is one of the major catabolic pathways for the breakdown of carbohydrates, down to the tricarboxylic acid (TCA) cycle, to supply energy for different processes within the cell [23]. For this reason, these metabolites and intermediates were first considered. They could indicate the winter dormancy period, through downregulated intensity and later, during growth and development, the transition between the different development stages.

**Figure 2:**
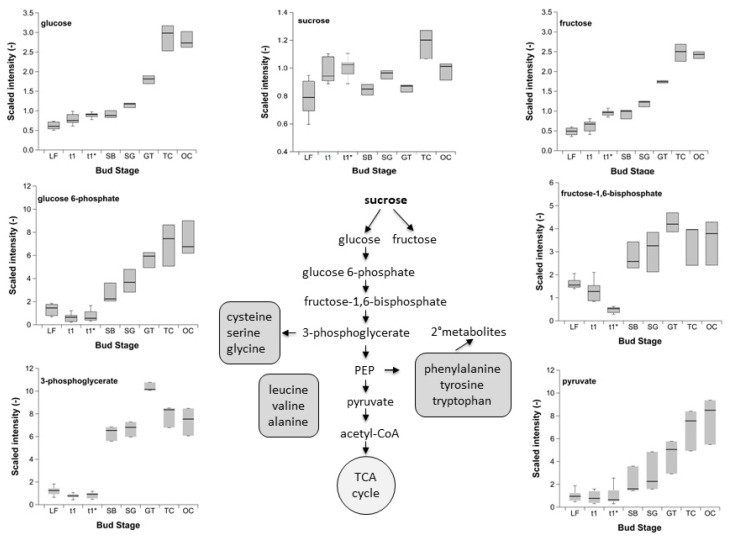
Intensity of sucrose, glucose, fructose and selected intermediates of glycolysis in sweet cherry buds cv. ‘Summit’. ‘Total leaf fall’ (LF), endodormancy release (t_1_), beginning of ontogenetic development (t_1_*) ‘swollen bud’ (SB), ‘side green’ (SG), ‘green tip’ (GT), ‘tight cluster’ (TC), and ‘open cluster’ (OC). Sample size for the stages LF, t_1_, t_1_* (*n* = 6), for the stages between SB and OC (*n* = 3).

The intensity of glucose (Figure 2) was similar among LF and SB, day of year (DOY) 307 (2015) and DOY 89 (2016), respectively, displayed a long period of 147 days. Afterwards the intensity rose stepwise from SG to TC and OC, and both developmental stages had similar intensity. The first adenosine triphosphate (ATP) consuming step of glycolysis is the phosphorylation of glucose to glucose 6-phosphate (G6P). This metabolite had a low and unchanged intensity between LF and t_1_*, representing 119 days. During the growth stages assigned to the visible ontogenetic development, the intensity of G6P raised linearly from SB onward until TC. Step seven of glycolysis is generating 3-phosphoglycerate (3PG), and thereby ATP, a further intermediate of the pathway to produce pyruvate. As shown for G6P, the intensity of 3PG between LF and t_1_* was also low and unchanged. For this intermediate, the increase in the water content of the buds also leads to a marked increase, with a clear maximum of the intensity at ‘green tip’ (GT), compared to SB, SG, TC and OC. It is well known that 3PG can also act as a precursor for the amino acid serine, from which it can build cysteine and glycine. One molecule of pyruvate and one molecule of ATP are the result of the transfer of phosphate from phosphoenolpyruvate (PEP) to adenosine diphosphate (ADP), the final reaction of glycolysis. The downregulation, as shown for G6P and 3PG, was also true for pyruvate, visible as low and unchanged intensity from LF until t_1_*. During the later development stages, the intensity of pyruvate rose in parallel. 

Phosphorylation of fructose 6-phosphate led to a further intermediate of glycolysis, the fructose 1,6-bisphosphate (F1,6-BP), from which 3PG can be generated. F1,6-BP shows a clear depletion of intensity from LF until the minimum intensity at t_1_*. Later, during the ontogenetic development, the pattern of F1,6-BP corresponds well to that of 3PG, but at a lower level of intensity. 

The presence of organic acids, particularly TCA cycle intermediates, in plants is known to support numerous and diverse functions within and beyond cellular metabolism [24]. However, the level of accumulation of the various organic acids is variable between species, development stages and tissue types. This suggest that enzymes involved in the interconversion of these metabolic intermediates are subject to regulatory control.

**Figure 3:**
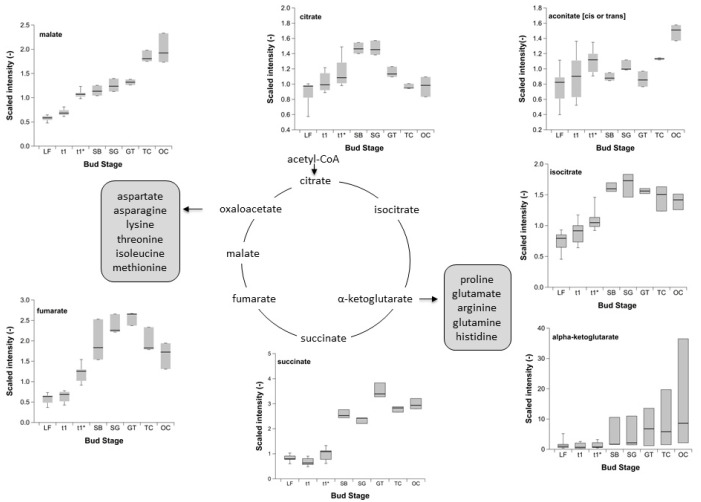
Intensity of selected TCA cycle intermediates in sweet cherry buds cv. ‘Summit’. ‘Total leaf fall’ (LF), endodormancy release (t_1_), beginning ontogenetic development (t_1_*) ‘swollen bud’ (SB), ‘side green’ (SG), ‘green tip’ (GT), ‘tight cluster’ (TC), and ‘open cluster’ (OC). Sample size for the stages LF, t_1_, t_1_* (*n* = 6), for the stages between SB and OC (*n* = 3).

The first step of the TCA cycle, which takes place in the mitochondria, known as the ‘powerhouse of the cell’, a membrane-bound organelle found in the cytoplasm, is binding acetyl-CoA to oxaloacetate (Figure 3). After subsequent hydrolysis citrate is formed. The intensity of citrate is markedly lower at LF, t_1_ and t_1_*, compared to a maximum intensity at SB and SG, and later comparable to the stages GT, TC and OC. The isomerization of citrate yields then *cis*-aconitate and isocitrate (Figure 3). The importance of this step lies in the conversion of a difficult-to-oxidize tertiary alcohol (citrate) into an easy-to-oxidize secondary alcohol (isocitrate). The intensity of cis-aconitate was almost identical over the observation period, with one exception at OC, with a higher intensity. The intensity of the intermediate isocitrate can be assigned to two groups, among the endo- and ecodormancy phase, and to the bud development-related stages SB to OC. In a further step, isocitrate is oxidized and decarboxylated. In addition to the first reduction equivalent NADH, α-ketoglutarate is formed. 

The hydrolysis of the high-energy thioester succinyl-CoA, catalysed by succinyl-CoA synthetase, leads to succinate. Its intensity was well below the intensity during bud development, with a maximum at GT, compared to SB, SG, TC and OC. In a further step of the TCA cycle, succinate is oxidized to fumarate. At LF and t_1_, the intensity of fumarate is below that of t_1_*, increases afterwards and shows a similar pattern as citrate, with a decreasing tendency compared to the increasing bud development. The addition of water to the double bond of the fumarate then creates malate. A low intensity also applies to this intermediate, which increases in an almost linear form from here onwards until TC and OC.

### 2.3. Selection of Potential Metabolites That Indicate Dormancy Phases

**Figure 4:**
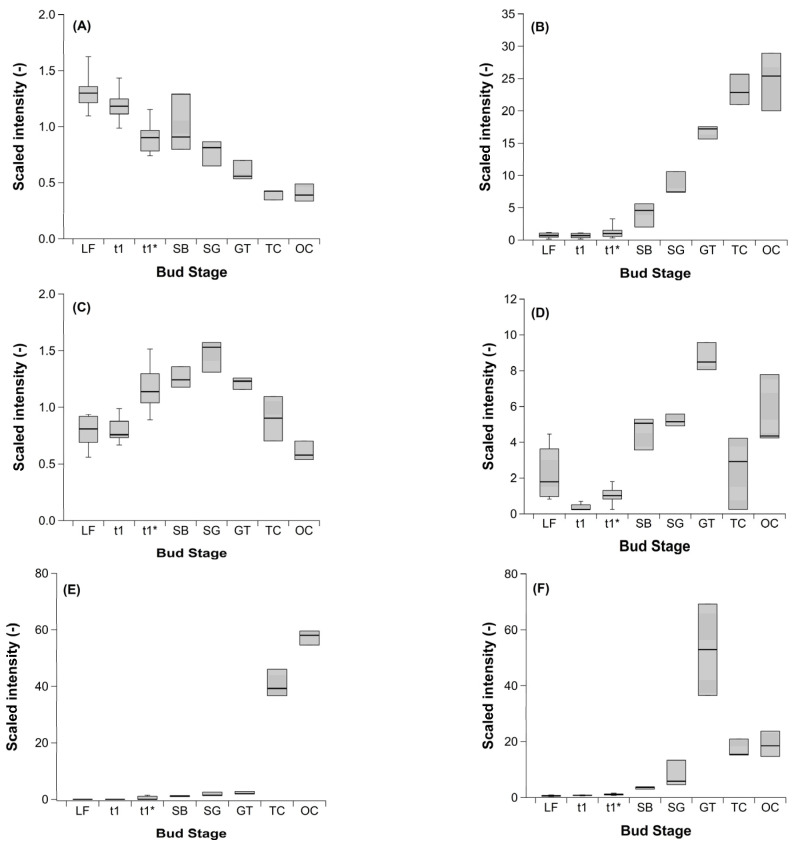
Selected examples of ‘data trends’ in the untargeted profiling of sweet cherry buds cv. ‘Summit’. ‘Total leaf fall’ (LF), endodormancy release (t_1_), beginning ontogenetic development (t_1_*) ‘swollen bud’ (SB), ‘side green’ (SG), ‘green tip’ (GT), ‘tight cluster’ (TC), and ‘open cluster’ (OC). Sample size for the stages LF, t_1_, and t_1_* (*n* = 6), for the stages between SB and OC (*n* = 3). (**A**) steady decrease: catechin, (**B**) steady increase: 1-linolenoylglycerol (18:3), (**C**) steady rise and decline: 1-linolenoyl-digalactosylglycerol (18:3), (**D**) varies with growth stage: gamma-glutamylvaline, (**E**) undetectable, low level, high level: cytidine 2′ or 3′-monophosphate (2′ or 3′-CMP), (**F**) peak at one stage: 2,3-dihydroxyisovalerate.

A methodological challenge arose from the fact that the untargeted global profiling showed different data trends (Figure 4 and Figure S2).

A steady decrease, a steady increase, and a steady rise and decline (Figure 4A–C) are here shown exemplarily by catechin, a polyphenol related to the flavonoids with antioxidative potential, 1-linolenolylglycerol (18:3), 1-linolenolyldigalactosylglycerol (18:3), respectively. Examples of trends which ‘vary with growth stage’, ‘undetectable low level, high level’, and ‘peak at one stage’ (Figure 4D–F) include gamma-glutamylvaline, cytidine-2′ or 3′-monophosphate (2′ or 3′-CMP), a nucleotide that is used as a monomer in RNA, and 2,3 dihydroxyisovalerate, which functions as an intermediate of the valine biosynthesis pathway, respectively. These types of data trend are not suitable to display transitions of the phenological phases endo- and ecodormancy, indicated by the three important stages, LF, t_1_ and t_1_*. 

Another important aspect in the selection of suitable metabolites is the examination of these candidates for environmental control, for instance by air temperature. However, this test is only possible if the course of metabolites is available over several years, i.e., after targeted profiling. For phenological modelling, those metabolites would be of particular interest, because they could allow one to model the unobservable stages t_1_ and t_1_* according to environmental parameters. At this point, it must be pointed out again that the timing of stages t_1_ and t_1_* must be known in order to select relevant metabolites for modelling purposes. Conversely, precisely timed changes in the intensity of the metabolites at t_1_ and t_1_* can confirm the experimentally determined dates.

**Figure 5:**
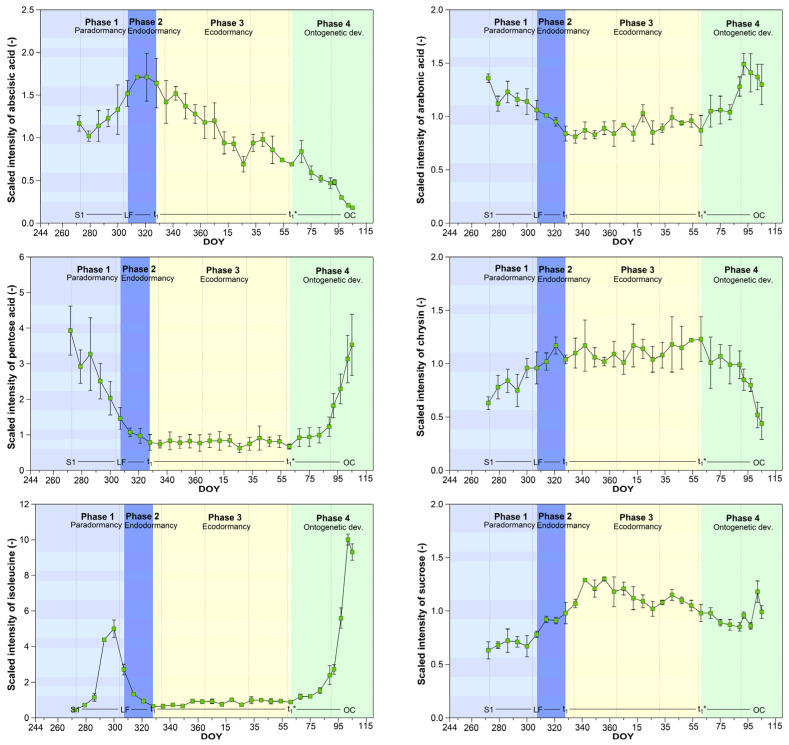
Pattern of mean intensity of abscisic acid, arabonic acid, pentose acid, chrysin, isoleucine and sucrose in the season 2015/16. Phase 1: end of paradormancy (first sampling S1-‘total leaf fall’ LF), phase 2: endodormancy (LF-endodormancy release t_1_), phase 3: ecodormancy (t_1_-beginning of ontogenetic development t_1_*), phase 4: first stage of ontogenetic development (t_1_*-‘open cluster’ OC). Error bars show the standard deviation (*n* = 3), DOY, day of year.

Thus, the identification of suitable metabolites continues to be a challenge. Neither its absolute intensity nor its abundance in the flower buds are suitable selection criteria. These markers can also be intermediate products that only occur in small intensities. For this reason, we explored the time course of all 445 metabolites during endo- and ecodormancy as well as at the beginning of ontogenetic development detailed (Figure S2). We analysed significant differences in their intensity between the phenological stages, including the unobservable stages and phenological phases (Table 1 and Table 2). By this means, it was possible to identify some metabolites that show significant changes at the key stages (Figure 5). 

Metabolites, which we finally suggested for targeted profiling over nine years, included the major phytohormone abscisic acid (ABA) and additionally abscisic acid glucosyl ester (ABA-GE, not shown here), which contributes to the maintenance of ABA homeostasis, arabonic acid, obtained by oxidation of arabinose, dextrose, or levulose (stereoisomer of fructose) and used in synthesizing riboflavin, pentose acid, any of the various nucleic acids yielding a pentose on hydrolysis especially RNA, chrysin, a member of the flavonoids, isoleucine, an branched-chain aliphatic amino acid, which influenced the number of meristem cells and epidermal cell length and sucrose, the cryoprotective compound which prevents buds from freezing during winter [25,26]. All of these metabolites showed clear trend changes at the interesting stages, t_1_ and t_1_*. Apart from ABA, all of these metabolites display almost constant values during ecodormancy, indicating reduced biological activity during this relatively long-lasting winter phase at our experimental site. ABA was the only metabolite which steadily decreased between t_1_ and open cluster, and chrysin was the only one which decreased after t_1_*, while arabonic acid, pentose acid and isoleucine then rose. Metabolites which show an ‘u-shape’ (arabonic acid, pentose acid, isoleucine) or its inverse form (chrysin, sucrose) between LF and OC can be key markers which allow us to confirm the previously found dates for t_1_ and t_1_*.

## 3. Discussion

This global metabolite profiling for the 2015/16 season was the first step in the identification of metabolites which occur in cherry flower buds during winter dormancy and ontogenetic development. The aim was to detect key metabolites which could be able to confirm our previously found dates in the timing of the visible (LF, SB, GT, etc.) and unobservable stages (t_1_, t_1_*) of bud development for ‘Summit’ [17]. The season used for this untargeted profiling showed no extreme timings of phenological events related to the 2011/12–2019/20 seasons. From 445 metabolites found in cherry flower buds, only 64% of them showed significant changes in their intensity among the dormancy phases. Large changes were found between the dormancy phases and ontogenetic phase. The increase in intensity clearly exceeded that of the decrease, indicating the resumption of growth and development after winter rest. These finding were clearly confirmed by the metabolites which are involved in the glycolysis and TCA cycle. 

The selected intermediates of glycolysis (Figure 2) G6P, 3PG, F1,6-BP and pyruvate, had a very low and minimum intensity between LF and t_1_*, compared to the later growth stages between SB and OC. This clearly indicates that the energy demand in sweet cherry buds during endo- and ecodormancy is downregulated, and reflects the view of the so called ‘winter rest’. Indeed, these intermediates can also have a function as building blocks for anabolism and can play an important role in adaptation to environmental factors like cold or freezing temperatures. Plaxton [27] gave an excellent and detailed overview of the glycolysis. It was stated that glycolysis is an example of how a ubiquitous metabolic pathway can show significant differences in terms of its roles, structure, regulation, and compartmentation in plant species and the various mechanisms of metabolic regulation, applied to a specific pathway in vivo. During the ontogenetic development of the buds, the breakdown of glucose during glycolysis provides the needed chemical energy (ATP or NADH), reductant, and pyruvate, through the sequence of enzymatically catalysed reactions, which is seen by the increasing intensity of the intermediates. The magnitude of metabolite flux through any metabolic pathway depends upon the activities of the individual enzymes involved. ‘Coarse’ and/or ‘fine’ metabolic controls can vary the reaction velocity of a particular enzyme in vivo. Coarse control of enzymes is achieved through varying the total of enzyme molecules via alterations in the rates of enzyme biosynthesis or proteolysis. It most frequently comes into play during tissue differentiation or environmental changes [27]. In summary, for the glycolysis, the intermediates during winter rest are downregulated to a minimum. The following ontogenetic development of cherry buds, starting with SB until OC, was understandably closely linked to the demand and increase in the energy metabolism, and therefore to the intensity of intermediates of the glycolysis.

As shown for intermediates of glycolysis, the intensity of the organic acids of the TCA cycle (citrate, isocitrate, α-ketoglutarate, succinate, fumarate, malate; Figure 3) was different at endo- and ecodormancy, compared to stages of ontogenetic development. The TCA cycle is a sequence of catabolic reactions that support ATP synthesis in the mitochondria, but at the same time it is embedded in a wider metabolic network that allows TCA cycle activity to contribute to other aspects of metabolism ([28] and references therein). The level of accumulation of the various organic acids are extremely variable between species, developmental stages and tissue types, providing further support for the hypothesis that the enzymes involved in the interconversion of these metabolic intermediates are subject to regulatory control ([28] and references therein).

In summary, it can be stated that, during the so-called winter rest, specifically the phases of endo- and ecodormancy, the energy metabolism in the form of glycolysis and TCA cycle is shut down to a minimum. The ontogenetic development is closely related to the upregulation of the carbohydrate metabolism and thus with the generation of energy for growth and development of sweet cherry buds. None of the intermediates are suitable for determining simultaneously the phase transitions of endo- and ecodormancy.

Only six metabolites were found which confirm the timing of the unobservable stages t_1_ and t_1_*. Except for ABA, all metabolites showed no significant changes in their intensity during ecodormancy. Both, high levels (e.g., sucrose, chrysin) and low levels (pentose acid, isoleucine) occurred during this phase. This points to different functions of these metabolites during ecodormancy. Sucrose is known for their cryoprotective potential, not only in *Prunus avium* [25,26,29,30]. This explains its increased intensity during ecodormancy, the main winter period in the study region. The metabolic pathway for isoleucine is widely described in higher plants, but only few references point to its specific role in plant development. For an Arabidopsis mutant with low isoleucine biosynthesis, Yu et al. [31] reported that the partial deficiency of isoleucine alters transcript levels of the genes and encoding enzymes, involved in branched-chain amino acid synthesis and glycosylates metabolic pathways. The consequences were a markedly lower meristem cell number and epidermal cell length in roots. Deficiency symptoms could be reversed by exogenously applied isoleucine. Therefore, it has to be proved whether isoleucine might also play a role in different growth phases over nine years or if correlations with environmental parameters exist. To our knowledge, the function of chrysin and pentose acid is still unclear and requires further investigation. It will be very interesting to see if the course of these metabolite will be confirmed in the other seasons.

The reason why we selected ABA as a key metabolite it is not only its increase until t_1_ and its following decrease until OC; ABA is also increasingly confirmed as having a function in the induction and maintenance of dormancy in fruit tree buds [13,32,33,34], including cherries [9,25]. In this context, the ABA-GE content (not shown here) is also relevant and was therefore selected as an additional key metabolite [9,35]. With this knowledge, in the next step the selected metabolites will be analysed, as part of a targeted profiling over a period of nine observation years (2011/12–2019/20), in order to either confirm or reject their suitability as markers which indicate the timing of endodormancy release (t_1_) and/or the beginning of ontogenetic development (t_1_*). Both stages limit the ecodormancy phase—a period that needs to be studied much more intensively in phenological models than at present [36]. To our knowledge, data for the seasonal metabolism of winter dormancy in cherry buds, in a period of 6 to 7 months over nine years, represent a ‘novelty’. This robust dataset can help in elucidating the mechanisms and biochemical pathways associated with the dormancy phases of cherries. The targeted metabolite profiling will reveal whether there is indeed only one key metabolite that reflects the phases well, or whether multiple metabolites must be used for their explanation. Results of the targeted profiling will be published in a separate paper, including an extensive discussion of all key metabolites related to their role during dormancy and ontogenetic development. 

In recent years, genomic analyses have also been increasingly used to confirm the timing of relevant stages of cherry bud development during winter and spring. Recently, Vimont et al. [2] used transcriptomic analysis to predict its timing by seven relevant genes. However, our study additionally illustrates the course of potential metabolites which are induced by gene expression. Thus, it is still necessary to combine both methods in order to establish a causal relationship between these levels of dormancy control [9,15].

## 4. Materials and Methods

### 4.1. Experimental Site

The untargeted metabolite profiling was conducted from flower bud samples from the 2015/16 season at the experimental sweet cherry orchard at Berlin-Dahlem (52.47° N, 13.30° E, h = 51 m a.s.l.). The orchard (980 m^2^) comprises 80 cherry trees (cultivars ‘Summit’, ‘Regina’ and ‘Karina’, all grafted onto Gisela-5 rootstocks). The plantation was established in the autumn of 1999. All trees were planted in the N–S direction. In each of the eight rows, ten trees were planted at a distance of 4 m × 3.5 m. The main soil type is parabrown soil with weak marks of pale soil (*Albic Luvisol*). Surface soil is a silty to medium-loamy sand which becomes a silty-loamy sand to sandy clayey loam in deeper layers. The long-term (1991–2020) average air temperature and precipitation were 10.4 °C and 562 mm, respectively. Irrigation, fertilisation and pruning of the trees were performed on demand. Samples were taken from the cv. ‘Summit’ only. 

### 4.2. Sampling of Sweet Cherry Buds

To analyse metabolites in cherry buds, from the end of September (272 DOY) until the middle of April (105 DOY) 2015/16 (altogether, 31 sampling dates), 3 flower-bud cluster per tree, randomly over 3 trees (replications), were taken each week between 10 and 12 a.m. After the beginning of ontogenetic development (t_1_*, 61 DOY), sampling was performed in a development-related manner at ‘swollen bud’ (SB, 89 DOY), ‘side green’ (SG, 92 DOY), ‘green tip’ (GT, 97 DOY), ‘tight cluster’ (TC, 102 DOY) and ‘open cluster’ (OC, 105 DOY). After cutting, clusters were immediately placed in plastic bags in a polystyrene box on ice, frozen in liquid nitrogen, freeze dried, subsequently ground in a ball mill (Retsch M1, Haan, Germany) and stored at −80 °C until analysis. 

In order to determine the beginning of ontogenetic development (t_1_*), additional flower buds were sampled between September and April to determine the bud’s water content. During ecodormancy, this value was almost constant at 54%, and only began to rise continuously at the end of winter, indicating the beginning of ontogenetic development [17]. 

### 4.3. Sampling of Twigs

In order to determine the date of endodormancy release (t_1_) experimentally, 2 multi-branched twigs from selected ‘Summit’ trees (length 20–30 cm) were cut off weekly in November and December. After cutting, twigs were placed in 500 mL plastic flasks, filled with water to observe the beginning of blossom in a climate chamber with 12 h light, temperatures of ~20/~15 °C (day/night) and 70% relative humidity. Buds were observed daily to determine the time of bud burst (BBCH 53) and beginning of blossom (BBCH 60) [37,38]. When twigs started to bloom under controlled conditions, this was the indication that the chilling requirement at sampling was fulfilled in the orchard. We assumed that the chilling requirement was sufficient if 3–4 flowers per twig completely opened (BBCH 60) on the first and the following samplings. 

### 4.4. Global Metabolite Profiling

Global metabolite profiling was conducted by Metabolon Inc., 617 Davis Drive, Morrisville, NC 27560 (www.metabolon.com, accessed on 7 February 2022). The final report contains information on sample preparation, UPLC-MS/MS methodology, data extraction and compound identification (Supplementary file S1).

### 4.5. Statistical Methods

Welch’s two-sample t-test was used to test whether two unknown means are different from two independent populations. It can be used for unequal variances and has an approximate t-distribution with degrees of freedom, estimated using Satterthwaite’s approximation. We used a two-sided test, testing whether the means are different. For statistical significance testing, *p*-values are given. The lower the *p*-value, the more evidence exists that the null hypothesis (H_0_: two population means are equal) is not true. 

The timing of the stages LF, t_1_, t_1_* cannot be exactly assigned to one week, meaning that we also included the data from the previous and following week (*n* = 6). This explains the larger error bars for these three stages in Figure 2, Figure 3 and Figure 4. The dates for the stages between SB and TC are precise enough to relate them to a single date, so that the sample size is *n* = 3 (replications).

## Figures and Tables

**Table 1:** Number (*n*) of metabolites in sweet cherry buds, cv. ‘Summit’, which significantly changed between phenological phases in the 2015/16 season (Welch’s two-sample t-Test; *p* ≤ 0.05). Phase 1: end of paradormancy (S1-LF), phase 2: endodormancy (LF-t_1_), phase 3: ecodormancy phase (t_1_-t_1_*), phase 4: first stage of ontogenetic development (t_1_*-OC). First sampling (S1, DOY 272), ‘total leaf fall’ (LF, DOY 307), endodormancy release (t_1_, DOY 328), beginning of ontogenetic development (t_1_*, DOY 61), ‘open cluster’ (OC, DOY 105). DOY, day of year.

Phenological Phase	Phase ½ S1-LF/LF-t_1_	Phase 2/3 LF-t_1_/t_1_-t_1_*	Phase 1/3 S1-LF/t_1_-t_1_*	Phase 1/4 S1-LF/t_1_*-OC	Phase 2/4 LF-t_1_/t_1_*-OC	Phase 3/4 t_1_-t_1_*/t_1_*-OC
*n*	217	208	288	362	369	390
increase	114	119	159	305	323	338
decrease	103	89	129	57	46	52

**Table 2:** Number (*n*) of metabolites in sweet cherry buds, cv. ‘Summit’, which significantly changed between phenological stages in the 2015/16 season (Welch’s two-sample t-Test; *p* ≤ 0.05). ‘Total leaf fall’ (LF), endodormancy release (t_1_), beginning of ontogenetic development (t_1_*) ‘swollen bud’ (SB, DOY 89), ‘side green’ (SG, DOY 92), ‘green tip’ (GT, DOY 97), ‘tight cluster’ (TC, DOY 102), ‘open cluster’ (OC). DOY, day of year.

Phenological Stage	LF/t_1_	t_1/_t_1_*	t_1_*/SB	SB/SG	SG/GT	GT/TC	TC/OC
Days between stages (2015/16)	21	98	28	3	5	5	3
*n*	85	94	158	39	171	186	49
increase|decrease in total	16|69	84|10	150|8	38|1	142|29	141|45	38|11
increase|decrease in %	19|81	89|11	95|5	97|3	83|17	76|24	78|22

## Data Availability

Output of the metabolite identification are available on reasonable request to the corresponding author.

## Supplementary Materials

The following supporting information can be downloaded at: https://www.mdpi.com/article/10.3390/metabo12030247/s1, Table S1: List of 445 metabolites found in sweet cherry buds (cv. ‘Summit’) in the 2015/16 season, Figure S1: Heatmap of the scaled intensity for 445 metabolites during 4 phases of sweet cherry bud development (cv. ‘Summit’) in the 2015/16 season. Phase 1: End of paradormancy, Phase 2: Endodormancy, Phase 3: Ecodormancy, Phase 4: First stage of ontogenetic development, Figure S2: Pattern of the scaled intensity for 445 metabolites during 4 phases of sweet cherry bud development (cv. ‘Summit’) in the 2015/16 season. Phase 1: End of paradormancy, Phase 2: Endodormancy, Phase 3: Ecodormancy, Phase 4: First stage of ontogenetic development, Supplementary file S1: Extraction of Metabolon’s Report (Global Metabolite Profiling).
