# Supplementary material for: Metabolites in Cherry Buds to Detect Winter Dormancy
Source: Metabolites. 2022 Mar 16;12(3):247. doi: 10.3390/metabo12030247 (PMC8951522; doi:10.3390/metabo12030247)
Supplement: Supplementary file 1 [file metabolites-12-00247-s001.zip › metabolites-1609763-supplementary/Figure S1.pdf]

Figure S1: Heatmap of the scaled intensity for 445 metabolites during 4 phases of sweet cherry bud devel-opment (cv. ‘Summit') in the 2015/16 season. Phase 1: End of paradormancy, Phase 2: Endodormancy, Phase 3: Ecodormancy, Phase 4: First stage of ontogenetic development

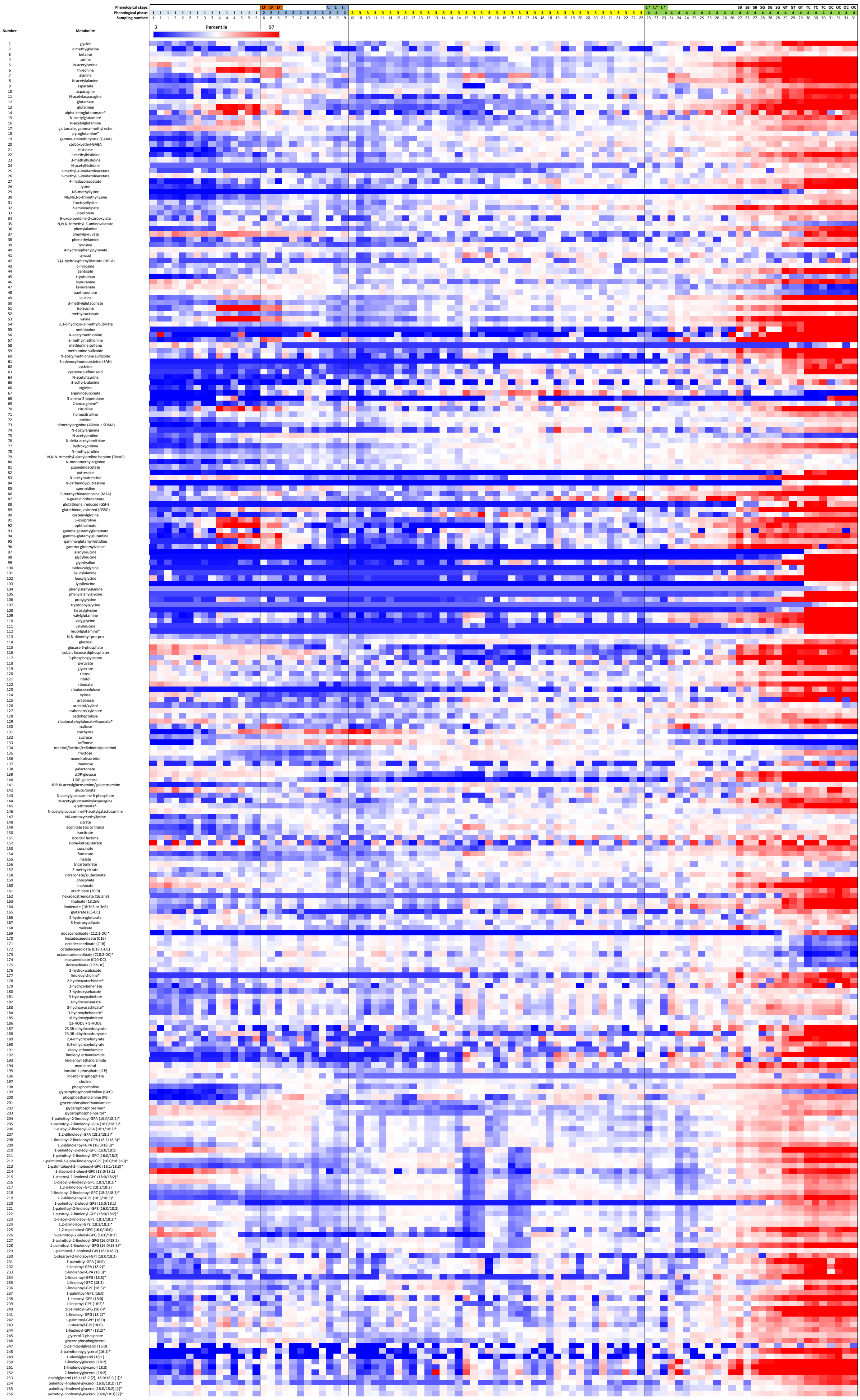

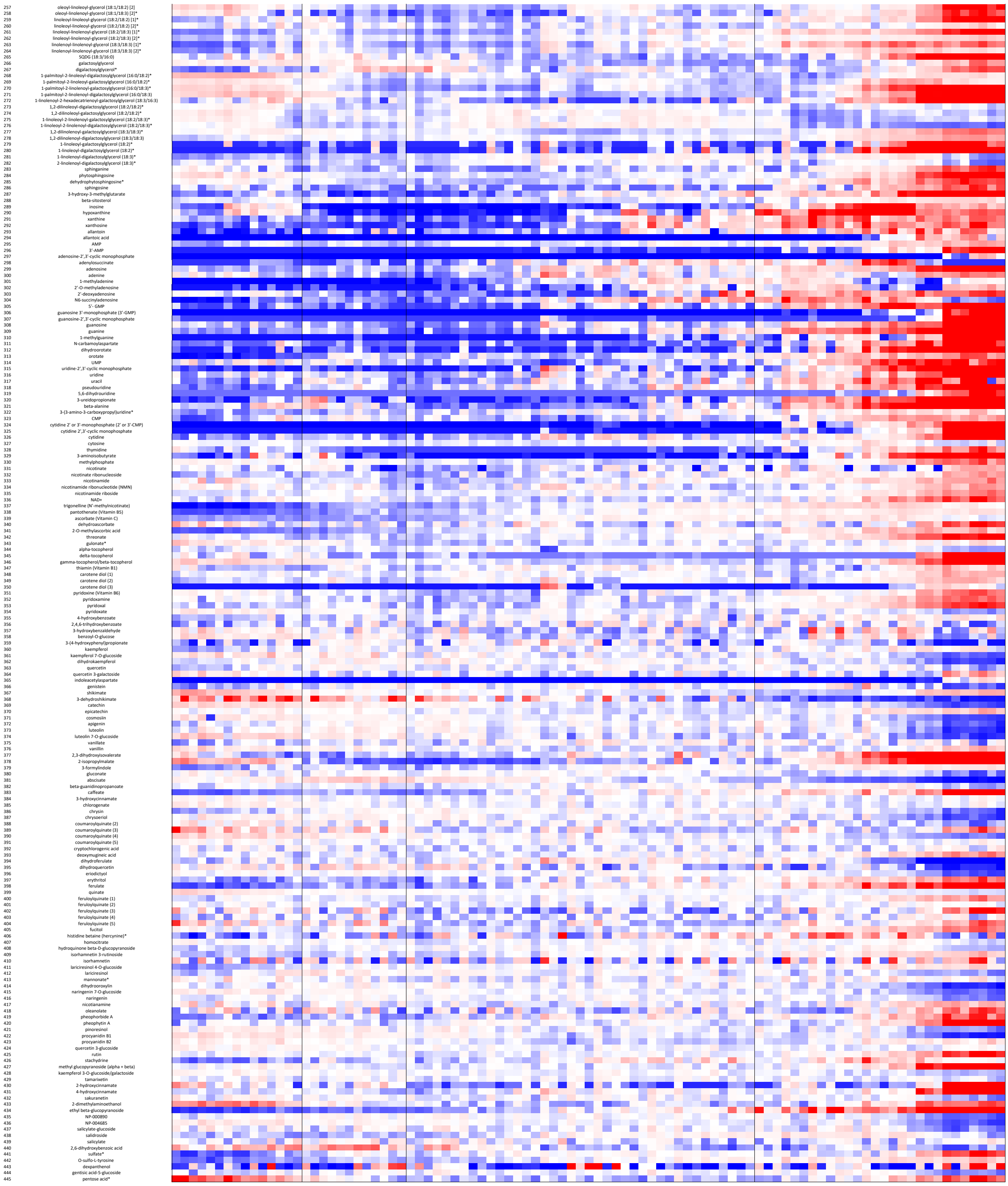
257 oleoyl-linoleoyl-glycerol (18:1/18:2) [2]
258 oleoyl-linolenoyl-glycerol (18:1/18:3) [2]*
259 linoleoyl-linoleoyl-glycerol (18:2/18:2) [1]*
260 linoleoyl-linoleoyl-glycerol (18:2/18:2) [2]*
261 linoleoyl-linolenoyl-glycerol (18:2/18:3) [1]*
262 linoleoyl-linolenoyl-glycerol (18:2/18:3) [2]*
263 linolenoyl-linolenoyl-glycerol (18:3/18:3) [1]*
264 linolenoyl-linolenoyl-glycerol (18:3/18:3) [2]*
265 SQDG (18:3/16:0)
266 galactosylglycerol
267 digalactosylglycerol*
268 1-palmitoyl-2-linoleoyl-digalactosylglycerol (16:0/18:2)*
269 1-palmitoyl-2-linoleoyl-galactosylglycerol (16:0/18:2)*
270 1-palmitoyl-2-linolenoyl-galactosylglycerol (16:0/18:3)*
271 1-palmitoyl-2-linolenoyl-digalactosylglycerol (16:0/18:3)
272 1-linolenoyl-2-hexadecatrienoyl-galactosylglycerol (18:3/16:3)
273 1,2-dilinoleoyl-digalactosylglycerol (18:2/18:2)*
274 1,2-dilinoleoyl-galactosylglycerol (18:2/18:2)*
275 1-linoleoyl-2-linolenoyl-galactosylglycerol (18:2/18:3)*
276 1-linoleoyl-2-linolenoyl-digalactosylglycerol (18:2/18:3)*
277 1,2-dilinolenoyl-galactosylglycerol (18:3/18:3)*
278 1,2-dilinolenoyl-digalactosylglycerol (18:3/18:3)
279 1-linoleoyl-galactosylglycerol (18:2)*
280 1-linoleoyl-digalactosylglycerol (18:2)*
281 1-linolenoyl-digalactosylglycerol (18:3)*
282 2-linolenoyl-digalactosylglycerol (18:3)*
283 sphinganine
284 phytosphingosine
285 dehydrophytosphingosine*
286 sphingosine
287 3-hydroxy-3-methylglutarate
288 beta-sitosterol
289 inosine
290 hypoxanthine
291 xanthine
292 xanthosine
293 allantoin
294 allantoic acid
295 AMP
296 3'-AMP
297 adenosine-2',3'-cyclic monophosphate
298 adenylosuccinate
299 adenosine
300 adenine
301 1-methyladenine
302 2'-O-methyladenosine
303 2'-deoxyadenosine
304 N6-succinyladenosine
305 5'- GMP
306 guanosine 3'-monophosphate (3'-GMP)
307 guanosine-2',3'-cyclic monophosphate
308 guanosine
309 guanine
310 1-methylguanine
311 N-carbamoylaspartate
312 dihydroorotate
313 orotate
314 UMP
315 uridine-2',3'-cyclic monophosphate
316 uridine
317 uracil
318 pseudouridine
319 5,6-dihydrouridine
320 3-ureidopropionate
321 beta-alanine
322 3-(3-amino-3-carboxypropyl)uridine*
323 CMP
324 cytidine 2' or 3'-monophosphate (2' or 3'-CMP)
325 cytidine 2',3'-cyclic monophosphate
326 cytidine
327 cytosine
328 thymidine
329 3-aminoisobutyrate
330 methylphosphate
331 nicotinate
332 nicotinate ribonucleoside
333 nicotinamide
334 nicotinamide ribonucleotide (NMN)
335 nicotinamide riboside
336 NAD+
337 trigonelline (N'-methylnicotinate)
338 pantothenate (Vitamin B5)
339 ascorbate (Vitamin C)
340 dehydroascorbate
341 2-O-methylascorbic acid
342 threonate
343 gulonate*
344 alpha-tocopherol
345 delta-tocopherol
346 gamma-tocopherol/beta-tocopherol
347 thiamin (Vitamin B1)
348 carotene diol (1)
349 carotene diol (2)
350 carotene diol (3)
351 pyridoxine (Vitamin B6)
352 pyridoxamine
353 pyridoxal
354 pyridoxate
355 4-hydroxybenzoate
356 2,4,6-trihydroxybenzoate
357 3-hydroxybenzaldehyde
358 benzoyl-O-glucose
359 3-(4-hydroxyphenyl)propionate
360 kaempferol
361 kaempferol 7-O-glucoside
362 dihydrokaempferol
363 quercetin
364 quercetin 3-galactoside
365 indoleacetylaspartate
366 genistein
367 shikimate
368 3-dehydroshikimate
369 catechin
370 epicatechin
371 cosmosiin
372 apigenin
373 luteolin
374 luteolin 7-O-glucoside
375 vanillate
376 vanillin
377 2,3-dihydroxyisovalerate
378 2-isopropylmalate
379 3-formylindole
380 gluconate
381 abscisate
382 beta-guanidinopropanoate
383 caffeate
384 3-hydroxycinnamate
385 chlorogenate
386 chrysin
387 chrysoeriol
388 coumaroylquinate (2)
389 coumaroylquinate (3)
390 coumaroylquinate (4)
391 coumaroylquinate (5)
392 cryptochlorogenic acid
393 deoxymugineic acid
394 dihydroferulate
395 dihydroquercetin
396 eriodictyol
397 erythritol
398 ferulate
399 quinate
400 feruloylquinate (1)
401 feruloylquinate (2)
402 feruloylquinate (3)
403 feruloylquinate (4)
404 feruloylquinate (5)
405 fucitol
406 histidine betaine (hercynine)*
407 homocitrate
408 hydroquinone beta-D-glucopyranoside
409 isorhamnetin 3-rutinoside
410 isorhamnetin
411 lariciresinol 4-O-glucoside
412 lariciresinol
413 mannonate*
414 dihydrooroxylin
415 naringenin 7-O-glucoside
416 naringenin
417 nicotianamine
418 oleanolate
419 pheophorbide A
420 pheophytin A
421 pinoresinol
422 procyanidin B1
423 procyanidin B2
424 quercetin 3-glucoside
425 rutin
426 stachydrine
427 methyl glucopyranoside (alpha + beta)
428 kaempferol 3-O-glucoside/galactoside
429 tamarixetin
430 2-hydroxycinnamate
431 4-hydroxycinnamate
432 sakuranetin
433 2-dimethylaminoethanol
434 ethyl beta-glucopyranoside
435 NP-000890
436 NP-004685
437 salicylate-glucoside
438 salidroside
439 salicylate
440 2,6-dihydroxybenzoic acid
441 sulfate*
442 O-sulfo-L-tyrosine
443 dexpanthenol
444 gentisic acid-5-glucoside
445 pentose acid*
