# Supplementary material for: Metabolites in Cherry Buds to Detect Winter Dormancy
Source: Metabolites. 2022 Mar 16;12(3):247. doi: 10.3390/metabo12030247 (PMC8951522; doi:10.3390/metabo12030247)
Supplement: Supplementary file 1 [file metabolites-12-00247-s001.zip › metabolites-1609763-supplementary/Figure S2.pdf]

Figure S2: Pattern of the scaled intensity for 445 metabolites during 4 phases of sweet cherry bud development (cv. ‘Summit') in the 2015/16 season. Phase 1: End of paradormancy, Phase 2: Endodormancy, Phase 3: Ecodormancy, Phase 4: First stage of ontogenetic development

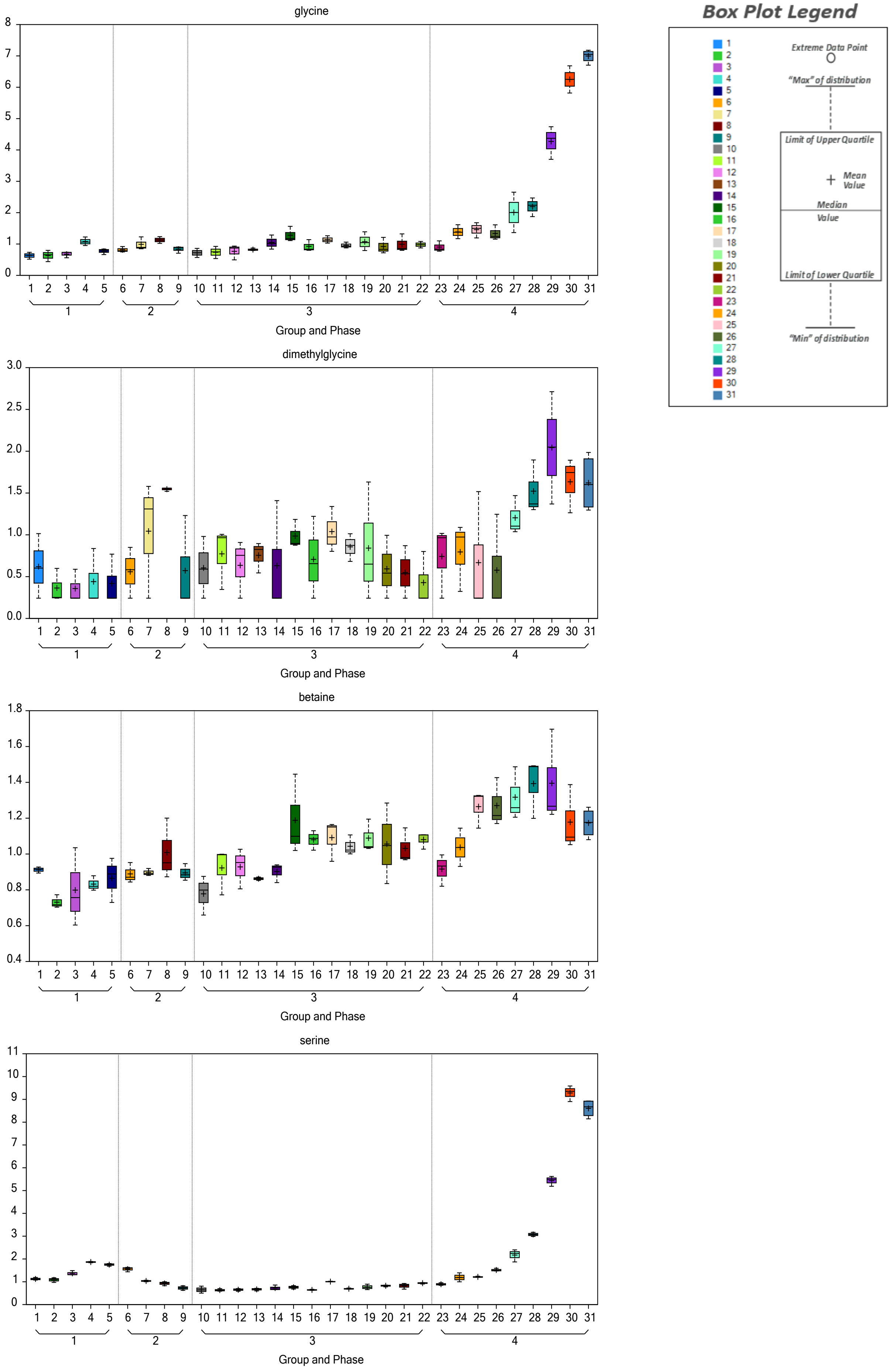

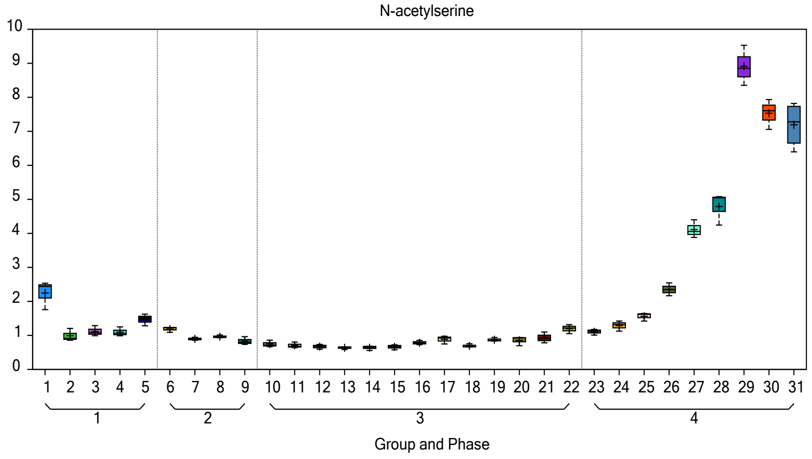
N-acetylserine
0 1 2 3 4 5 6 7 8 9 10
1 2 3 4 5 6 7 8 9 10 11 12 13 14 15 16 17 18 19 20 21 22 23 24 25 26 27 28 29 30 31
1 2 3 4
Group and Phase

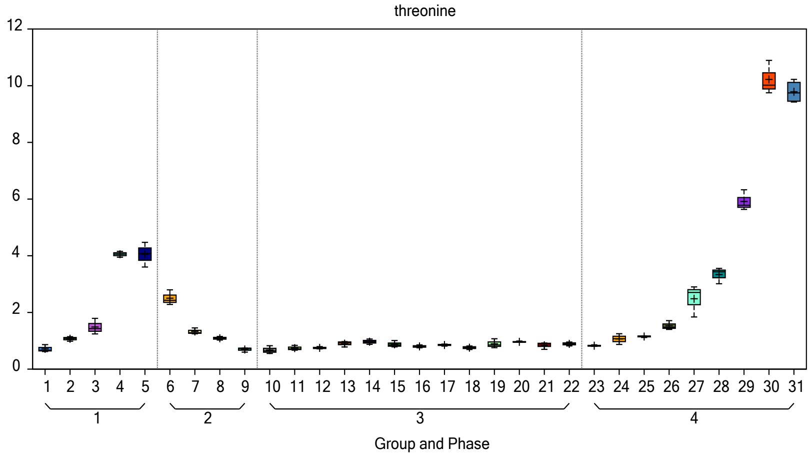
threonine
0 2 4 6 8 10 12
1 2 3 4 5 6 7 8 9 10 11 12 13 14 15 16 17 18 19 20 21 22 23 24 25 26 27 28 29 30 31
1 2 3 4
Group and Phase

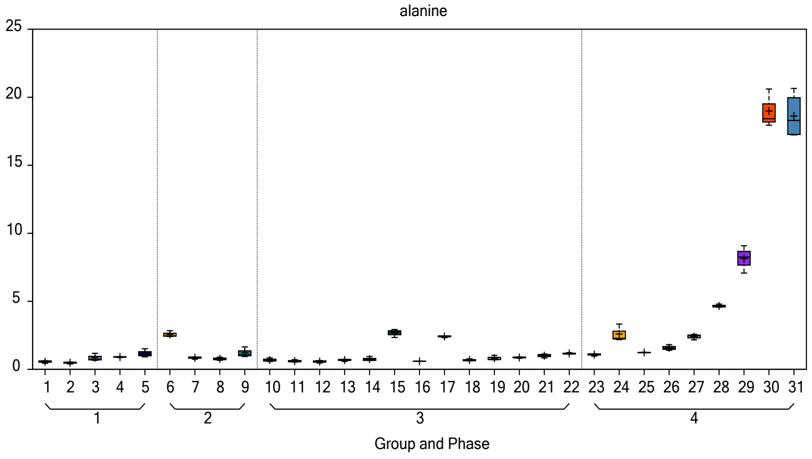
alanine
0 5 10 15 20 25
1 2 3 4 5 6 7 8 9 10 11 12 13 14 15 16 17 18 19 20 21 22 23 24 25 26 27 28 29 30 31
1 2 3 4
Group and Phase

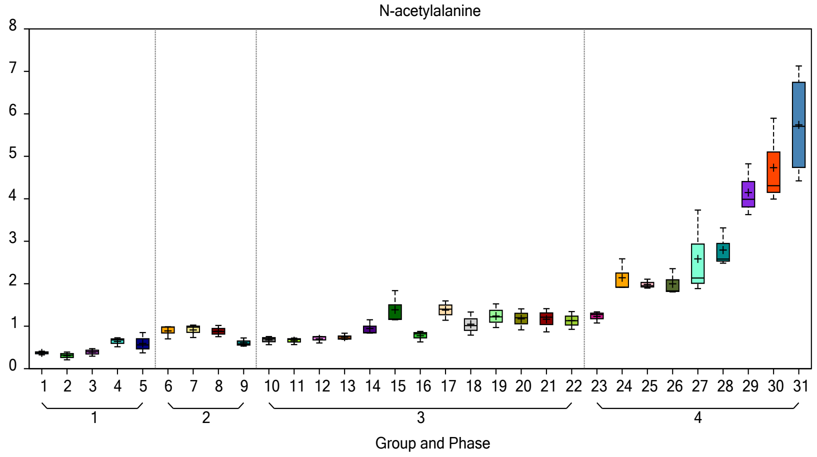
N-acetylalanine
0 1 2 3 4 5 6 7 8
1 2 3 4 5 6 7 8 9 10 11 12 13 14 15 16 17 18 19 20 21 22 23 24 25 26 27 28 29 30 31
1 2 3 4
Group and Phase

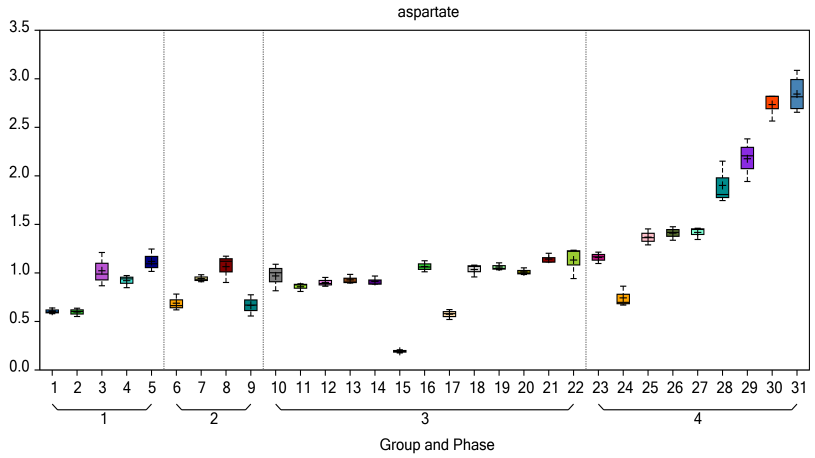
aspartate
3.5
3.0
2.5
2.0
1.5
1.0
0.5
0.0
1 2 3 4 5 6 7 8 9 10 11 12 13 14 15 16 17 18 19 20 21 22 23 24 25 26 27 28 29 30 31
1
2
3
4
Group and Phase

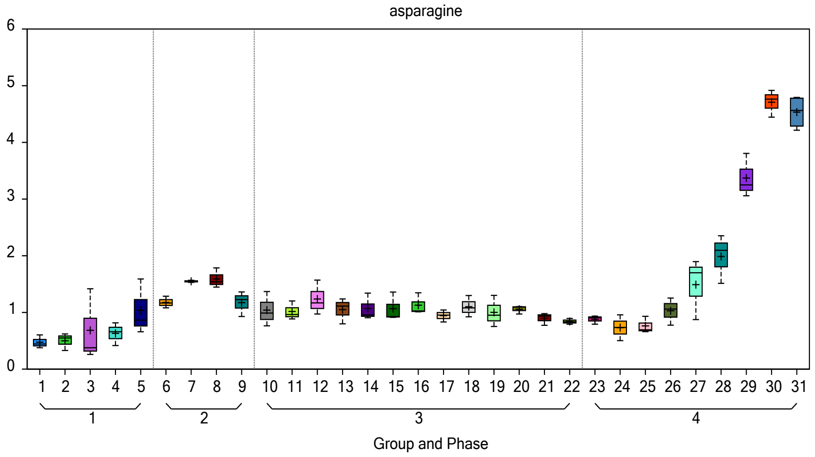
asparagine
6
5
4
3
2
1
0
1 2 3 4 5 6 7 8 9 10 11 12 13 14 15 16 17 18 19 20 21 22 23 24 25 26 27 28 29 30 31
1
2
3
4
Group and Phase

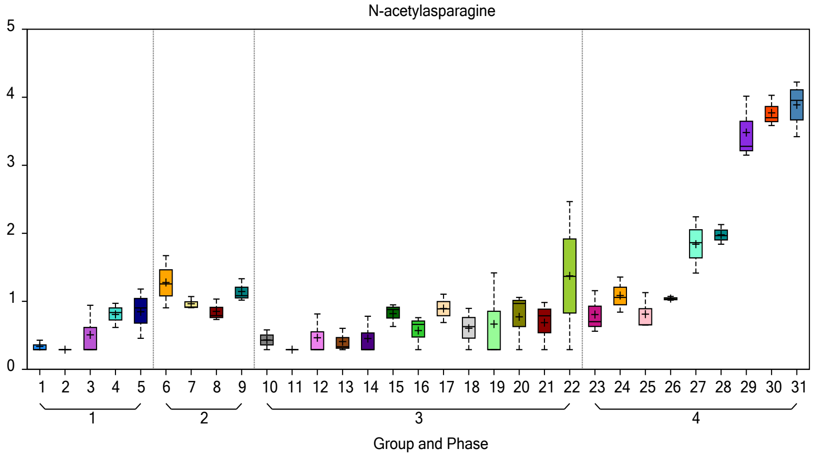
N-acetylasparagine
5
4
3
2
1
0
1 2 3 4 5 6 7 8 9 10 11 12 13 14 15 16 17 18 19 20 21 22 23 24 25 26 27 28 29 30 31
1
2
3
4
Group and Phase

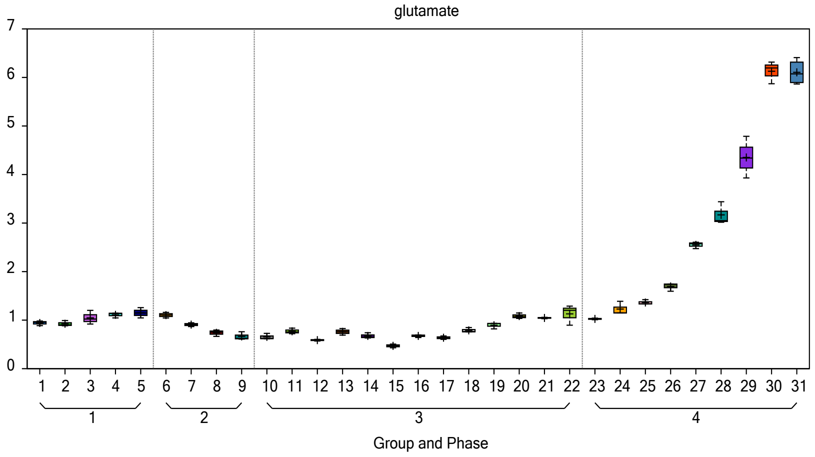
glutamate
7
6
5
4
3
2
1
0
1 2 3 4 5 6 7 8 9 10 11 12 13 14 15 16 17 18 19 20 21 22 23 24 25 26 27 28 29 30 31
1
2
3
4
Group and Phase

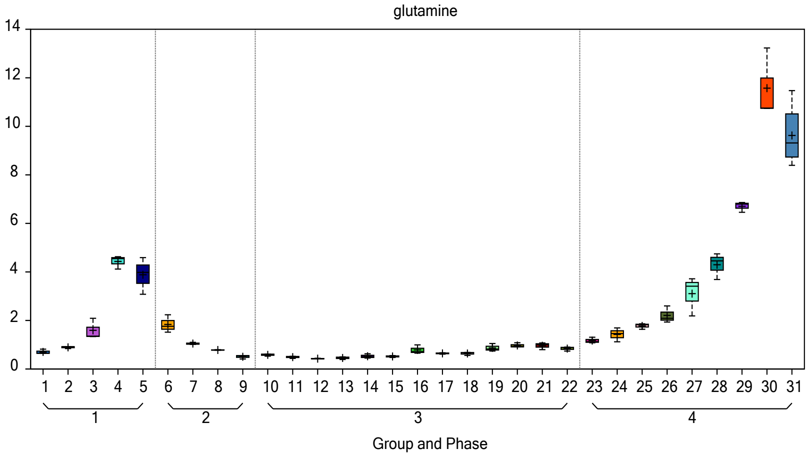
glutamine
0
2
4
6
8
10
12
14
1 2 3 4 5 6 7 8 9 10 11 12 13 14 15 16 17 18 19 20 21 22 23 24 25 26 27 28 29 30 31
1
2
3
4
Group and Phase

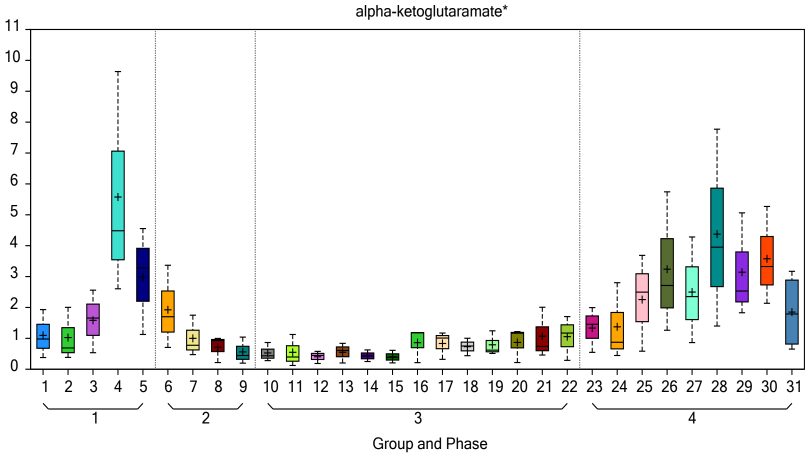
alpha-ketoglutaramate*
0
1
2
3
4
5
6
7
8
9
10
11
1 2 3 4 5 6 7 8 9 10 11 12 13 14 15 16 17 18 19 20 21 22 23 24 25 26 27 28 29 30 31
1
2
3
4
Group and Phase

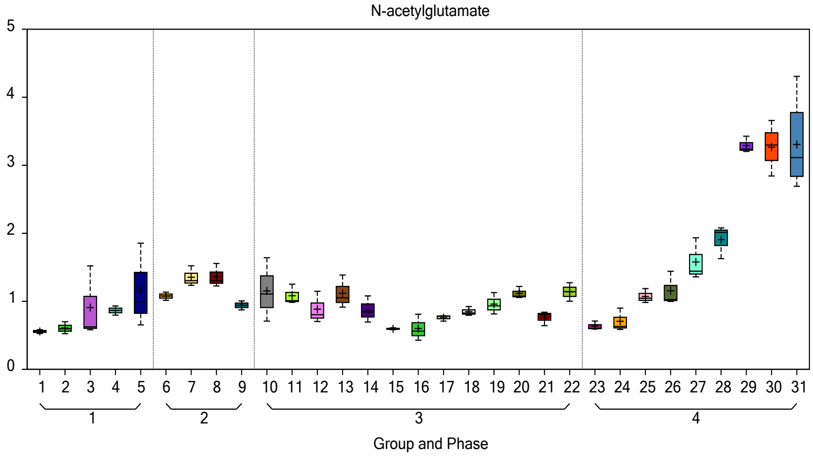
N-acetylglutamate
0
1
2
3
4
5
1 2 3 4 5 6 7 8 9 10 11 12 13 14 15 16 17 18 19 20 21 22 23 24 25 26 27 28 29 30 31
1
2
3
4
Group and Phase

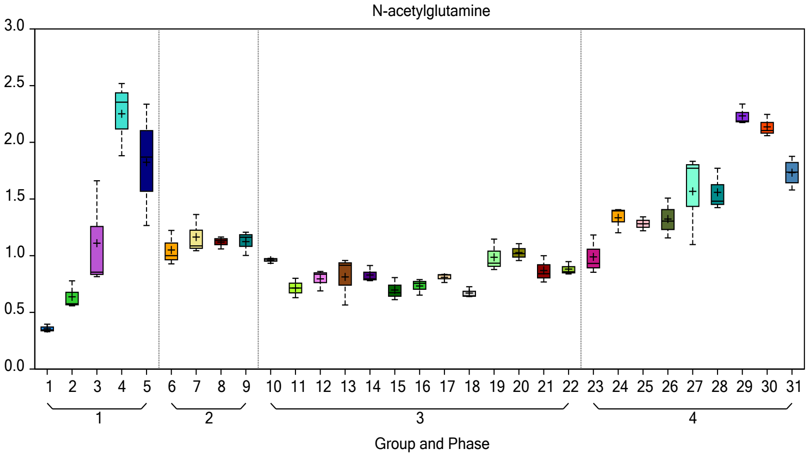
N-acetylglutamine
0.0
0.5
1.0
1.5
2.0
2.5
3.0
1 2 3 4 5 6 7 8 9 10 11 12 13 14 15 16 17 18 19 20 21 22 23 24 25 26 27 28 29 30 31
1
2
3
4
Group and Phase

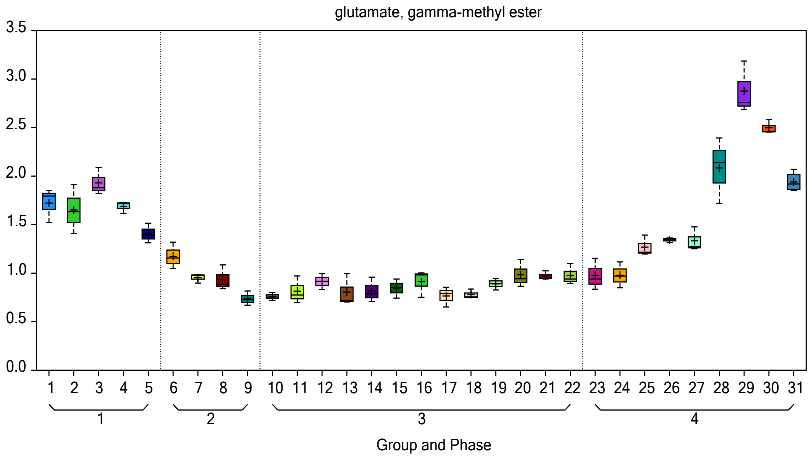
glutamate, gamma-methyl ester
3.5
3.0
2.5
2.0
1.5
1.0
0.5
0.0
1 2 3 4 5 6 7 8 9 10 11 12 13 14 15 16 17 18 19 20 21 22 23 24 25 26 27 28 29 30 31
1
2
3
4
Group and Phase

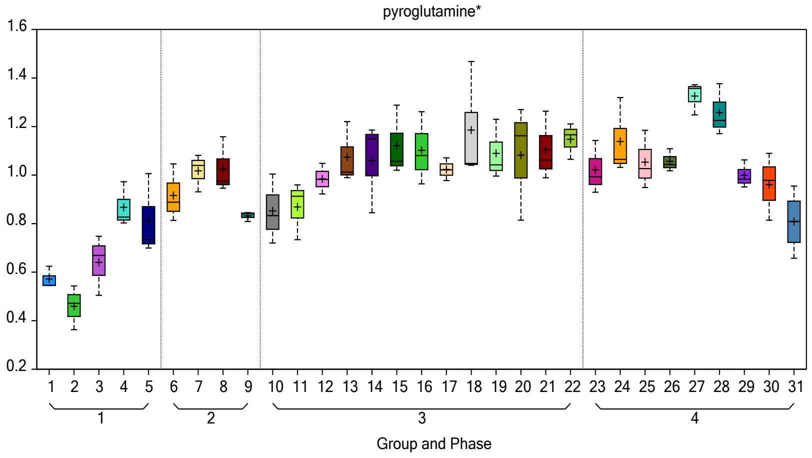
pyroglutamine*
1.6
1.4
1.2
1.0
0.8
0.6
0.4
0.2
1 2 3 4 5 6 7 8 9 10 11 12 13 14 15 16 17 18 19 20 21 22 23 24 25 26 27 28 29 30 31
1
2
3
4
Group and Phase

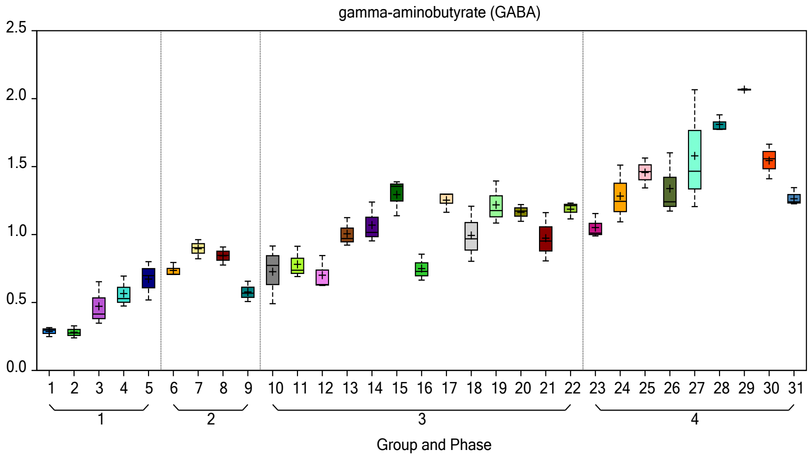
gamma-aminobutyrate (GABA)
2.5
2.0
1.5
1.0
0.5
0.0
1 2 3 4 5 6 7 8 9 10 11 12 13 14 15 16 17 18 19 20 21 22 23 24 25 26 27 28 29 30 31
1
2
3
4
Group and Phase

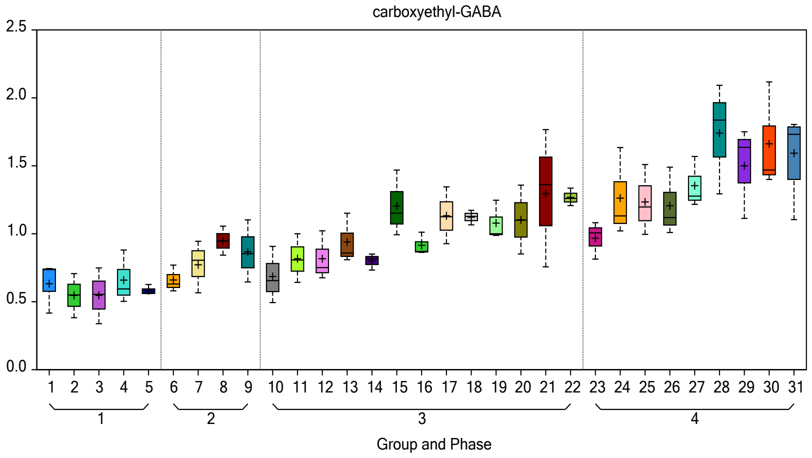
carboxyethyl-GABA
2.5
2.0
1.5
1.0
0.5
0.0
1 2 3 4 5 6 7 8 9 10 11 12 13 14 15 16 17 18 19 20 21 22 23 24 25 26 27 28 29 30 31
1
2
3
4
Group and Phase

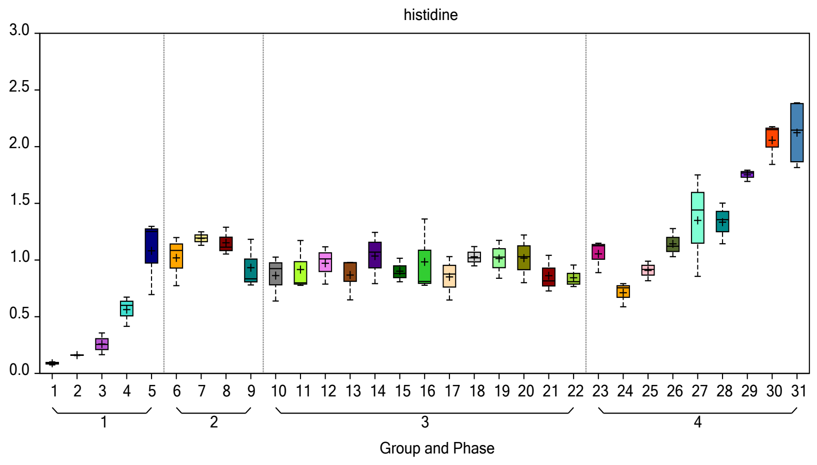
histidine
Group and Phase

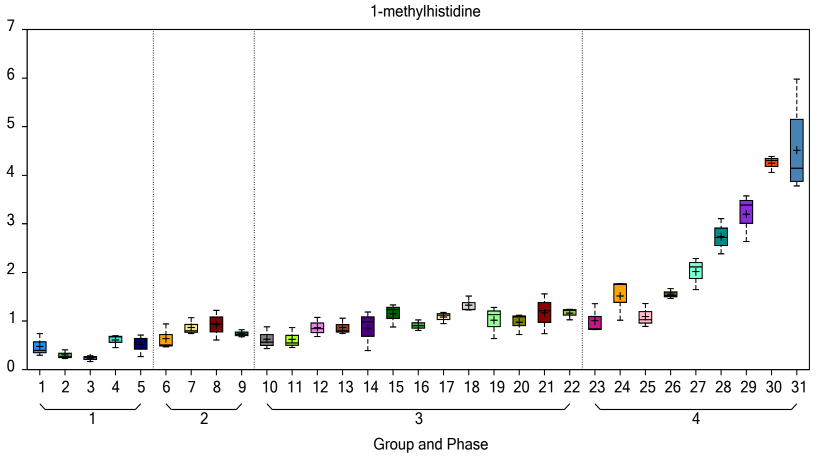
1-methylhistidine
Group and Phase

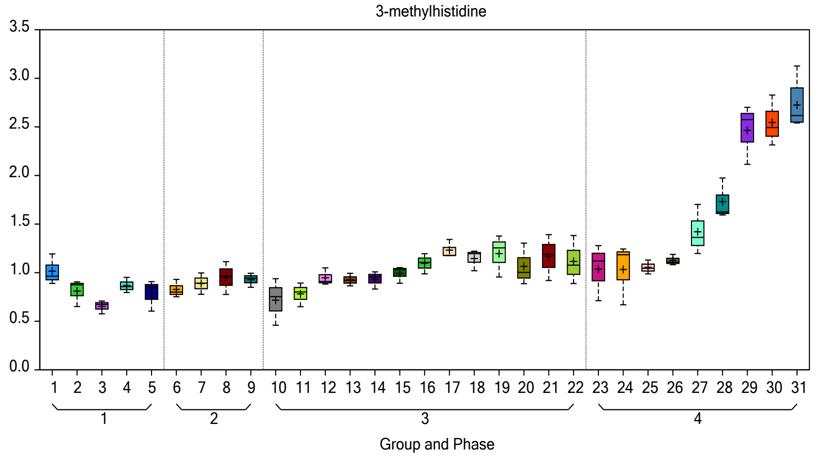
3-methylhistidine
Group and Phase

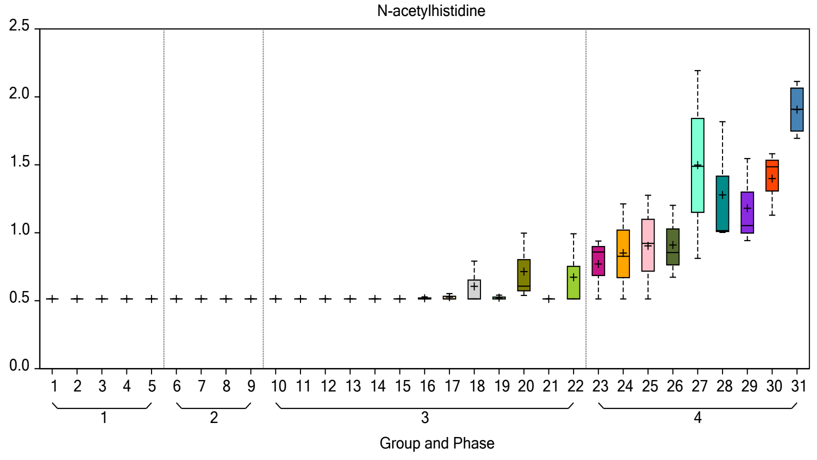
N-acetylhistidine
Group and Phase

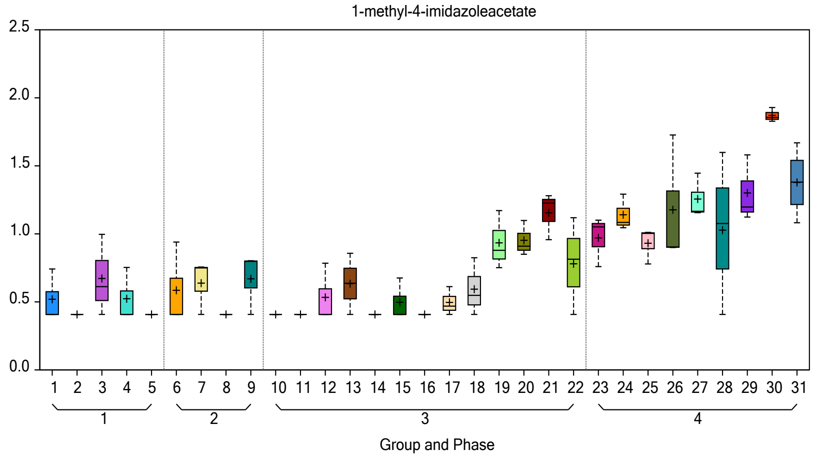
1-methyl-4-imidazoleacetate
2.5
2.0
1.5
1.0
0.5
0.0
1 2 3 4 5 6 7 8 9 10 11 12 13 14 15 16 17 18 19 20 21 22 23 24 25 26 27 28 29 30 31
1
2
3
4
Group and Phase

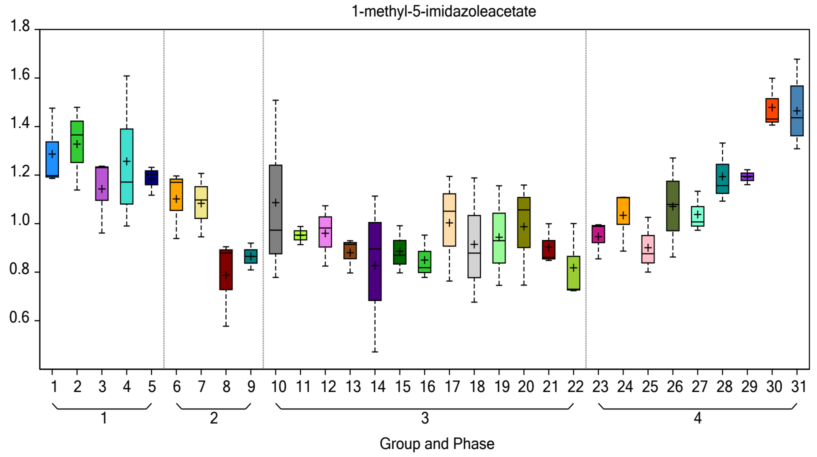
1-methyl-5-imidazoleacetate
1.8
1.6
1.4
1.2
1.0
0.8
0.6
1 2 3 4 5 6 7 8 9 10 11 12 13 14 15 16 17 18 19 20 21 22 23 24 25 26 27 28 29 30 31
1
2
3
4
Group and Phase

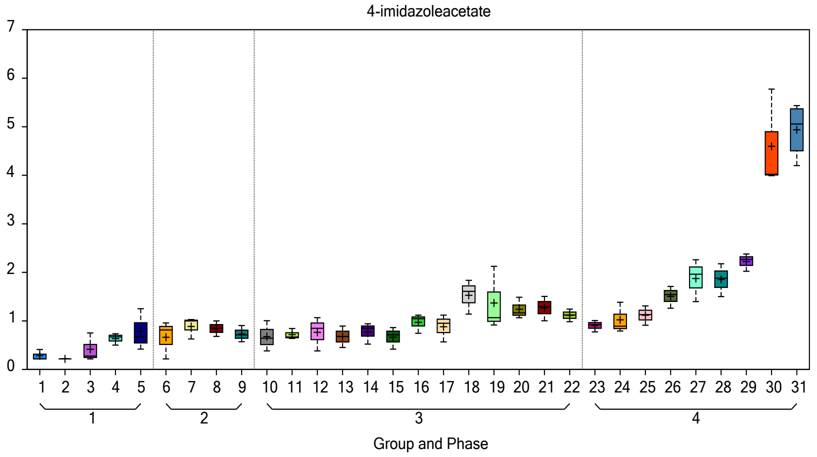
4-imidazoleacetate
7
6
5
4
3
2
1
0
1 2 3 4 5 6 7 8 9 10 11 12 13 14 15 16 17 18 19 20 21 22 23 24 25 26 27 28 29 30 31
1
2
3
4
Group and Phase

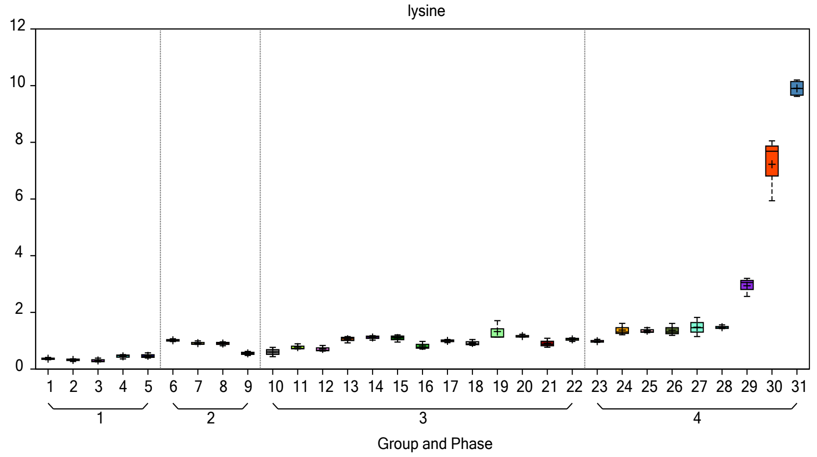
lysine
12
10
8
6
4
2
0
1 2 3 4 5 6 7 8 9 10 11 12 13 14 15 16 17 18 19 20 21 22 23 24 25 26 27 28 29 30 31
1
2
3
4
Group and Phase

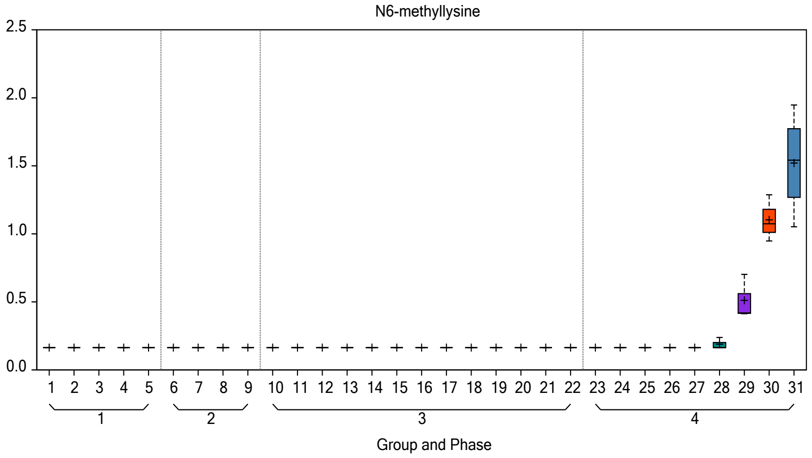
N6-methyllysine
0.0
0.5
1.0
1.5
2.0
2.5
1 2 3 4 5 6 7 8 9 10 11 12 13 14 15 16 17 18 19 20 21 22 23 24 25 26 27 28 29 30 31
1
2
3
4
Group and Phase

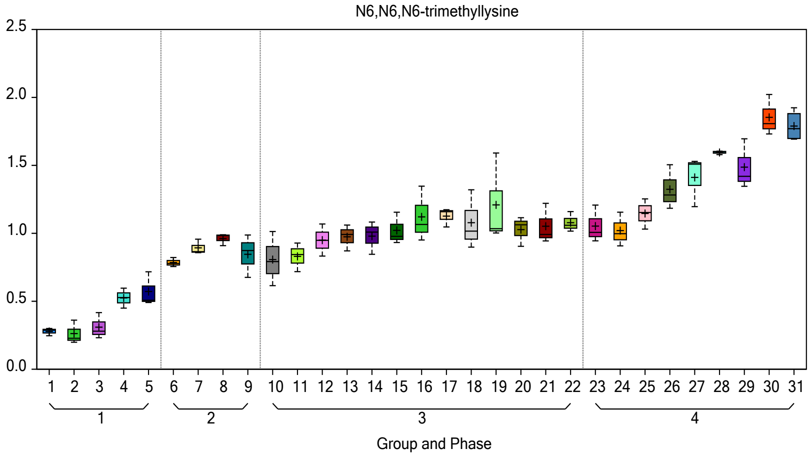
N6,N6,N6-trimethyllysine
0.0
0.5
1.0
1.5
2.0
2.5
1 2 3 4 5 6 7 8 9 10 11 12 13 14 15 16 17 18 19 20 21 22 23 24 25 26 27 28 29 30 31
1
2
3
4
Group and Phase

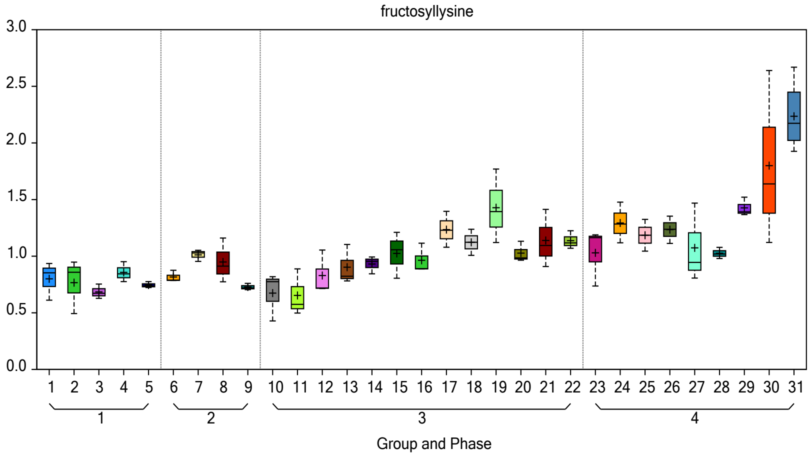
fructosyllysine
0.0
0.5
1.0
1.5
2.0
2.5
3.0
1 2 3 4 5 6 7 8 9 10 11 12 13 14 15 16 17 18 19 20 21 22 23 24 25 26 27 28 29 30 31
1
2
3
4
Group and Phase

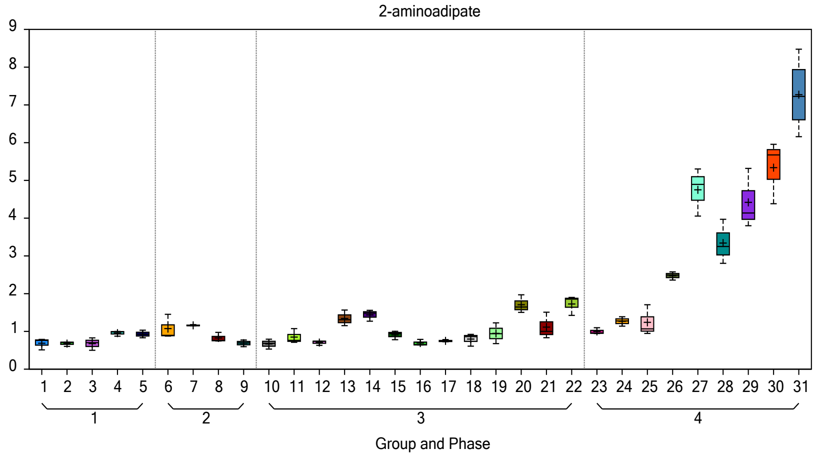
2-aminoadipate
0
1
2
3
4
5
6
7
8
9
1 2 3 4 5 6 7 8 9 10 11 12 13 14 15 16 17 18 19 20 21 22 23 24 25 26 27 28 29 30 31
1
2
3
4
Group and Phase

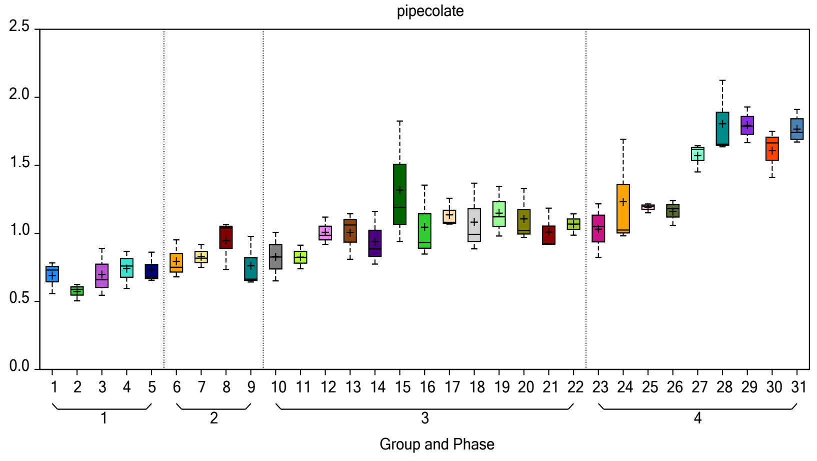
pipecolate
Group and Phase

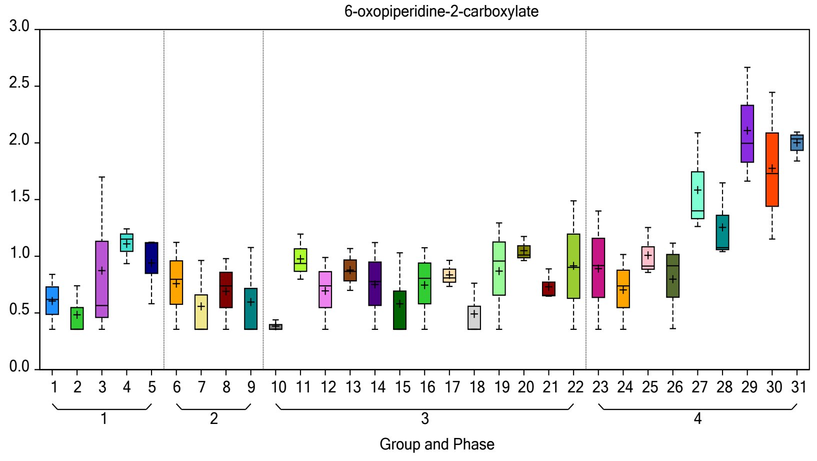
6-oxopiperidine-2-carboxylate
Group and Phase

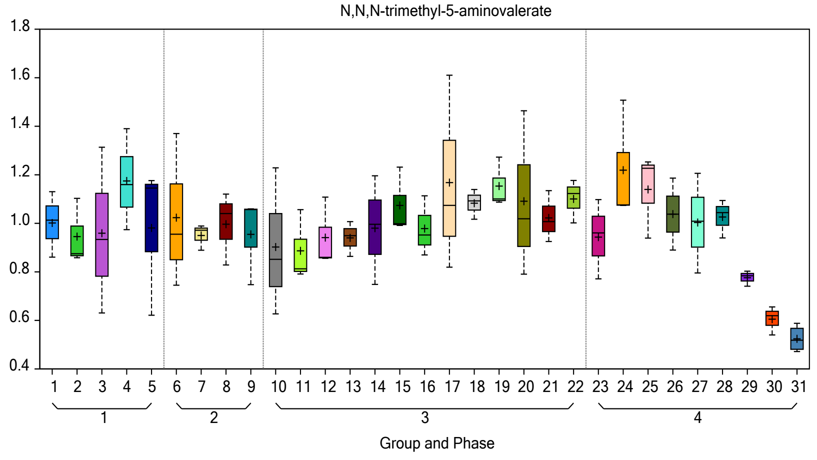
N,N,N-trimethyl-5-aminovalerate
Group and Phase

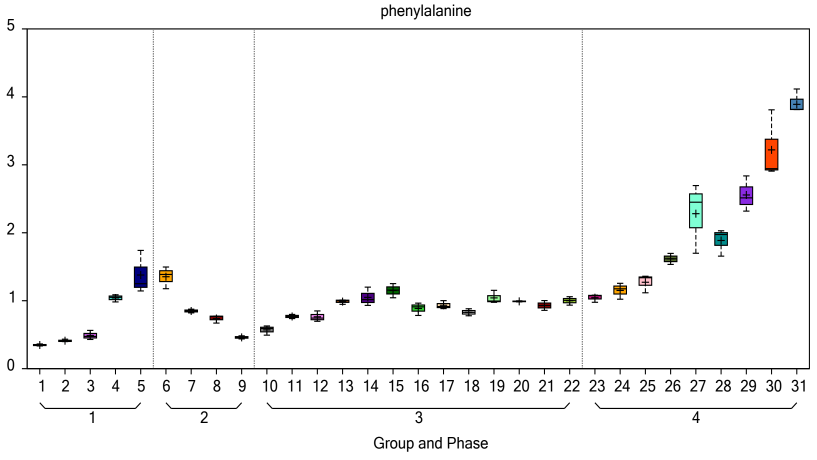
phenylalanine
Group and Phase

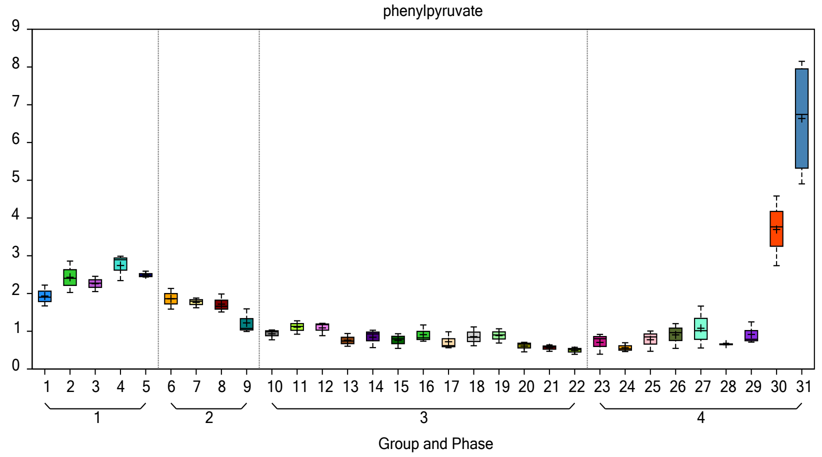
phenylpyruvate
0 1 2 3 4 5 6 7 8 9
1 2 3 4 5 6 7 8 9 10 11 12 13 14 15 16 17 18 19 20 21 22 23 24 25 26 27 28 29 30 31
1 2 3 4
Group and Phase

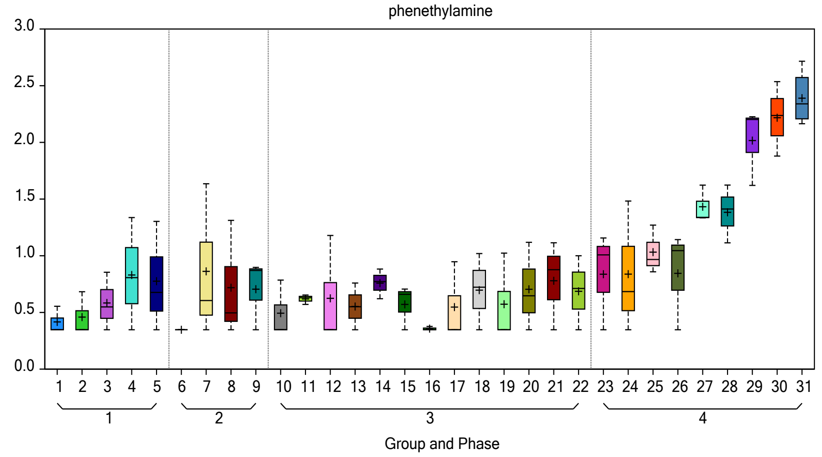
phenethylamine
0.0 0.5 1.0 1.5 2.0 2.5 3.0
1 2 3 4 5 6 7 8 9 10 11 12 13 14 15 16 17 18 19 20 21 22 23 24 25 26 27 28 29 30 31
1 2 3 4
Group and Phase

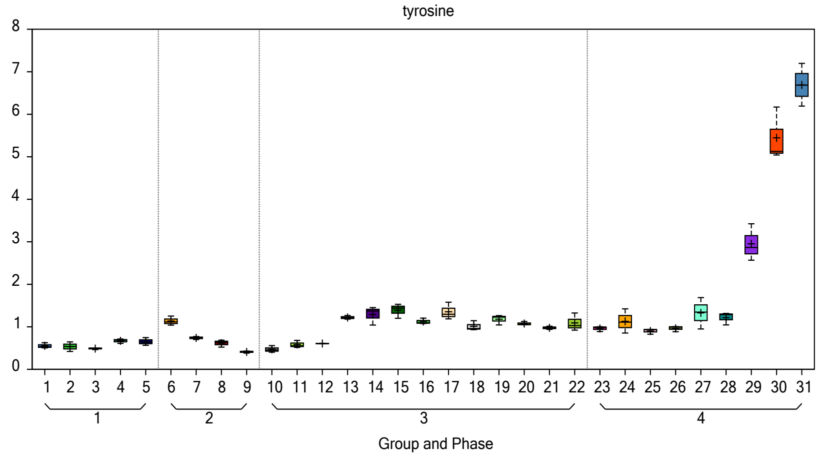
tyrosine
0 1 2 3 4 5 6 7 8
1 2 3 4 5 6 7 8 9 10 11 12 13 14 15 16 17 18 19 20 21 22 23 24 25 26 27 28 29 30 31
1 2 3 4
Group and Phase

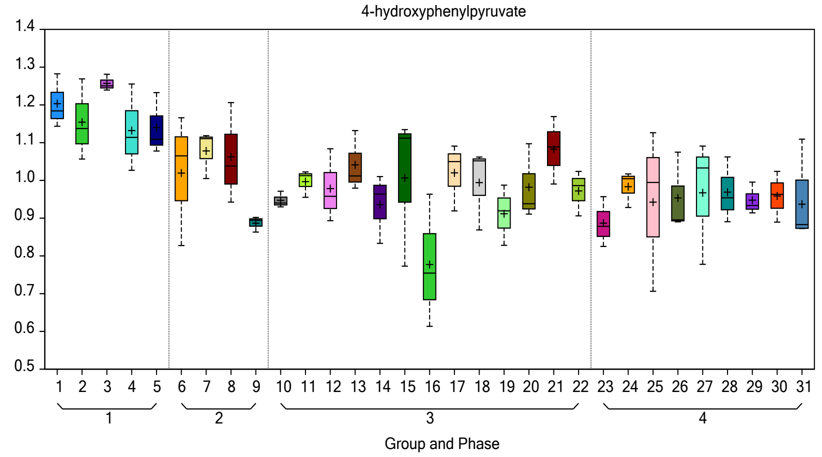
4-hydroxyphenylpyruvate
0.5 0.6 0.7 0.8 0.9 1.0 1.1 1.2 1.3 1.4
1 2 3 4 5 6 7 8 9 10 11 12 13 14 15 16 17 18 19 20 21 22 23 24 25 26 27 28 29 30 31
1 2 3 4
Group and Phase

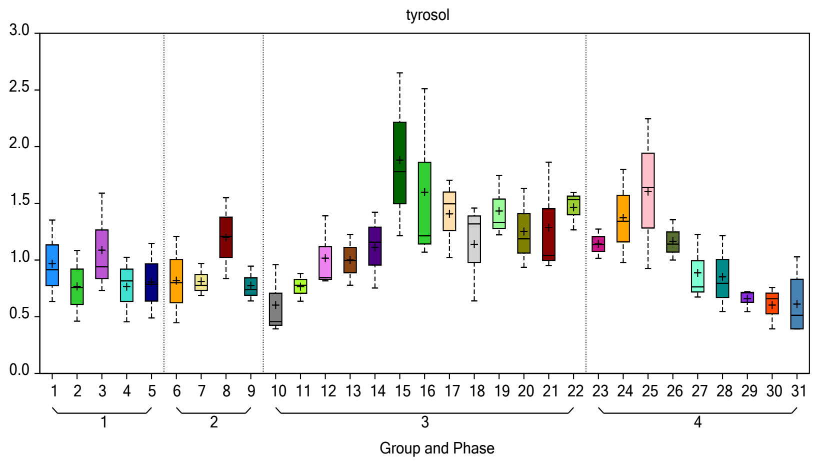
tyrosol
Group and Phase

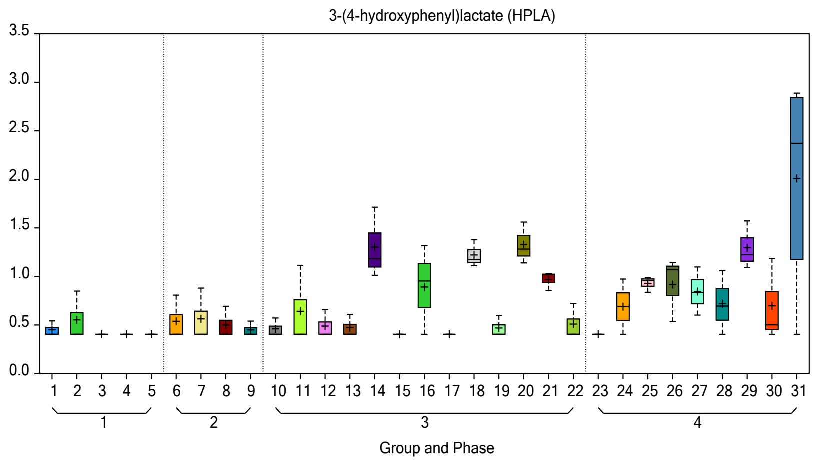
3-(4-hydroxyphenyl)lactate (HPLA)
Group and Phase

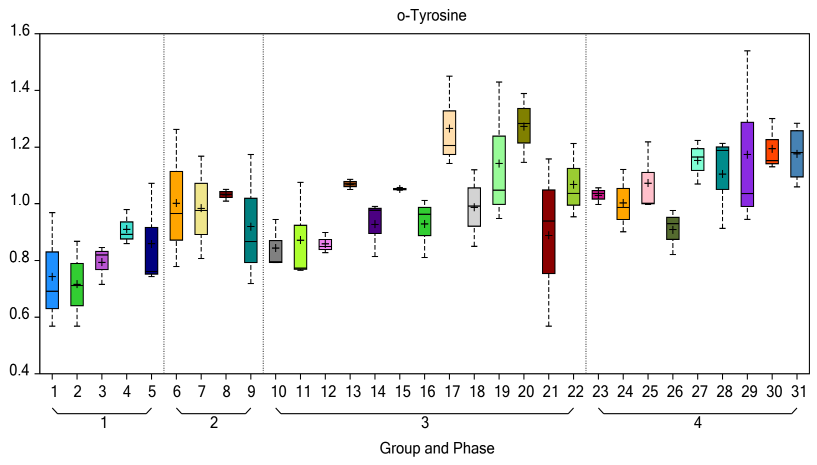
o-Tyrosine
Group and Phase

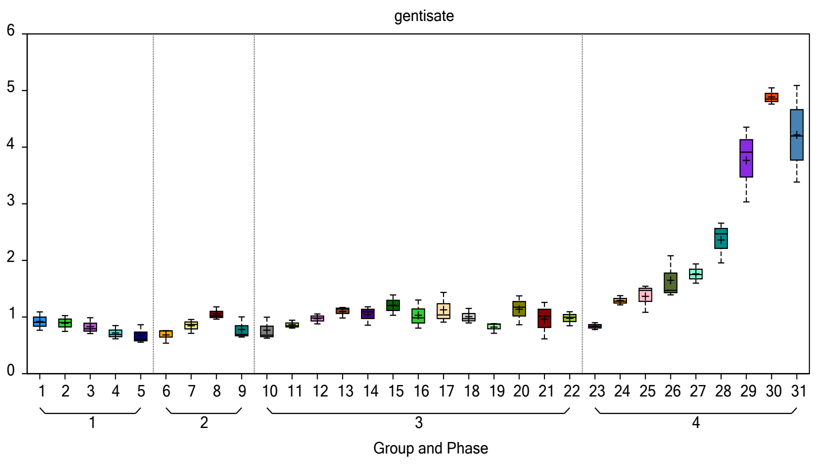
gentisate
Group and Phase

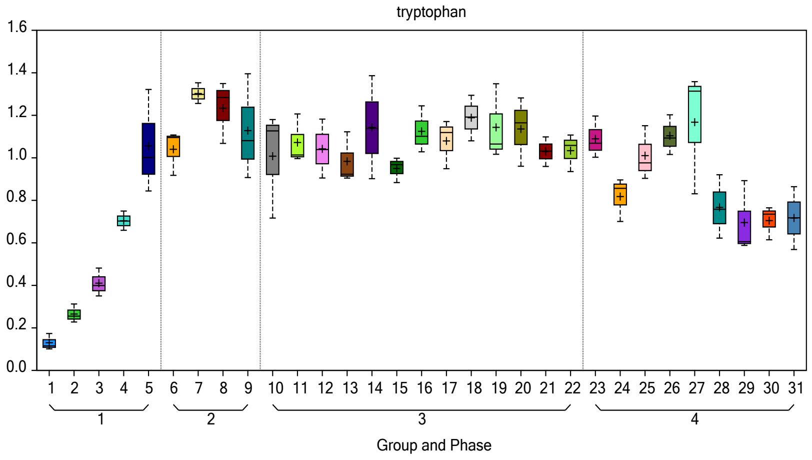
tryptophan
1.6
1.4
1.2
1.0
0.8
0.6
0.4
0.2
0.0
1 2 3 4 5 6 7 8 9 10 11 12 13 14 15 16 17 18 19 20 21 22 23 24 25 26 27 28 29 30 31
1
2
3
4
Group and Phase

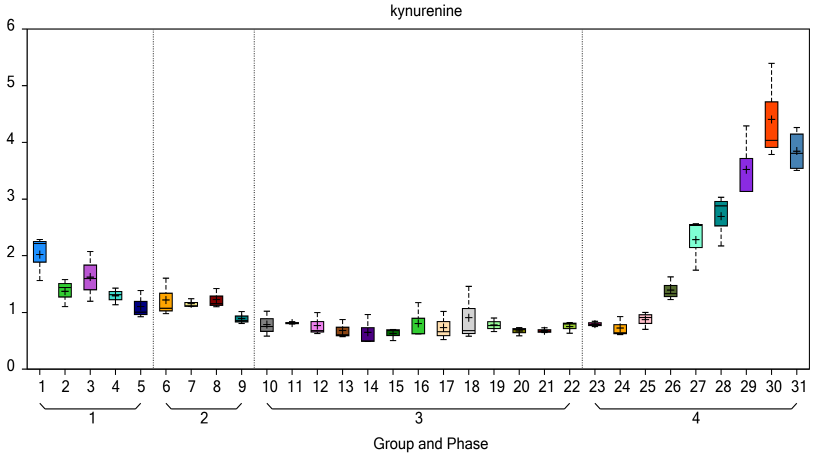
kynurenine
6
5
4
3
2
1
0
1 2 3 4 5 6 7 8 9 10 11 12 13 14 15 16 17 18 19 20 21 22 23 24 25 26 27 28 29 30 31
1
2
3
4
Group and Phase

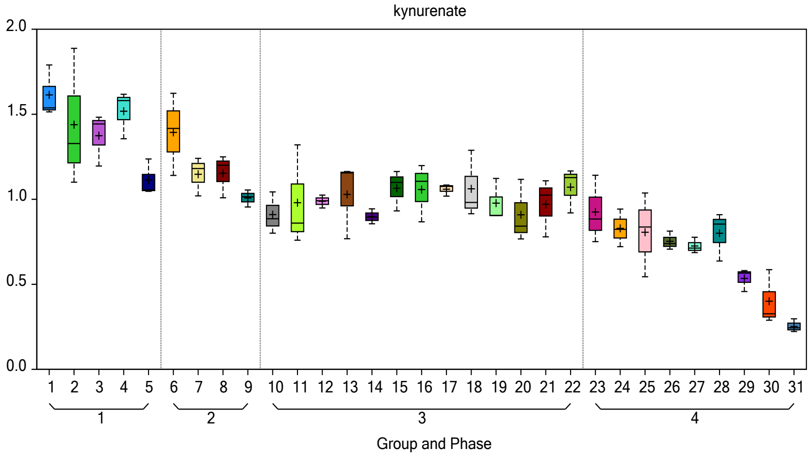
kynurenate
2.0
1.5
1.0
0.5
0.0
1 2 3 4 5 6 7 8 9 10 11 12 13 14 15 16 17 18 19 20 21 22 23 24 25 26 27 28 29 30 31
1
2
3
4
Group and Phase

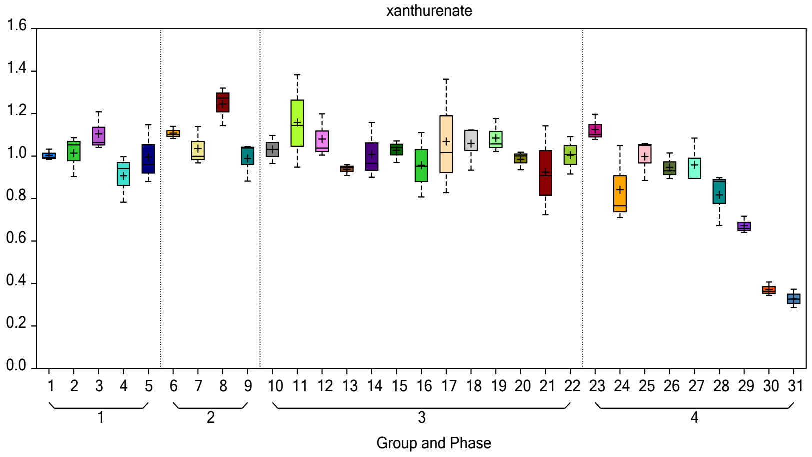
xanthurenate
1.6
1.4
1.2
1.0
0.8
0.6
0.4
0.2
0.0
1 2 3 4 5 6 7 8 9 10 11 12 13 14 15 16 17 18 19 20 21 22 23 24 25 26 27 28 29 30 31
1
2
3
4
Group and Phase

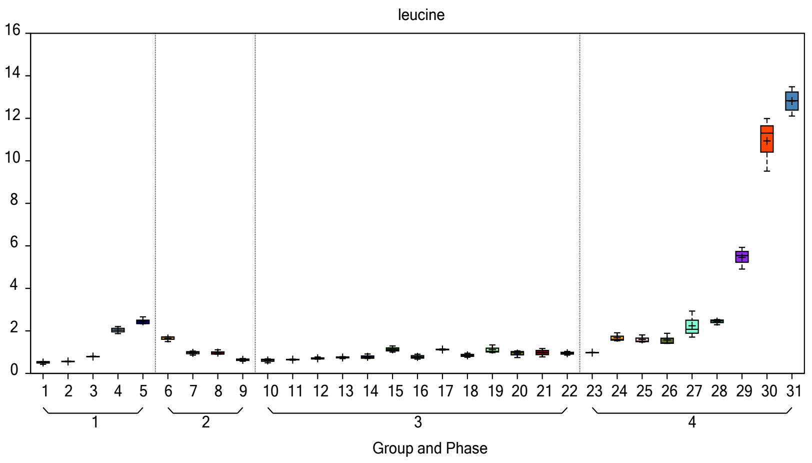
leucine
16
14
12
10
8
6
4
2
0
1 2 3 4 5 6 7 8 9 10 11 12 13 14 15 16 17 18 19 20 21 22 23 24 25 26 27 28 29 30 31
1
2
3
4
Group and Phase

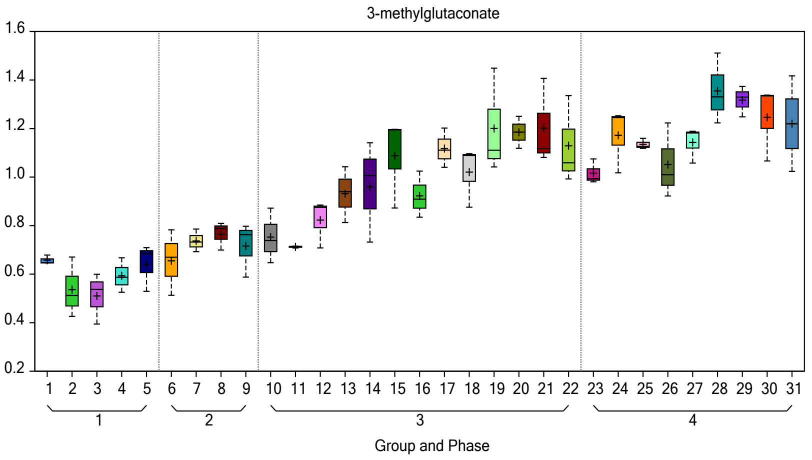
3-methylglutaconate
1.6
1.4
1.2
1.0
0.8
0.6
0.4
0.2
1 2 3 4 5 6 7 8 9 10 11 12 13 14 15 16 17 18 19 20 21 22 23 24 25 26 27 28 29 30 31
1
2
3
4
Group and Phase

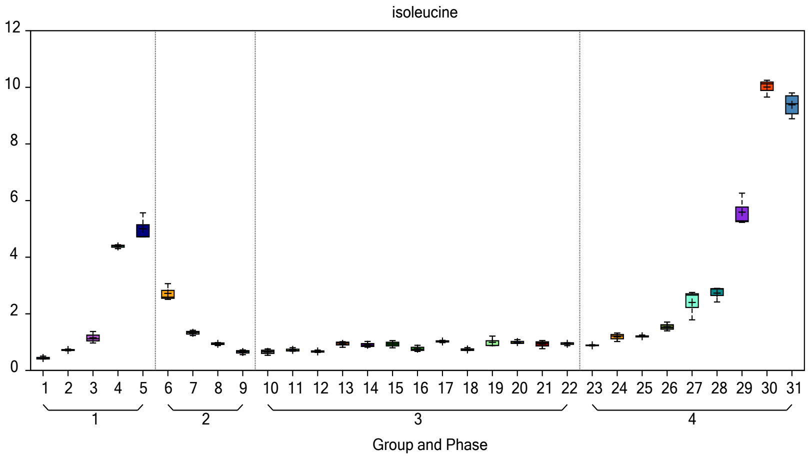
isoleucine
12
10
8
6
4
2
0
1 2 3 4 5 6 7 8 9 10 11 12 13 14 15 16 17 18 19 20 21 22 23 24 25 26 27 28 29 30 31
1
2
3
4
Group and Phase

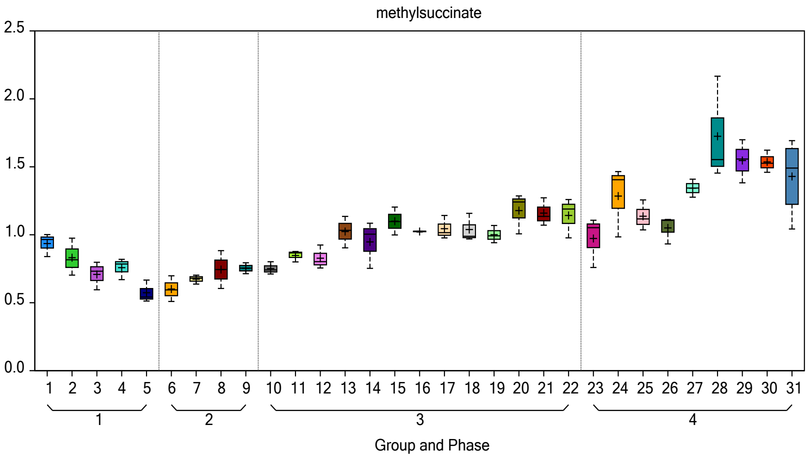
methylsuccinate
2.5
2.0
1.5
1.0
0.5
0.0
1 2 3 4 5 6 7 8 9 10 11 12 13 14 15 16 17 18 19 20 21 22 23 24 25 26 27 28 29 30 31
1
2
3
4
Group and Phase

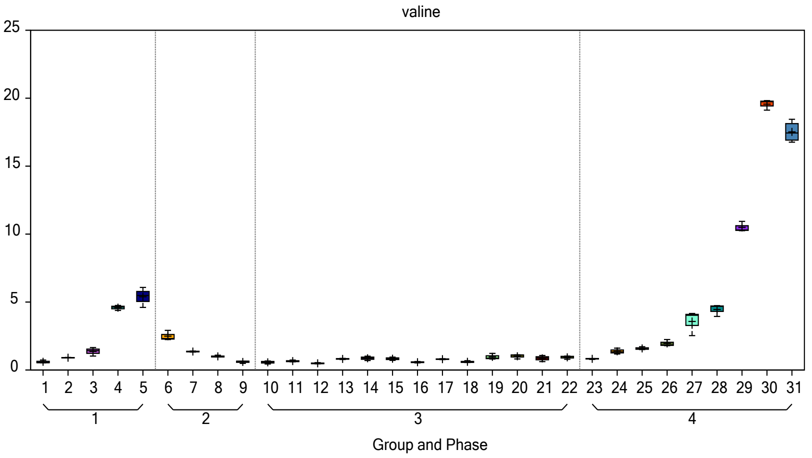
valine
0 5 10 15 20 25
1 2 3 4 5 6 7 8 9 10 11 12 13 14 15 16 17 18 19 20 21 22 23 24 25 26 27 28 29 30 31
1 2 3 4
Group and Phase

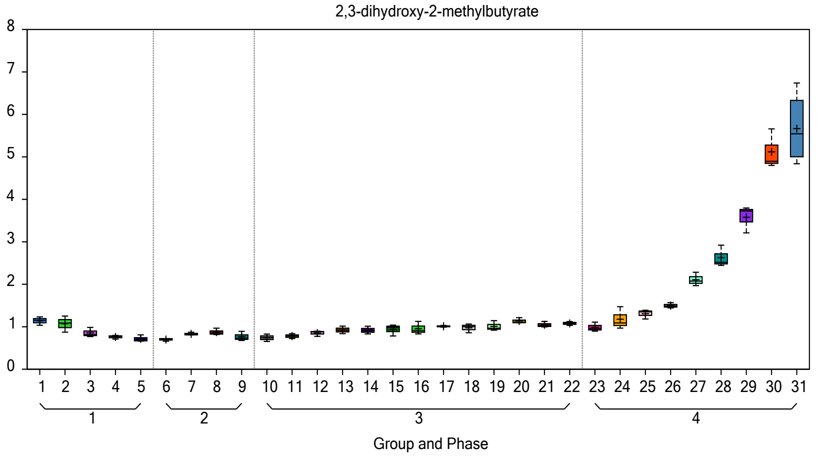
2,3-dihydroxy-2-methylbutyrate
0 1 2 3 4 5 6 7 8
1 2 3 4 5 6 7 8 9 10 11 12 13 14 15 16 17 18 19 20 21 22 23 24 25 26 27 28 29 30 31
1 2 3 4
Group and Phase

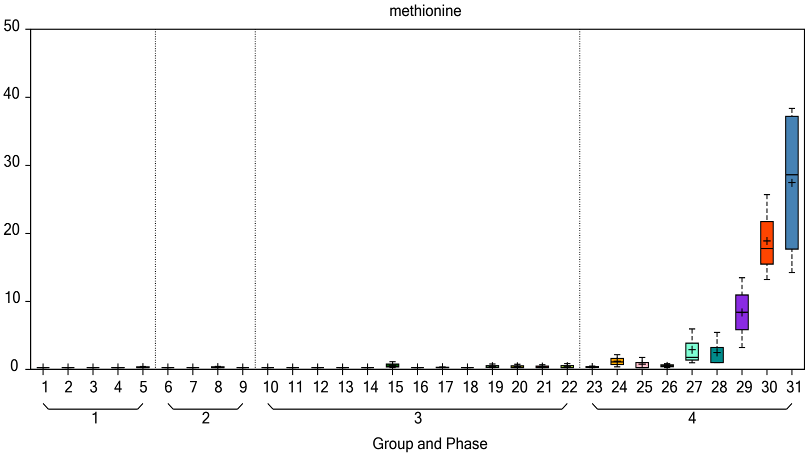
methionine
0 10 20 30 40 50
1 2 3 4 5 6 7 8 9 10 11 12 13 14 15 16 17 18 19 20 21 22 23 24 25 26 27 28 29 30 31
1 2 3 4
Group and Phase

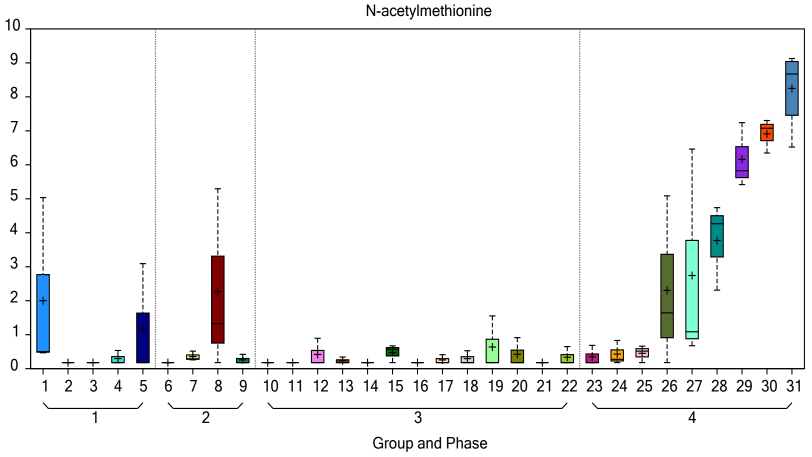
N-acetylmethionine
0 1 2 3 4 5 6 7 8 9 10
1 2 3 4 5 6 7 8 9 10 11 12 13 14 15 16 17 18 19 20 21 22 23 24 25 26 27 28 29 30 31
1 2 3 4
Group and Phase

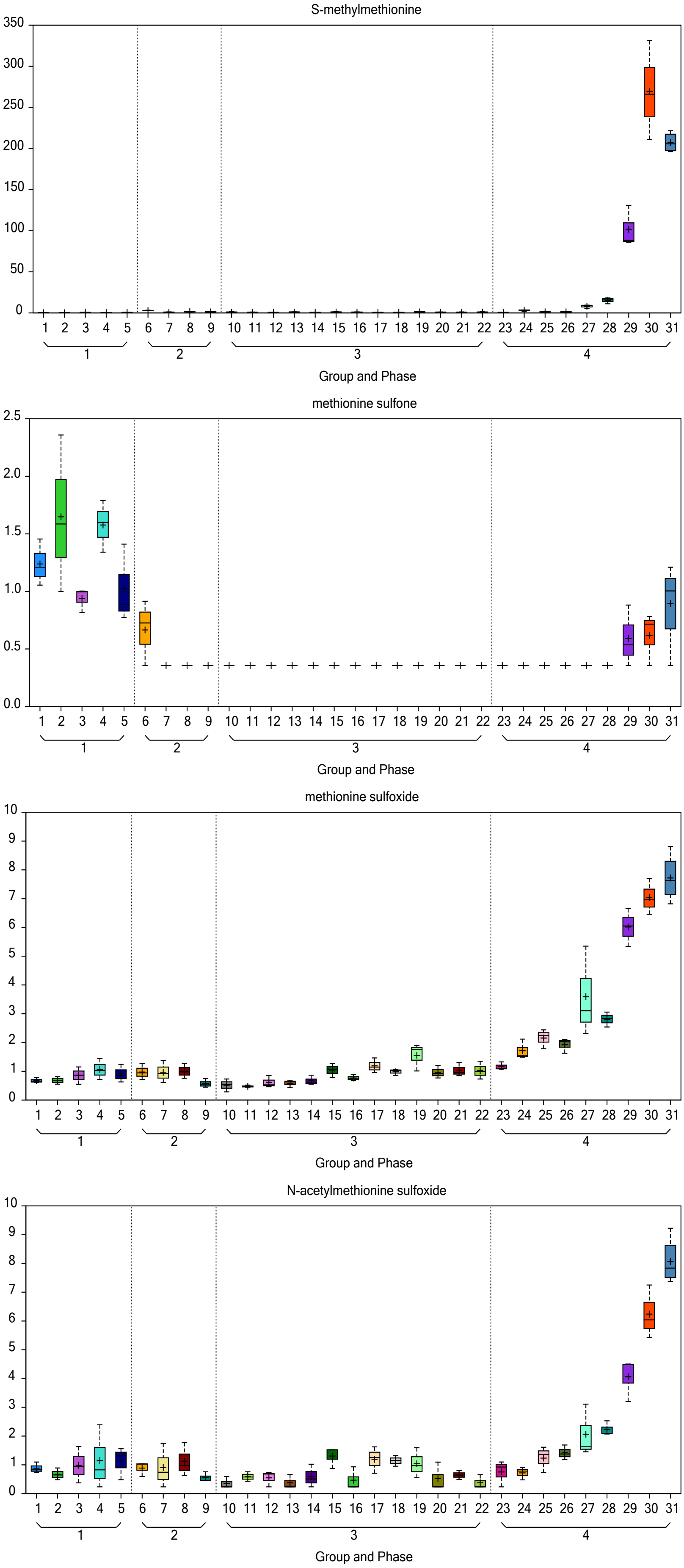
S-methylmethionine
methionine sulfone
methionine sulfoxide
N-acetylmethionine sulfoxide
Group and Phase

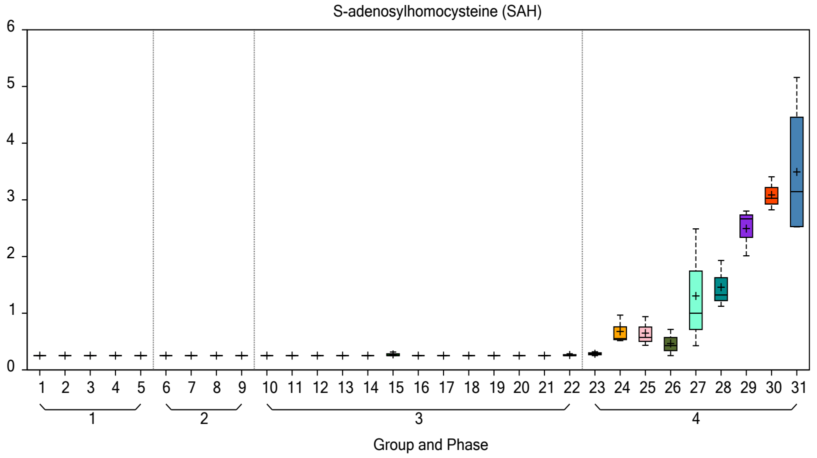
S-adenosylhomocysteine (SAH)
Group and Phase

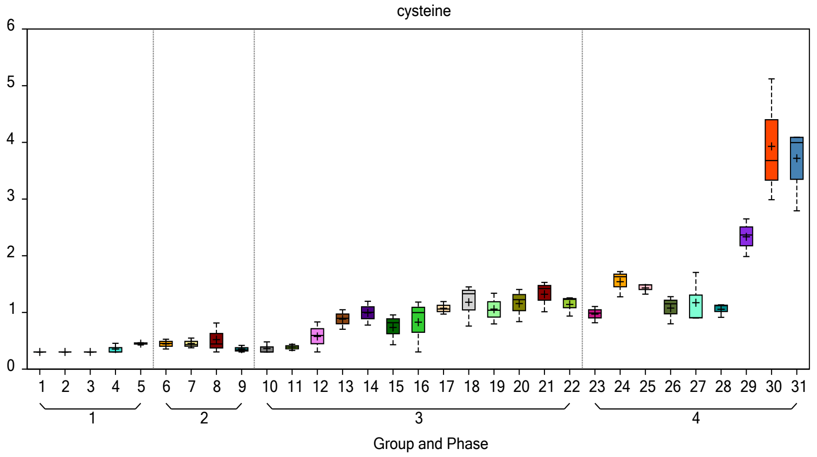
cysteine
Group and Phase

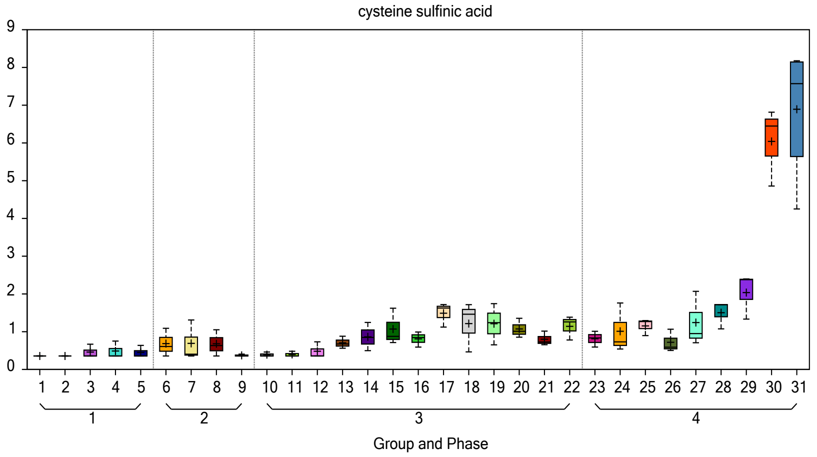
cysteine sulfinic acid
Group and Phase

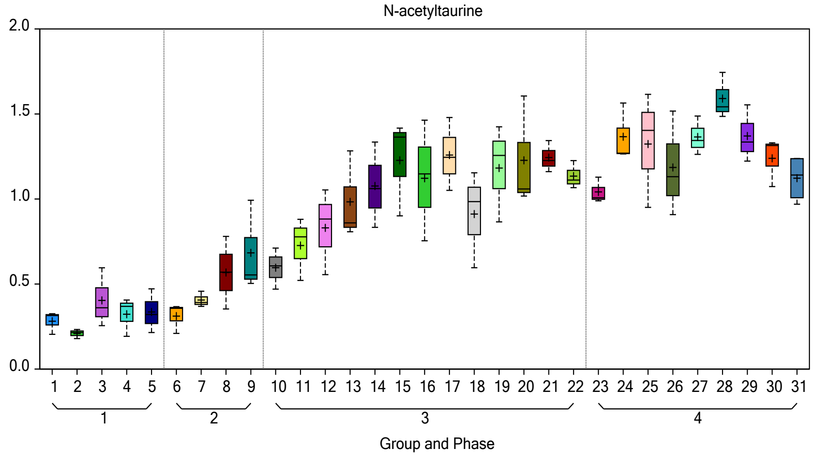
N-acetyltaurine
Group and Phase

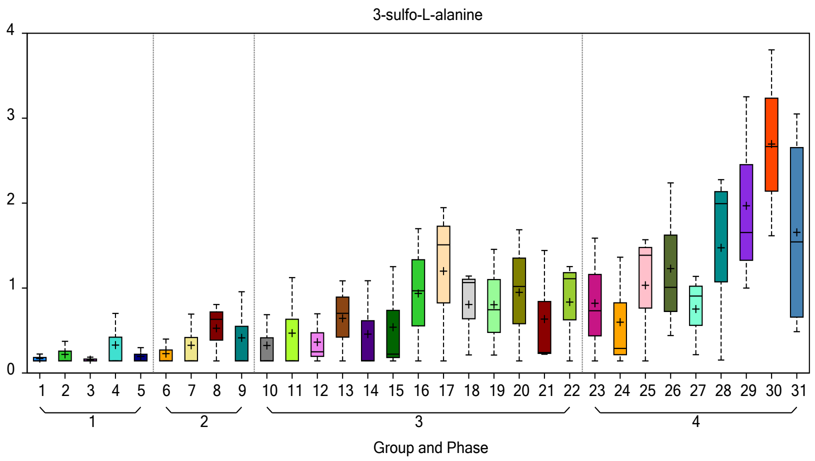
3-sulfo-L-alanine
0
1
2
3
4
1 2 3 4 5 6 7 8 9 10 11 12 13 14 15 16 17 18 19 20 21 22 23 24 25 26 27 28 29 30 31
1
2
3
4
Group and Phase

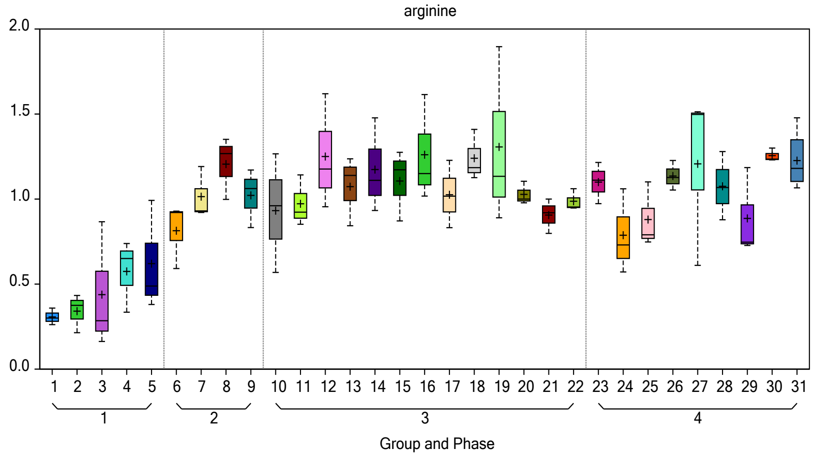
arginine
0.0
0.5
1.0
1.5
2.0
1 2 3 4 5 6 7 8 9 10 11 12 13 14 15 16 17 18 19 20 21 22 23 24 25 26 27 28 29 30 31
1
2
3
4
Group and Phase

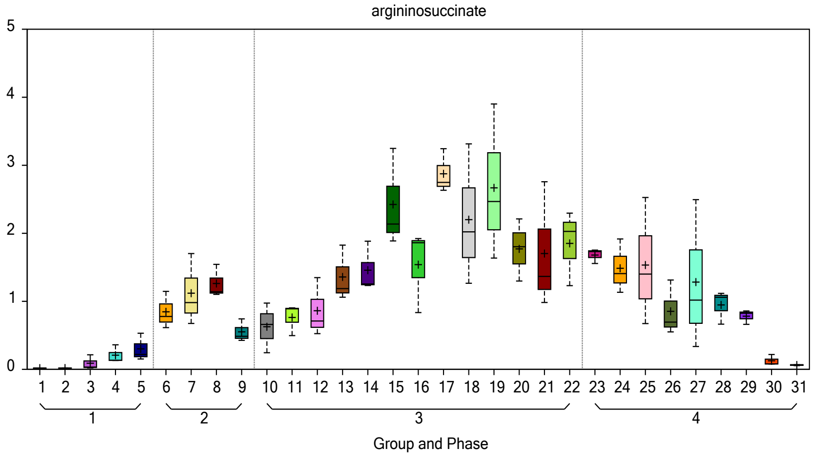
argininosuccinate
0
1
2
3
4
5
1 2 3 4 5 6 7 8 9 10 11 12 13 14 15 16 17 18 19 20 21 22 23 24 25 26 27 28 29 30 31
1
2
3
4
Group and Phase

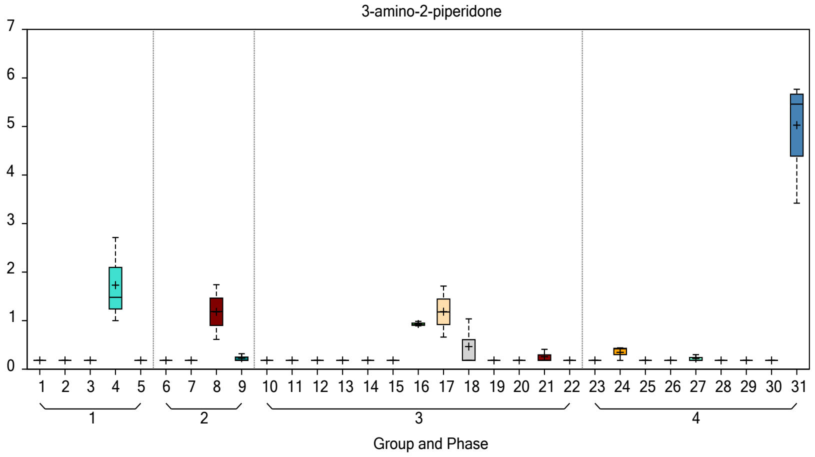
3-amino-2-piperidone
0
1
2
3
4
5
6
7
1 2 3 4 5 6 7 8 9 10 11 12 13 14 15 16 17 18 19 20 21 22 23 24 25 26 27 28 29 30 31
1
2
3
4
Group and Phase

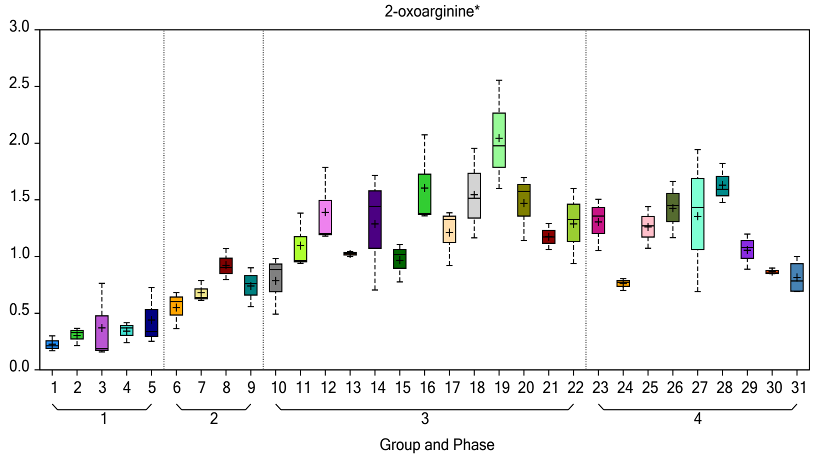
2-oxoarginine*
Group and Phase

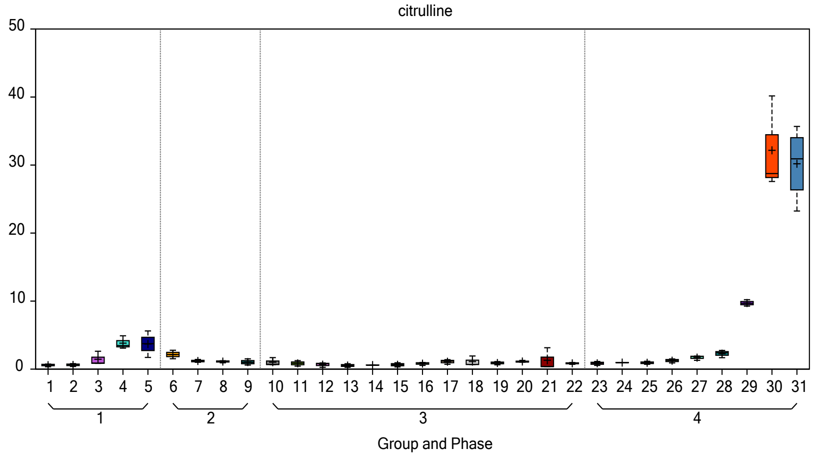
citrulline
Group and Phase

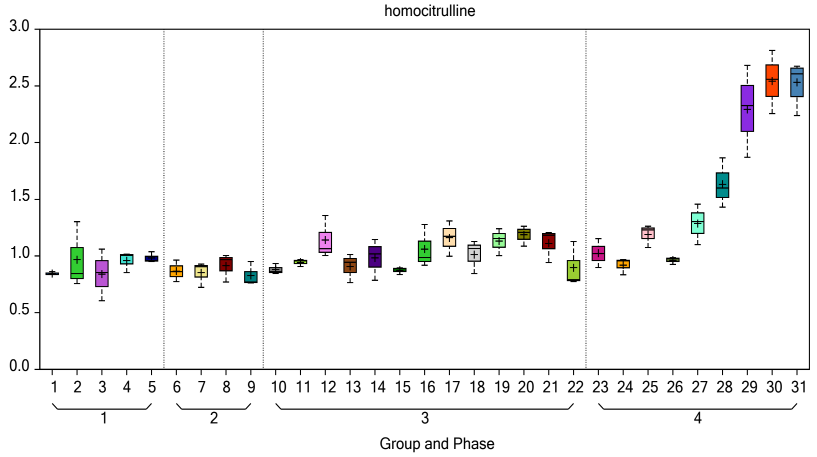
homocitrulline
Group and Phase

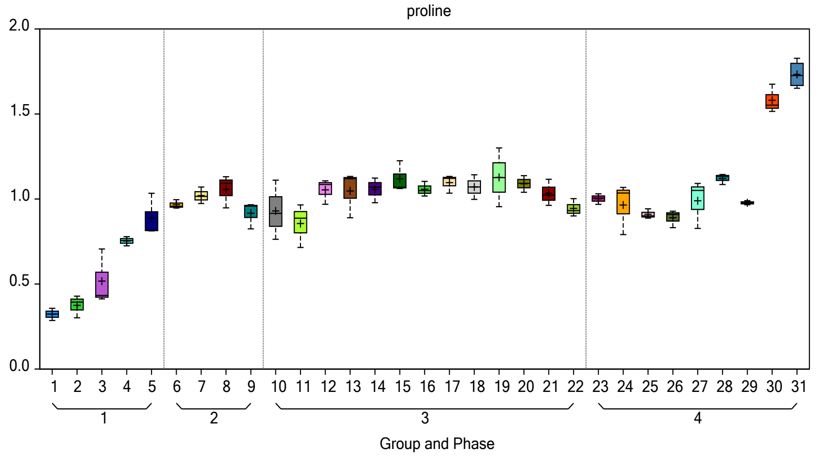
proline
Group and Phase

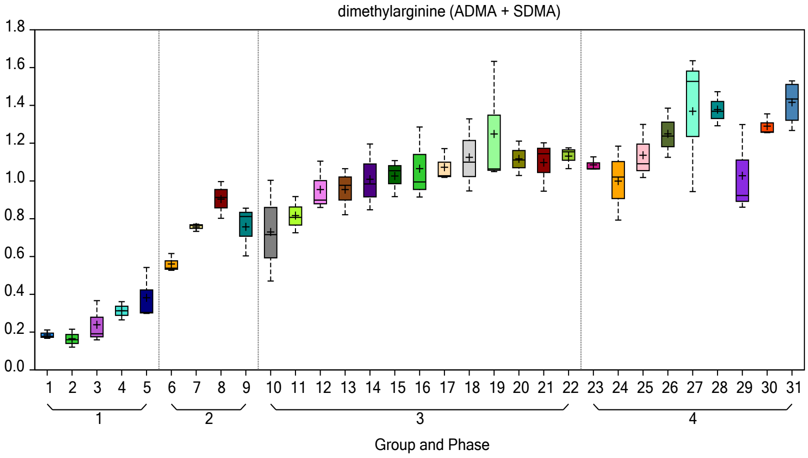
dimethylarginine (ADMA + SDMA)
Group and Phase

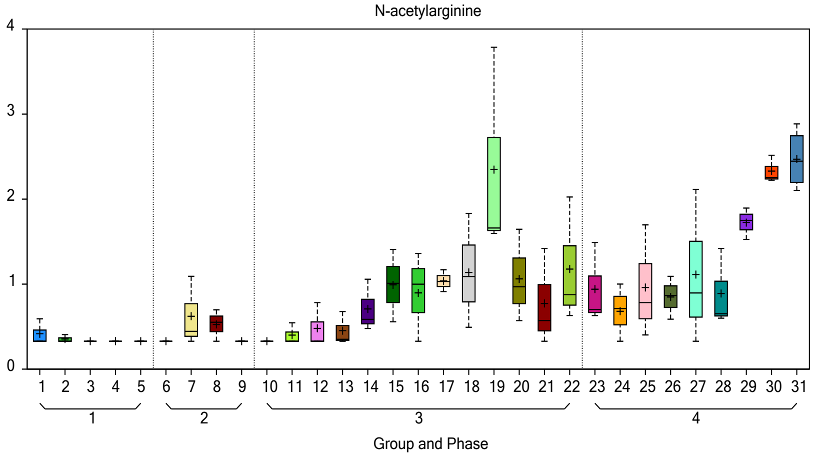
N-acetylarginine
Group and Phase

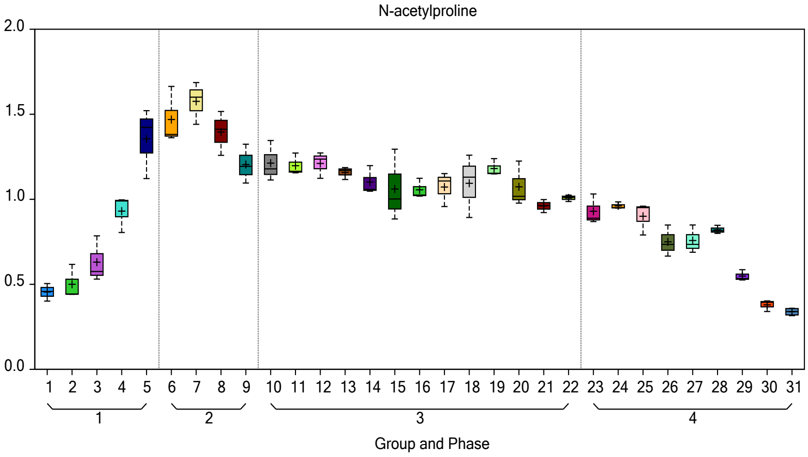
N-acetylproline
Group and Phase

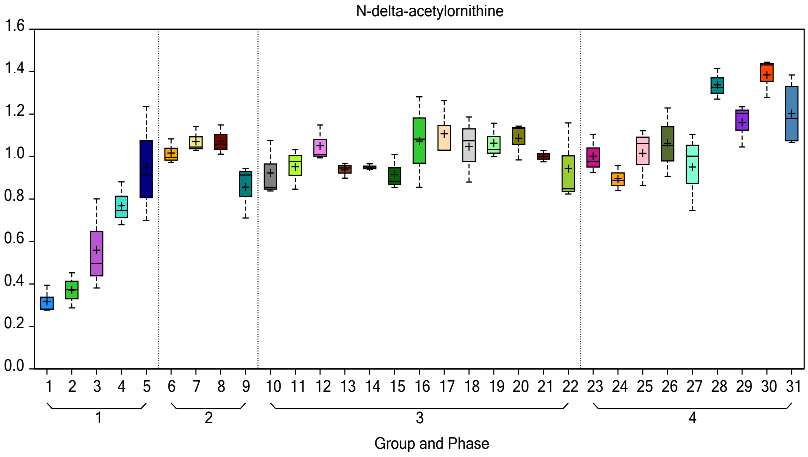
N-delta-acetylornithine
Group and Phase

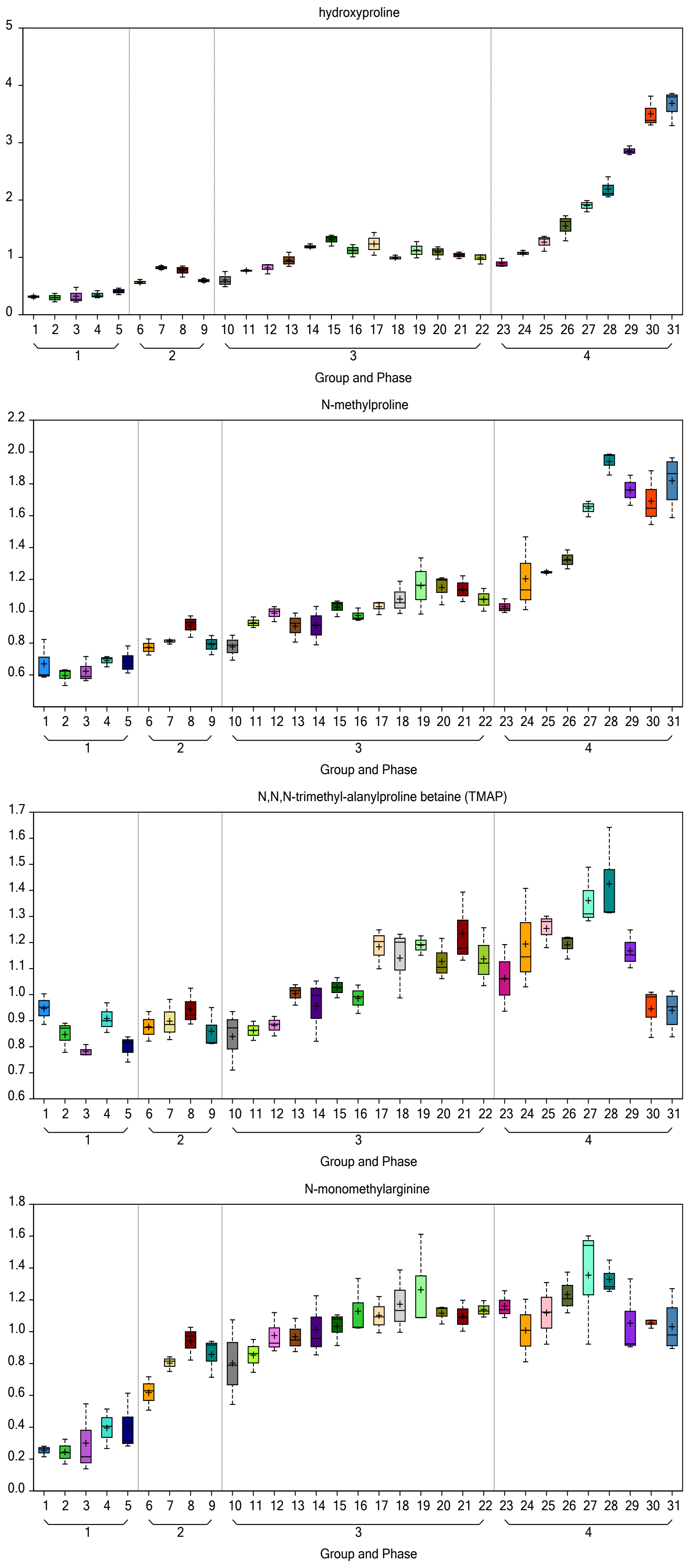
hydroxyproline
Group and Phase
N-methylproline
Group and Phase
N,N,N-trimethyl-alanylproline betaine (TMAP)
Group and Phase
N-monomethylarginine
Group and Phase

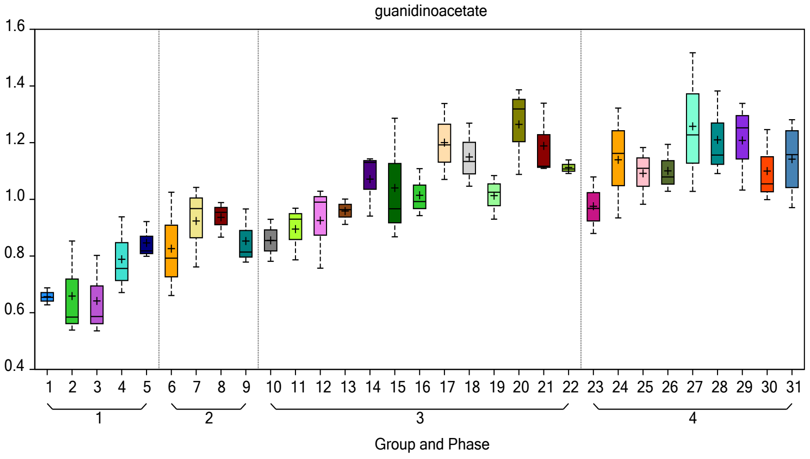
guanidinoacetate
1.6
1.4
1.2
1.0
0.8
0.6
0.4
1 2 3 4 5 6 7 8 9 10 11 12 13 14 15 16 17 18 19 20 21 22 23 24 25 26 27 28 29 30 31
1
2
3
4
Group and Phase

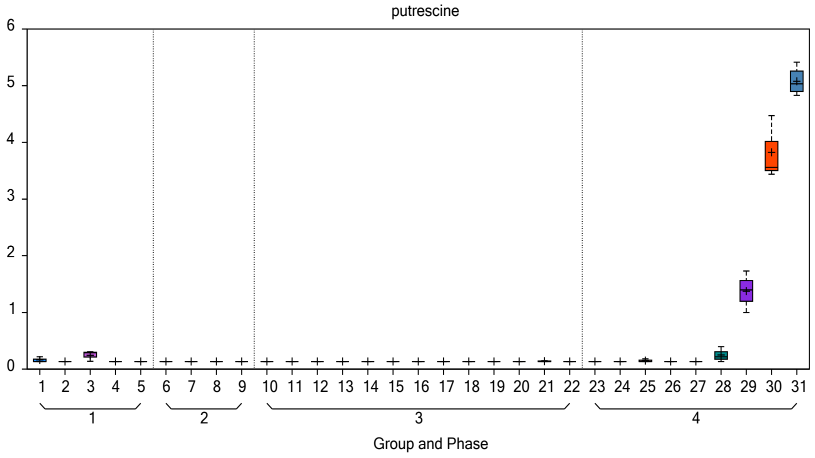
putrescine
6
5
4
3
2
1
0
1 2 3 4 5 6 7 8 9 10 11 12 13 14 15 16 17 18 19 20 21 22 23 24 25 26 27 28 29 30 31
1
2
3
4
Group and Phase

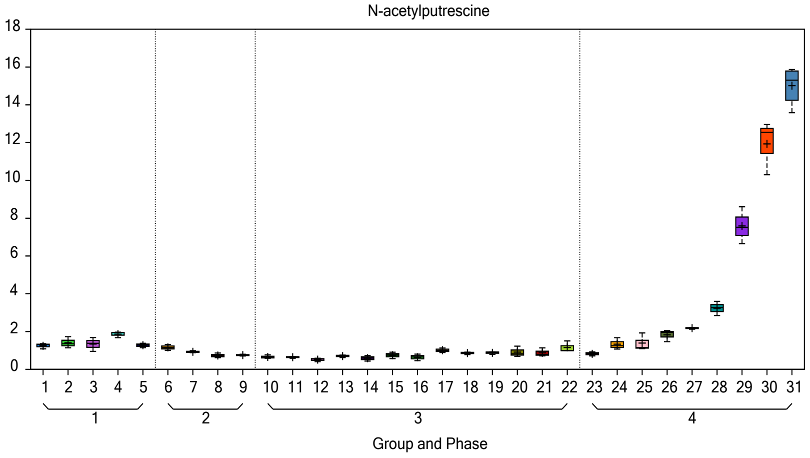
N-acetylputrescine
18
16
14
12
10
8
6
4
2
0
1 2 3 4 5 6 7 8 9 10 11 12 13 14 15 16 17 18 19 20 21 22 23 24 25 26 27 28 29 30 31
1
2
3
4
Group and Phase

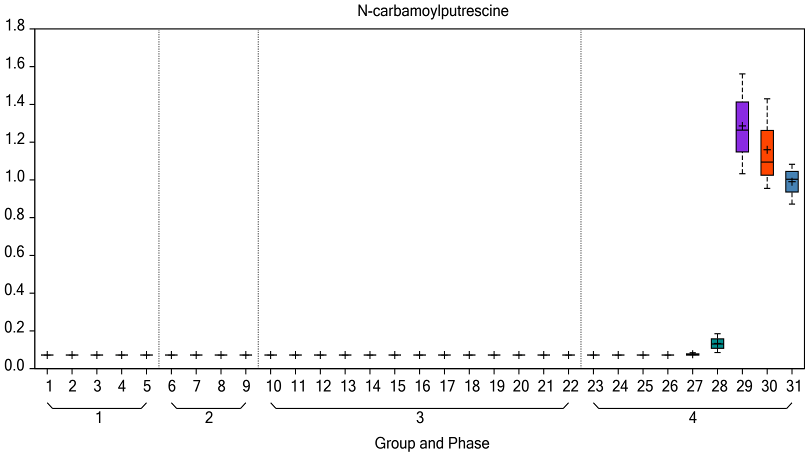
N-carbamoylputrescine
1.8
1.6
1.4
1.2
1.0
0.8
0.6
0.4
0.2
0.0
1 2 3 4 5 6 7 8 9 10 11 12 13 14 15 16 17 18 19 20 21 22 23 24 25 26 27 28 29 30 31
1
2
3
4
Group and Phase

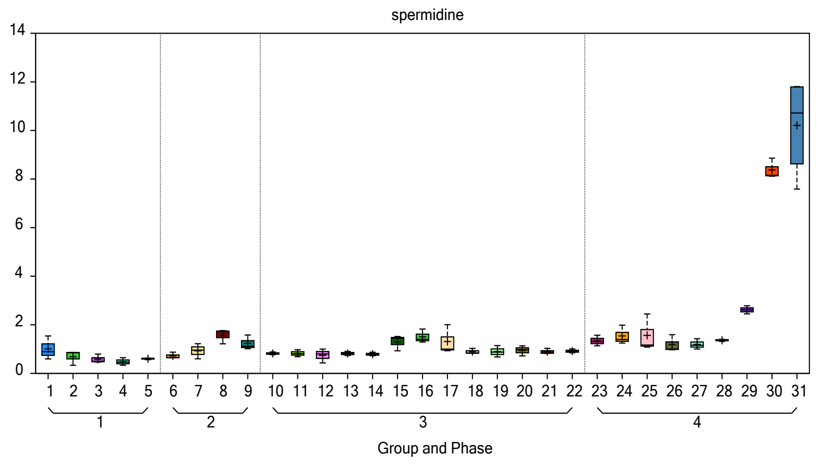
spermidine
Group and Phase

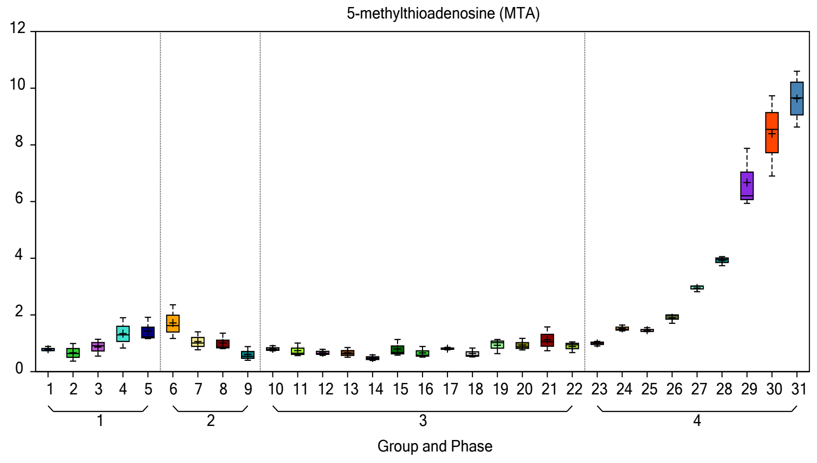
5-methylthioadenosine (MTA)
Group and Phase

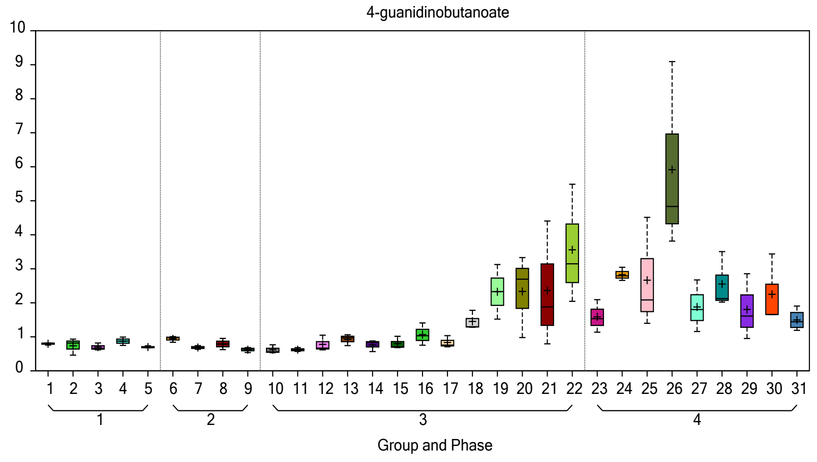
4-guanidinobutanoate
Group and Phase

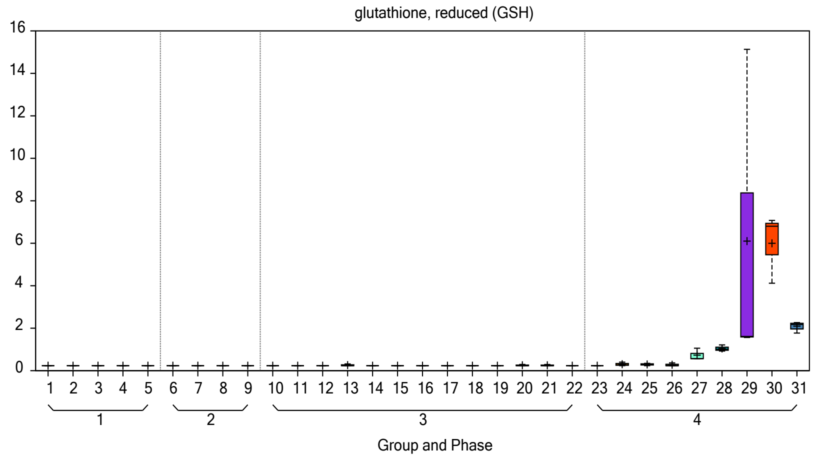
glutathione, reduced (GSH)
Group and Phase

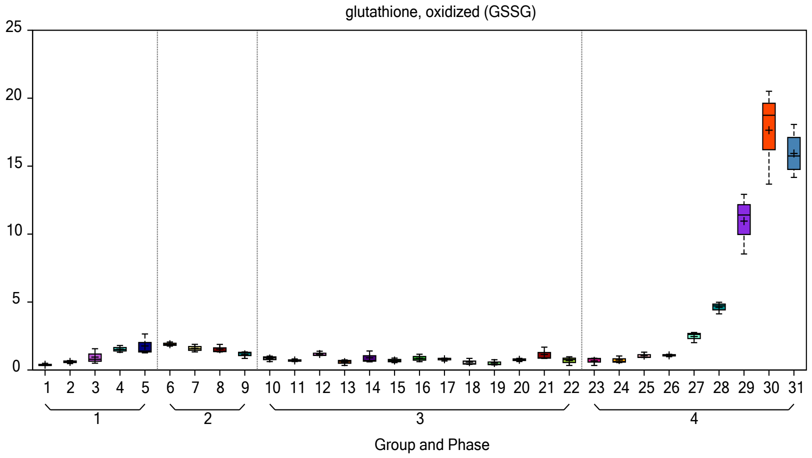
glutathione, oxidized (GSSG)
0
5
10
15
20
25
1 2 3 4 5 6 7 8 9 10 11 12 13 14 15 16 17 18 19 20 21 22 23 24 25 26 27 28 29 30 31
1
2
3
4
Group and Phase

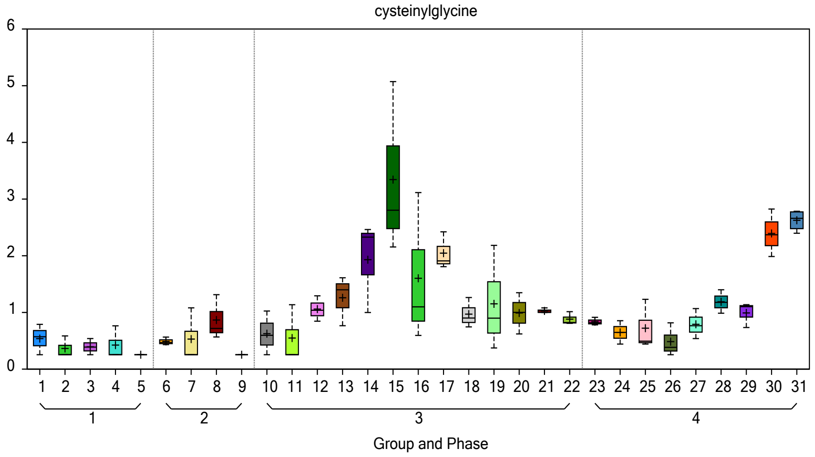
cysteinylglycine
0
1
2
3
4
5
6
1 2 3 4 5 6 7 8 9 10 11 12 13 14 15 16 17 18 19 20 21 22 23 24 25 26 27 28 29 30 31
1
2
3
4
Group and Phase

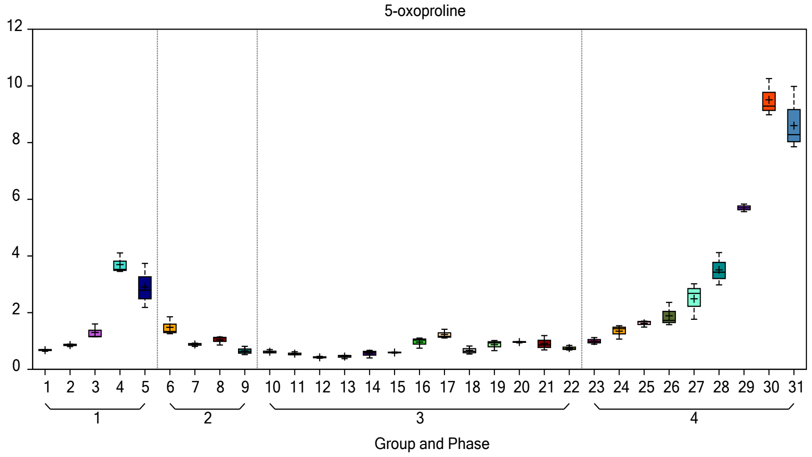
5-oxoproline
0
2
4
6
8
10
12
1 2 3 4 5 6 7 8 9 10 11 12 13 14 15 16 17 18 19 20 21 22 23 24 25 26 27 28 29 30 31
1
2
3
4
Group and Phase

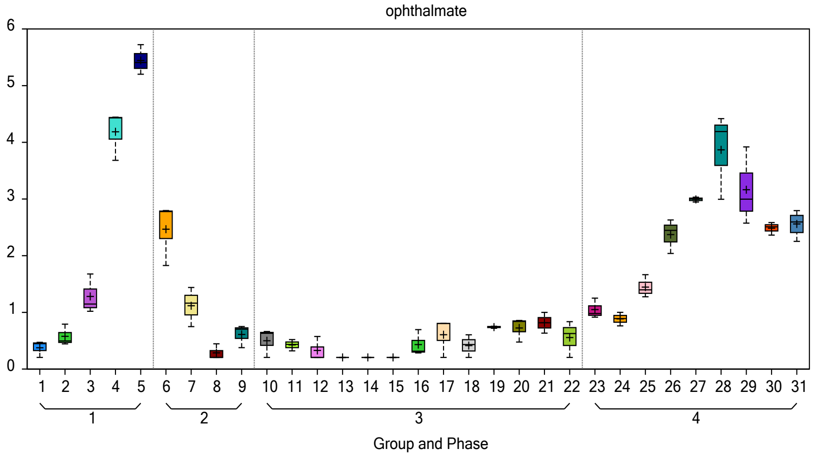
ophthalmate
0
1
2
3
4
5
6
1 2 3 4 5 6 7 8 9 10 11 12 13 14 15 16 17 18 19 20 21 22 23 24 25 26 27 28 29 30 31
1
2
3
4
Group and Phase

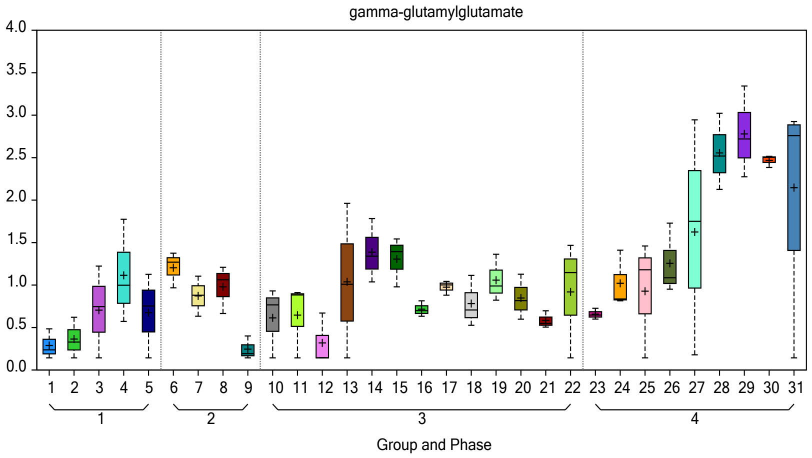
gamma-glutamylglutamate
4.0
3.5
3.0
2.5
2.0
1.5
1.0
0.5
0.0
1 2 3 4 5 6 7 8 9 10 11 12 13 14 15 16 17 18 19 20 21 22 23 24 25 26 27 28 29 30 31
1 2 3 4
Group and Phase

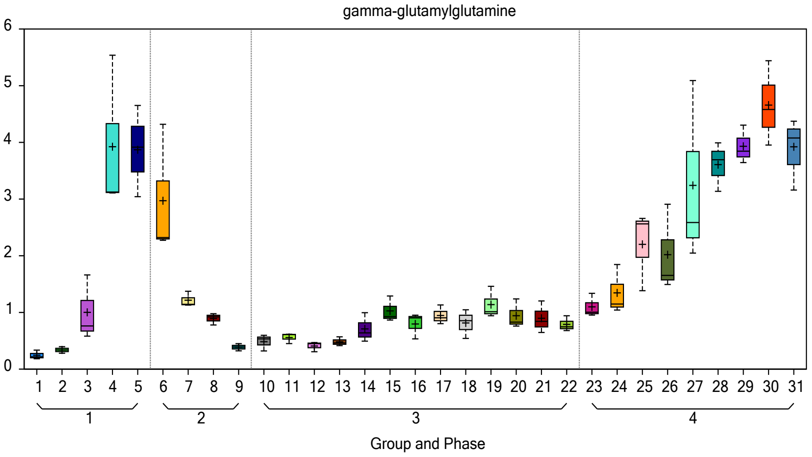
gamma-glutamylglutamine
6
5
4
3
2
1
0
1 2 3 4 5 6 7 8 9 10 11 12 13 14 15 16 17 18 19 20 21 22 23 24 25 26 27 28 29 30 31
1 2 3 4
Group and Phase

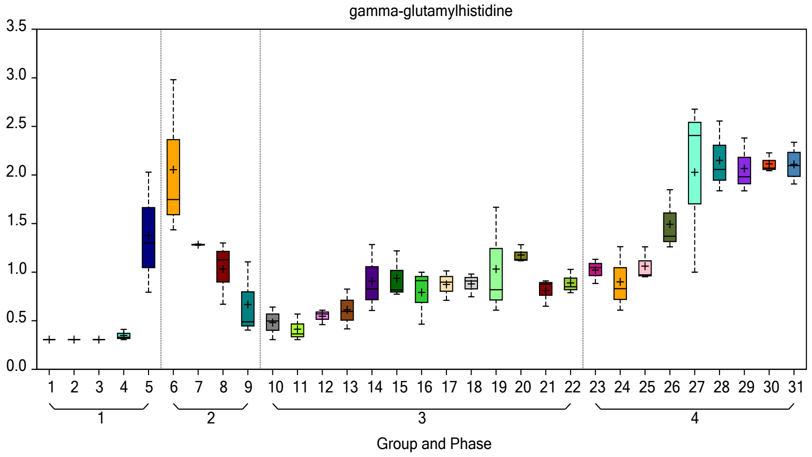
gamma-glutamylhistidine
3.5
3.0
2.5
2.0
1.5
1.0
0.5
0.0
1 2 3 4 5 6 7 8 9 10 11 12 13 14 15 16 17 18 19 20 21 22 23 24 25 26 27 28 29 30 31
1 2 3 4
Group and Phase

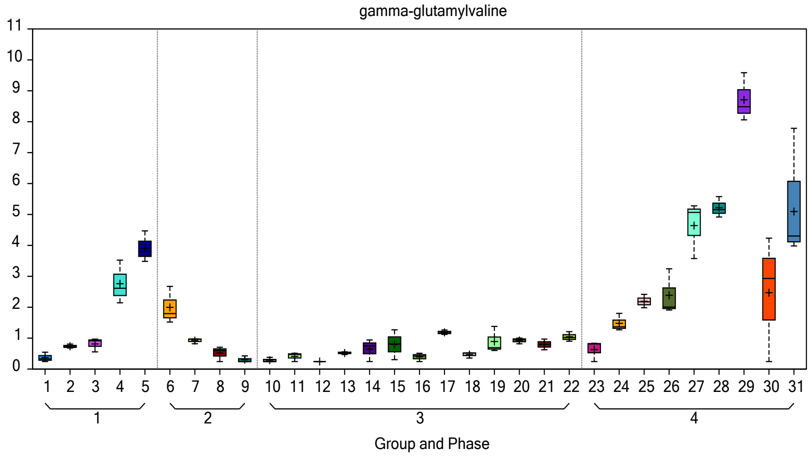
gamma-glutamylvaline
11
10
9
8
7
6
5
4
3
2
1
0
1 2 3 4 5 6 7 8 9 10 11 12 13 14 15 16 17 18 19 20 21 22 23 24 25 26 27 28 29 30 31
1 2 3 4
Group and Phase

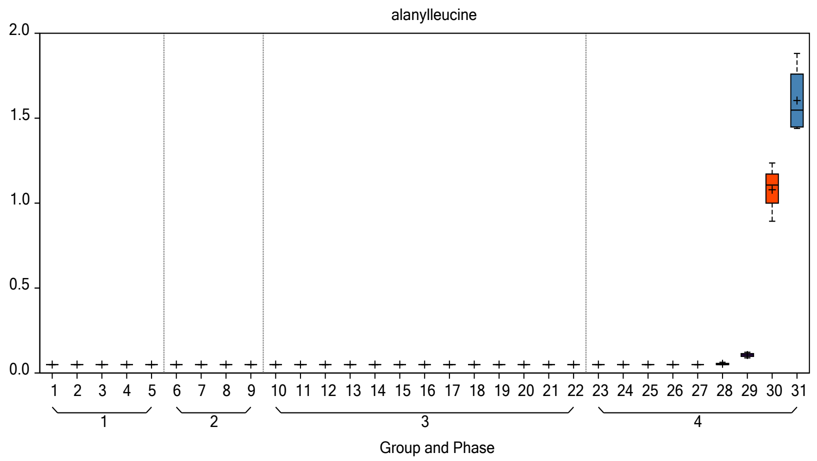
alanylleucine
2.0
1.5
1.0
0.5
0.0
1 2 3 4 5 6 7 8 9 10 11 12 13 14 15 16 17 18 19 20 21 22 23 24 25 26 27 28 29 30 31
1
2
3
4
Group and Phase

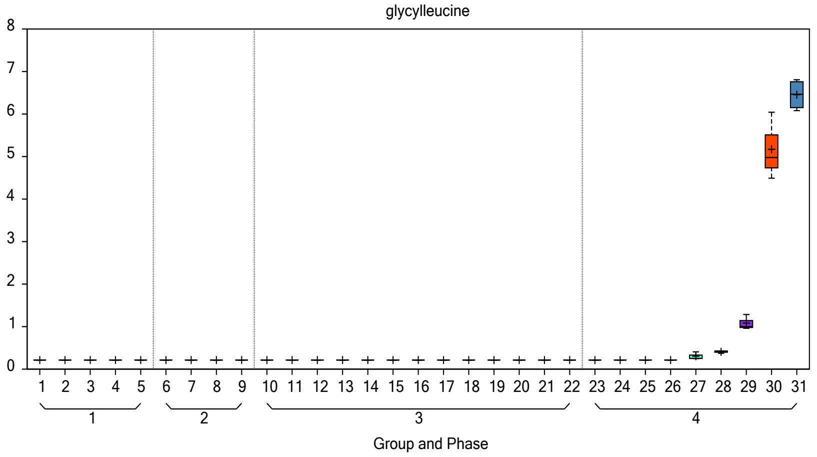
glycylleucine
8
7
6
5
4
3
2
1
0
1 2 3 4 5 6 7 8 9 10 11 12 13 14 15 16 17 18 19 20 21 22 23 24 25 26 27 28 29 30 31
1
2
3
4
Group and Phase

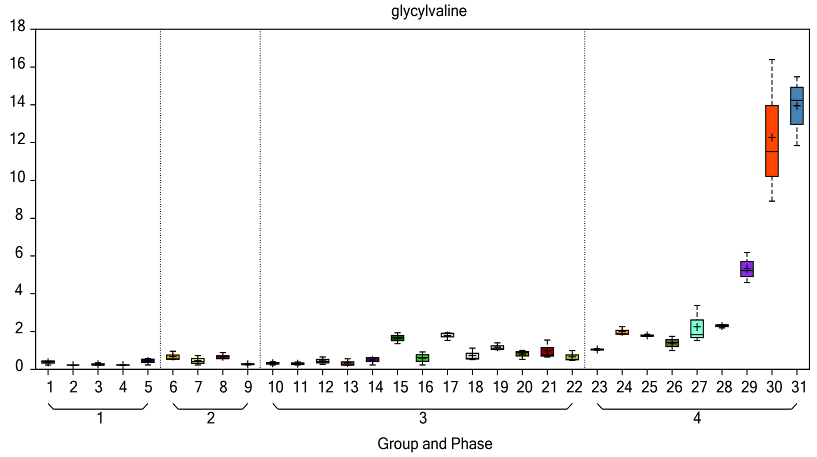
glycylvaline
18
16
14
12
10
8
6
4
2
0
1 2 3 4 5 6 7 8 9 10 11 12 13 14 15 16 17 18 19 20 21 22 23 24 25 26 27 28 29 30 31
1
2
3
4
Group and Phase

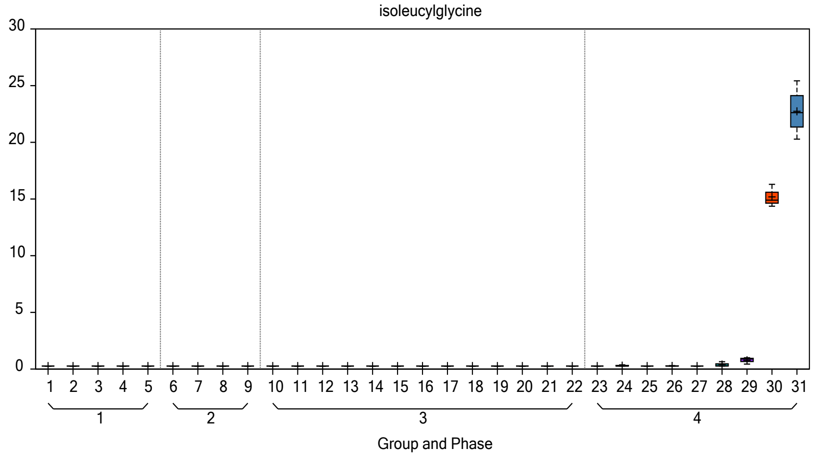
isoleucylglycine
30
25
20
15
10
5
0
1 2 3 4 5 6 7 8 9 10 11 12 13 14 15 16 17 18 19 20 21 22 23 24 25 26 27 28 29 30 31
1
2
3
4
Group and Phase

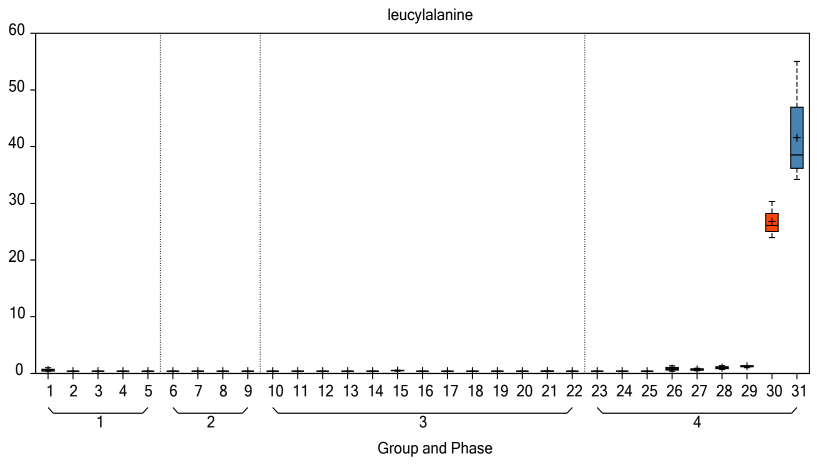
leucylalanine
60
50
40
30
20
10
0
1 2 3 4 5 6 7 8 9 10 11 12 13 14 15 16 17 18 19 20 21 22 23 24 25 26 27 28 29 30 31
1
2
3
4
Group and Phase

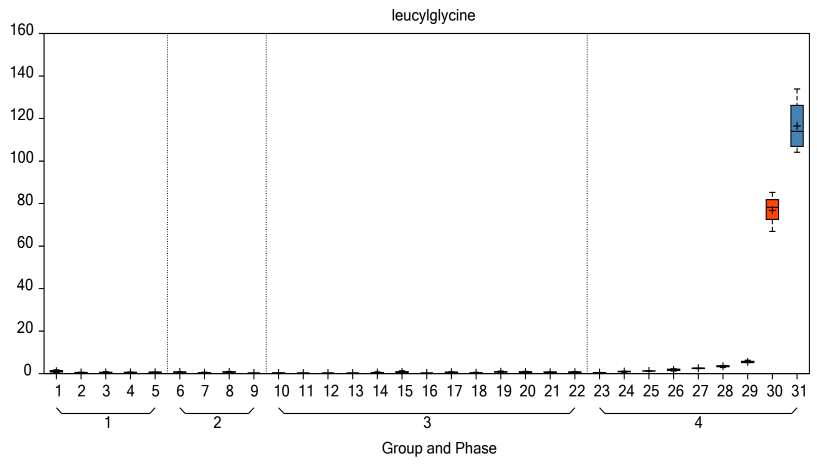
leucylglycine
160
140
120
100
80
60
40
20
0
1 2 3 4 5 6 7 8 9 10 11 12 13 14 15 16 17 18 19 20 21 22 23 24 25 26 27 28 29 30 31
1
2
3
4
Group and Phase

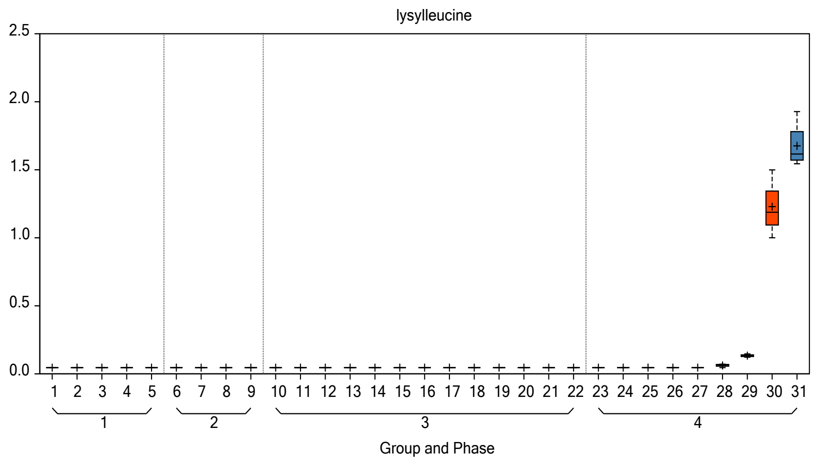
lysylleucine
2.5
2.0
1.5
1.0
0.5
0.0
1 2 3 4 5 6 7 8 9 10 11 12 13 14 15 16 17 18 19 20 21 22 23 24 25 26 27 28 29 30 31
1
2
3
4
Group and Phase

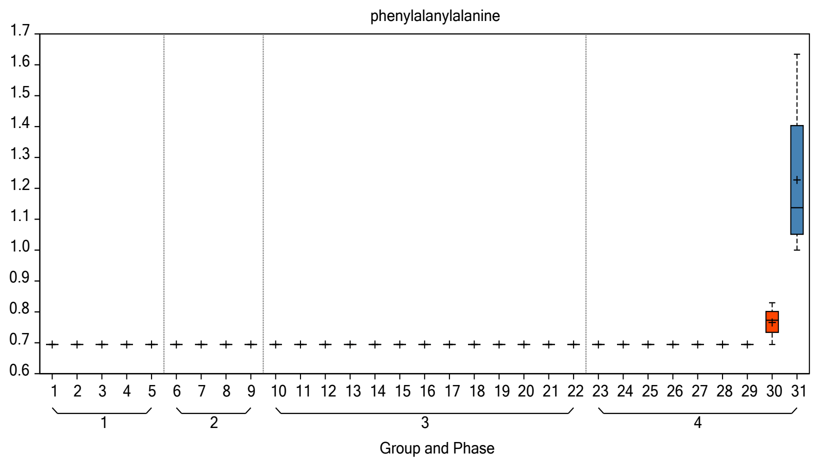
phenylalanylalanine
1.7
1.6
1.5
1.4
1.3
1.2
1.1
1.0
0.9
0.8
0.7
0.6
1 2 3 4 5 6 7 8 9 10 11 12 13 14 15 16 17 18 19 20 21 22 23 24 25 26 27 28 29 30 31
1
2
3
4
Group and Phase

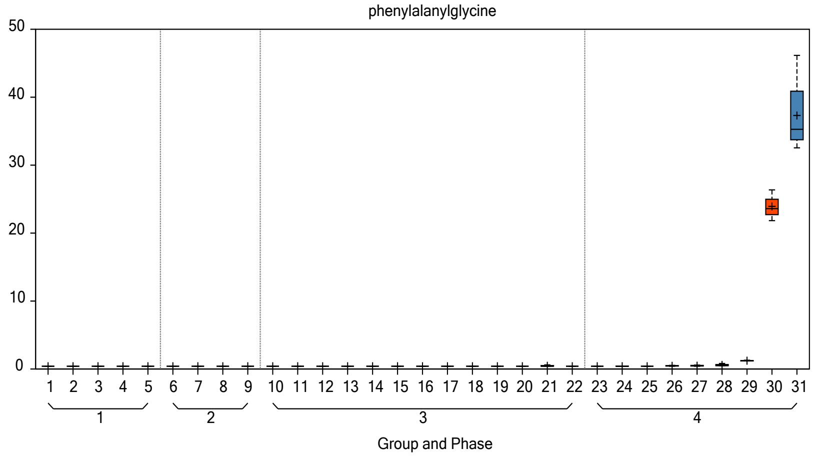
phenylalanylglycine
Group and Phase

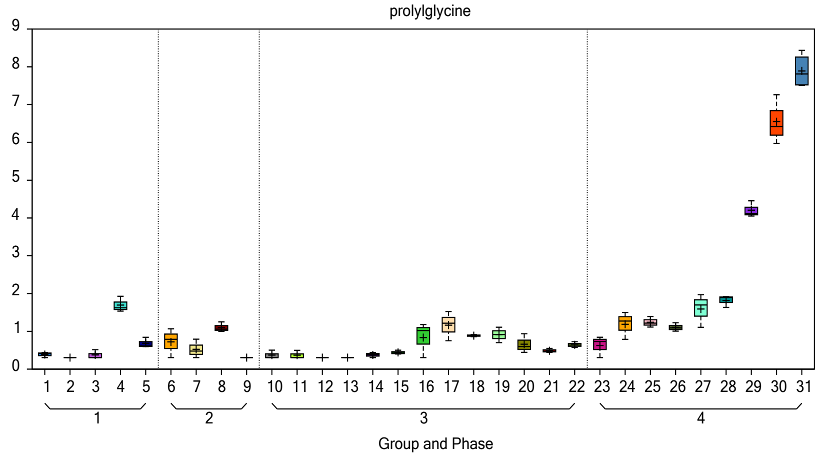
prolylglycine
Group and Phase

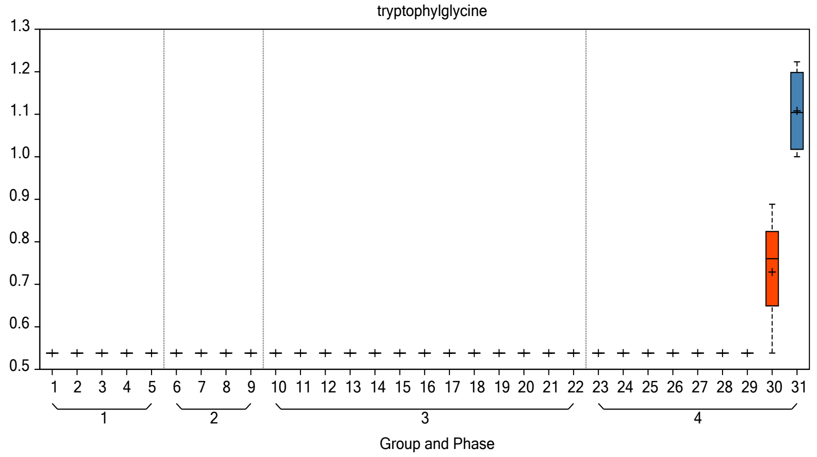
tryptophylglycine
Group and Phase

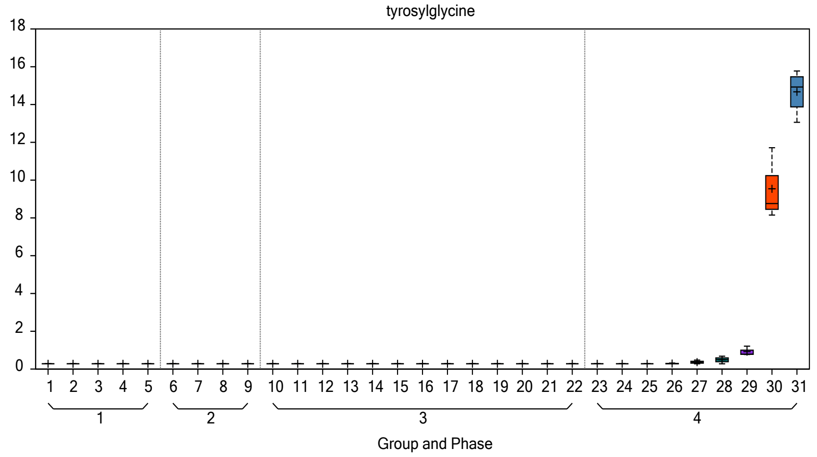
tyrosylglycine
Group and Phase

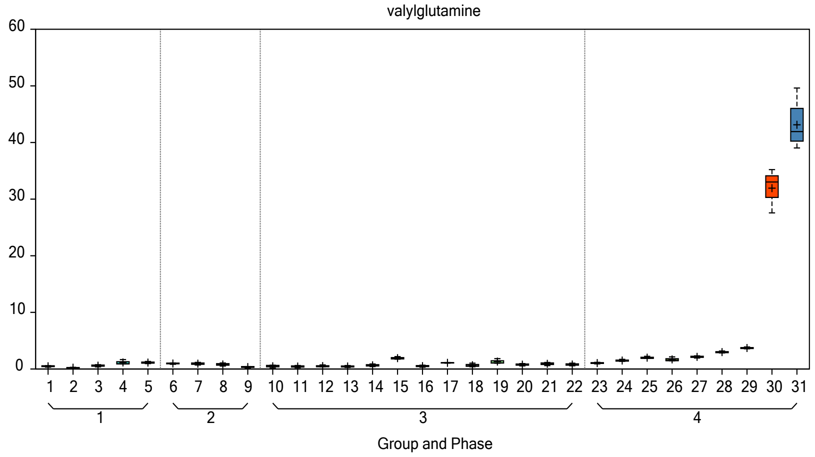
valylglutamine
0
10
20
30
40
50
60
1 2 3 4 5 6 7 8 9 10 11 12 13 14 15 16 17 18 19 20 21 22 23 24 25 26 27 28 29 30 31
1
2
3
4
Group and Phase

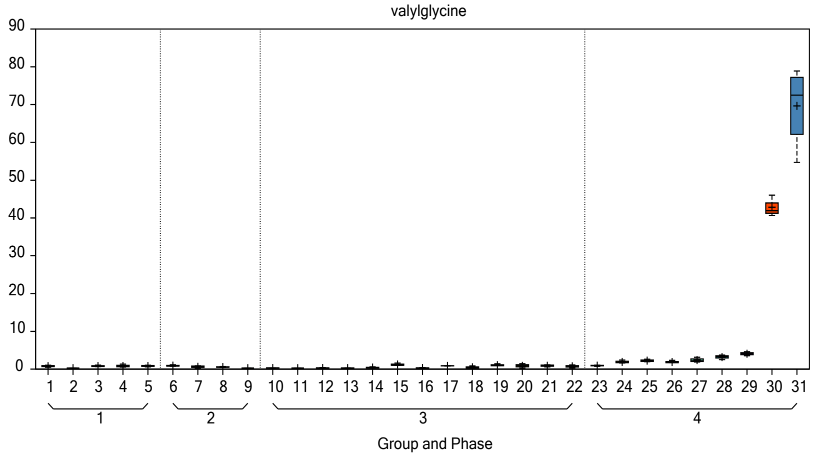
valylglycine
0
10
20
30
40
50
60
70
80
90
1 2 3 4 5 6 7 8 9 10 11 12 13 14 15 16 17 18 19 20 21 22 23 24 25 26 27 28 29 30 31
1
2
3
4
Group and Phase

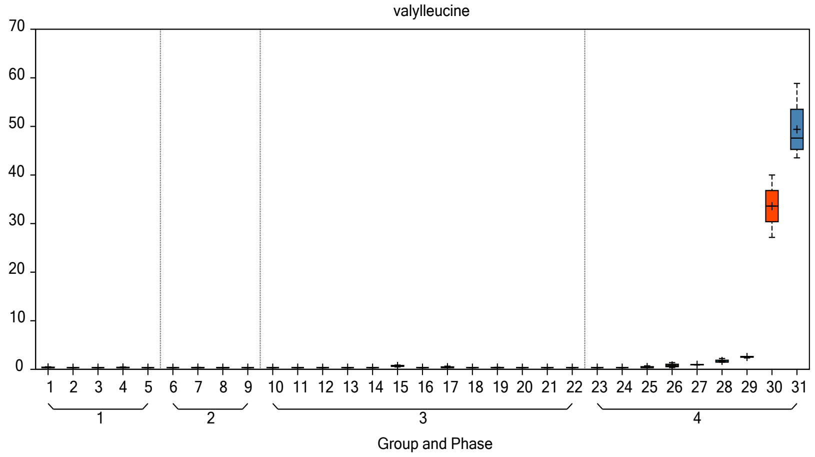
valylleucine
0
10
20
30
40
50
60
70
1 2 3 4 5 6 7 8 9 10 11 12 13 14 15 16 17 18 19 20 21 22 23 24 25 26 27 28 29 30 31
1
2
3
4
Group and Phase

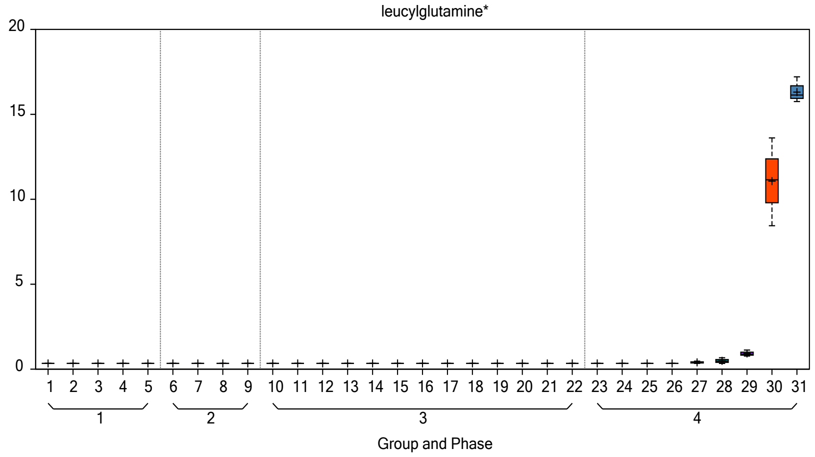
leucylglutamine*
0
5
10
15
20
1 2 3 4 5 6 7 8 9 10 11 12 13 14 15 16 17 18 19 20 21 22 23 24 25 26 27 28 29 30 31
1
2
3
4
Group and Phase

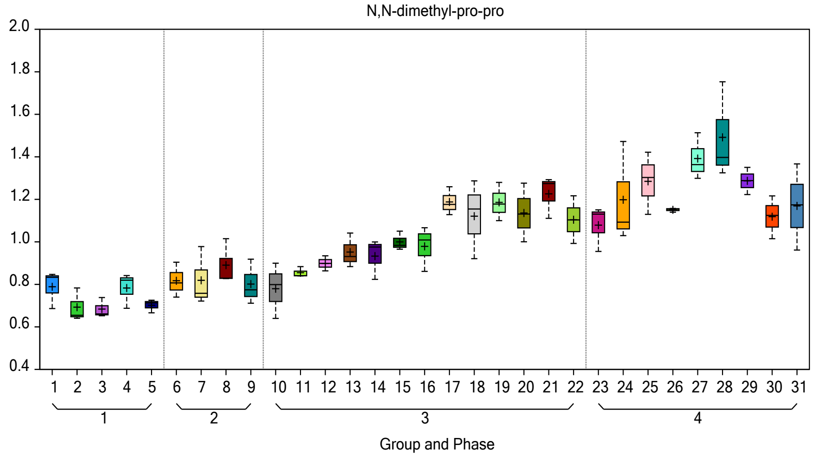
N,N-dimethyl-pro-pro
2.0
1.8
1.6
1.4
1.2
1.0
0.8
0.6
0.4
1 2 3 4 5 6 7 8 9 10 11 12 13 14 15 16 17 18 19 20 21 22 23 24 25 26 27 28 29 30 31
1
2
3
4
Group and Phase

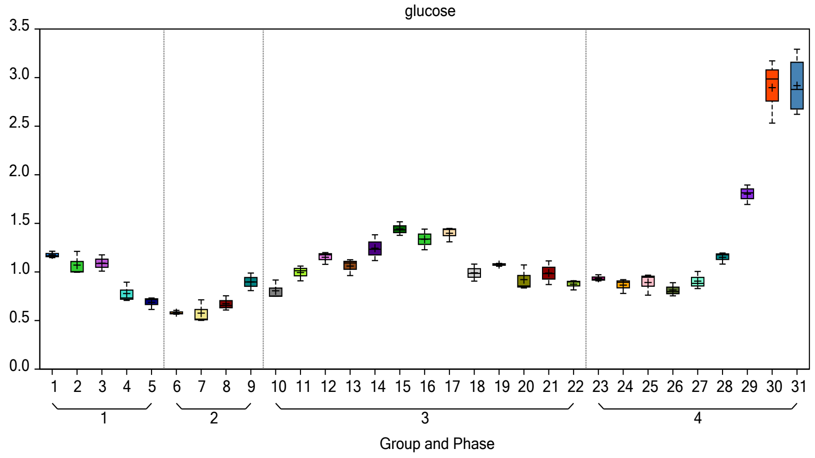
glucose
3.5
3.0
2.5
2.0
1.5
1.0
0.5
0.0
1 2 3 4 5 6 7 8 9 10 11 12 13 14 15 16 17 18 19 20 21 22 23 24 25 26 27 28 29 30 31
1
2
3
4
Group and Phase

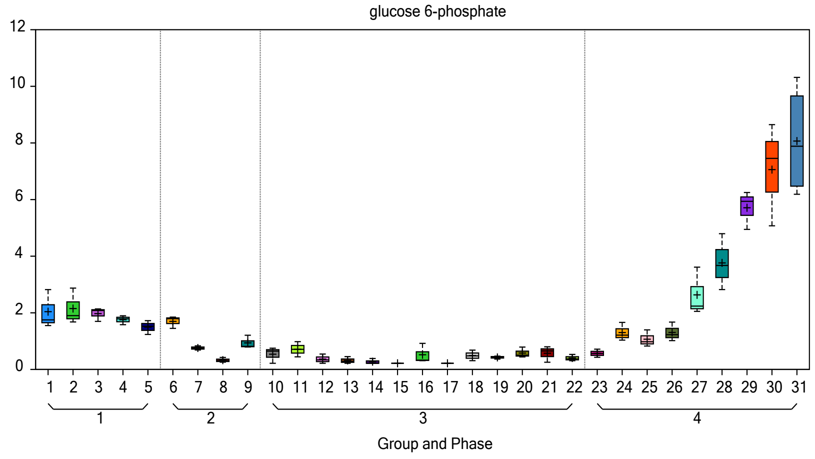
glucose 6-phosphate
12
10
8
6
4
2
0
1 2 3 4 5 6 7 8 9 10 11 12 13 14 15 16 17 18 19 20 21 22 23 24 25 26 27 28 29 30 31
1
2
3
4
Group and Phase

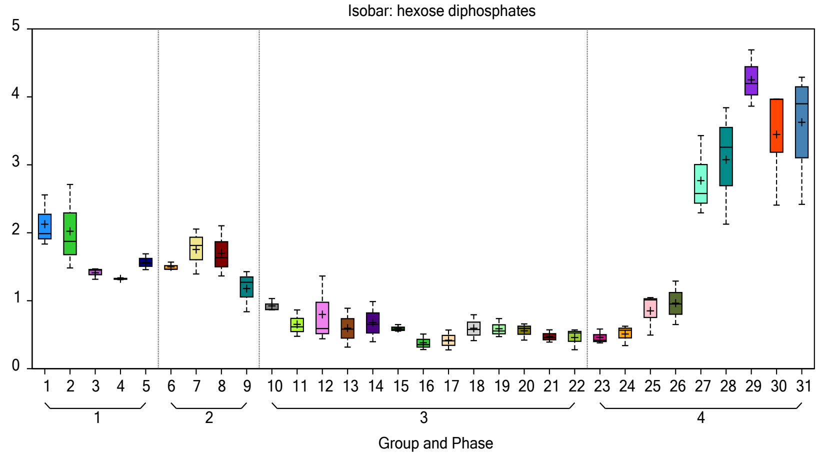
Isobar: hexose diphosphates
5
4
3
2
1
0
1 2 3 4 5 6 7 8 9 10 11 12 13 14 15 16 17 18 19 20 21 22 23 24 25 26 27 28 29 30 31
1
2
3
4
Group and Phase

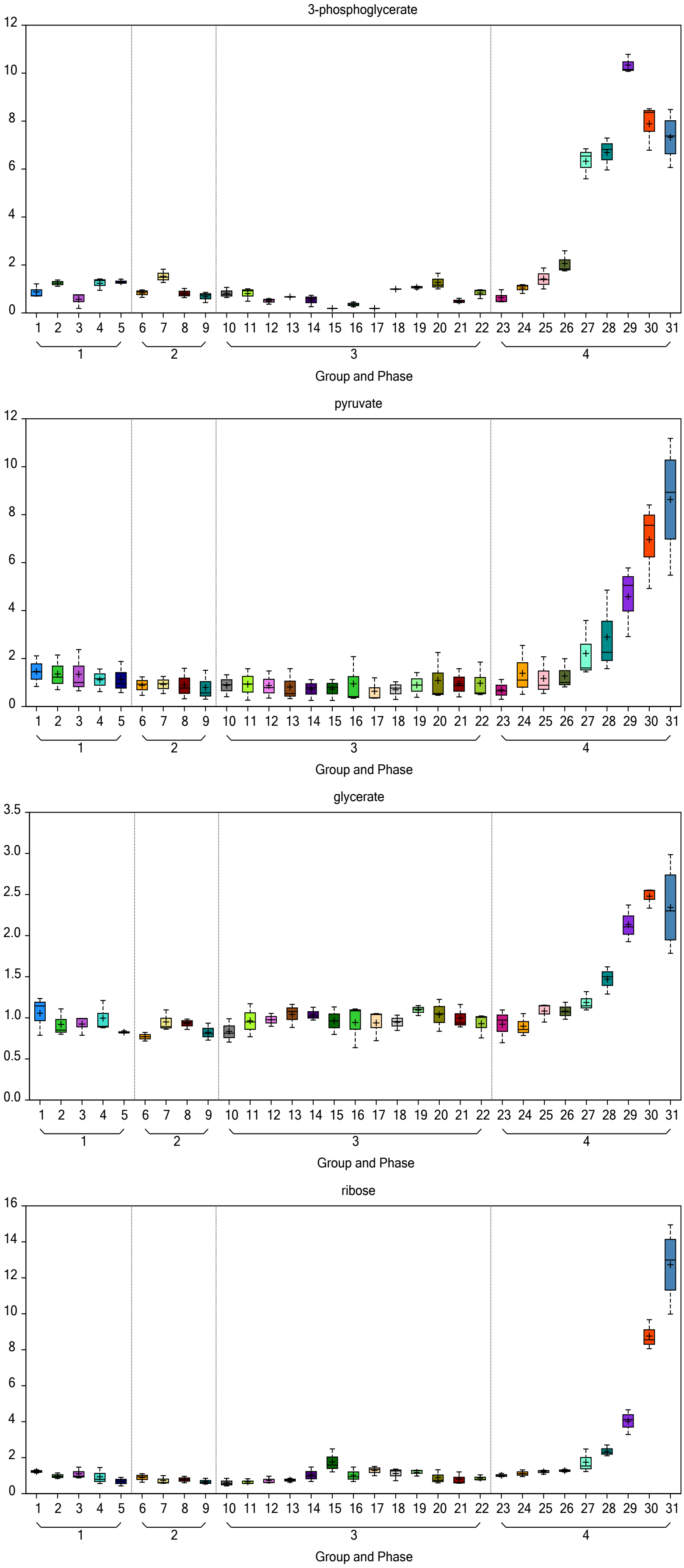
3-phosphoglycerate
Group and Phase
pyruvate
Group and Phase
glycerate
Group and Phase
ribose
Group and Phase

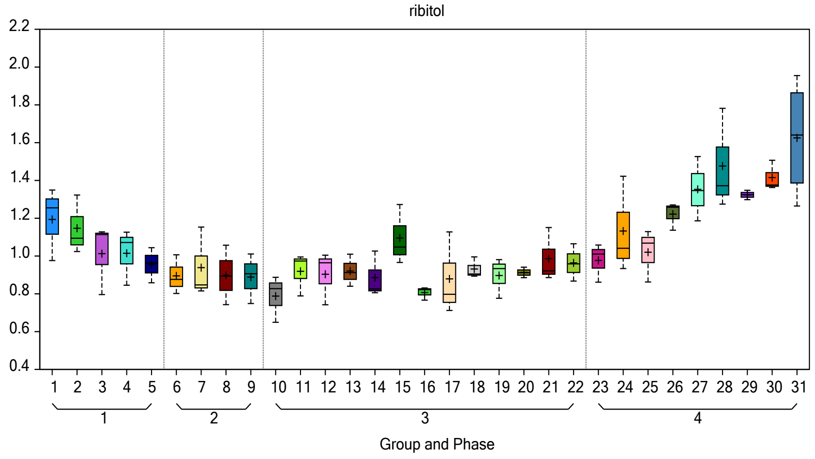
ribitol
2.2
2.0
1.8
1.6
1.4
1.2
1.0
0.8
0.6
0.4
1 2 3 4 5 6 7 8 9 10 11 12 13 14 15 16 17 18 19 20 21 22 23 24 25 26 27 28 29 30 31
1
2
3
4
Group and Phase

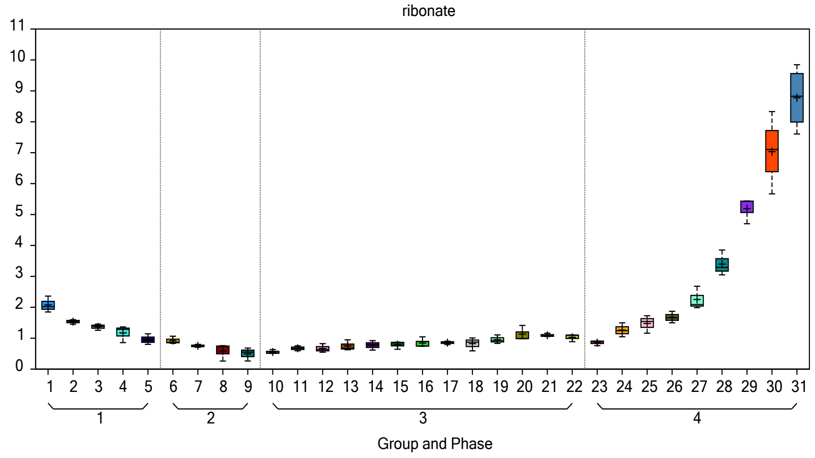
ribonate
11
10
9
8
7
6
5
4
3
2
1
0
1 2 3 4 5 6 7 8 9 10 11 12 13 14 15 16 17 18 19 20 21 22 23 24 25 26 27 28 29 30 31
1
2
3
4
Group and Phase

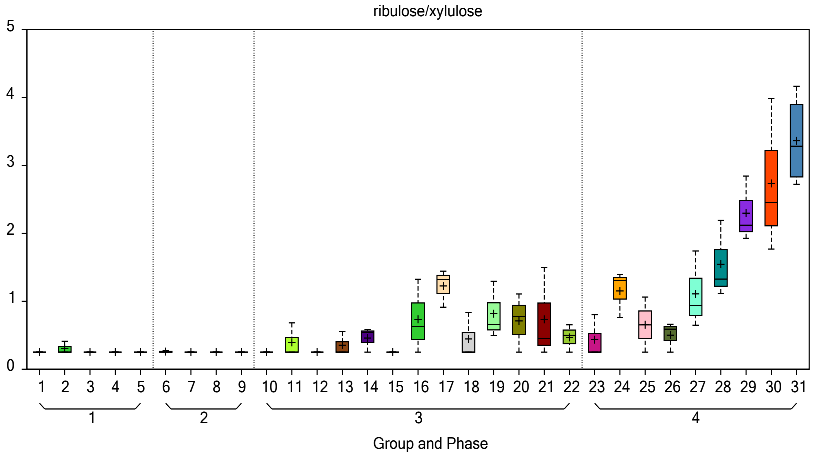
ribulose/xylulose
5
4
3
2
1
0
1 2 3 4 5 6 7 8 9 10 11 12 13 14 15 16 17 18 19 20 21 22 23 24 25 26 27 28 29 30 31
1
2
3
4
Group and Phase

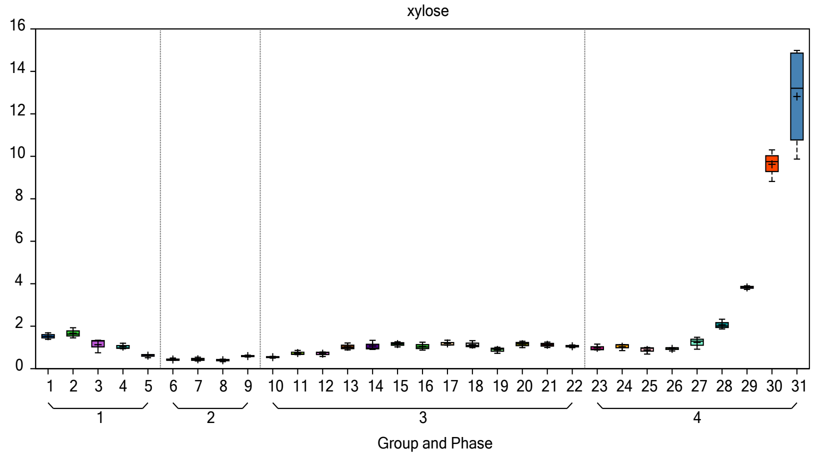
xylose
16
14
12
10
8
6
4
2
0
1 2 3 4 5 6 7 8 9 10 11 12 13 14 15 16 17 18 19 20 21 22 23 24 25 26 27 28 29 30 31
1
2
3
4
Group and Phase

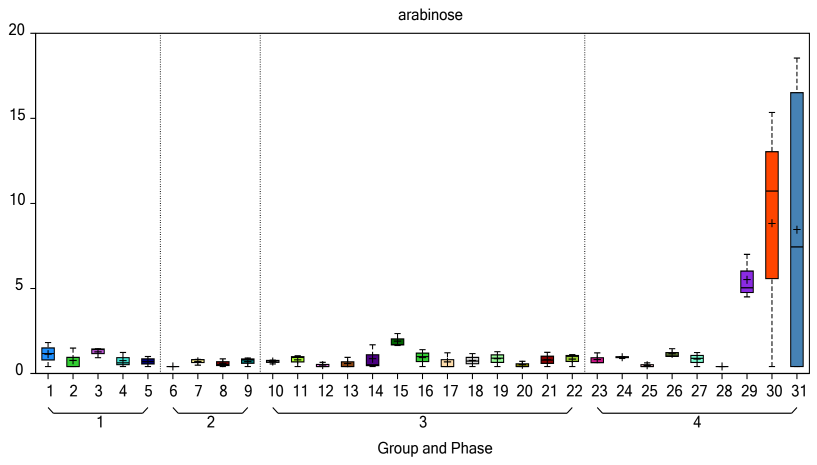
arabinose
20
15
10
5
0
1 2 3 4 5 6 7 8 9 10 11 12 13 14 15 16 17 18 19 20 21 22 23 24 25 26 27 28 29 30 31
1
2
3
4
Group and Phase

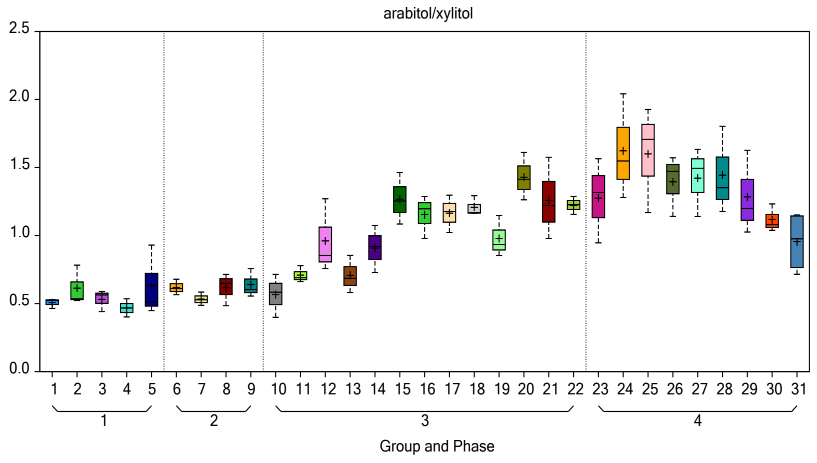
arabitol/xylitol
2.5
2.0
1.5
1.0
0.5
0.0
1 2 3 4 5 6 7 8 9 10 11 12 13 14 15 16 17 18 19 20 21 22 23 24 25 26 27 28 29 30 31
1
2
3
4
Group and Phase

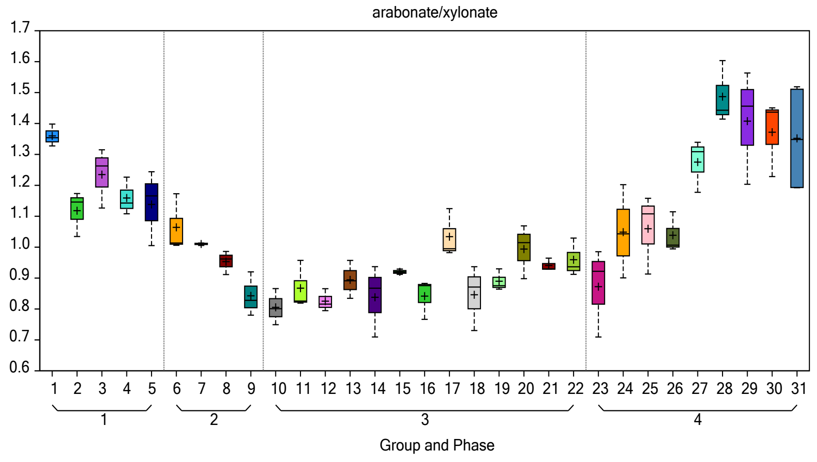
arabonate/xylonate
1.7
1.6
1.5
1.4
1.3
1.2
1.1
1.0
0.9
0.8
0.7
0.6
1 2 3 4 5 6 7 8 9 10 11 12 13 14 15 16 17 18 19 20 21 22 23 24 25 26 27 28 29 30 31
1
2
3
4
Group and Phase

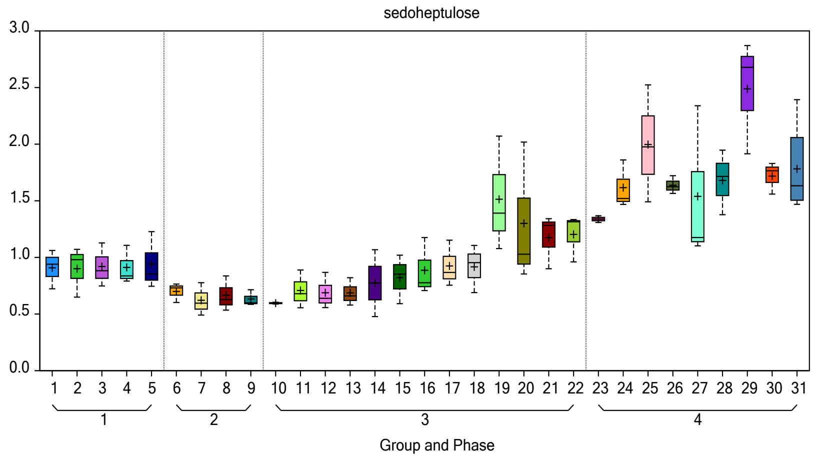
sedoheptulose
3.0
2.5
2.0
1.5
1.0
0.5
0.0
1 2 3 4 5 6 7 8 9 10 11 12 13 14 15 16 17 18 19 20 21 22 23 24 25 26 27 28 29 30 31
1
2
3
4
Group and Phase

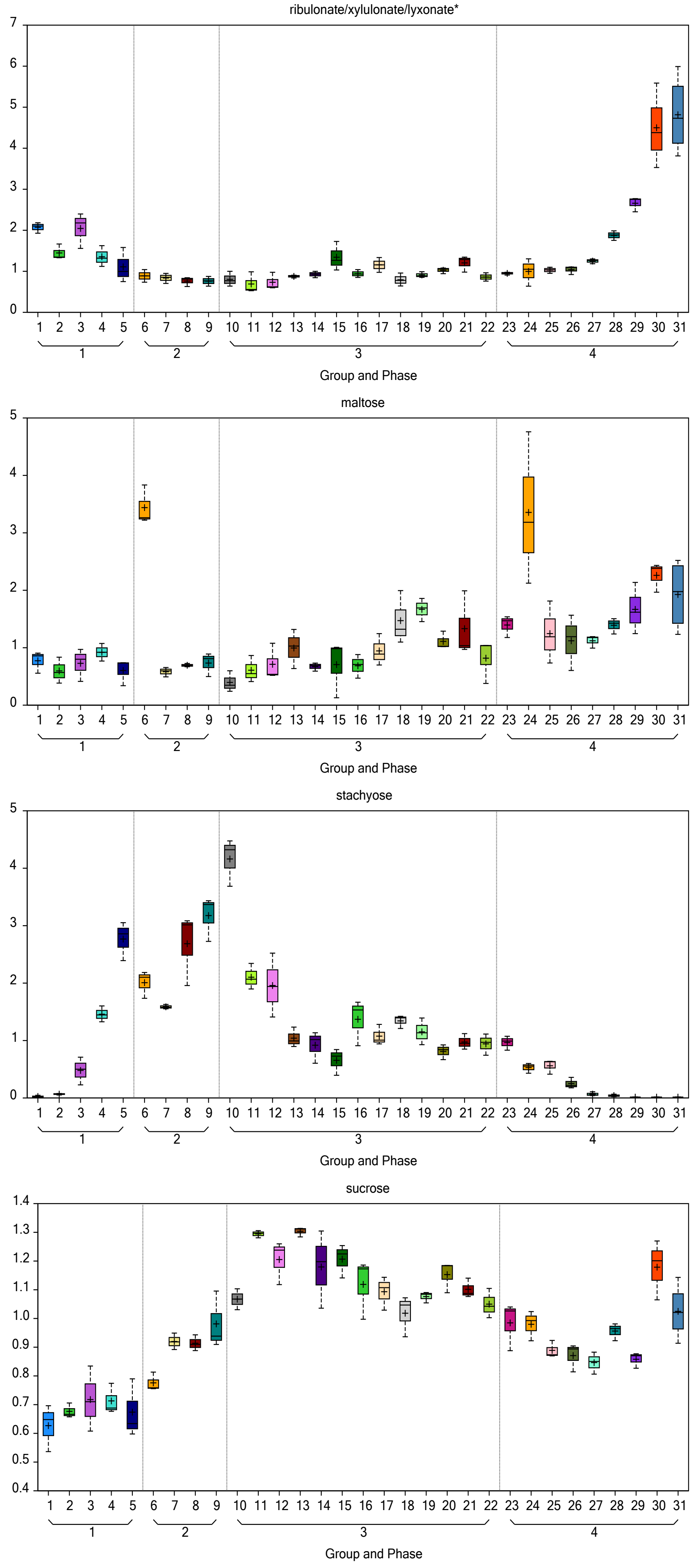

ribulonate/xylulonate/lyxonate*
maltose
stachyose
sucrose
Group and Phase

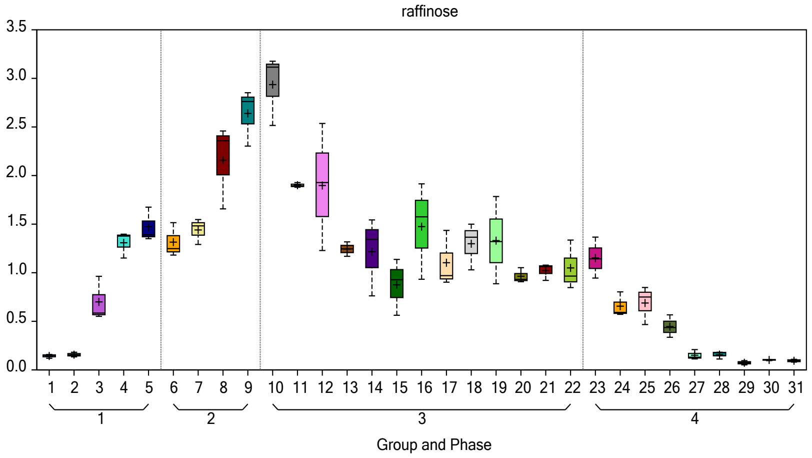
raffinose
3.5
3.0
2.5
2.0
1.5
1.0
0.5
0.0
1 2 3 4 5 6 7 8 9 10 11 12 13 14 15 16 17 18 19 20 21 22 23 24 25 26 27 28 29 30 31
1
2
3
4
Group and Phase

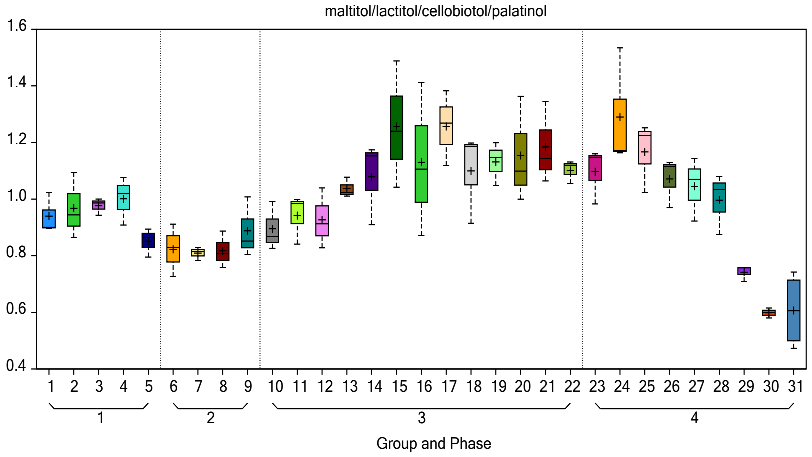
maltitol/lactitol/cellobiotol/palatinol
1.6
1.4
1.2
1.0
0.8
0.6
0.4
1 2 3 4 5 6 7 8 9 10 11 12 13 14 15 16 17 18 19 20 21 22 23 24 25 26 27 28 29 30 31
1
2
3
4
Group and Phase

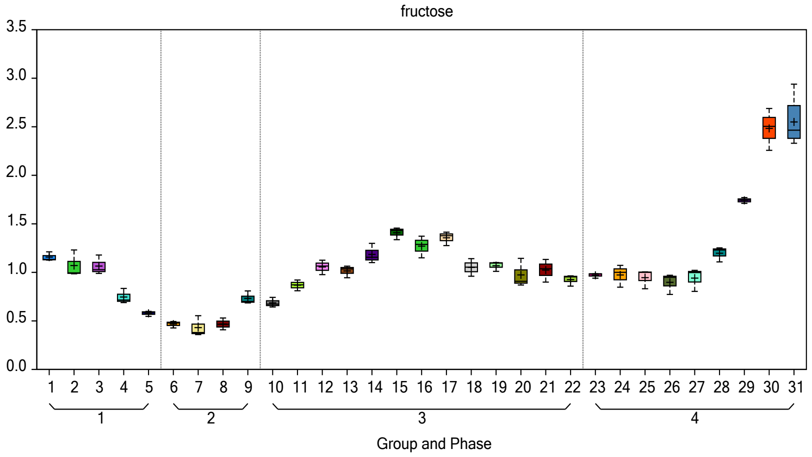
fructose
3.5
3.0
2.5
2.0
1.5
1.0
0.5
0.0
1 2 3 4 5 6 7 8 9 10 11 12 13 14 15 16 17 18 19 20 21 22 23 24 25 26 27 28 29 30 31
1
2
3
4
Group and Phase

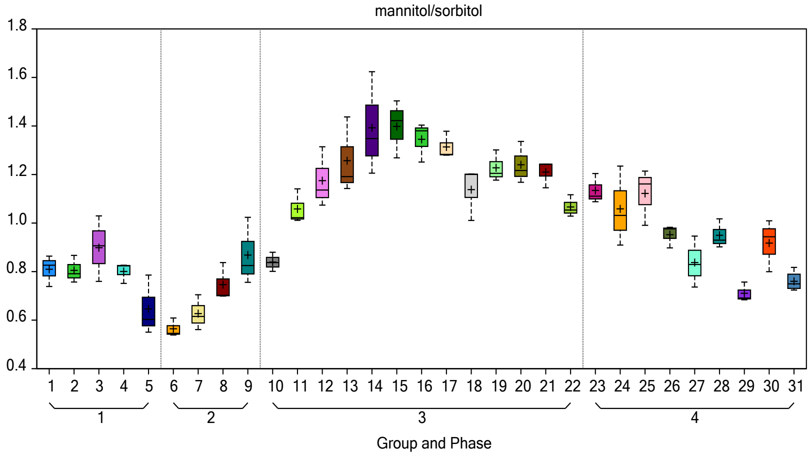
mannitol/sorbitol
1.8
1.6
1.4
1.2
1.0
0.8
0.6
0.4
1 2 3 4 5 6 7 8 9 10 11 12 13 14 15 16 17 18 19 20 21 22 23 24 25 26 27 28 29 30 31
1
2
3
4
Group and Phase

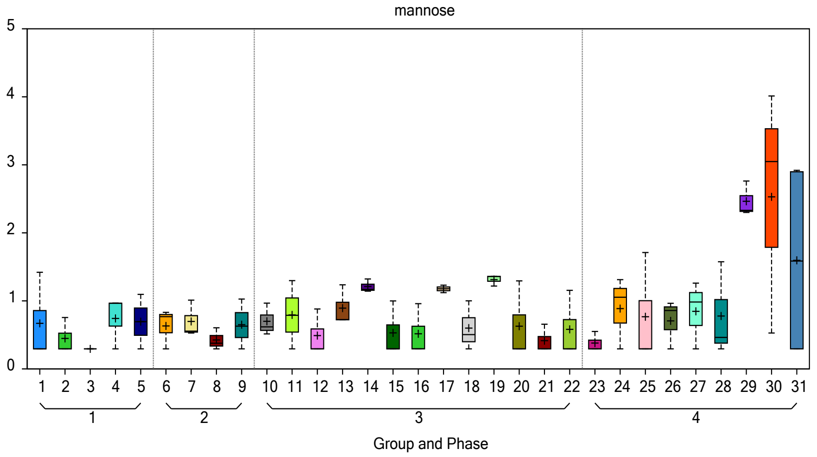
mannose
Group and Phase

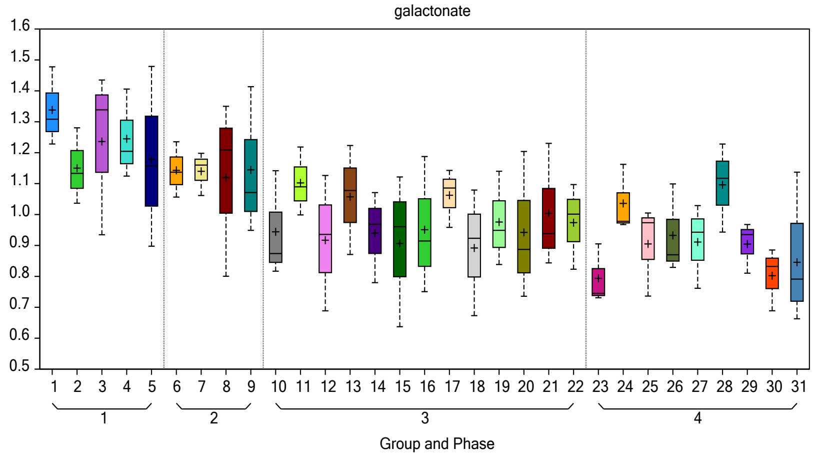
galactonate
Group and Phase

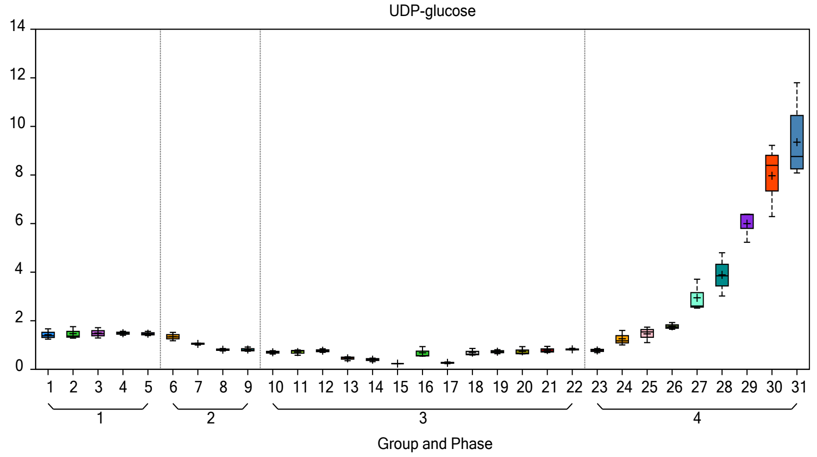
UDP-glucose
Group and Phase

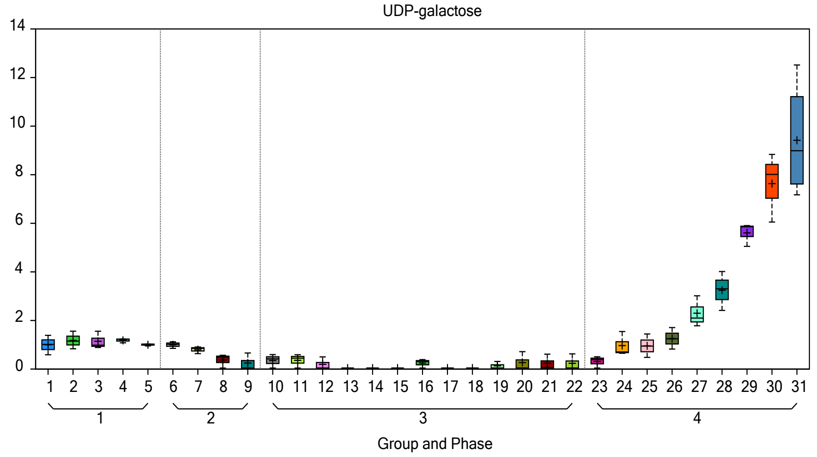
UDP-galactose
Group and Phase

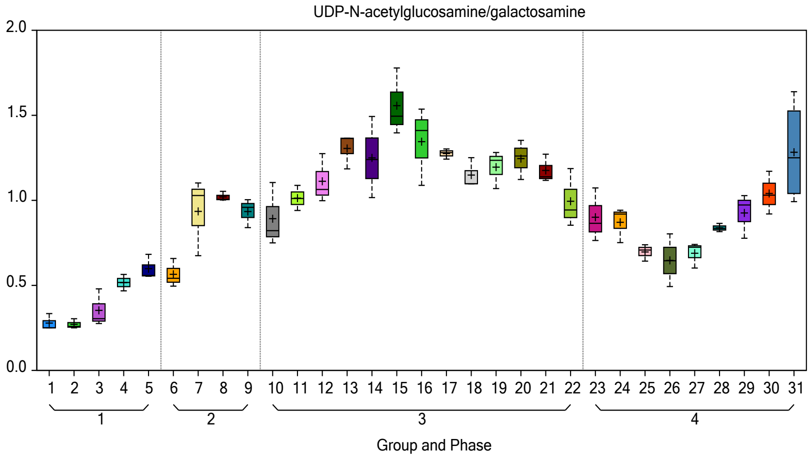
UDP-N-acetylglucosamine/galactosamine
2.0
1.5
1.0
0.5
0.0
1 2 3 4 5 6 7 8 9 10 11 12 13 14 15 16 17 18 19 20 21 22 23 24 25 26 27 28 29 30 31
1
2
3
4
Group and Phase

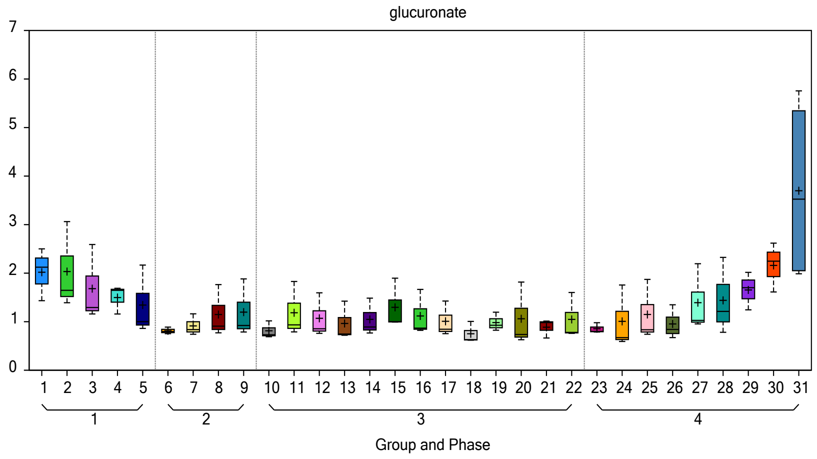
glucuronate
7
6
5
4
3
2
1
0
1 2 3 4 5 6 7 8 9 10 11 12 13 14 15 16 17 18 19 20 21 22 23 24 25 26 27 28 29 30 31
1
2
3
4
Group and Phase

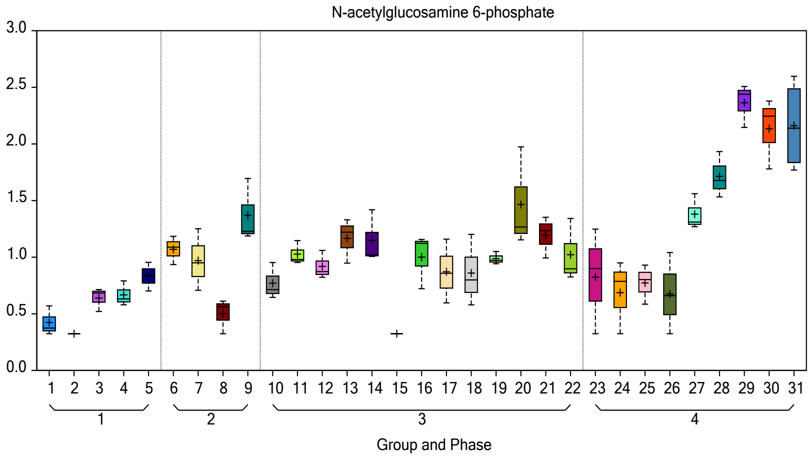
N-acetylglucosamine 6-phosphate
3.0
2.5
2.0
1.5
1.0
0.5
0.0
1 2 3 4 5 6 7 8 9 10 11 12 13 14 15 16 17 18 19 20 21 22 23 24 25 26 27 28 29 30 31
1
2
3
4
Group and Phase

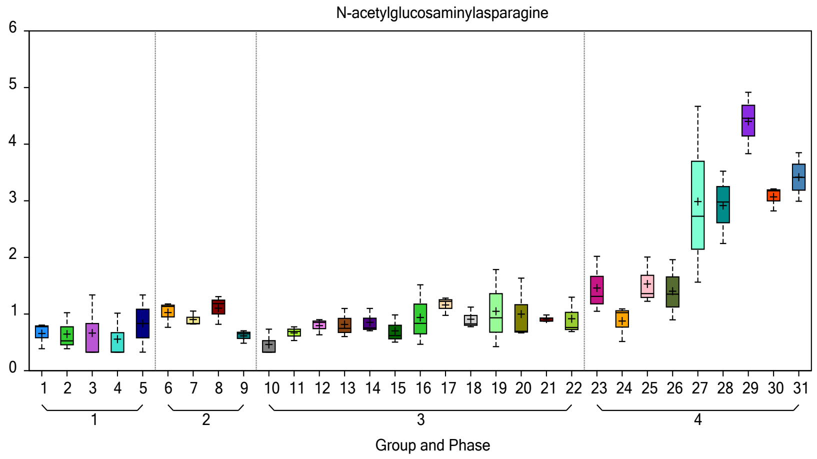
N-acetylglucosaminylasparagine
6
5
4
3
2
1
0
1 2 3 4 5 6 7 8 9 10 11 12 13 14 15 16 17 18 19 20 21 22 23 24 25 26 27 28 29 30 31
1
2
3
4
Group and Phase

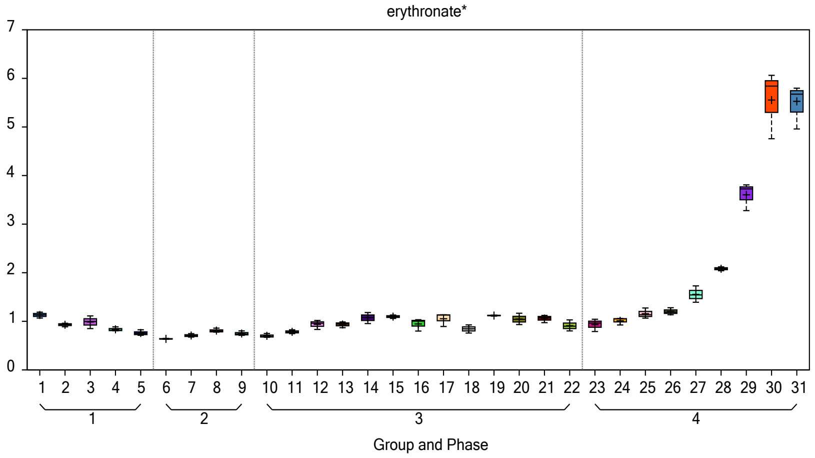
erythronate*
0 1 2 3 4 5 6 7
1 2 3 4 5 6 7 8 9 10 11 12 13 14 15 16 17 18 19 20 21 22 23 24 25 26 27 28 29 30 31
1 2 3 4
Group and Phase

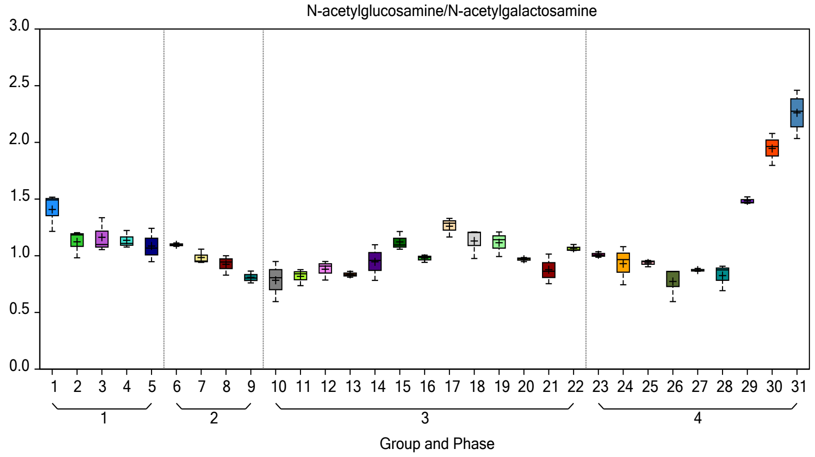
N-acetylglucosamine/N-acetylgalactosamine
0.0 0.5 1.0 1.5 2.0 2.5 3.0
1 2 3 4 5 6 7 8 9 10 11 12 13 14 15 16 17 18 19 20 21 22 23 24 25 26 27 28 29 30 31
1 2 3 4
Group and Phase

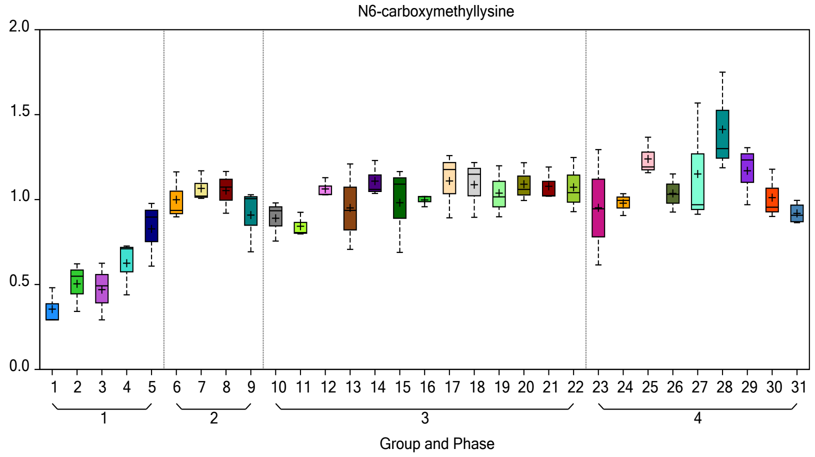
N6-carboxymethyllysine
0.0 0.5 1.0 1.5 2.0
1 2 3 4 5 6 7 8 9 10 11 12 13 14 15 16 17 18 19 20 21 22 23 24 25 26 27 28 29 30 31
1 2 3 4
Group and Phase

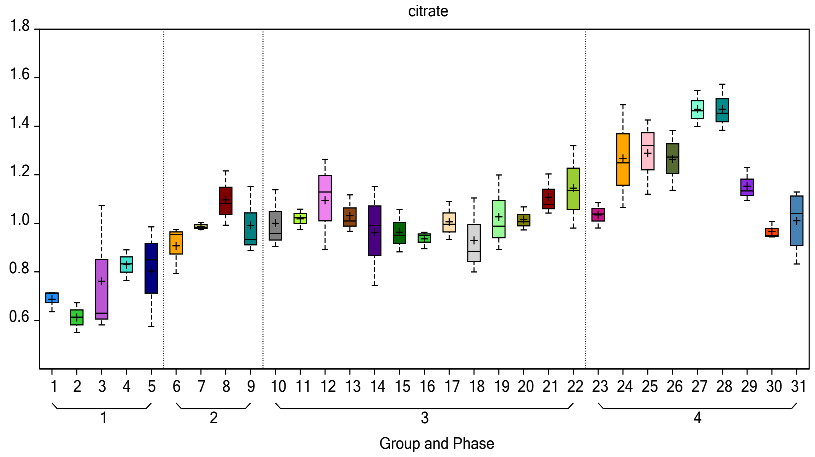
citrate
0.6 0.8 1.0 1.2 1.4 1.6 1.8
1 2 3 4 5 6 7 8 9 10 11 12 13 14 15 16 17 18 19 20 21 22 23 24 25 26 27 28 29 30 31
1 2 3 4
Group and Phase

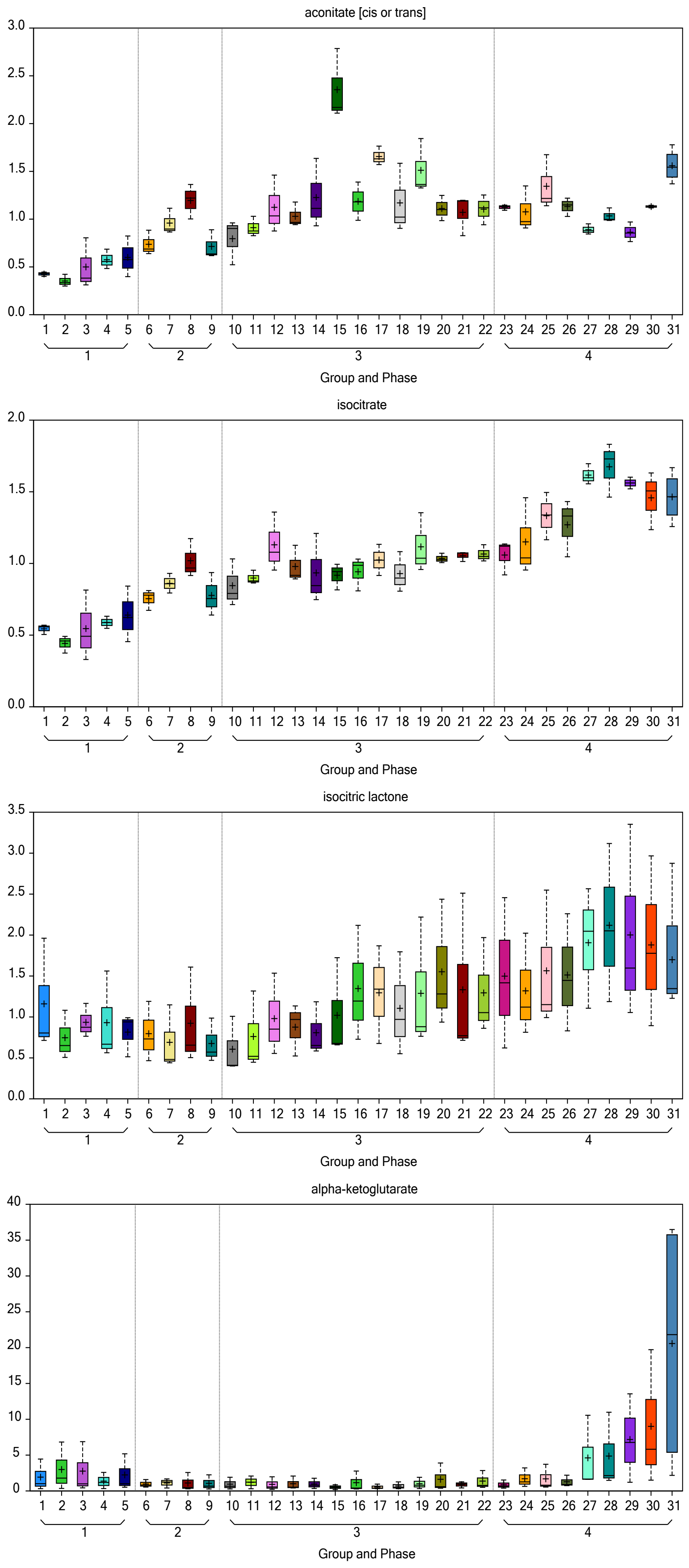
aconitate [cis or trans]
isocitrate
isocitric lactone
alpha-ketoglutarate
Group and Phase

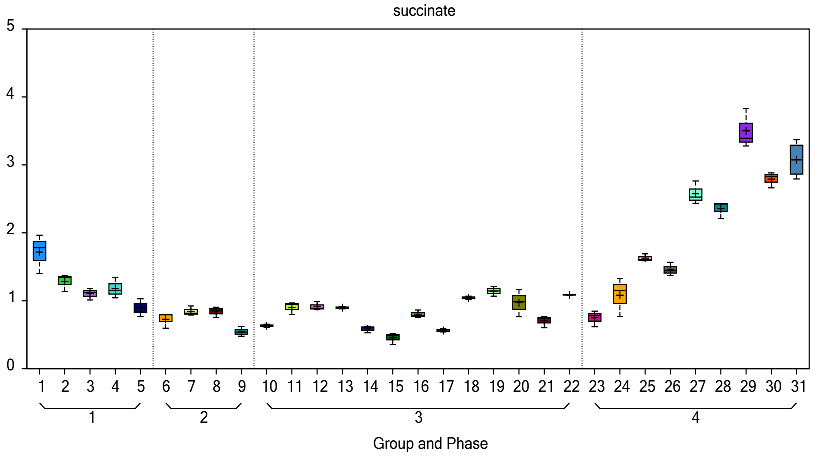
succinate
Group and Phase

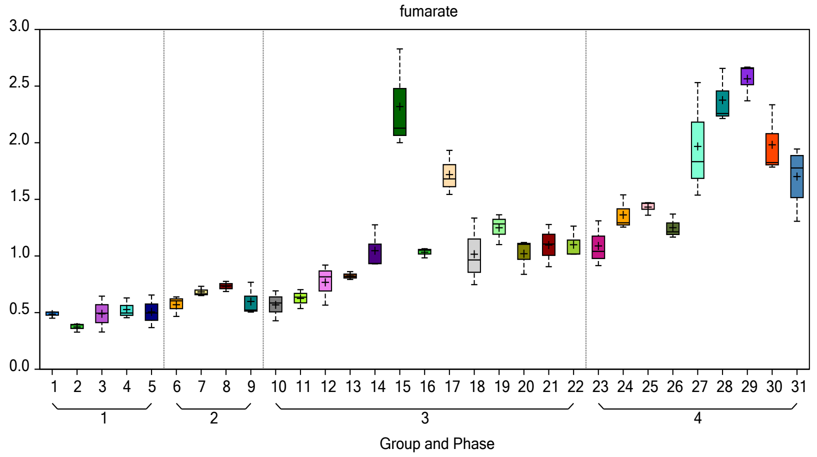
fumarate
Group and Phase

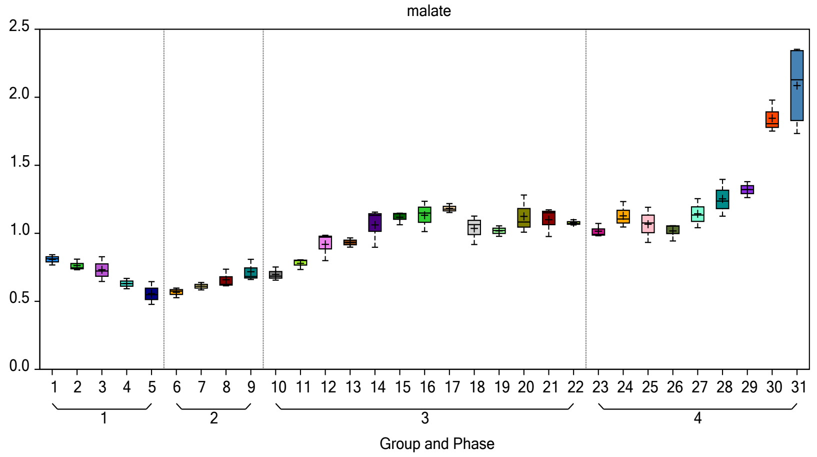
malate
Group and Phase

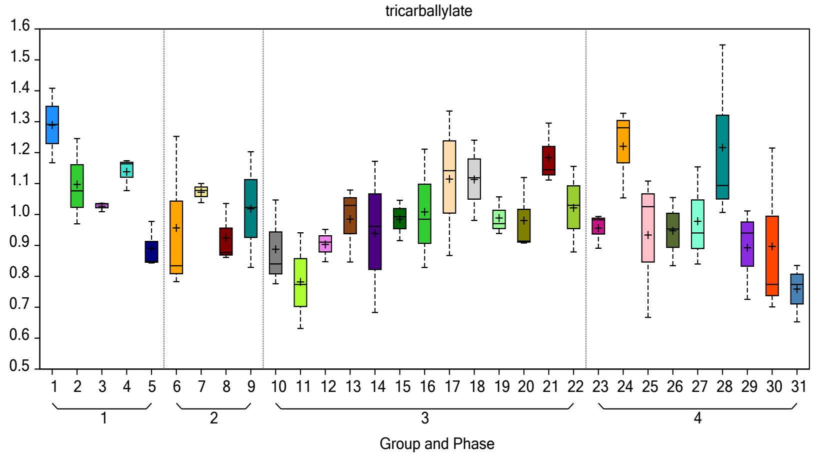
tricarballylate
Group and Phase

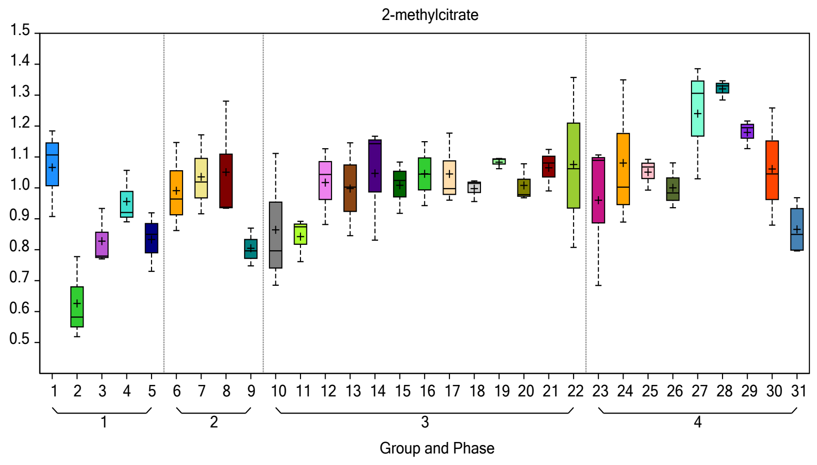
2-methylcitrate
1.5
1.4
1.3
1.2
1.1
1.0
0.9
0.8
0.7
0.6
0.5
1 2 3 4 5 6 7 8 9 10 11 12 13 14 15 16 17 18 19 20 21 22 23 24 25 26 27 28 29 30 31
1
2
3
4
Group and Phase

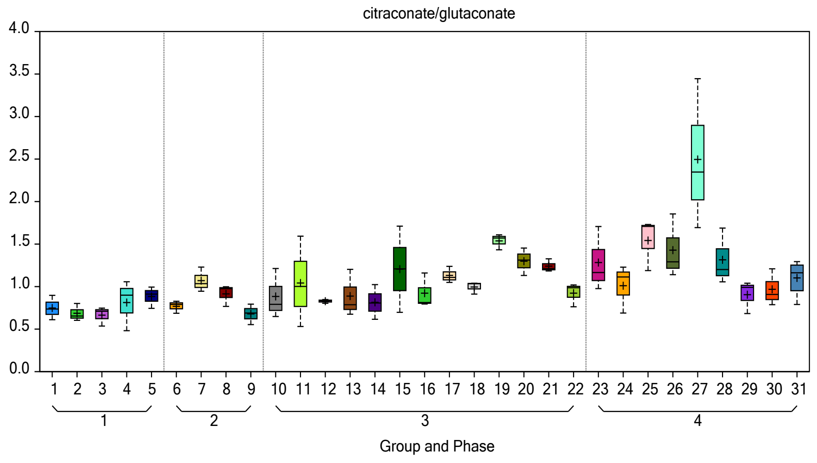
citraconate/glutaconate
4.0
3.5
3.0
2.5
2.0
1.5
1.0
0.5
0.0
1 2 3 4 5 6 7 8 9 10 11 12 13 14 15 16 17 18 19 20 21 22 23 24 25 26 27 28 29 30 31
1
2
3
4
Group and Phase

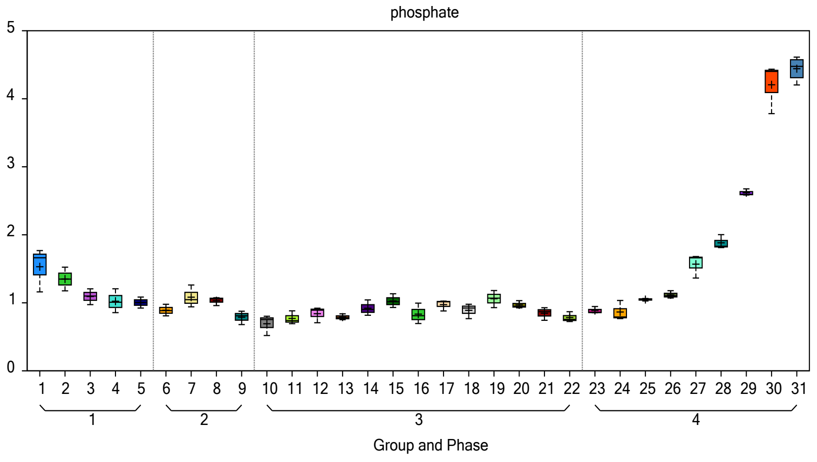
phosphate
5
4
3
2
1
0
1 2 3 4 5 6 7 8 9 10 11 12 13 14 15 16 17 18 19 20 21 22 23 24 25 26 27 28 29 30 31
1
2
3
4
Group and Phase

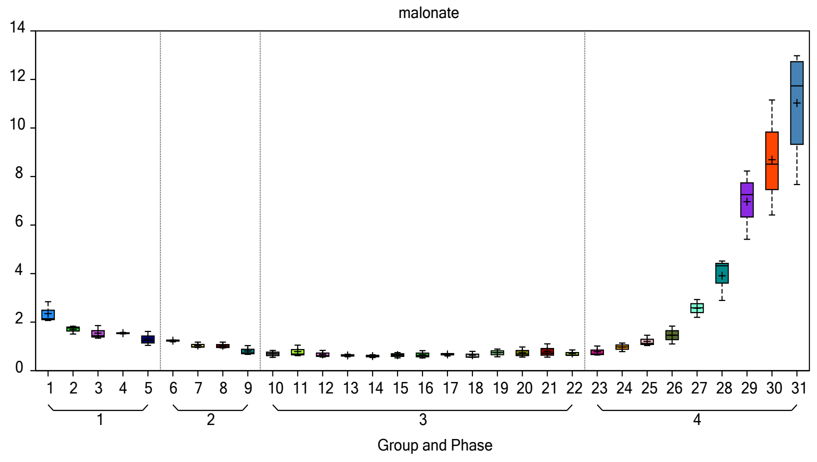
malonate
14
12
10
8
6
4
2
0
1 2 3 4 5 6 7 8 9 10 11 12 13 14 15 16 17 18 19 20 21 22 23 24 25 26 27 28 29 30 31
1
2
3
4
Group and Phase

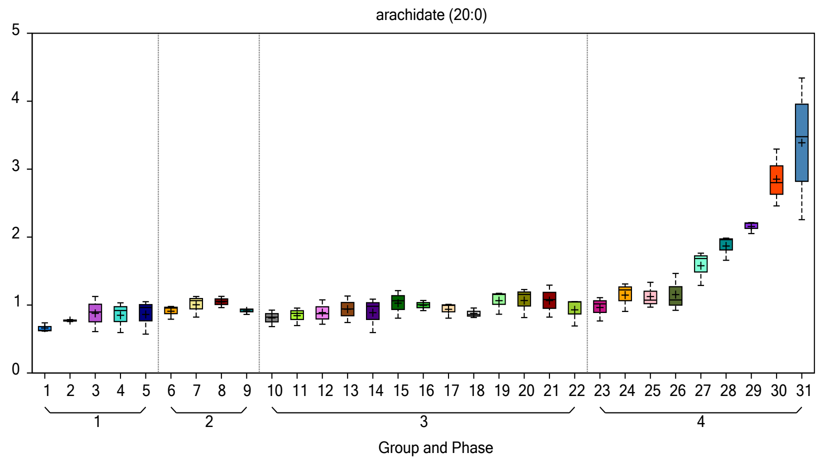
arachidate (20:0)
Group and Phase
1
2
3
4

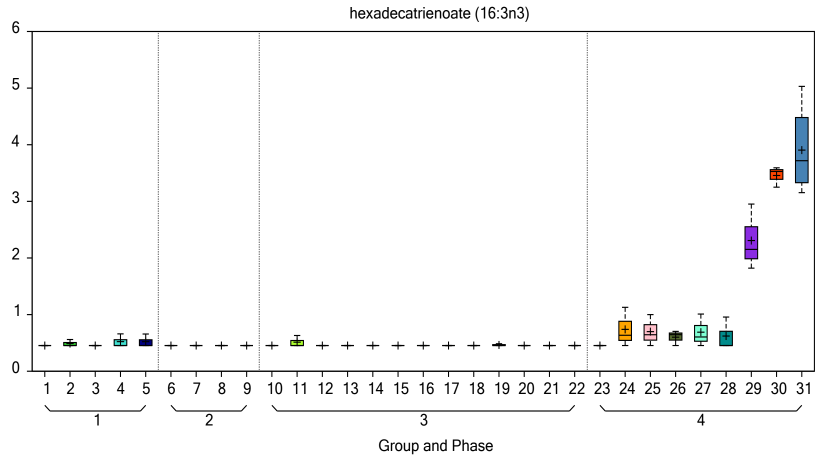
hexadecatrienoate (16:3n3)
Group and Phase
1
2
3
4

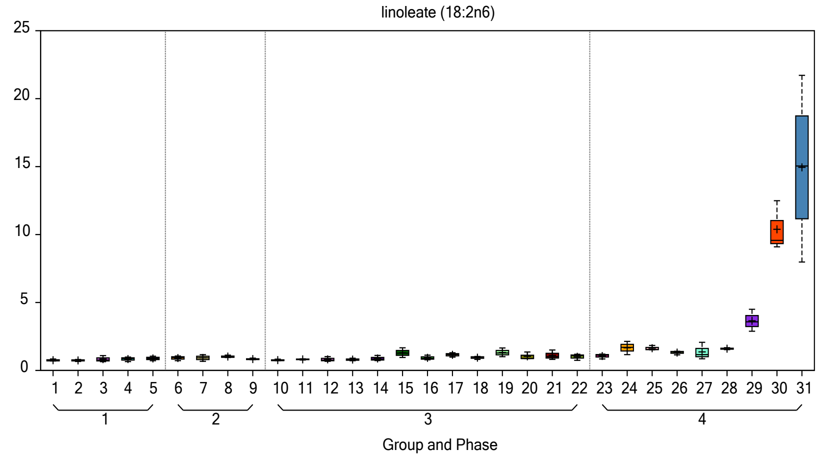
linoleate (18:2n6)
Group and Phase
1
2
3
4

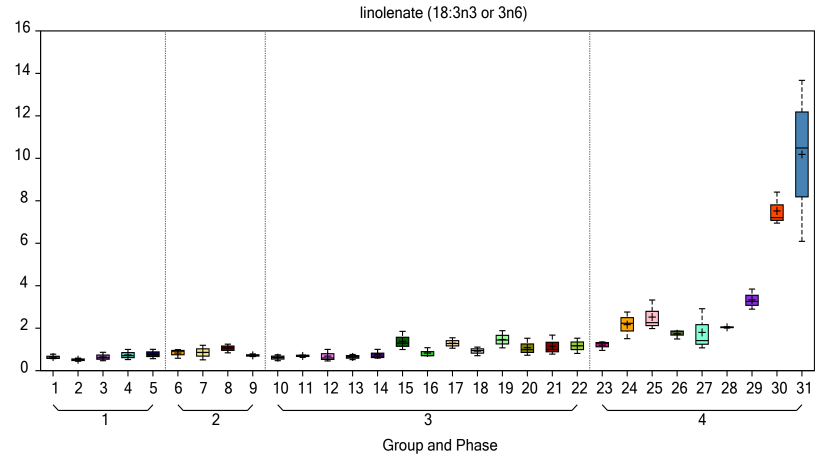
linolenate (18:3n3 or 3n6)
Group and Phase
1
2
3
4

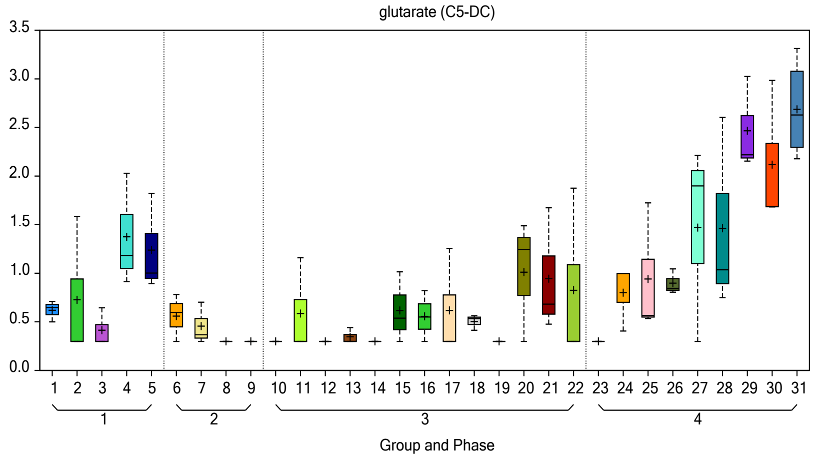
glutarate (C5-DC)
Group and Phase

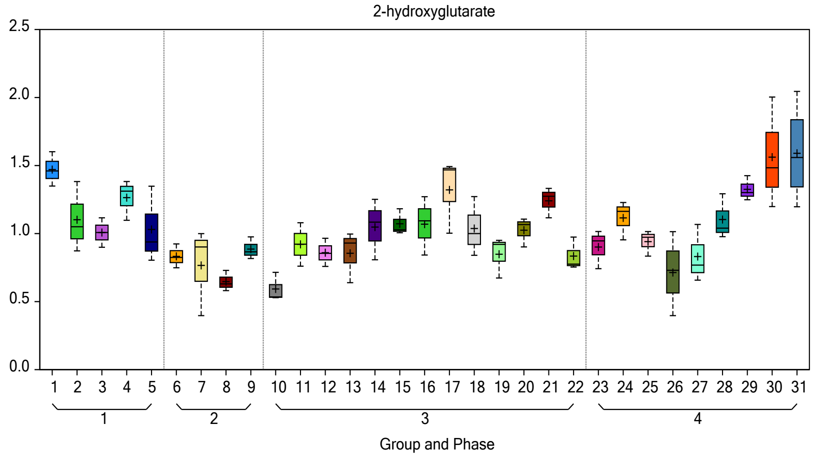
2-hydroxyglutarate
Group and Phase

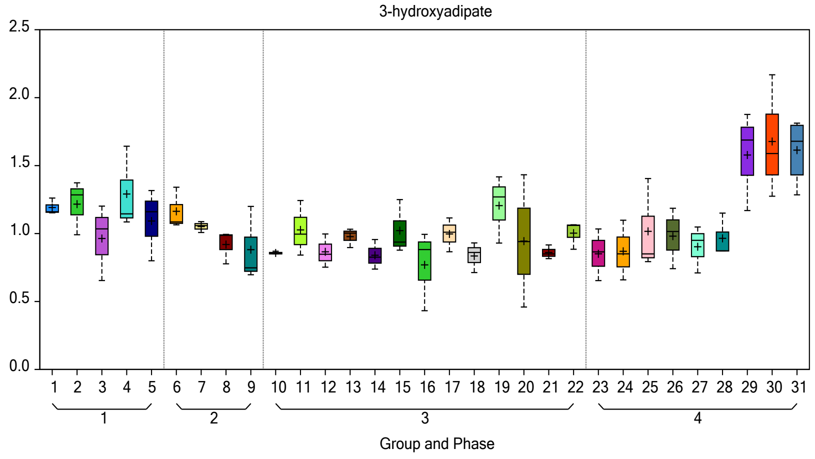
3-hydroxyadipate
Group and Phase

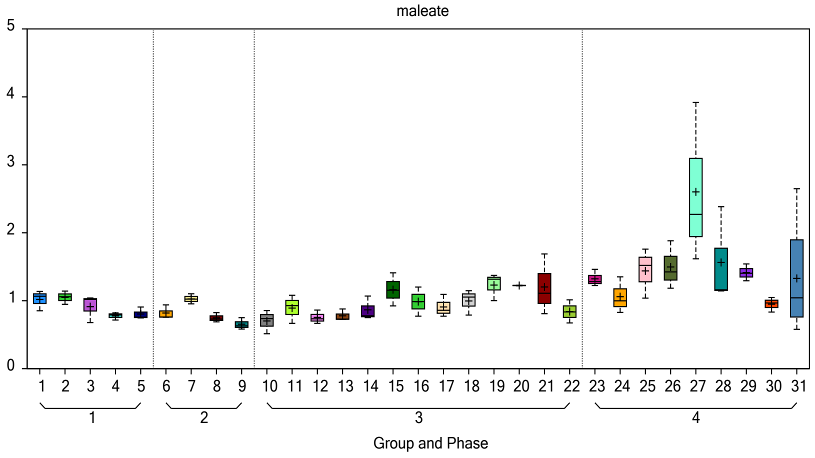
maleate
Group and Phase

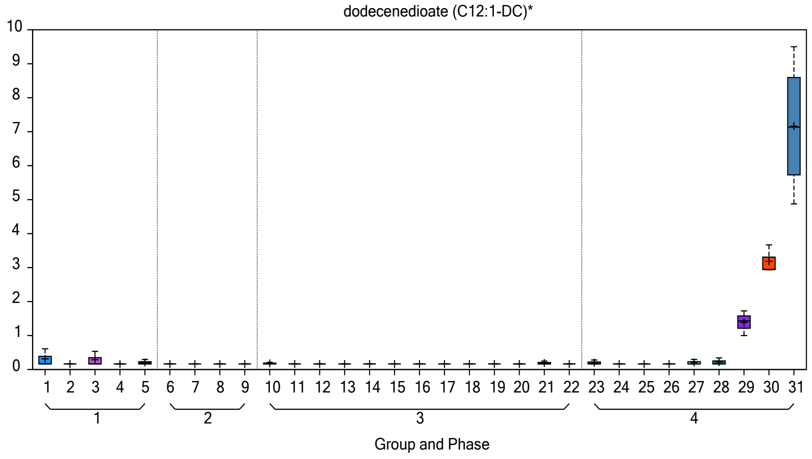
dodecenedioate (C12:1-DC)*
0
1
2
3
4
5
6
7
8
9
10
1 2 3 4 5 6 7 8 9 10 11 12 13 14 15 16 17 18 19 20 21 22 23 24 25 26 27 28 29 30 31
1
2
3
4
Group and Phase

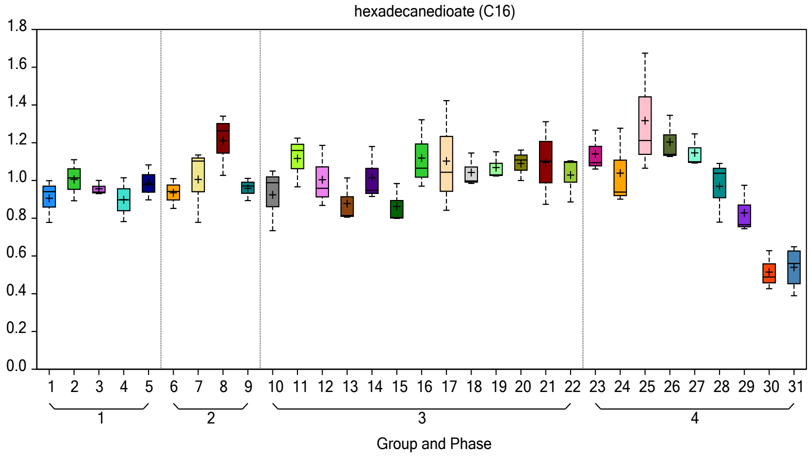
hexadecanedioate (C16)
0.0
0.2
0.4
0.6
0.8
1.0
1.2
1.4
1.6
1.8
1 2 3 4 5 6 7 8 9 10 11 12 13 14 15 16 17 18 19 20 21 22 23 24 25 26 27 28 29 30 31
1
2
3
4
Group and Phase

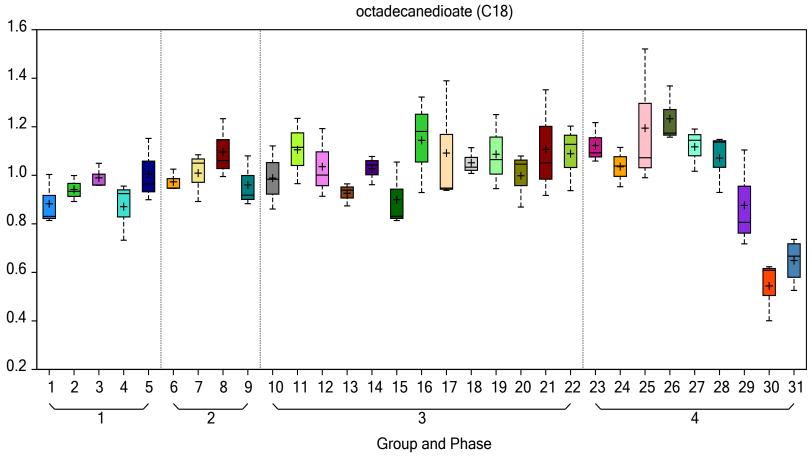
octadecanedioate (C18)
0.2
0.4
0.6
0.8
1.0
1.2
1.4
1.6
1 2 3 4 5 6 7 8 9 10 11 12 13 14 15 16 17 18 19 20 21 22 23 24 25 26 27 28 29 30 31
1
2
3
4
Group and Phase

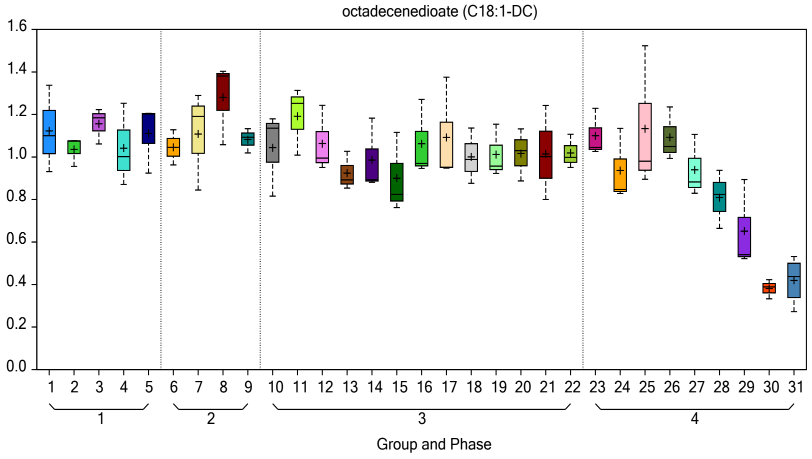
octadecenedioate (C18:1-DC)
0.0
0.2
0.4
0.6
0.8
1.0
1.2
1.4
1.6
1 2 3 4 5 6 7 8 9 10 11 12 13 14 15 16 17 18 19 20 21 22 23 24 25 26 27 28 29 30 31
1
2
3
4
Group and Phase

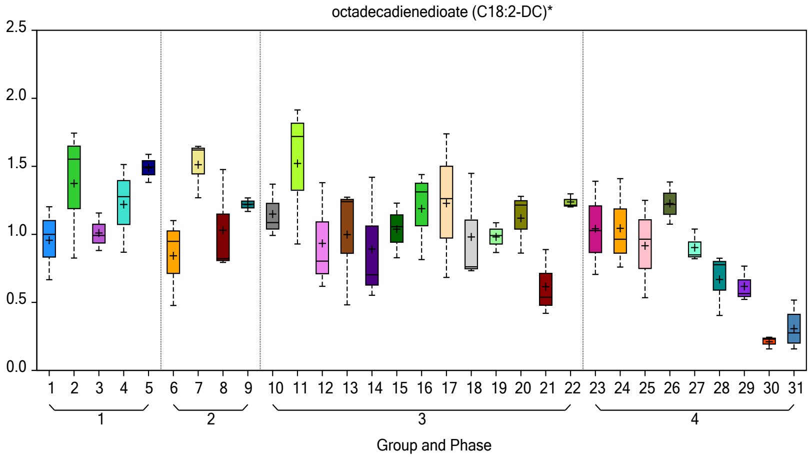
octadecadienedioate (C18:2-DC)*
0.0 0.5 1.0 1.5 2.0 2.5
1 2 3 4 5 6 7 8 9 10 11 12 13 14 15 16 17 18 19 20 21 22 23 24 25 26 27 28 29 30 31
1 2 3 4
Group and Phase

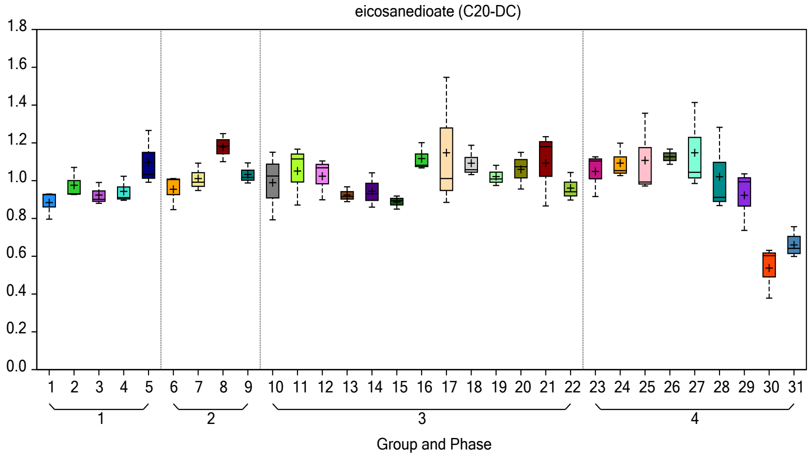
eicosanedioate (C20-DC)
0.0 0.2 0.4 0.6 0.8 1.0 1.2 1.4 1.6 1.8
1 2 3 4 5 6 7 8 9 10 11 12 13 14 15 16 17 18 19 20 21 22 23 24 25 26 27 28 29 30 31
1 2 3 4
Group and Phase

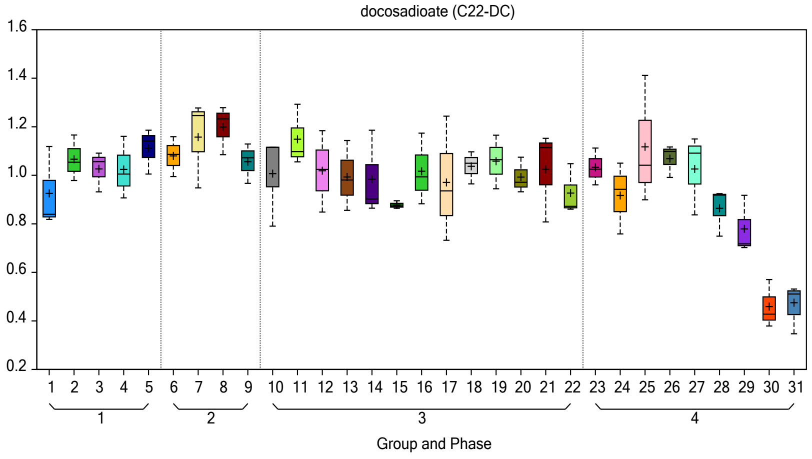
docosadioate (C22-DC)
0.2 0.4 0.6 0.8 1.0 1.2 1.4 1.6
1 2 3 4 5 6 7 8 9 10 11 12 13 14 15 16 17 18 19 20 21 22 23 24 25 26 27 28 29 30 31
1 2 3 4
Group and Phase

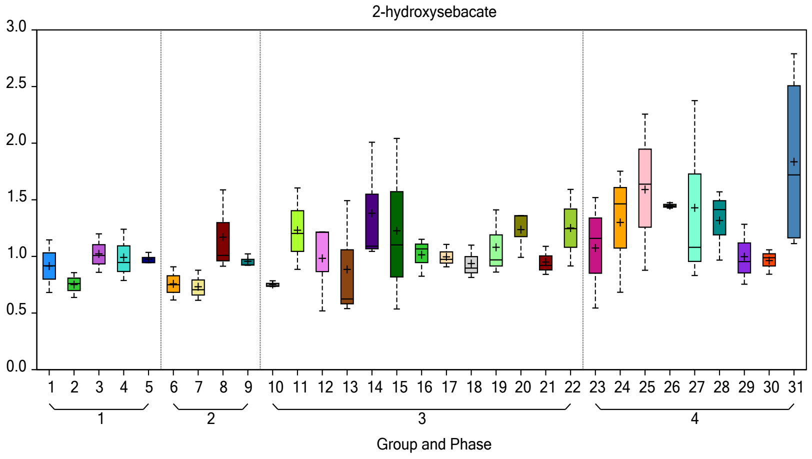
2-hydroxysebacate
0.0 0.5 1.0 1.5 2.0 2.5 3.0
1 2 3 4 5 6 7 8 9 10 11 12 13 14 15 16 17 18 19 20 21 22 23 24 25 26 27 28 29 30 31
1 2 3 4
Group and Phase

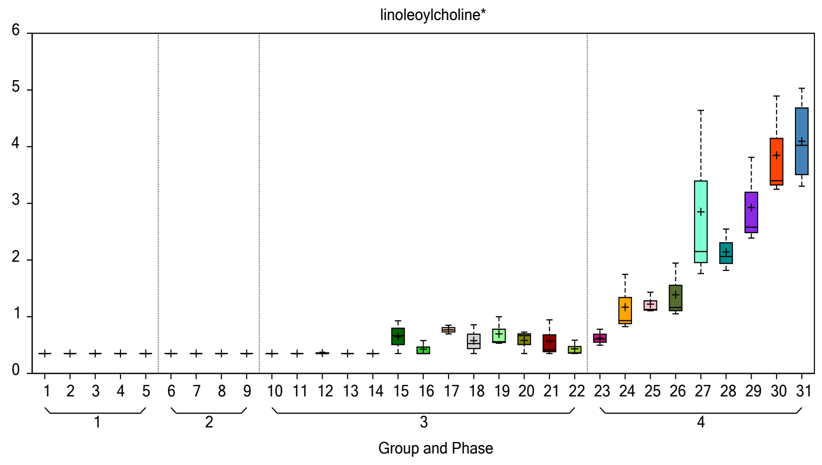
linoleoylcholine*
0
1
2
3
4
5
6
1 2 3 4 5 6 7 8 9 10 11 12 13 14 15 16 17 18 19 20 21 22 23 24 25 26 27 28 29 30 31
1
2
3
4
Group and Phase

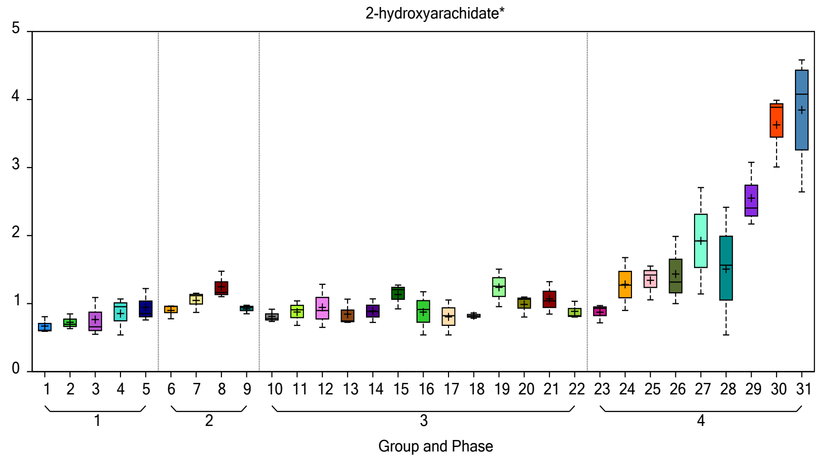
2-hydroxyarachidate*
0
1
2
3
4
5
1 2 3 4 5 6 7 8 9 10 11 12 13 14 15 16 17 18 19 20 21 22 23 24 25 26 27 28 29 30 31
1
2
3
4
Group and Phase

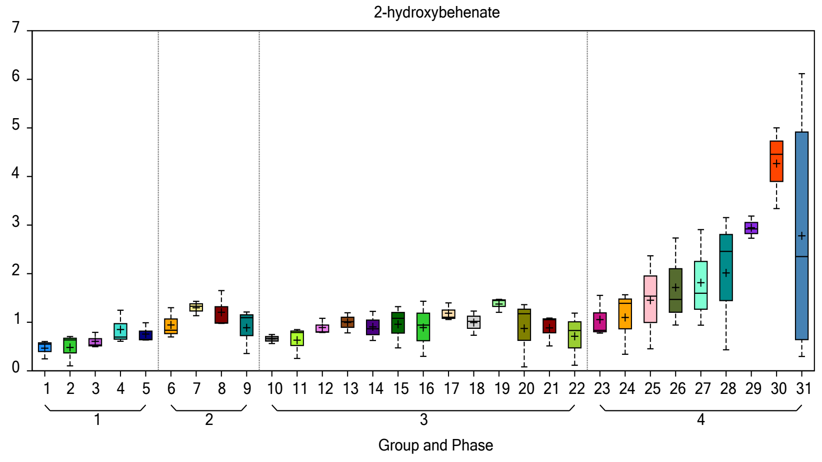
2-hydroxybehenate
0
1
2
3
4
5
6
7
1 2 3 4 5 6 7 8 9 10 11 12 13 14 15 16 17 18 19 20 21 22 23 24 25 26 27 28 29 30 31
1
2
3
4
Group and Phase

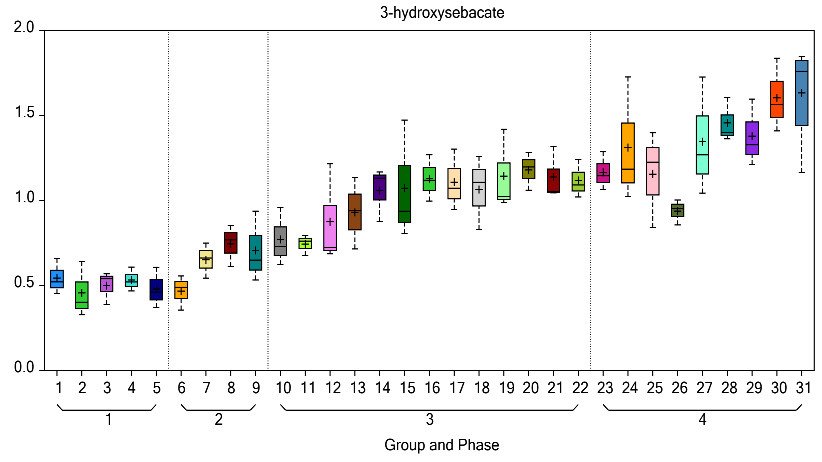
3-hydroxysebacate
0.0
0.5
1.0
1.5
2.0
1 2 3 4 5 6 7 8 9 10 11 12 13 14 15 16 17 18 19 20 21 22 23 24 25 26 27 28 29 30 31
1
2
3
4
Group and Phase

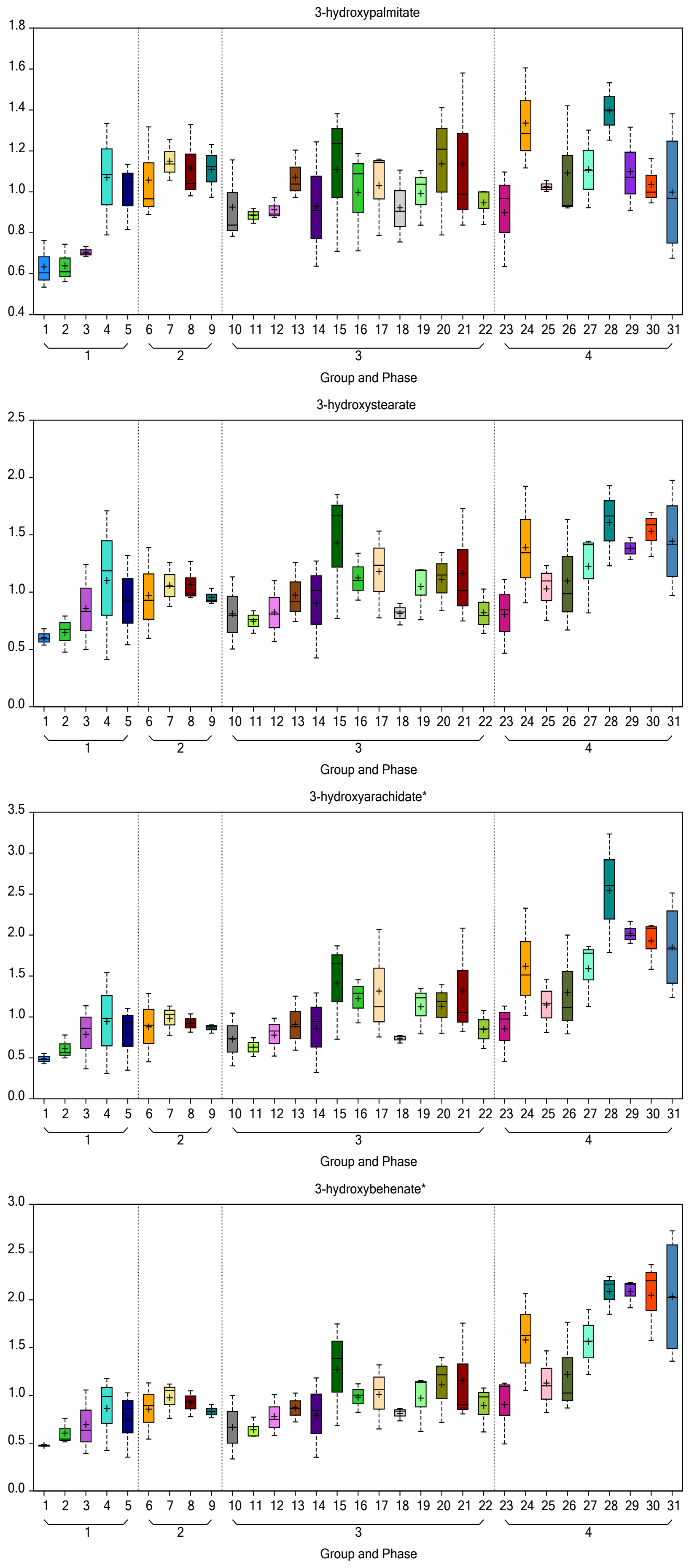
3-hydroxypalmitate
1.8
1.6
1.4
1.2
1.0
0.8
0.6
0.4
1 2 3 4 5 6 7 8 9 10 11 12 13 14 15 16 17 18 19 20 21 22 23 24 25 26 27 28 29 30 31
1 2 3 4
Group and Phase
3-hydroxystearate
2.5
2.0
1.5
1.0
0.5
0.0
1 2 3 4 5 6 7 8 9 10 11 12 13 14 15 16 17 18 19 20 21 22 23 24 25 26 27 28 29 30 31
1 2 3 4
Group and Phase
3-hydroxyarachidate*
3.5
3.0
2.5
2.0
1.5
1.0
0.5
0.0
1 2 3 4 5 6 7 8 9 10 11 12 13 14 15 16 17 18 19 20 21 22 23 24 25 26 27 28 29 30 31
1 2 3 4
Group and Phase
3-hydroxybehenate*
3.0
2.5
2.0
1.5
1.0
0.5
0.0
1 2 3 4 5 6 7 8 9 10 11 12 13 14 15 16 17 18 19 20 21 22 23 24 25 26 27 28 29 30 31
1 2 3 4
Group and Phase

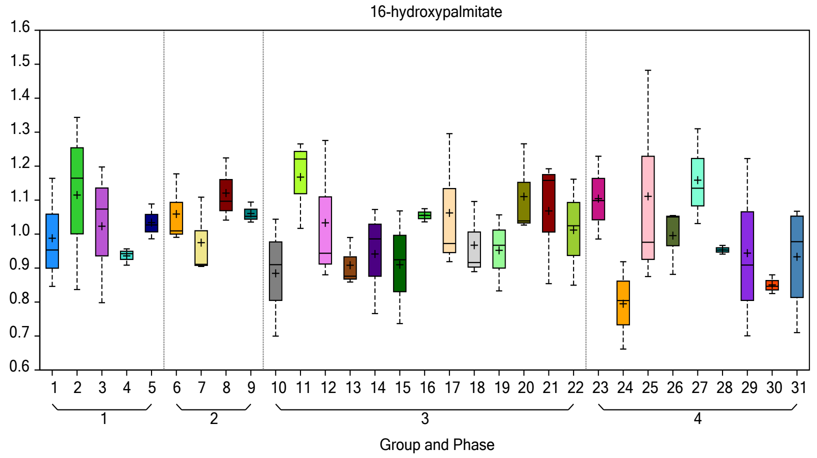
16-hydroxypalmitate
1.6
1.5
1.4
1.3
1.2
1.1
1.0
0.9
0.8
0.7
0.6
1 2 3 4 5 6 7 8 9 10 11 12 13 14 15 16 17 18 19 20 21 22 23 24 25 26 27 28 29 30 31
1
2
3
4
Group and Phase

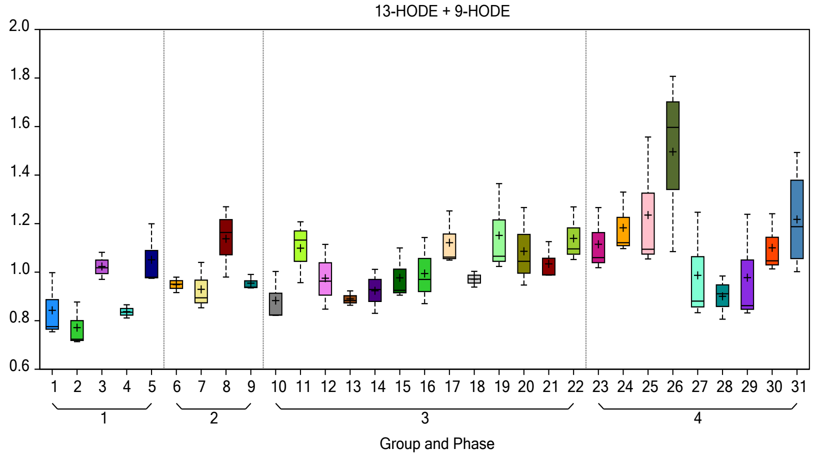
13-HODE + 9-HODE
2.0
1.8
1.6
1.4
1.2
1.0
0.8
0.6
1 2 3 4 5 6 7 8 9 10 11 12 13 14 15 16 17 18 19 20 21 22 23 24 25 26 27 28 29 30 31
1
2
3
4
Group and Phase

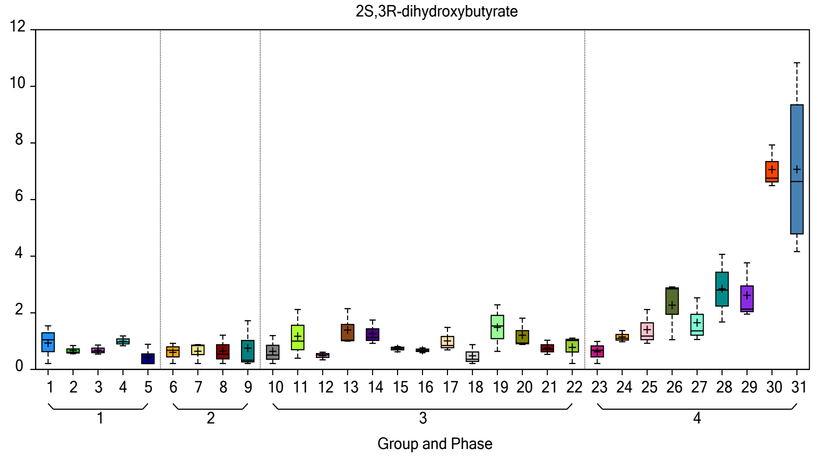
2S,3R-dihydroxybutyrate
12
10
8
6
4
2
0
1 2 3 4 5 6 7 8 9 10 11 12 13 14 15 16 17 18 19 20 21 22 23 24 25 26 27 28 29 30 31
1
2
3
4
Group and Phase

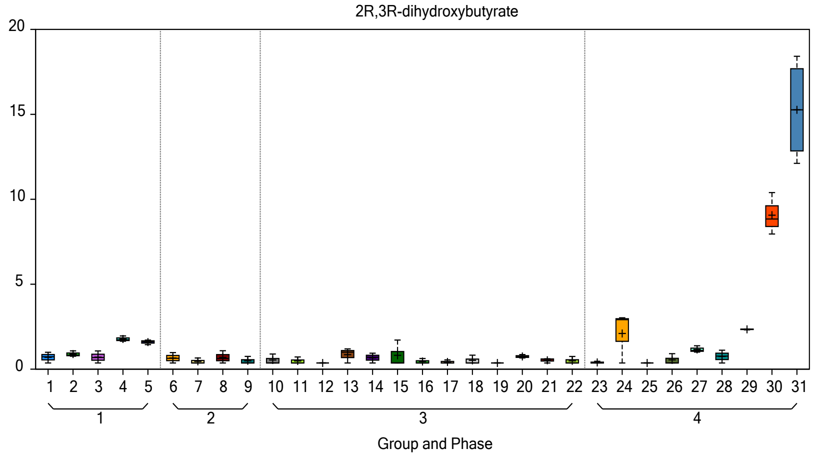
2R,3R-dihydroxybutyrate
20
15
10
5
0
1 2 3 4 5 6 7 8 9 10 11 12 13 14 15 16 17 18 19 20 21 22 23 24 25 26 27 28 29 30 31
1
2
3
4
Group and Phase

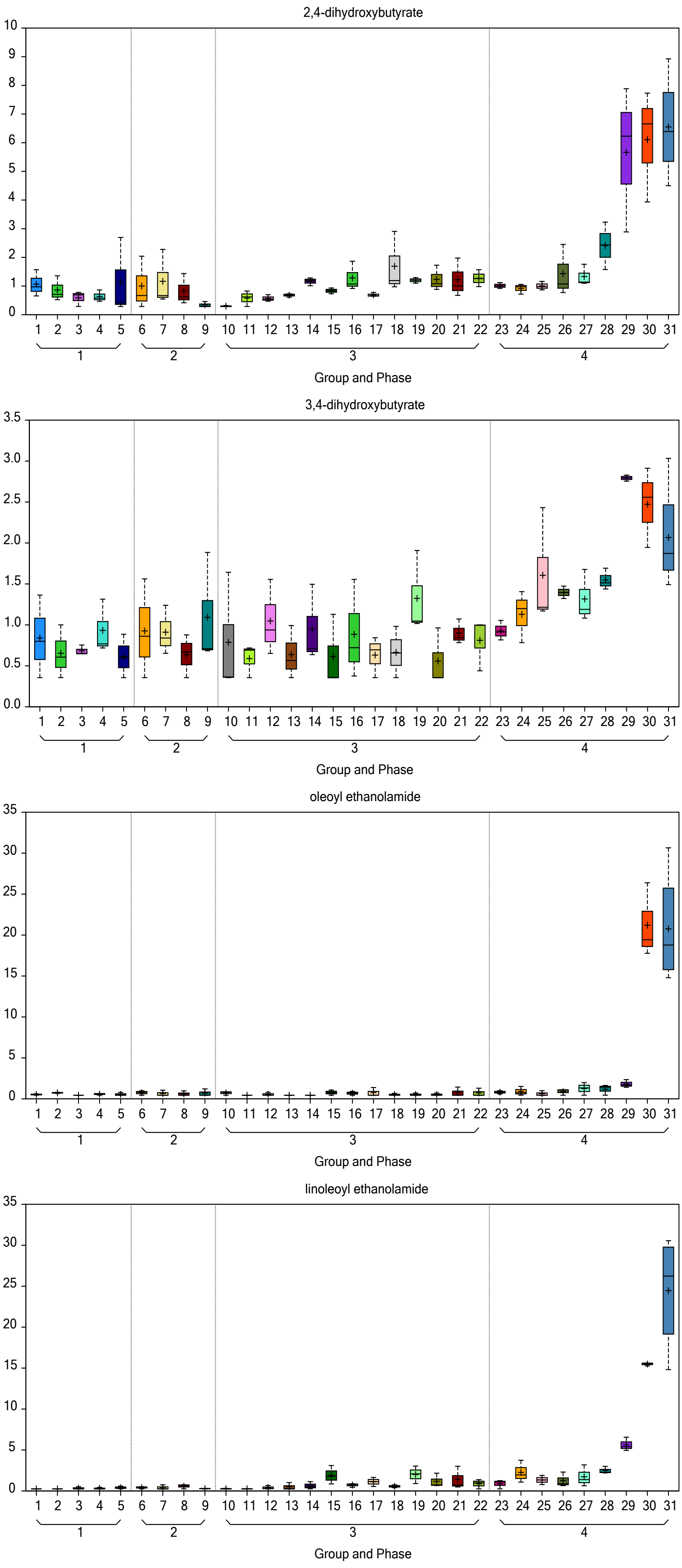
2,4-dihydroxybutyrate
3,4-dihydroxybutyrate
oleoyl ethanolamide
linoleoyl ethanolamide
Group and Phase

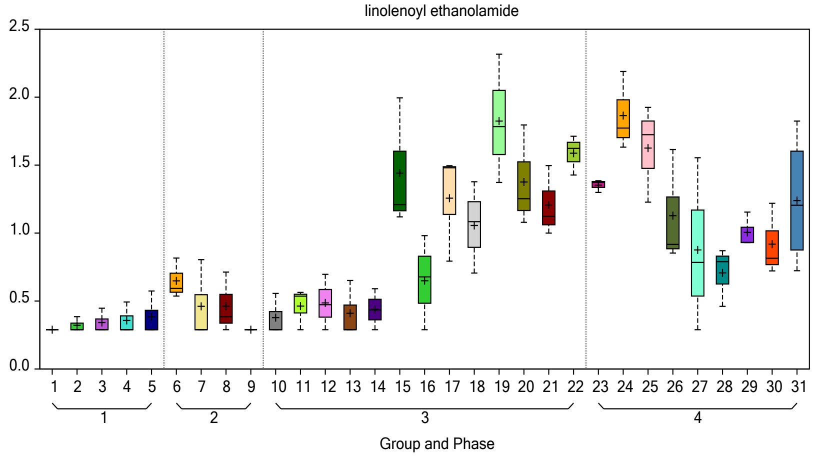
linolenoyl ethanolamide
2.5
2.0
1.5
1.0
0.5
0.0
1 2 3 4 5 6 7 8 9 10 11 12 13 14 15 16 17 18 19 20 21 22 23 24 25 26 27 28 29 30 31
1
2
3
4
Group and Phase

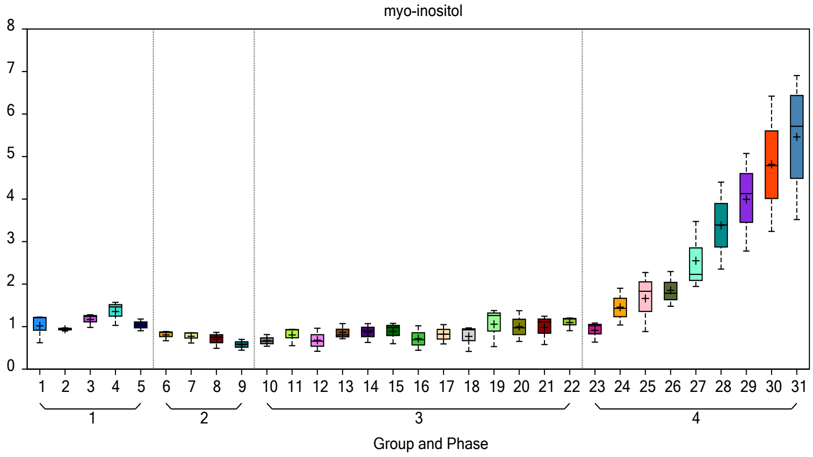
myo-inositol
8
7
6
5
4
3
2
1
0
1 2 3 4 5 6 7 8 9 10 11 12 13 14 15 16 17 18 19 20 21 22 23 24 25 26 27 28 29 30 31
1
2
3
4
Group and Phase

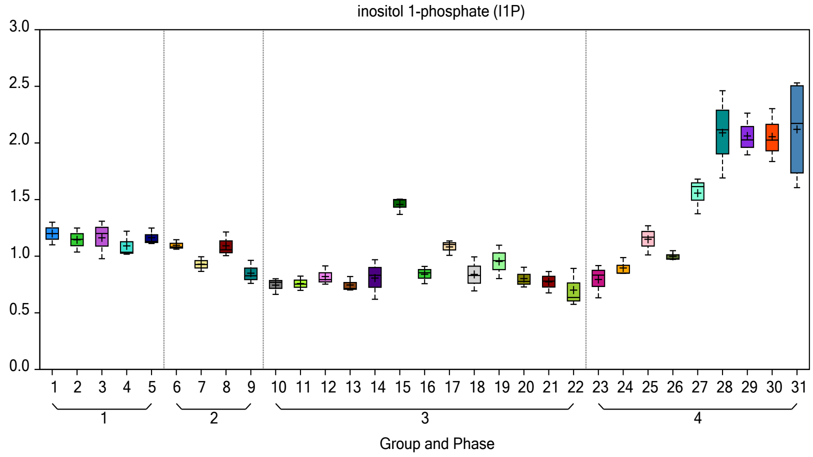
inositol 1-phosphate (I1P)
3.0
2.5
2.0
1.5
1.0
0.5
0.0
1 2 3 4 5 6 7 8 9 10 11 12 13 14 15 16 17 18 19 20 21 22 23 24 25 26 27 28 29 30 31
1
2
3
4
Group and Phase

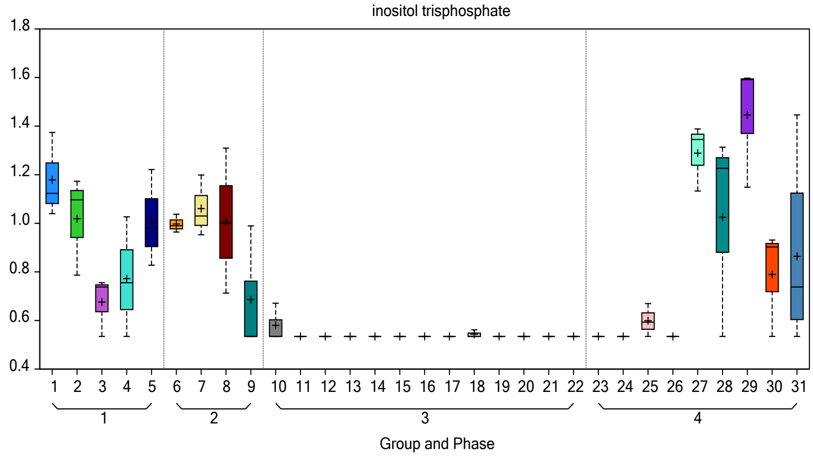
inositol trisphosphate
1.8
1.6
1.4
1.2
1.0
0.8
0.6
0.4
1 2 3 4 5 6 7 8 9 10 11 12 13 14 15 16 17 18 19 20 21 22 23 24 25 26 27 28 29 30 31
1
2
3
4
Group and Phase

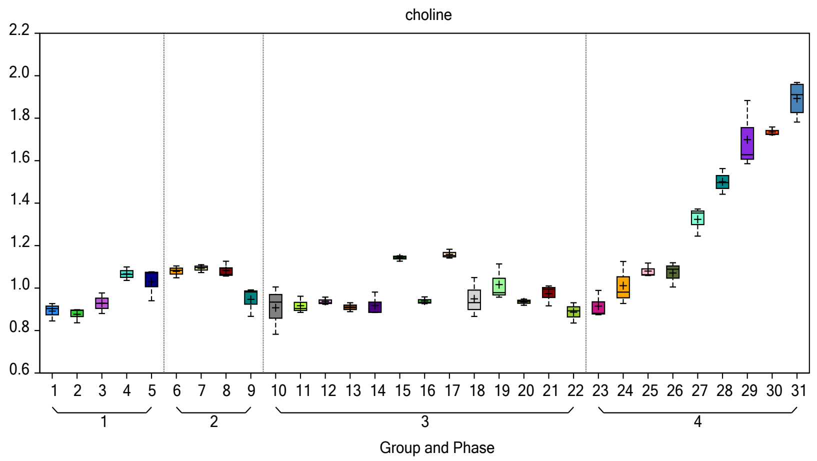
choline
2.2
2.0
1.8
1.6
1.4
1.2
1.0
0.8
0.6
1 2 3 4 5 6 7 8 9 10 11 12 13 14 15 16 17 18 19 20 21 22 23 24 25 26 27 28 29 30 31
1
2
3
4
Group and Phase

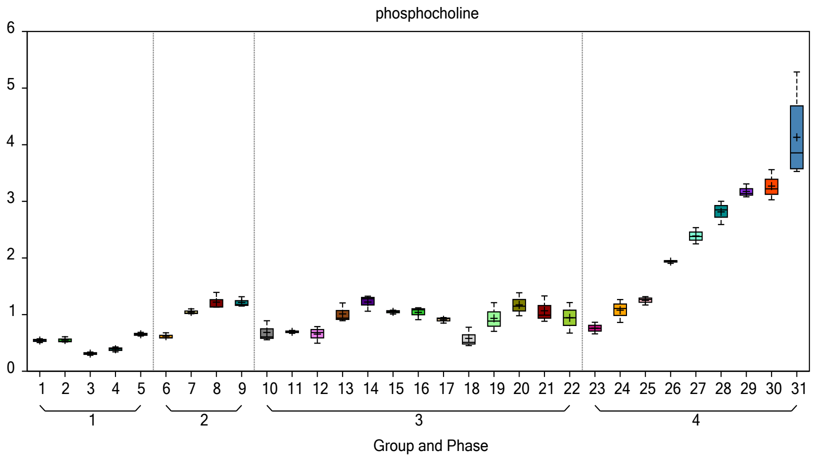
phosphocholine
6
5
4
3
2
1
0
1 2 3 4 5 6 7 8 9 10 11 12 13 14 15 16 17 18 19 20 21 22 23 24 25 26 27 28 29 30 31
1
2
3
4
Group and Phase

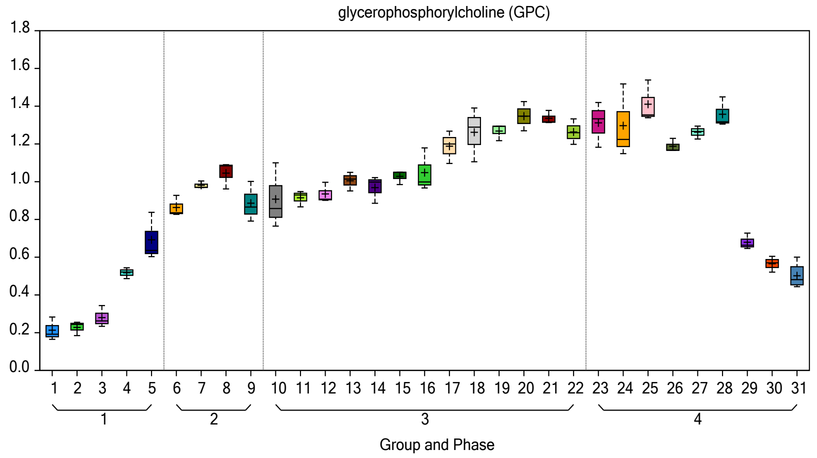
glycerophosphorylcholine (GPC)
1.8
1.6
1.4
1.2
1.0
0.8
0.6
0.4
0.2
0.0
1 2 3 4 5 6 7 8 9 10 11 12 13 14 15 16 17 18 19 20 21 22 23 24 25 26 27 28 29 30 31
1
2
3
4
Group and Phase

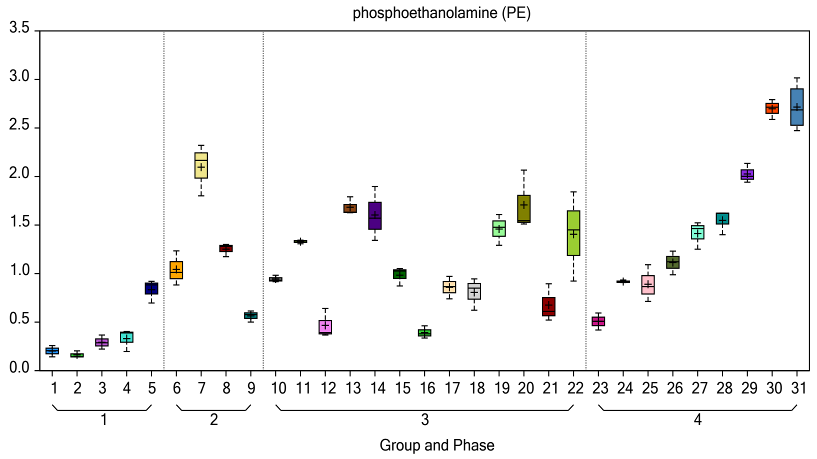
phosphoethanolamine (PE)
3.5
3.0
2.5
2.0
1.5
1.0
0.5
0.0
1 2 3 4 5 6 7 8 9 10 11 12 13 14 15 16 17 18 19 20 21 22 23 24 25 26 27 28 29 30 31
1
2
3
4
Group and Phase

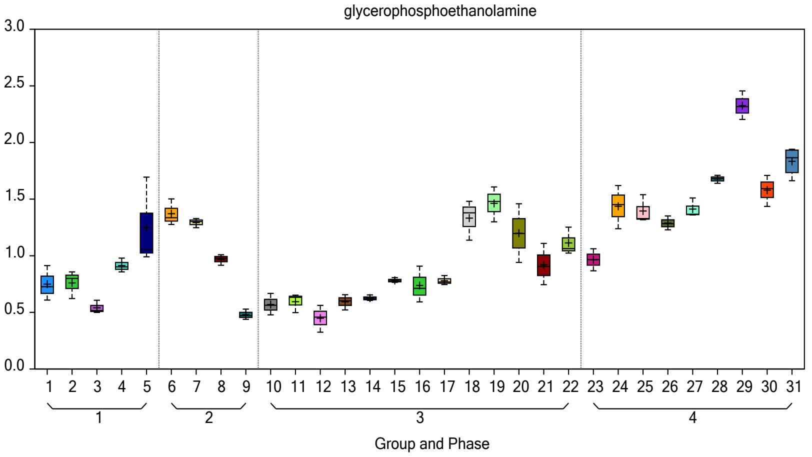
glycerophosphoethanolamine
3.0
2.5
2.0
1.5
1.0
0.5
0.0
1 2 3 4 5 6 7 8 9 10 11 12 13 14 15 16 17 18 19 20 21 22 23 24 25 26 27 28 29 30 31
1
2
3
4
Group and Phase

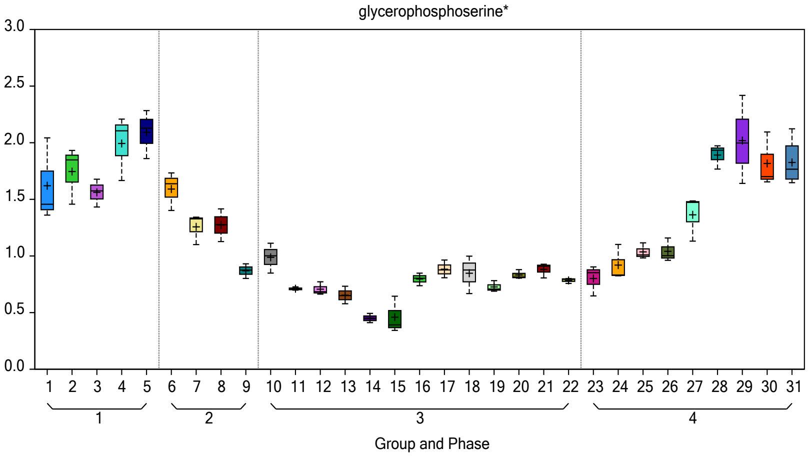
glycerophosphoserine*
3.0
2.5
2.0
1.5
1.0
0.5
0.0
1 2 3 4 5 6 7 8 9 10 11 12 13 14 15 16 17 18 19 20 21 22 23 24 25 26 27 28 29 30 31
1
2
3
4
Group and Phase

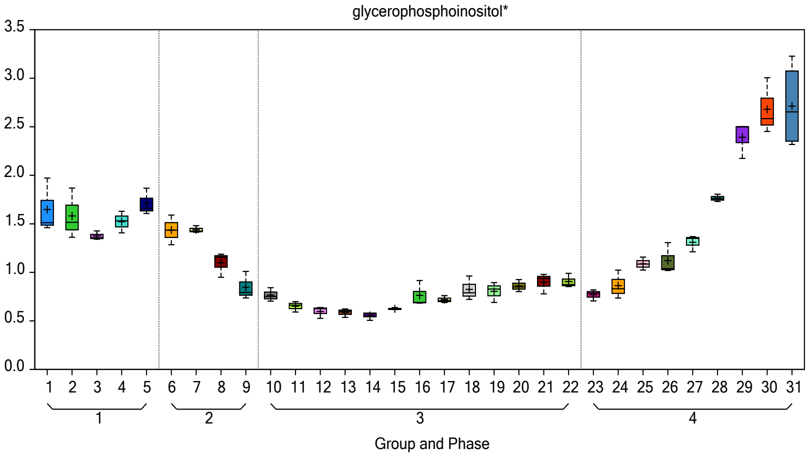
glycerophosphoinositol*
3.5
3.0
2.5
2.0
1.5
1.0
0.5
0.0
1 2 3 4 5 6 7 8 9 10 11 12 13 14 15 16 17 18 19 20 21 22 23 24 25 26 27 28 29 30 31
1
2
3
4
Group and Phase

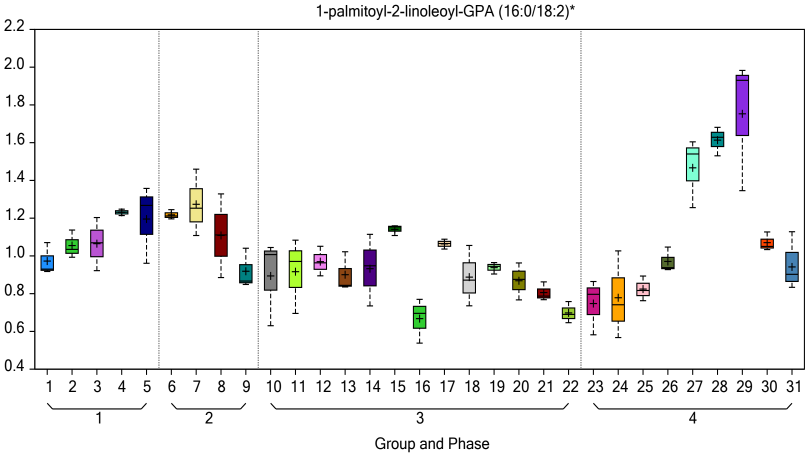
1-palmitoyl-2-linoleoyl-GPA (16:0/18:2)*
2.2
2.0
1.8
1.6
1.4
1.2
1.0
0.8
0.6
0.4
1 2 3 4 5 6 7 8 9 10 11 12 13 14 15 16 17 18 19 20 21 22 23 24 25 26 27 28 29 30 31
1
2
3
4
Group and Phase

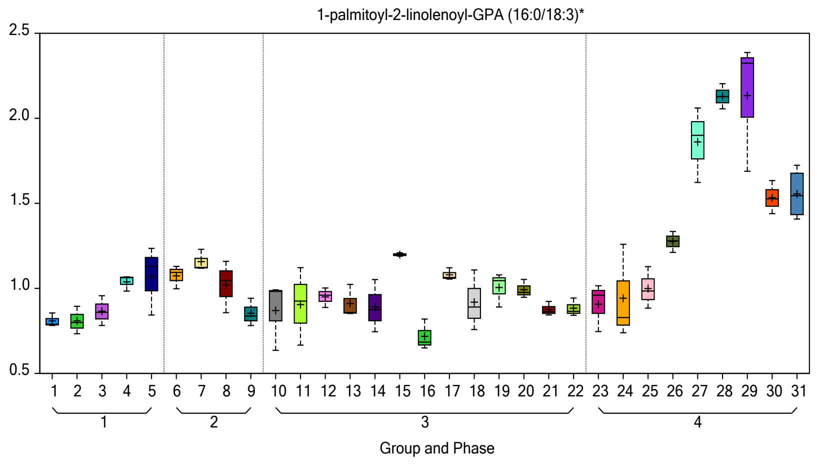
1-palmitoyl-2-linolenoyl-GPA (16:0/18:3)*
2.5
2.0
1.5
1.0
0.5
1 2 3 4 5 6 7 8 9 10 11 12 13 14 15 16 17 18 19 20 21 22 23 24 25 26 27 28 29 30 31
1 2 3 4
Group and Phase

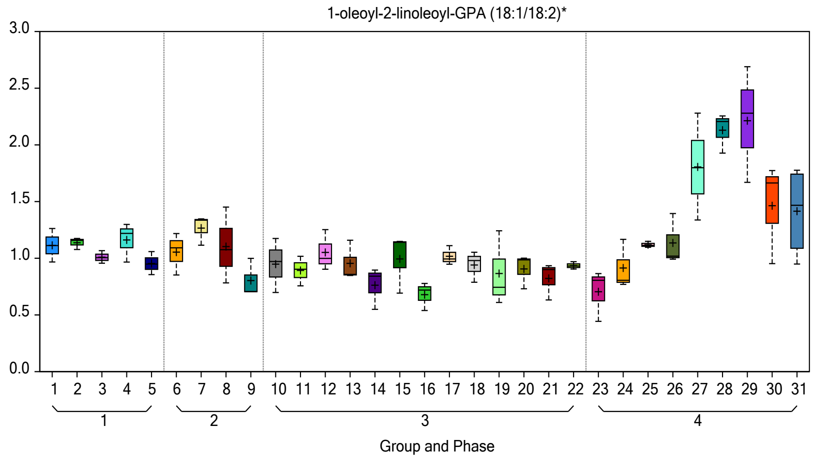
1-oleoyl-2-linoleoyl-GPA (18:1/18:2)*
3.0
2.5
2.0
1.5
1.0
0.5
0.0
1 2 3 4 5 6 7 8 9 10 11 12 13 14 15 16 17 18 19 20 21 22 23 24 25 26 27 28 29 30 31
1 2 3 4
Group and Phase

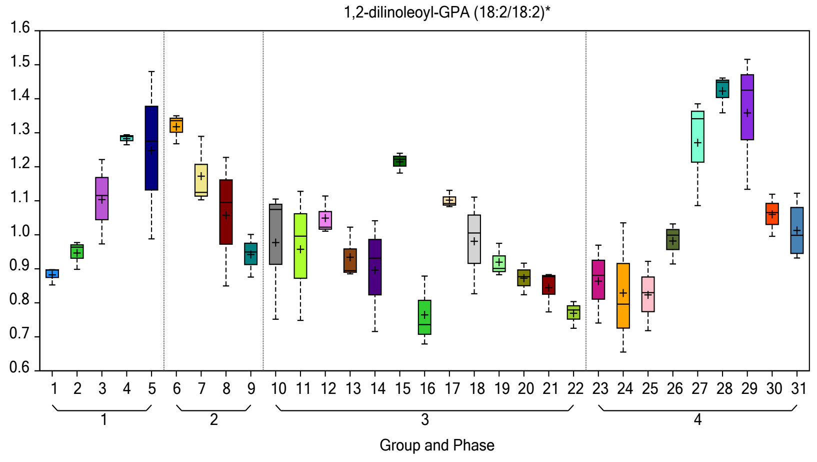
1,2-dilinoleoyl-GPA (18:2/18:2)*
1.6
1.5
1.4
1.3
1.2
1.1
1.0
0.9
0.8
0.7
0.6
1 2 3 4 5 6 7 8 9 10 11 12 13 14 15 16 17 18 19 20 21 22 23 24 25 26 27 28 29 30 31
1 2 3 4
Group and Phase

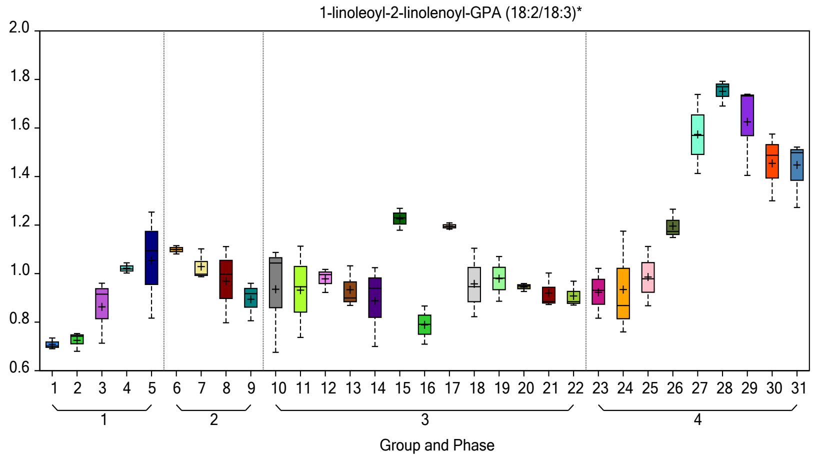
1-linoleoyl-2-linolenoyl-GPA (18:2/18:3)*
2.0
1.8
1.6
1.4
1.2
1.0
0.8
0.6
1 2 3 4 5 6 7 8 9 10 11 12 13 14 15 16 17 18 19 20 21 22 23 24 25 26 27 28 29 30 31
1 2 3 4
Group and Phase

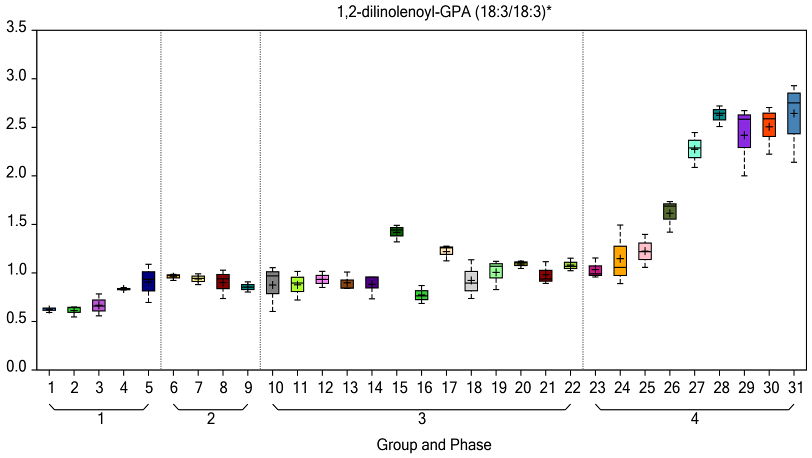
1,2-dilinolenoyl-GPA (18:3/18:3)*
0.0
0.5
1.0
1.5
2.0
2.5
3.0
3.5
1 2 3 4 5 6 7 8 9 10 11 12 13 14 15 16 17 18 19 20 21 22 23 24 25 26 27 28 29 30 31
1
2
3
4
Group and Phase

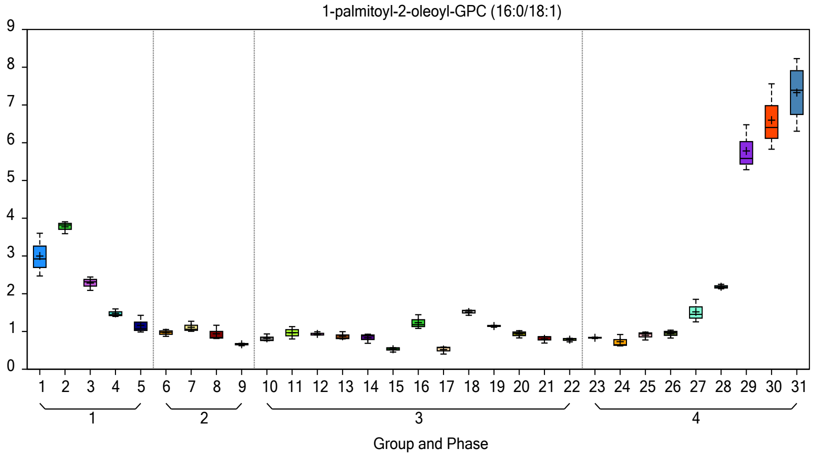
1-palmitoyl-2-oleoyl-GPC (16:0/18:1)
0
1
2
3
4
5
6
7
8
9
1 2 3 4 5 6 7 8 9 10 11 12 13 14 15 16 17 18 19 20 21 22 23 24 25 26 27 28 29 30 31
1
2
3
4
Group and Phase

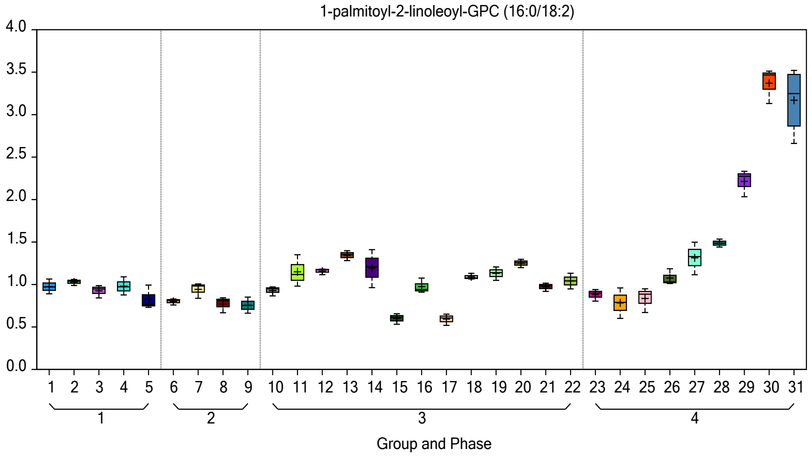
1-palmitoyl-2-linoleoyl-GPC (16:0/18:2)
0.0
0.5
1.0
1.5
2.0
2.5
3.0
3.5
4.0
1 2 3 4 5 6 7 8 9 10 11 12 13 14 15 16 17 18 19 20 21 22 23 24 25 26 27 28 29 30 31
1
2
3
4
Group and Phase

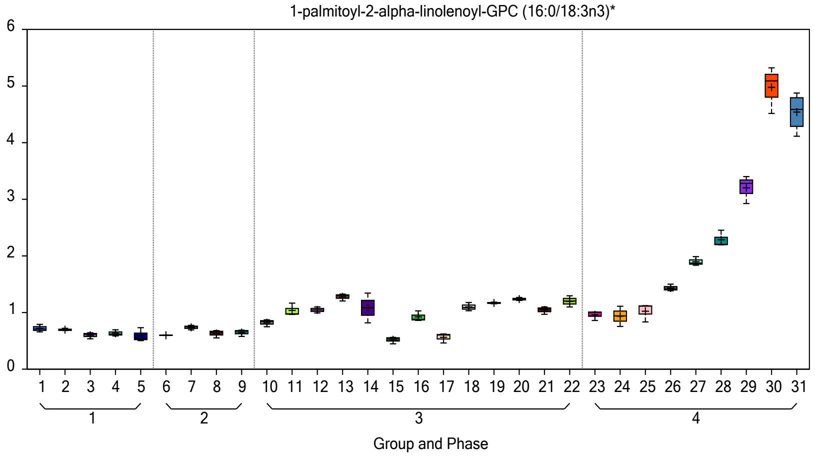
1-palmitoyl-2-alpha-linolenoyl-GPC (16:0/18:3n3)*
0
1
2
3
4
5
6
1 2 3 4 5 6 7 8 9 10 11 12 13 14 15 16 17 18 19 20 21 22 23 24 25 26 27 28 29 30 31
1
2
3
4
Group and Phase

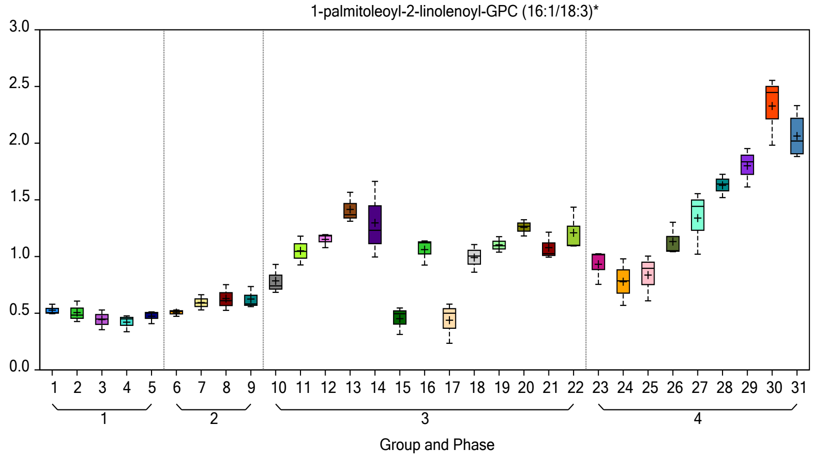
1-palmitoleoyl-2-linolenoyl-GPC (16:1/18:3)*
3.0
2.5
2.0
1.5
1.0
0.5
0.0
1 2 3 4 5 6 7 8 9 10 11 12 13 14 15 16 17 18 19 20 21 22 23 24 25 26 27 28 29 30 31
1
2
3
4
Group and Phase

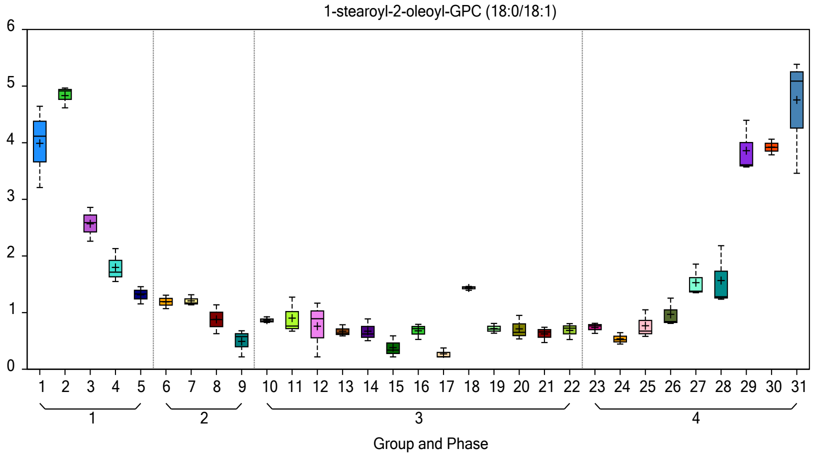
1-stearoyl-2-oleoyl-GPC (18:0/18:1)
6
5
4
3
2
1
0
1 2 3 4 5 6 7 8 9 10 11 12 13 14 15 16 17 18 19 20 21 22 23 24 25 26 27 28 29 30 31
1
2
3
4
Group and Phase

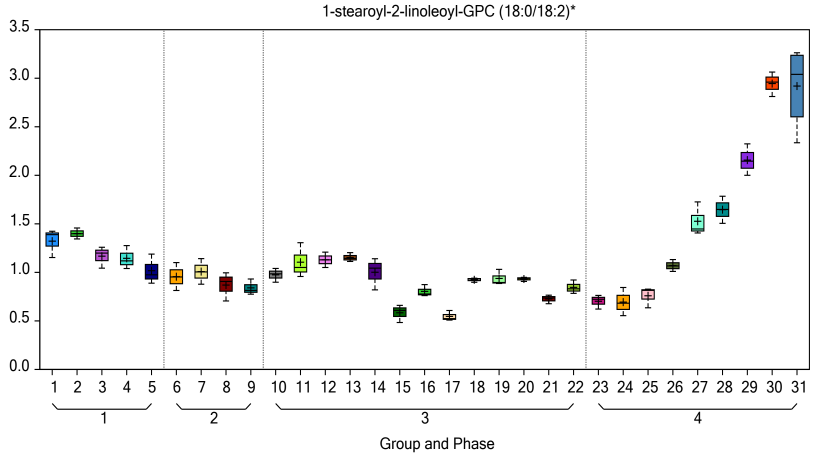
1-stearoyl-2-linoleoyl-GPC (18:0/18:2)*
3.5
3.0
2.5
2.0
1.5
1.0
0.5
0.0
1 2 3 4 5 6 7 8 9 10 11 12 13 14 15 16 17 18 19 20 21 22 23 24 25 26 27 28 29 30 31
1
2
3
4
Group and Phase

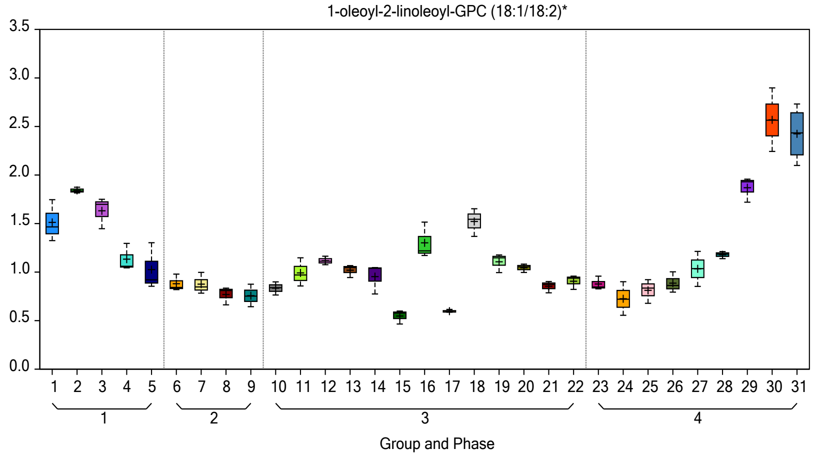
1-oleoyl-2-linoleoyl-GPC (18:1/18:2)*
3.5
3.0
2.5
2.0
1.5
1.0
0.5
0.0
1 2 3 4 5 6 7 8 9 10 11 12 13 14 15 16 17 18 19 20 21 22 23 24 25 26 27 28 29 30 31
1
2
3
4
Group and Phase

1,2-dilinoleoyl-GPC (18:2/18:2)

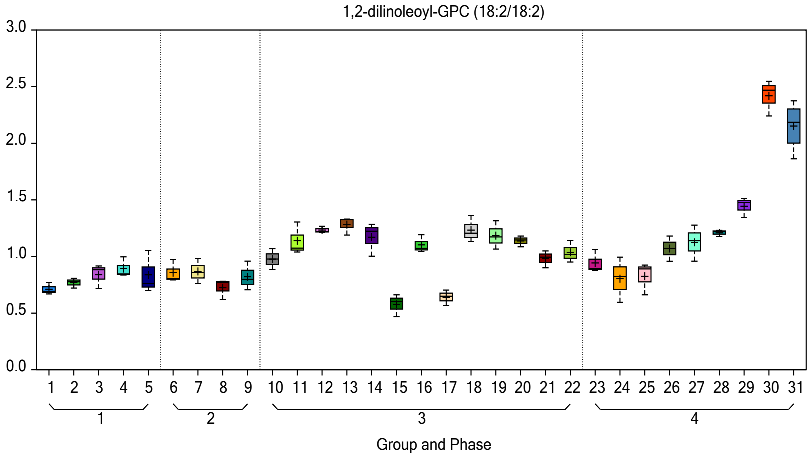

1-linoleoyl-2-linolenoyl-GPC (18:2/18:3)*

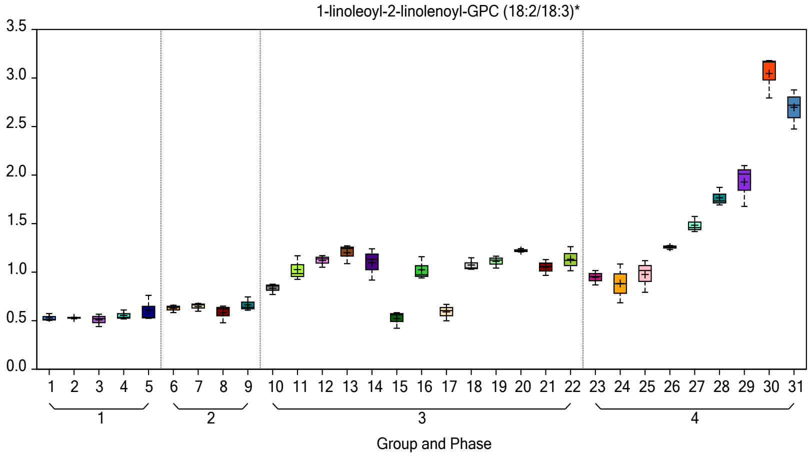

1,2-dilinolenoyl-GPC (18:3/18:3)*

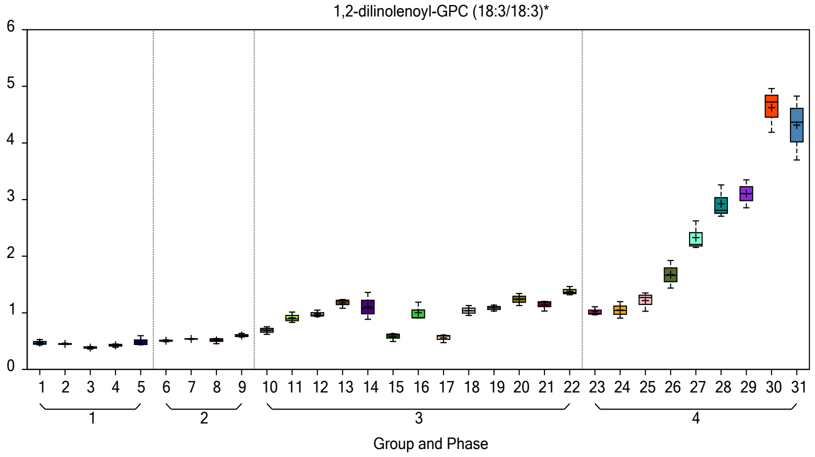

1-palmitoyl-2-oleoyl-GPE (16:0/18:1)

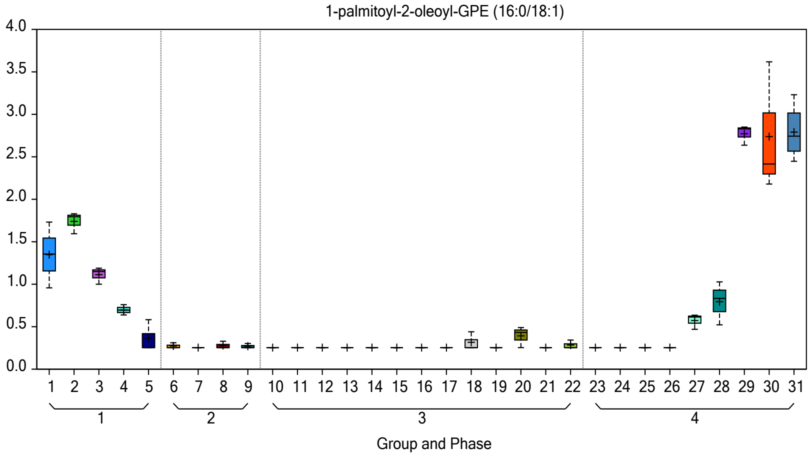

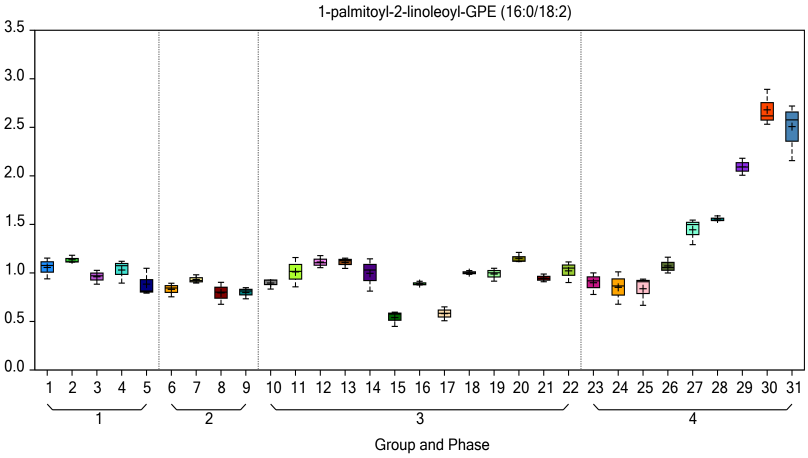
1-palmitoyl-2-linoleoyl-GPE (16:0/18:2)
3.5
3.0
2.5
2.0
1.5
1.0
0.5
0.0
1 2 3 4 5 6 7 8 9 10 11 12 13 14 15 16 17 18 19 20 21 22 23 24 25 26 27 28 29 30 31
1
2
3
4
Group and Phase

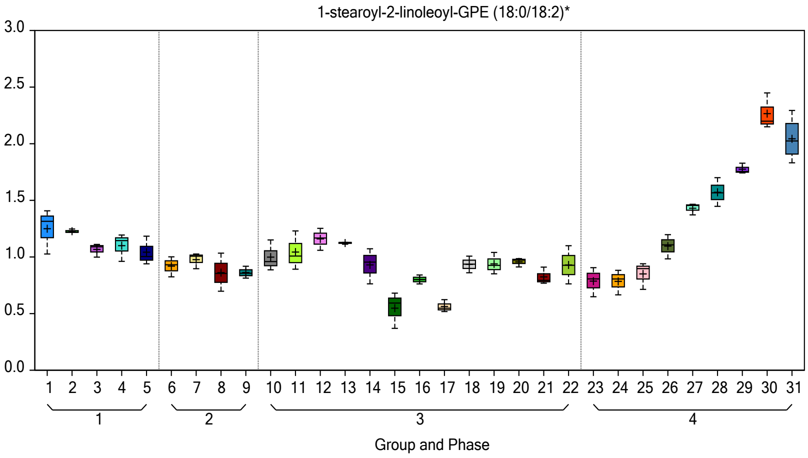
1-stearoyl-2-linoleoyl-GPE (18:0/18:2)*
3.0
2.5
2.0
1.5
1.0
0.5
0.0
1 2 3 4 5 6 7 8 9 10 11 12 13 14 15 16 17 18 19 20 21 22 23 24 25 26 27 28 29 30 31
1
2
3
4
Group and Phase

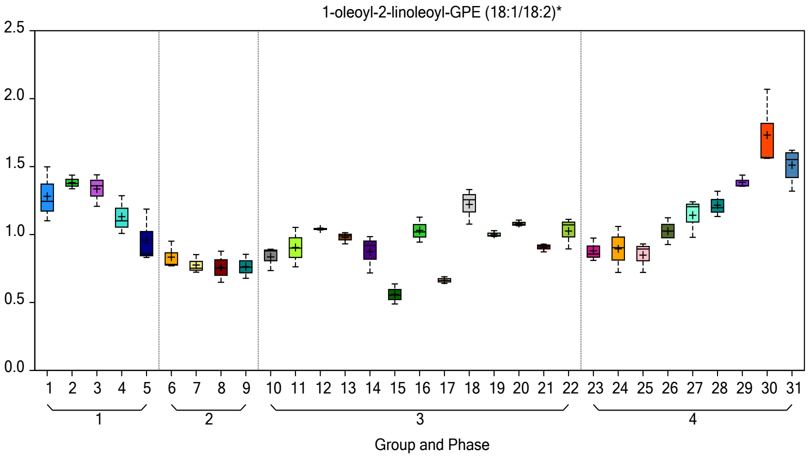
1-oleoyl-2-linoleoyl-GPE (18:1/18:2)*
2.5
2.0
1.5
1.0
0.5
0.0
1 2 3 4 5 6 7 8 9 10 11 12 13 14 15 16 17 18 19 20 21 22 23 24 25 26 27 28 29 30 31
1
2
3
4
Group and Phase

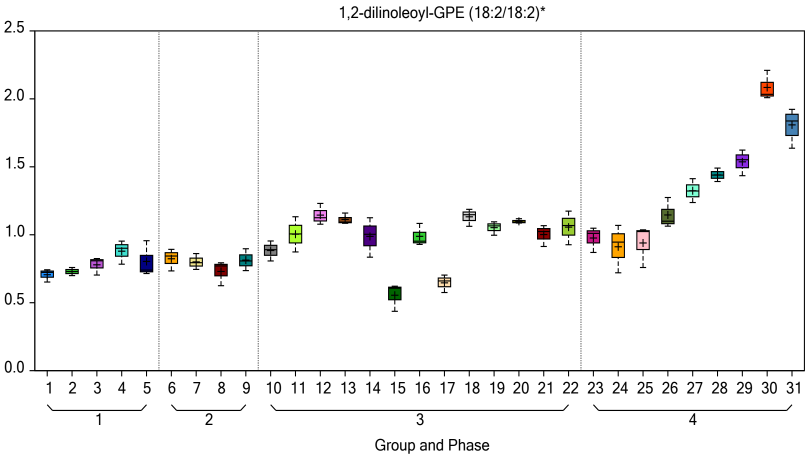
1,2-dilinoleoyl-GPE (18:2/18:2)*
2.5
2.0
1.5
1.0
0.5
0.0
1 2 3 4 5 6 7 8 9 10 11 12 13 14 15 16 17 18 19 20 21 22 23 24 25 26 27 28 29 30 31
1
2
3
4
Group and Phase

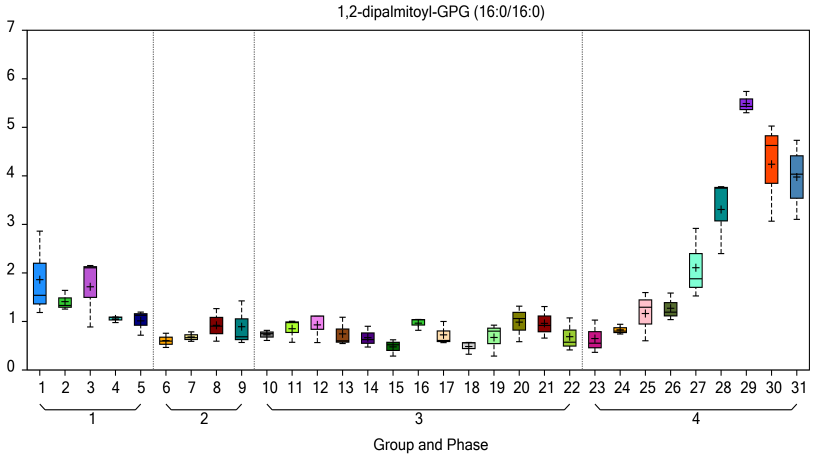
1,2-dipalmitoyl-GPG (16:0/16:0)
0
1
2
3
4
5
6
7
1 2 3 4 5 6 7 8 9 10 11 12 13 14 15 16 17 18 19 20 21 22 23 24 25 26 27 28 29 30 31
1
2
3
4
Group and Phase

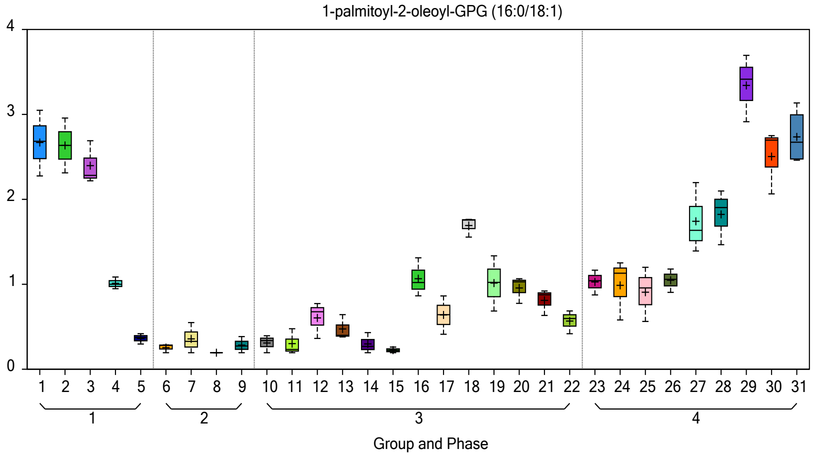
1-palmitoyl-2-oleoyl-GPG (16:0/18:1)
0
1
2
3
4
1 2 3 4 5 6 7 8 9 10 11 12 13 14 15 16 17 18 19 20 21 22 23 24 25 26 27 28 29 30 31
1
2
3
4
Group and Phase

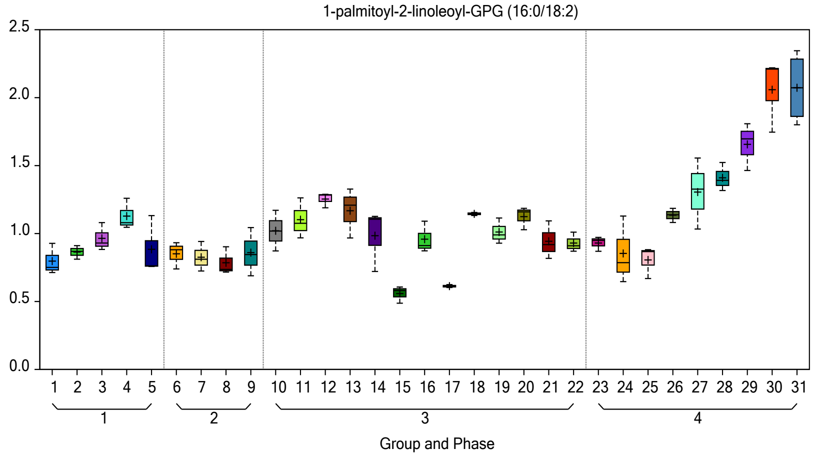
1-palmitoyl-2-linoleoyl-GPG (16:0/18:2)
0.0
0.5
1.0
1.5
2.0
2.5
1 2 3 4 5 6 7 8 9 10 11 12 13 14 15 16 17 18 19 20 21 22 23 24 25 26 27 28 29 30 31
1
2
3
4
Group and Phase

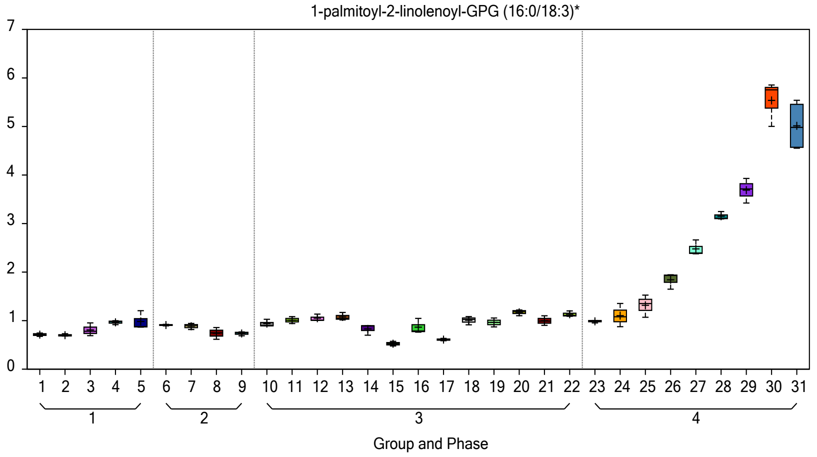
1-palmitoyl-2-linolenoyl-GPG (16:0/18:3)*
0
1
2
3
4
5
6
7
1 2 3 4 5 6 7 8 9 10 11 12 13 14 15 16 17 18 19 20 21 22 23 24 25 26 27 28 29 30 31
1
2
3
4
Group and Phase

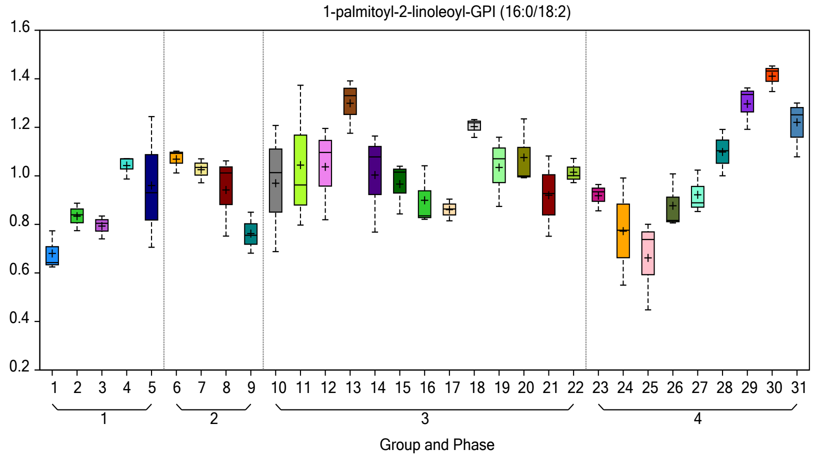
1-palmitoyl-2-linoleoyl-GPI (16:0/18:2)
0.2 0.4 0.6 0.8 1.0 1.2 1.4 1.6
1 2 3 4 5 6 7 8 9 10 11 12 13 14 15 16 17 18 19 20 21 22 23 24 25 26 27 28 29 30 31
1 2 3 4
Group and Phase

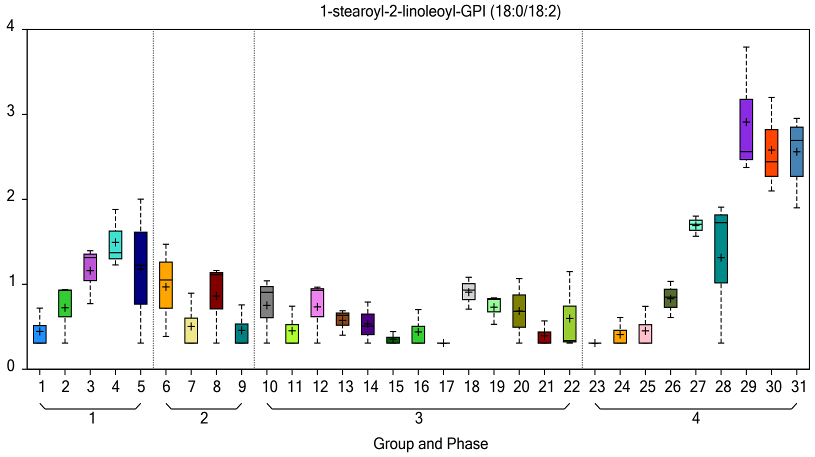
1-stearoyl-2-linoleoyl-GPI (18:0/18:2)
0 1 2 3 4
1 2 3 4 5 6 7 8 9 10 11 12 13 14 15 16 17 18 19 20 21 22 23 24 25 26 27 28 29 30 31
1 2 3 4
Group and Phase

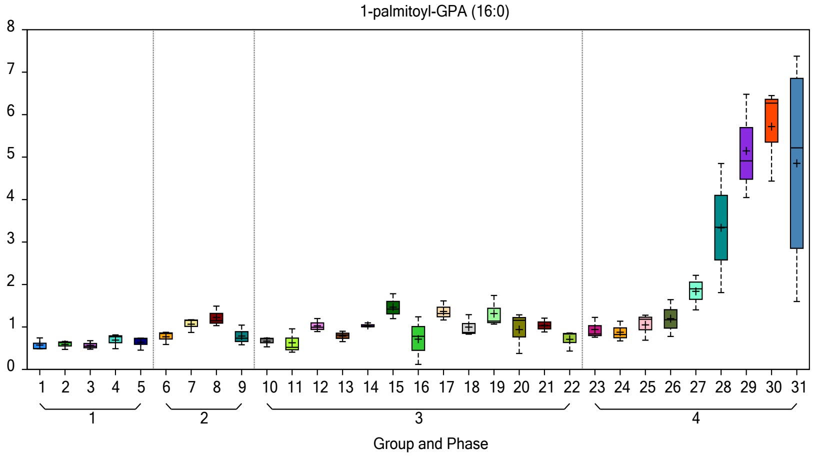
1-palmitoyl-GPA (16:0)
0 1 2 3 4 5 6 7 8
1 2 3 4 5 6 7 8 9 10 11 12 13 14 15 16 17 18 19 20 21 22 23 24 25 26 27 28 29 30 31
1 2 3 4
Group and Phase

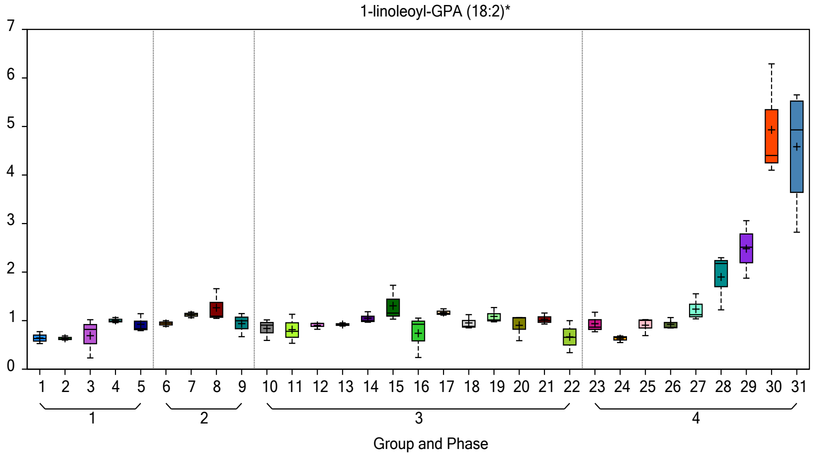
1-linoleoyl-GPA (18:2)*
0 1 2 3 4 5 6 7
1 2 3 4 5 6 7 8 9 10 11 12 13 14 15 16 17 18 19 20 21 22 23 24 25 26 27 28 29 30 31
1 2 3 4
Group and Phase

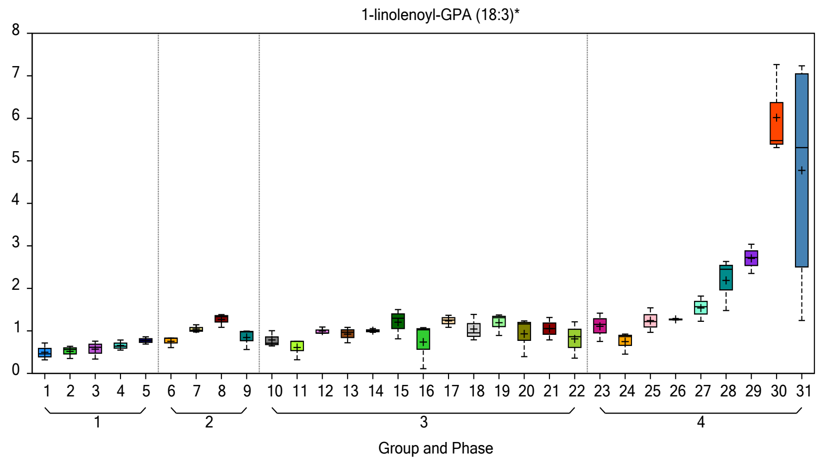
1-linolenoyl-GPA (18:3)*
0
1
2
3
4
5
6
7
8
1 2 3 4 5 6 7 8 9 10 11 12 13 14 15 16 17 18 19 20 21 22 23 24 25 26 27 28 29 30 31
1
2
3
4
Group and Phase

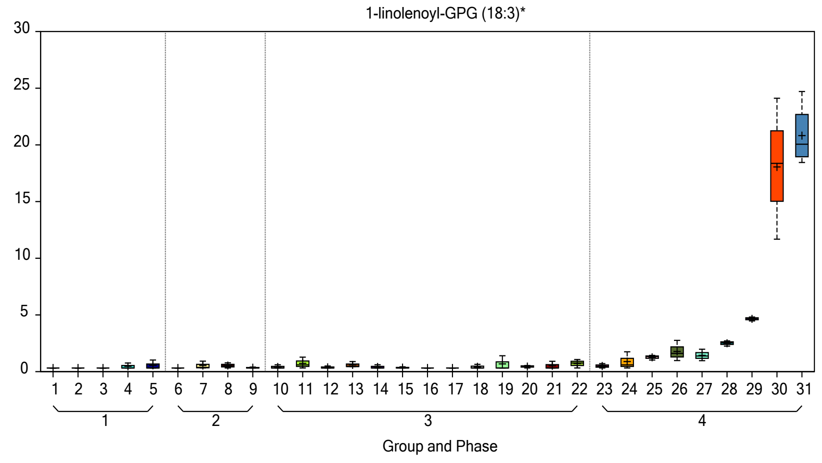
1-linolenoyl-GPG (18:3)*
0
5
10
15
20
25
30
1 2 3 4 5 6 7 8 9 10 11 12 13 14 15 16 17 18 19 20 21 22 23 24 25 26 27 28 29 30 31
1
2
3
4
Group and Phase

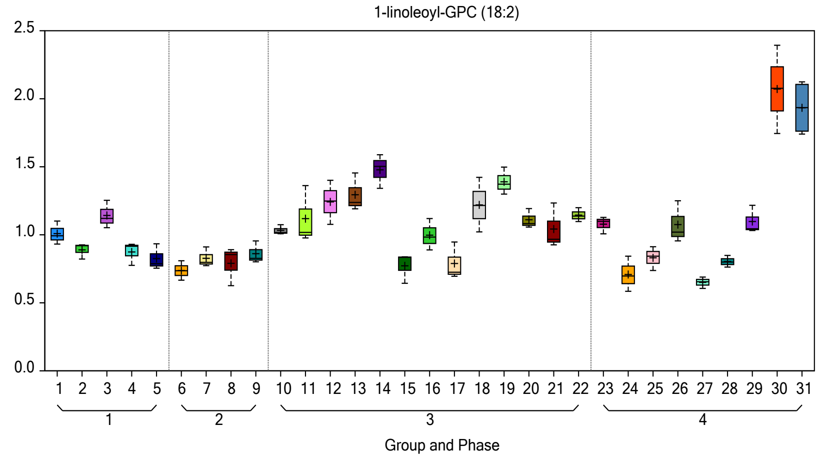
1-linoleoyl-GPC (18:2)
0.0
0.5
1.0
1.5
2.0
2.5
1 2 3 4 5 6 7 8 9 10 11 12 13 14 15 16 17 18 19 20 21 22 23 24 25 26 27 28 29 30 31
1
2
3
4
Group and Phase

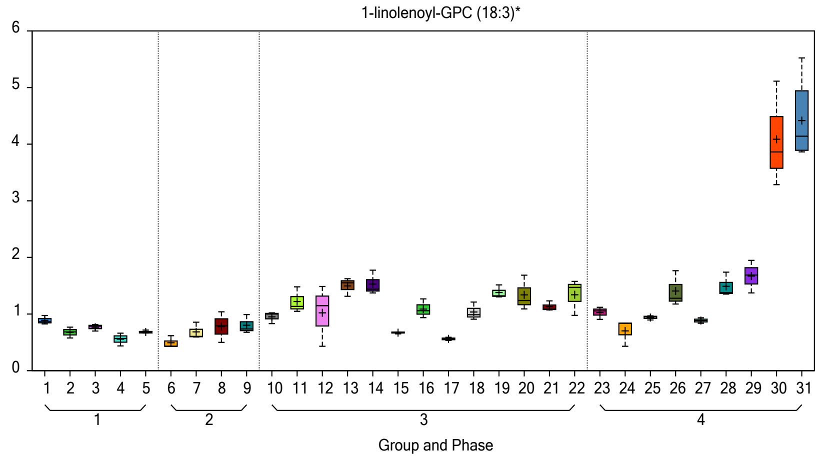
1-linolenoyl-GPC (18:3)*
0
1
2
3
4
5
6
1 2 3 4 5 6 7 8 9 10 11 12 13 14 15 16 17 18 19 20 21 22 23 24 25 26 27 28 29 30 31
1
2
3
4
Group and Phase

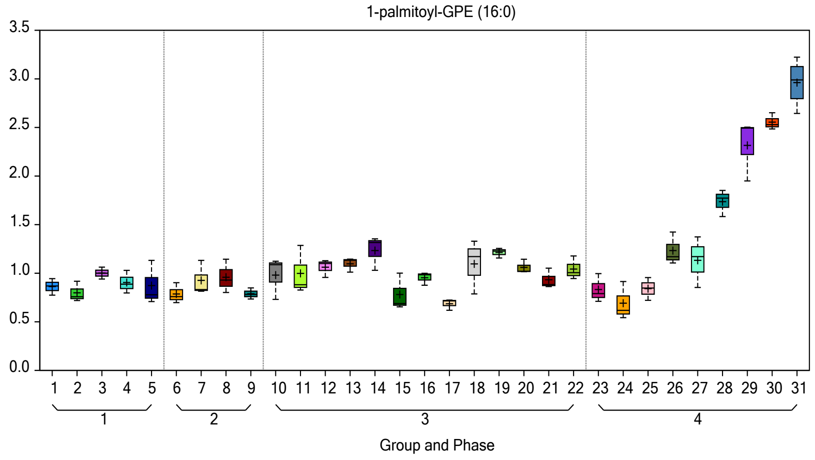
1-palmitoyl-GPE (16:0)
0.0 0.5 1.0 1.5 2.0 2.5 3.0 3.5
1 2 3 4 5 6 7 8 9 10 11 12 13 14 15 16 17 18 19 20 21 22 23 24 25 26 27 28 29 30 31
1 2 3 4
Group and Phase

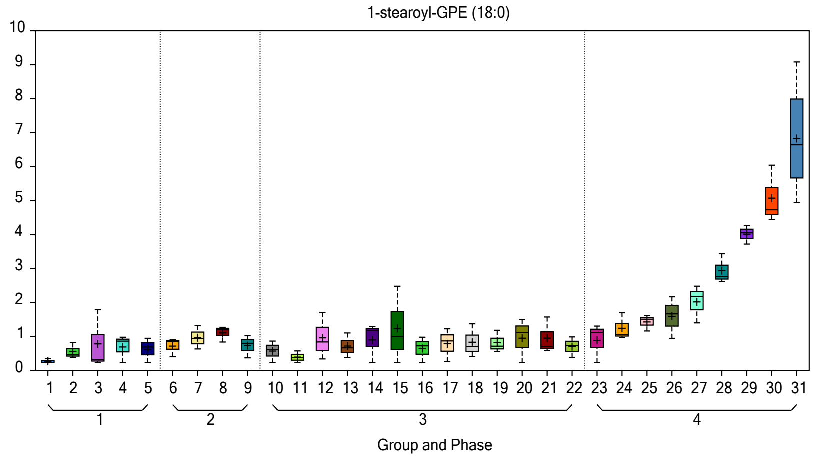
1-stearoyl-GPE (18:0)
0 1 2 3 4 5 6 7 8 9 10
1 2 3 4 5 6 7 8 9 10 11 12 13 14 15 16 17 18 19 20 21 22 23 24 25 26 27 28 29 30 31
1 2 3 4
Group and Phase

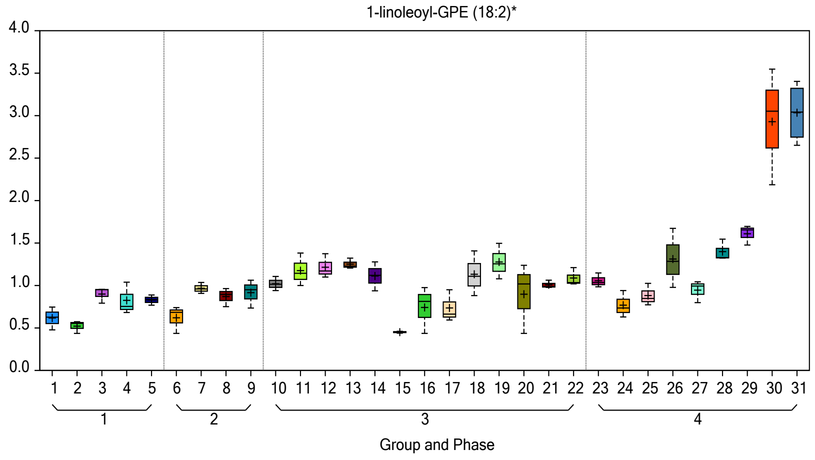
1-linoleoyl-GPE (18:2)*
0.0 0.5 1.0 1.5 2.0 2.5 3.0 3.5 4.0
1 2 3 4 5 6 7 8 9 10 11 12 13 14 15 16 17 18 19 20 21 22 23 24 25 26 27 28 29 30 31
1 2 3 4
Group and Phase

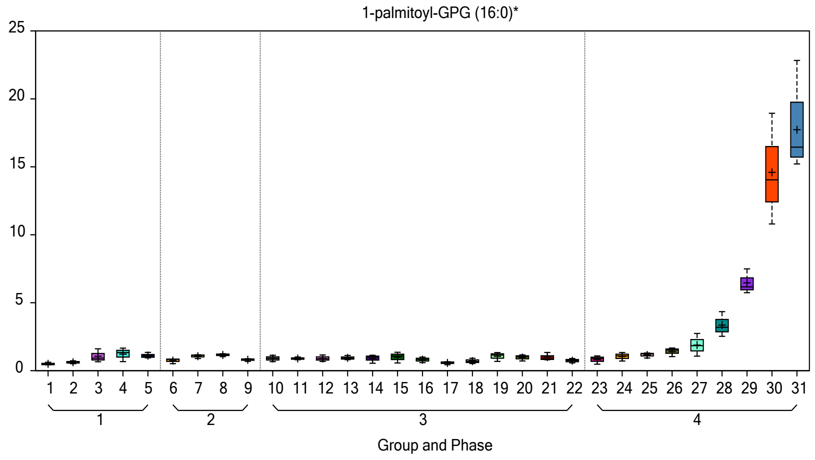
1-palmitoyl-GPG (16:0)*
0 5 10 15 20 25
1 2 3 4 5 6 7 8 9 10 11 12 13 14 15 16 17 18 19 20 21 22 23 24 25 26 27 28 29 30 31
1 2 3 4
Group and Phase

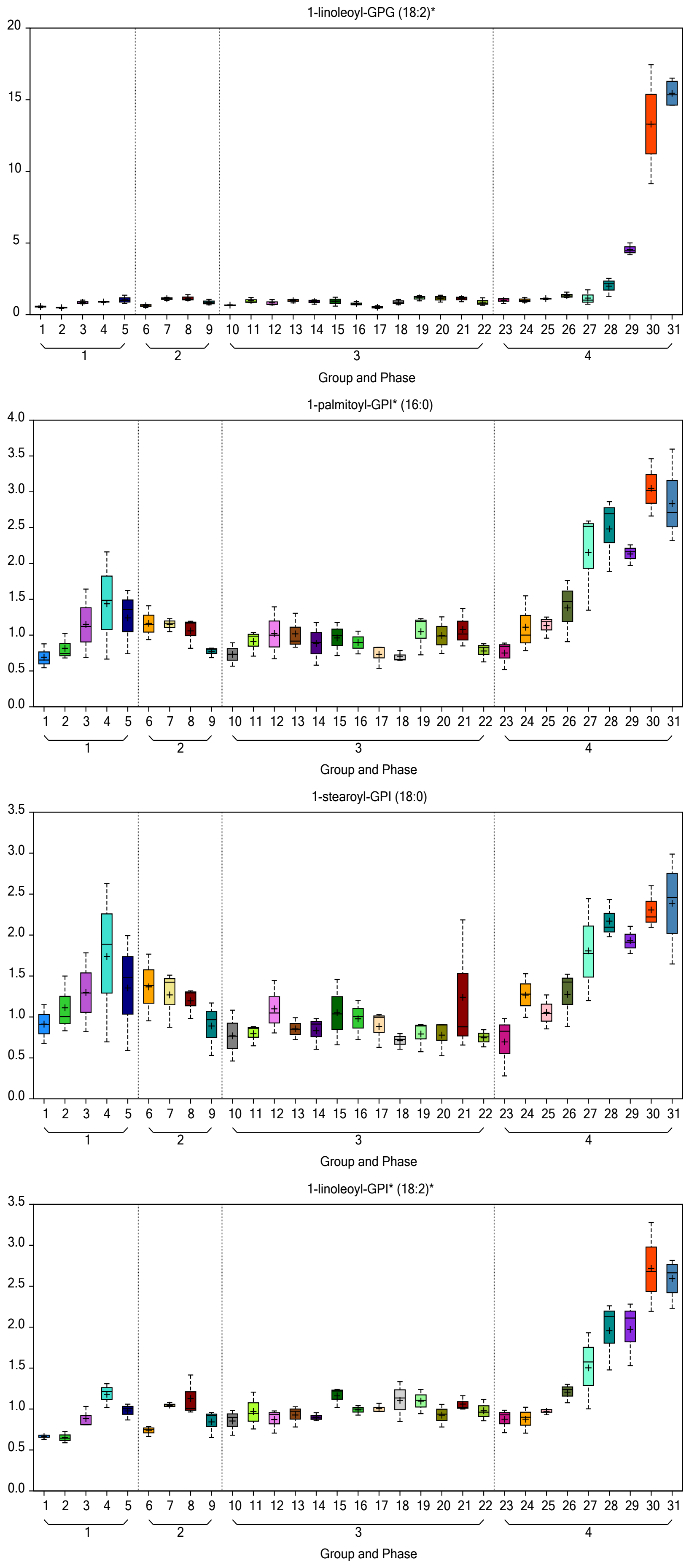
1-linoleoyl-GPG (18:2)*
1-palmitoyl-GPI* (16:0)
1-stearoyl-GPI (18:0)
1-linoleoyl-GPI* (18:2)*
Group and Phase

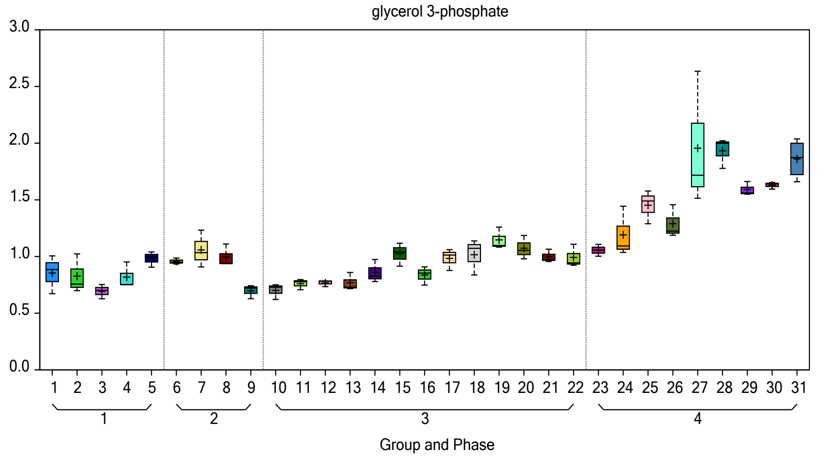
glycerol 3-phosphate
3.0
2.5
2.0
1.5
1.0
0.5
0.0
1 2 3 4 5 6 7 8 9 10 11 12 13 14 15 16 17 18 19 20 21 22 23 24 25 26 27 28 29 30 31
1
2
3
4
Group and Phase

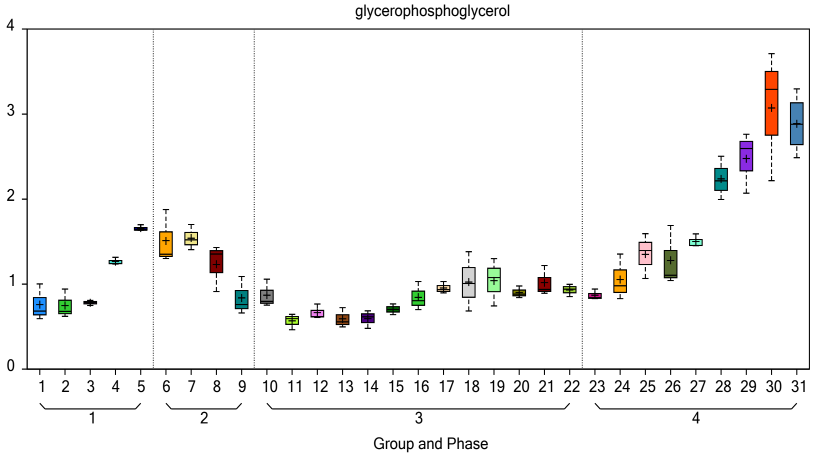
glycerophosphoglycerol
4
3
2
1
0
1 2 3 4 5 6 7 8 9 10 11 12 13 14 15 16 17 18 19 20 21 22 23 24 25 26 27 28 29 30 31
1
2
3
4
Group and Phase

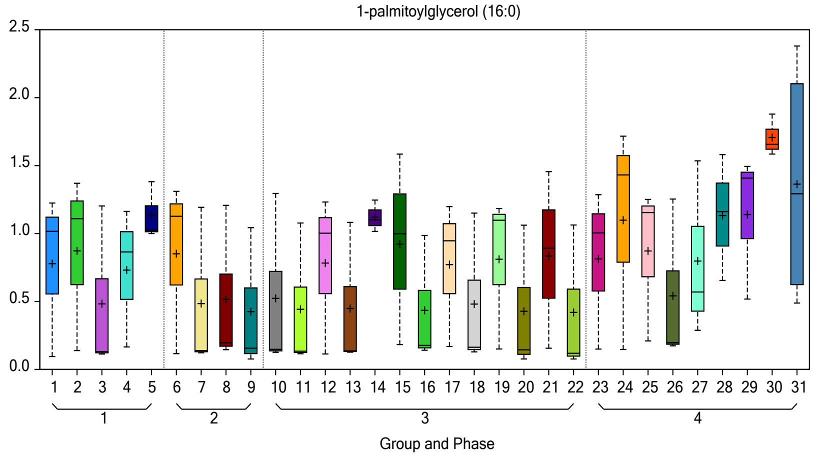
1-palmitoylglycerol (16:0)
2.5
2.0
1.5
1.0
0.5
0.0
1 2 3 4 5 6 7 8 9 10 11 12 13 14 15 16 17 18 19 20 21 22 23 24 25 26 27 28 29 30 31
1
2
3
4
Group and Phase

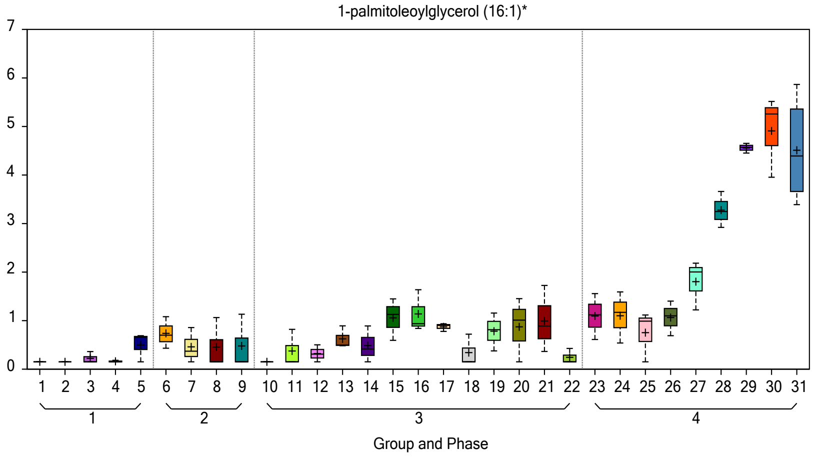
1-palmitoleoylglycerol (16:1)*
7
6
5
4
3
2
1
0
1 2 3 4 5 6 7 8 9 10 11 12 13 14 15 16 17 18 19 20 21 22 23 24 25 26 27 28 29 30 31
1
2
3
4
Group and Phase

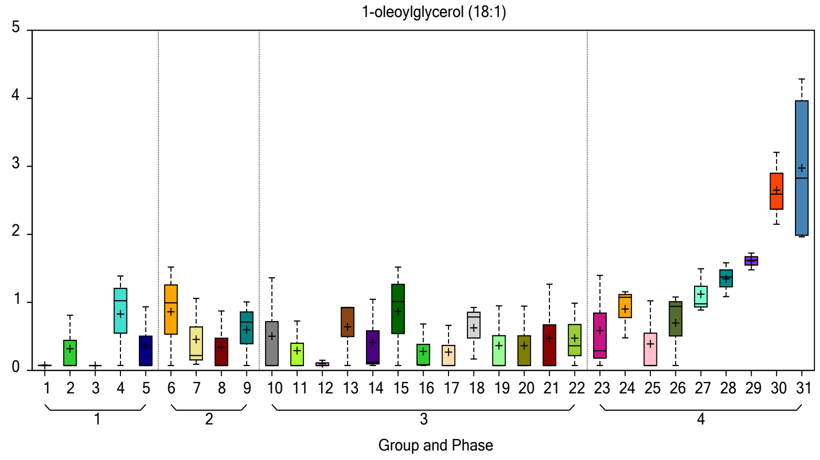
1-oleoylglycerol (18:1)
Group and Phase

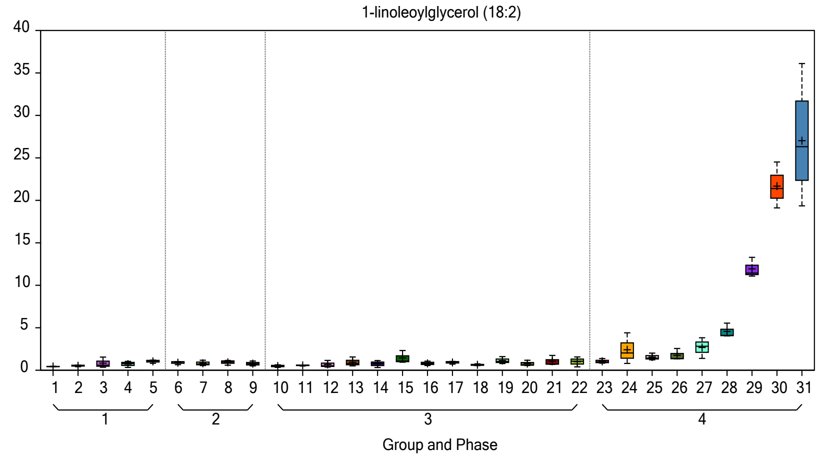
1-linoleoylglycerol (18:2)
Group and Phase

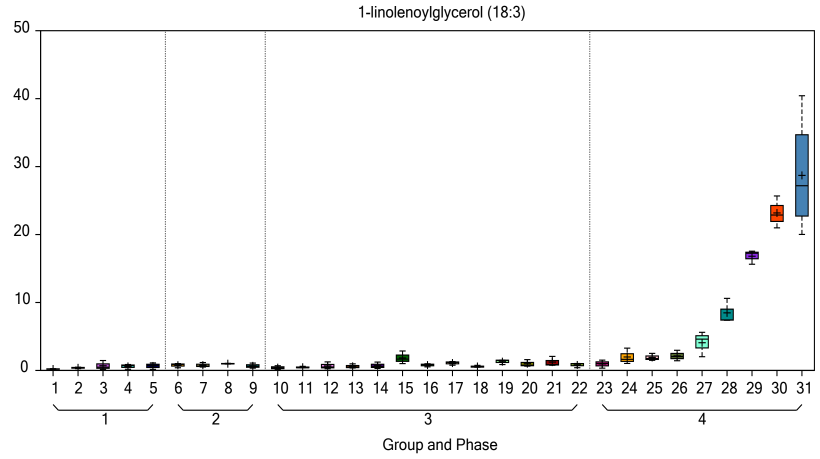
1-linolenoylglycerol (18:3)
Group and Phase

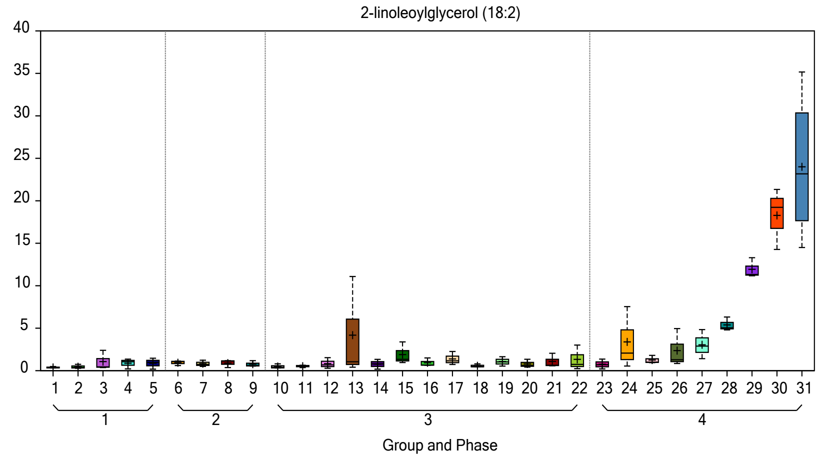
2-linoleoylglycerol (18:2)
Group and Phase

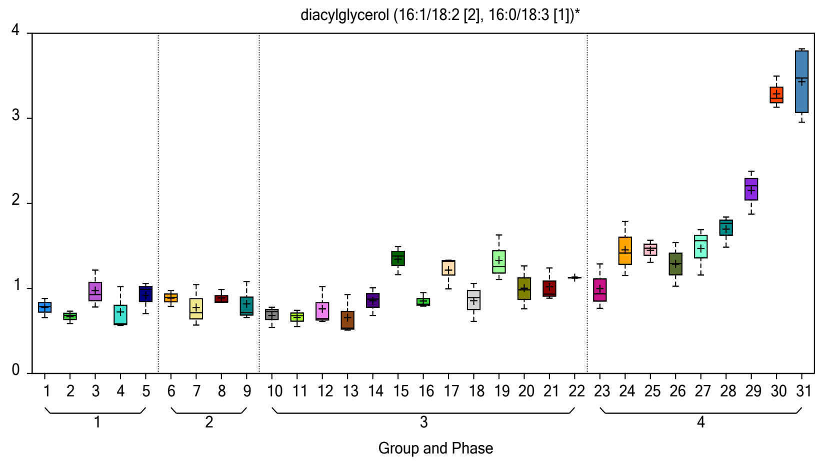
diacylglycerol (16:1/18:2 [2], 16:0/18:3 [1])*
Group and Phase

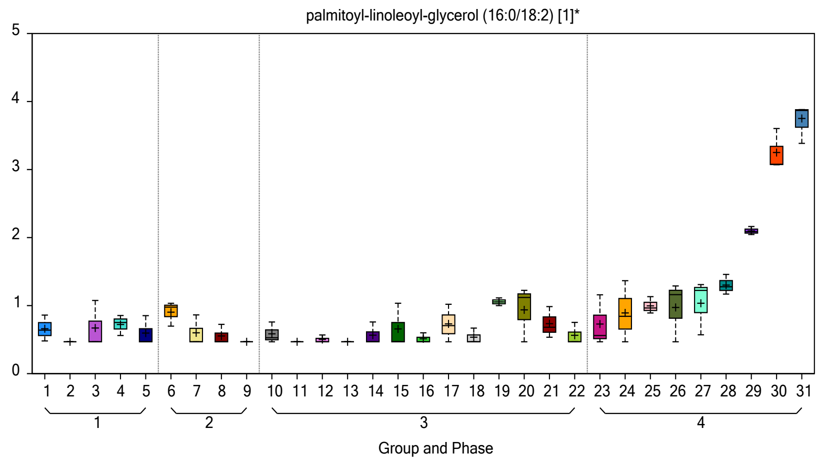
palmitoyl-linoleoyl-glycerol (16:0/18:2) [1]*
Group and Phase

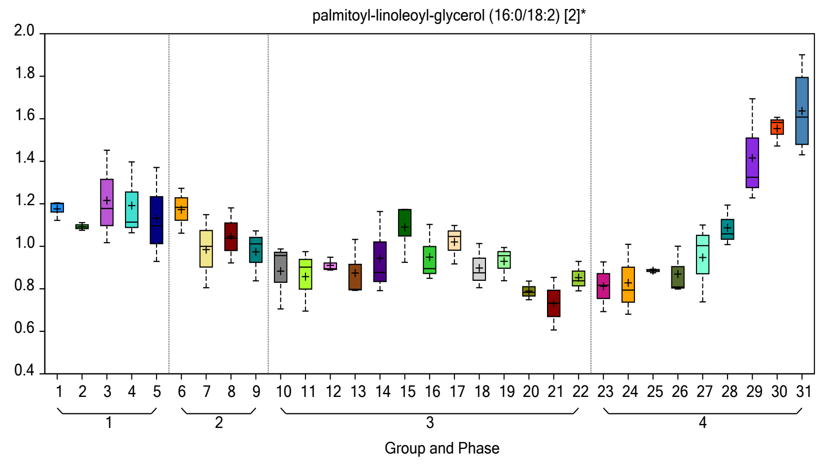
palmitoyl-linoleoyl-glycerol (16:0/18:2) [2]*
Group and Phase

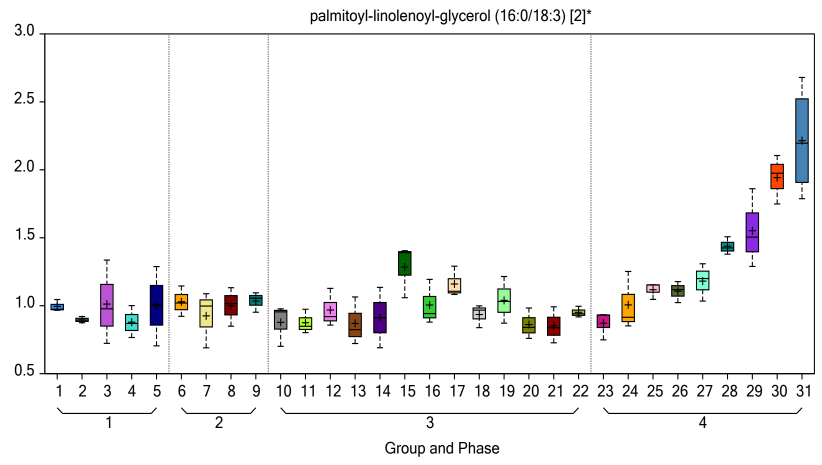
palmitoyl-linolenoyl-glycerol (16:0/18:3) [2]*
Group and Phase

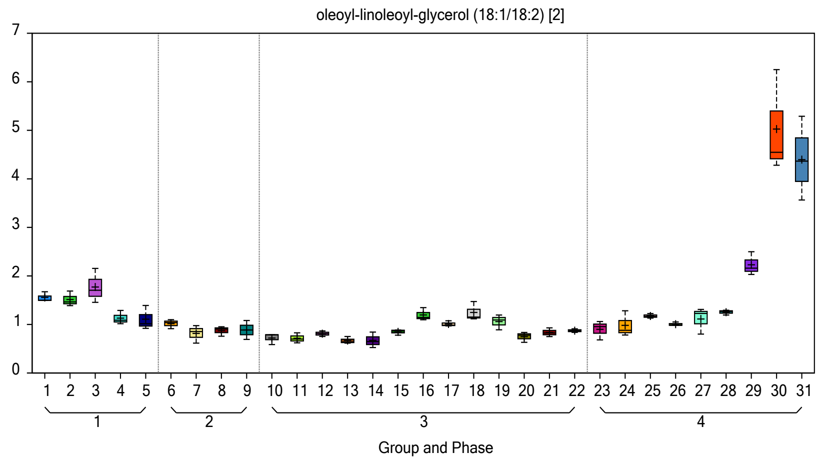
oleoyl-linoleoyl-glycerol (18:1/18:2) [2]
0
1
2
3
4
5
6
7
1 2 3 4 5 6 7 8 9 10 11 12 13 14 15 16 17 18 19 20 21 22 23 24 25 26 27 28 29 30 31
1
2
3
4
Group and Phase

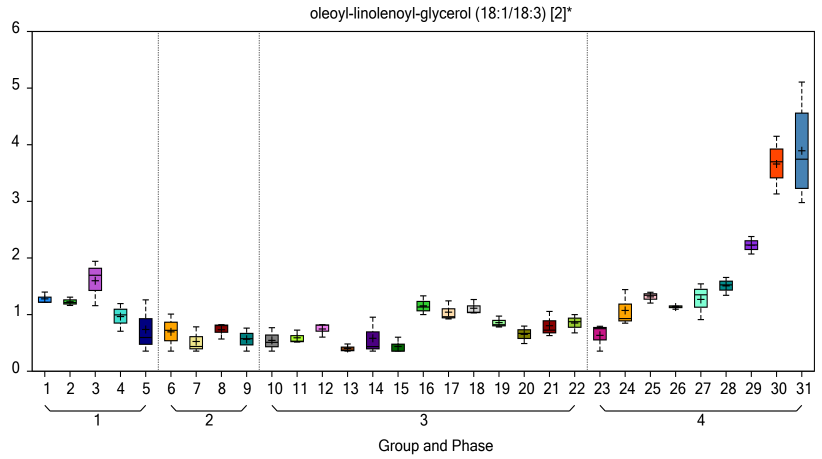
oleoyl-linolenoyl-glycerol (18:1/18:3) [2]*
0
1
2
3
4
5
6
1 2 3 4 5 6 7 8 9 10 11 12 13 14 15 16 17 18 19 20 21 22 23 24 25 26 27 28 29 30 31
1
2
3
4
Group and Phase

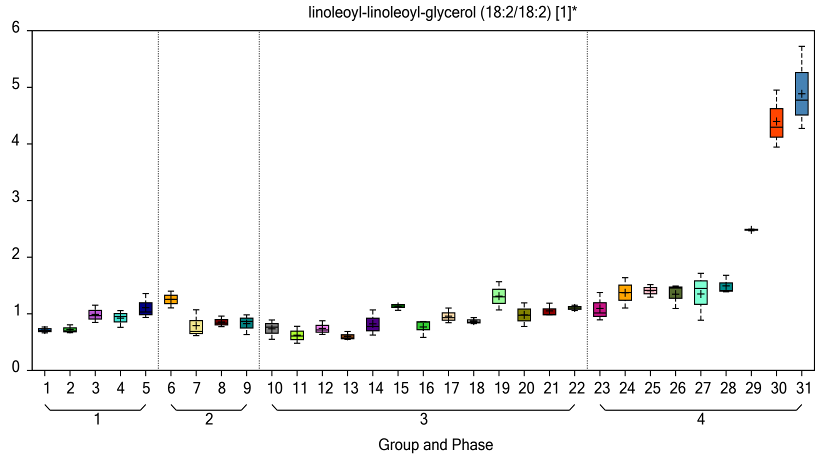
linoleoyl-linoleoyl-glycerol (18:2/18:2) [1]*
0
1
2
3
4
5
6
1 2 3 4 5 6 7 8 9 10 11 12 13 14 15 16 17 18 19 20 21 22 23 24 25 26 27 28 29 30 31
1
2
3
4
Group and Phase

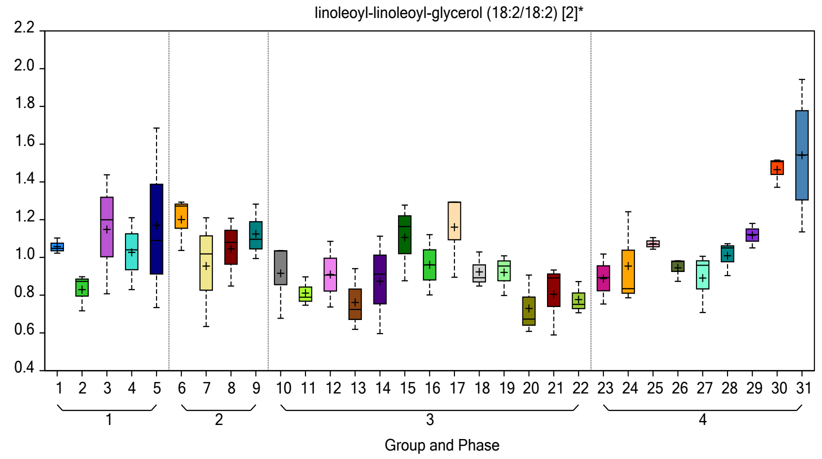
linoleoyl-linoleoyl-glycerol (18:2/18:2) [2]*
0.4
0.6
0.8
1.0
1.2
1.4
1.6
1.8
2.0
2.2
1 2 3 4 5 6 7 8 9 10 11 12 13 14 15 16 17 18 19 20 21 22 23 24 25 26 27 28 29 30 31
1
2
3
4
Group and Phase

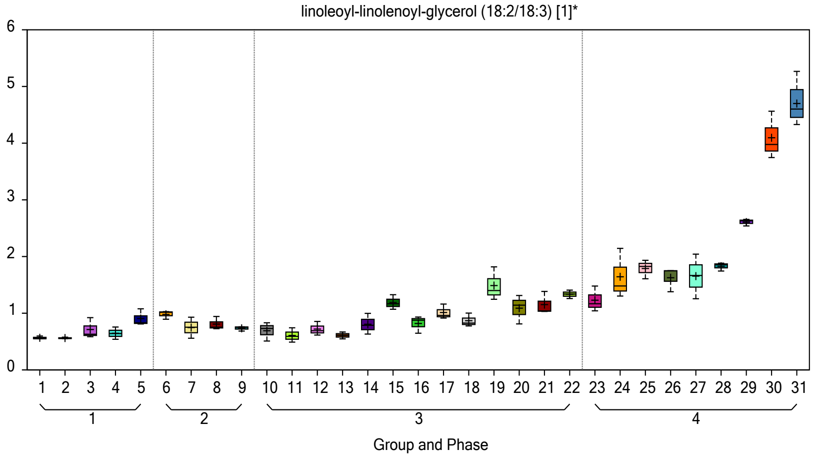
linoleoyl-linolenoyl-glycerol (18:2/18:3) [1]*
0
1
2
3
4
5
6
1 2 3 4 5 6 7 8 9 10 11 12 13 14 15 16 17 18 19 20 21 22 23 24 25 26 27 28 29 30 31
1
2
3
4
Group and Phase

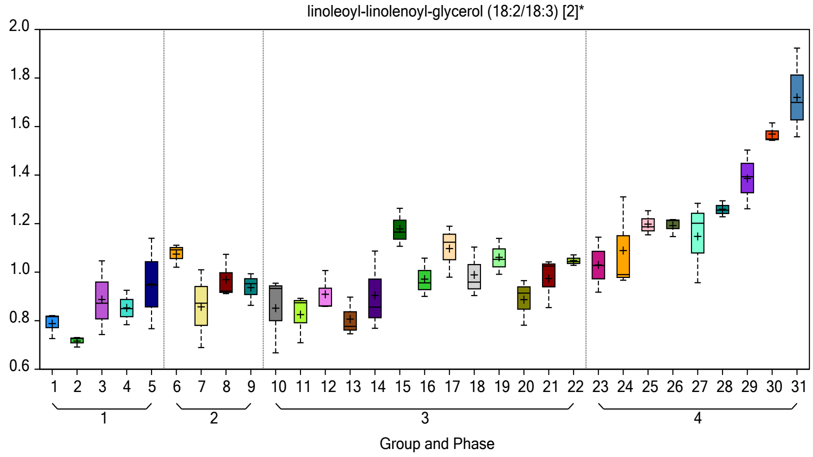
linoleoyl-linolenoyl-glycerol (18:2/18:3) [2]*
0.6
0.8
1.0
1.2
1.4
1.6
1.8
2.0
1 2 3 4 5 6 7 8 9 10 11 12 13 14 15 16 17 18 19 20 21 22 23 24 25 26 27 28 29 30 31
1
2
3
4
Group and Phase

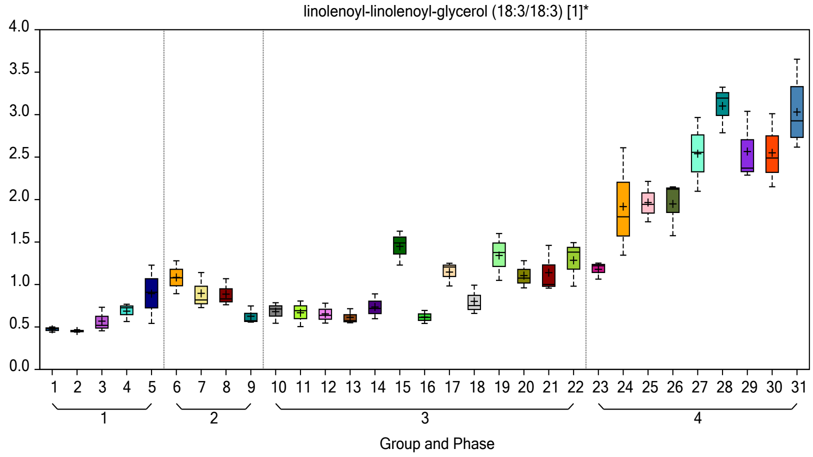
linolenoyl-linolenoyl-glycerol (18:3/18:3) [1]*
0.0
0.5
1.0
1.5
2.0
2.5
3.0
3.5
4.0
1 2 3 4 5 6 7 8 9 10 11 12 13 14 15 16 17 18 19 20 21 22 23 24 25 26 27 28 29 30 31
1
2
3
4
Group and Phase

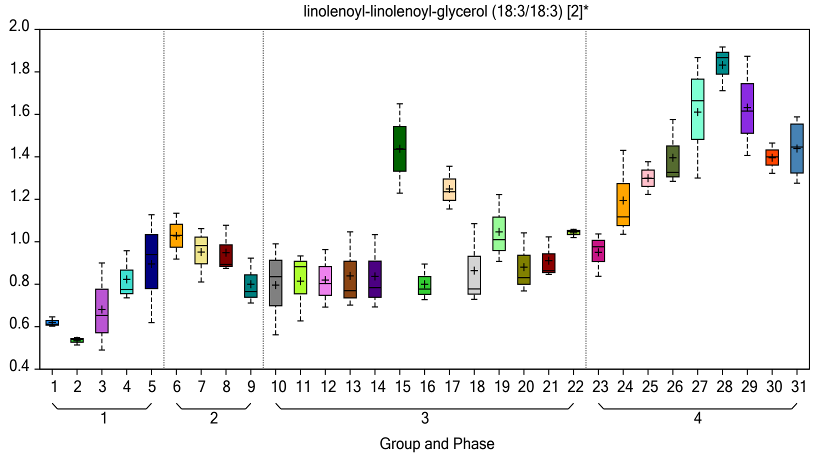
linolenoyl-linolenoyl-glycerol (18:3/18:3) [2]*
0.4
0.6
0.8
1.0
1.2
1.4
1.6
1.8
2.0
1 2 3 4 5 6 7 8 9 10 11 12 13 14 15 16 17 18 19 20 21 22 23 24 25 26 27 28 29 30 31
1
2
3
4
Group and Phase

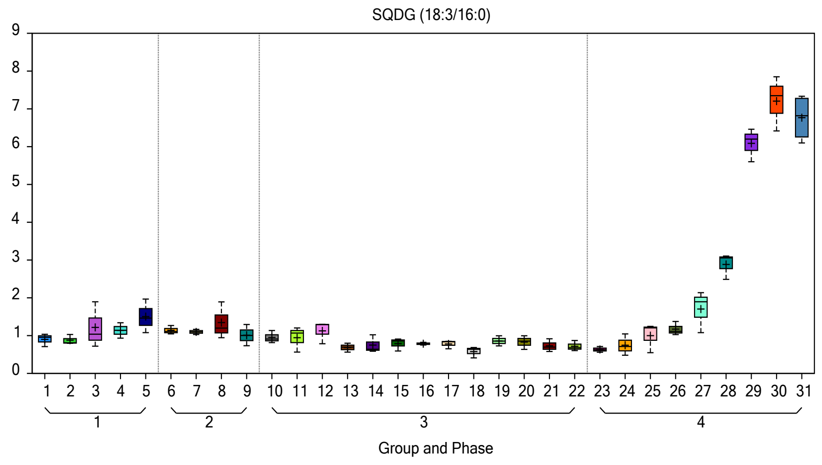
SQDG (18:3/16:0)
0 1 2 3 4 5 6 7 8 9
1 2 3 4 5 6 7 8 9 10 11 12 13 14 15 16 17 18 19 20 21 22 23 24 25 26 27 28 29 30 31
1 2 3 4
Group and Phase

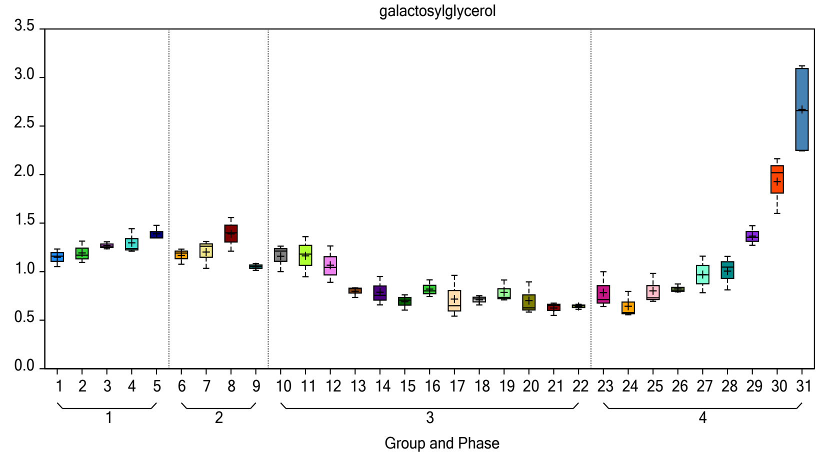
galactosylglycerol
0.0 0.5 1.0 1.5 2.0 2.5 3.0 3.5
1 2 3 4 5 6 7 8 9 10 11 12 13 14 15 16 17 18 19 20 21 22 23 24 25 26 27 28 29 30 31
1 2 3 4
Group and Phase

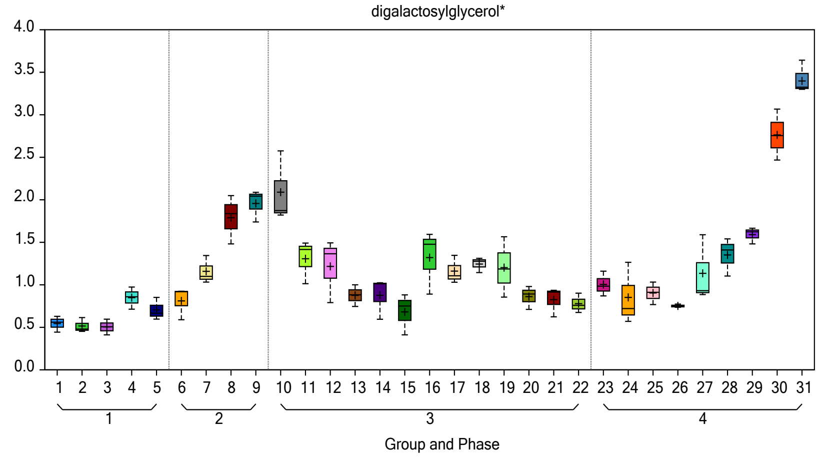
digalactosylglycerol*
0.0 0.5 1.0 1.5 2.0 2.5 3.0 3.5 4.0
1 2 3 4 5 6 7 8 9 10 11 12 13 14 15 16 17 18 19 20 21 22 23 24 25 26 27 28 29 30 31
1 2 3 4
Group and Phase

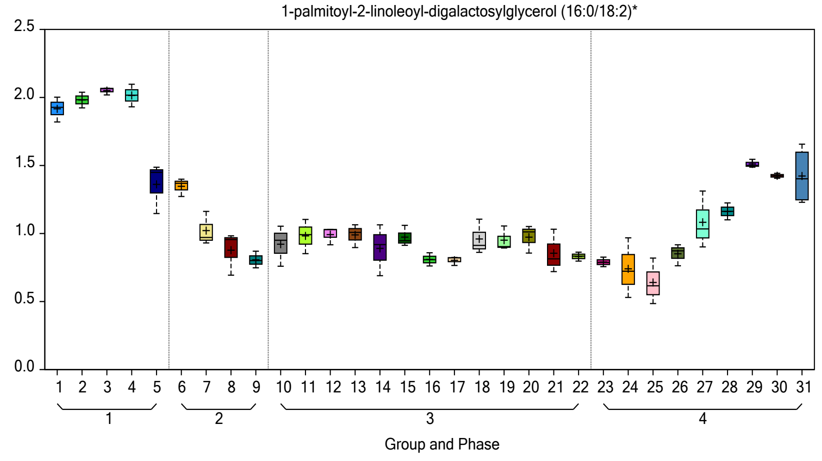
1-palmitoyl-2-linoleoyl-digalactosylglycerol (16:0/18:2)*
0.0 0.5 1.0 1.5 2.0 2.5
1 2 3 4 5 6 7 8 9 10 11 12 13 14 15 16 17 18 19 20 21 22 23 24 25 26 27 28 29 30 31
1 2 3 4
Group and Phase

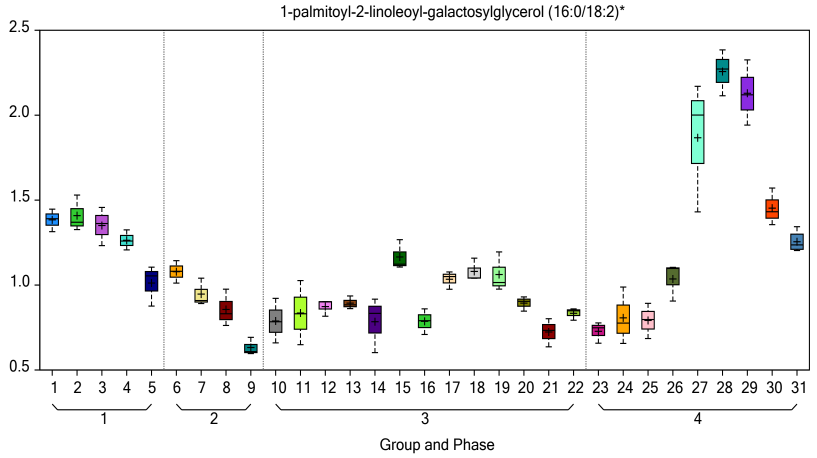
1-palmitoyl-2-linoleoyl-galactosylglycerol (16:0/18:2)*
Group and Phase

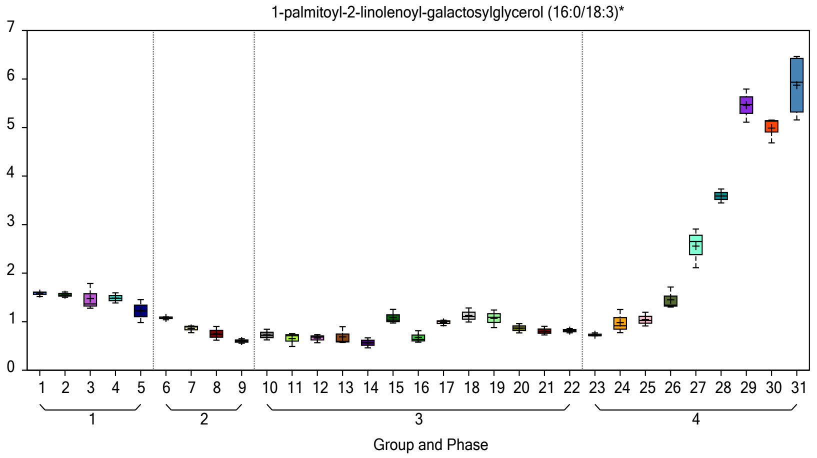
1-palmitoyl-2-linolenoyl-galactosylglycerol (16:0/18:3)*
Group and Phase

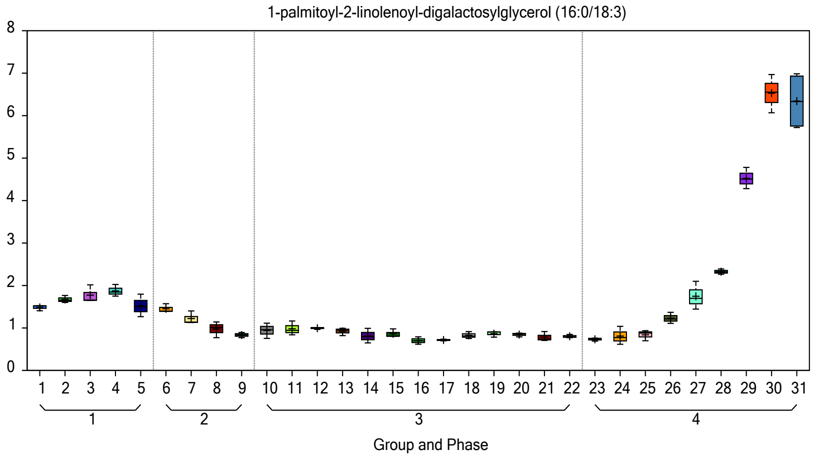
1-palmitoyl-2-linolenoyl-digalactosylglycerol (16:0/18:3)
Group and Phase

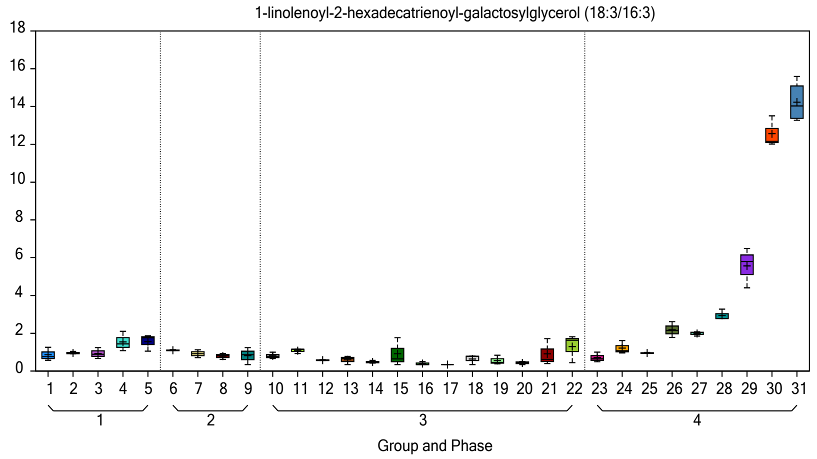
1-linolenoyl-2-hexadecatrienoyl-galactosylglycerol (18:3/16:3)
Group and Phase

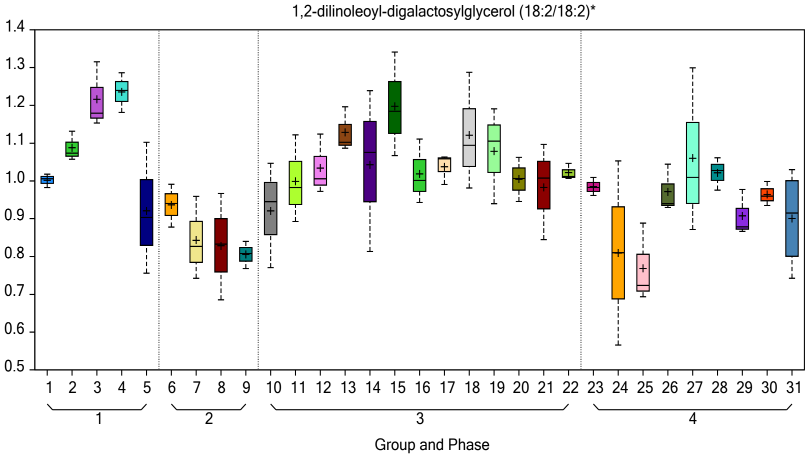
1,2-dilinoleoyl-digalactosylglycerol (18:2/18:2)*
1.4
1.3
1.2
1.1
1.0
0.9
0.8
0.7
0.6
0.5
1 2 3 4 5 6 7 8 9 10 11 12 13 14 15 16 17 18 19 20 21 22 23 24 25 26 27 28 29 30 31
1
2
3
4
Group and Phase

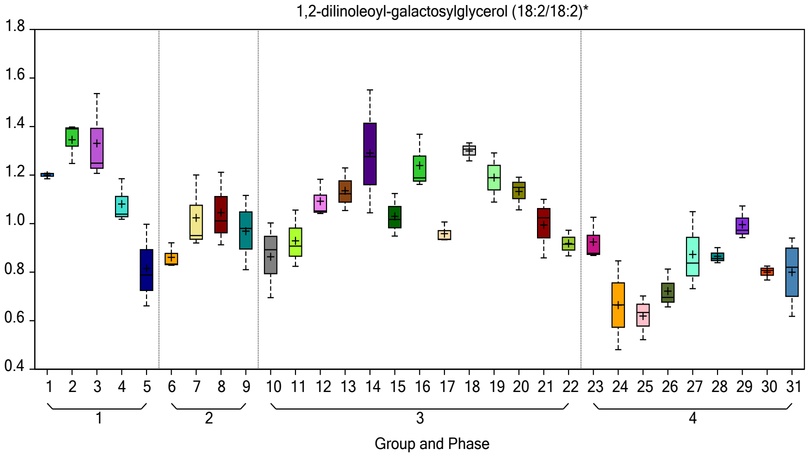
1,2-dilinoleoyl-galactosylglycerol (18:2/18:2)*
1.8
1.6
1.4
1.2
1.0
0.8
0.6
0.4
1 2 3 4 5 6 7 8 9 10 11 12 13 14 15 16 17 18 19 20 21 22 23 24 25 26 27 28 29 30 31
1
2
3
4
Group and Phase

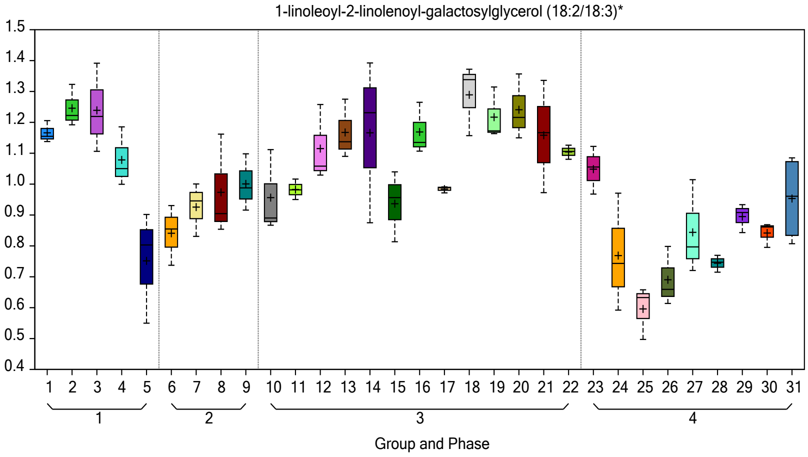
1-linoleoyl-2-linolenoyl-galactosylglycerol (18:2/18:3)*
1.5
1.4
1.3
1.2
1.1
1.0
0.9
0.8
0.7
0.6
0.5
0.4
1 2 3 4 5 6 7 8 9 10 11 12 13 14 15 16 17 18 19 20 21 22 23 24 25 26 27 28 29 30 31
1
2
3
4
Group and Phase

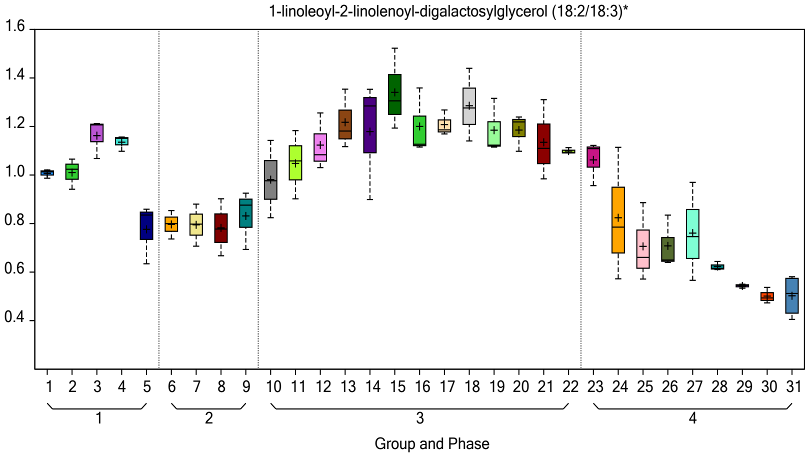
1-linoleoyl-2-linolenoyl-digalactosylglycerol (18:2/18:3)*
1.6
1.4
1.2
1.0
0.8
0.6
0.4
1 2 3 4 5 6 7 8 9 10 11 12 13 14 15 16 17 18 19 20 21 22 23 24 25 26 27 28 29 30 31
1
2
3
4
Group and Phase

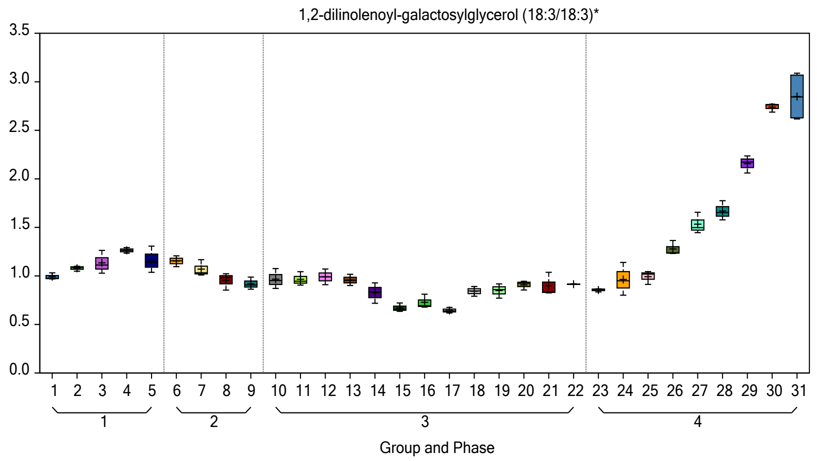
1,2-dilinolenoyl-galactosylglycerol (18:3/18:3)*
Group and Phase

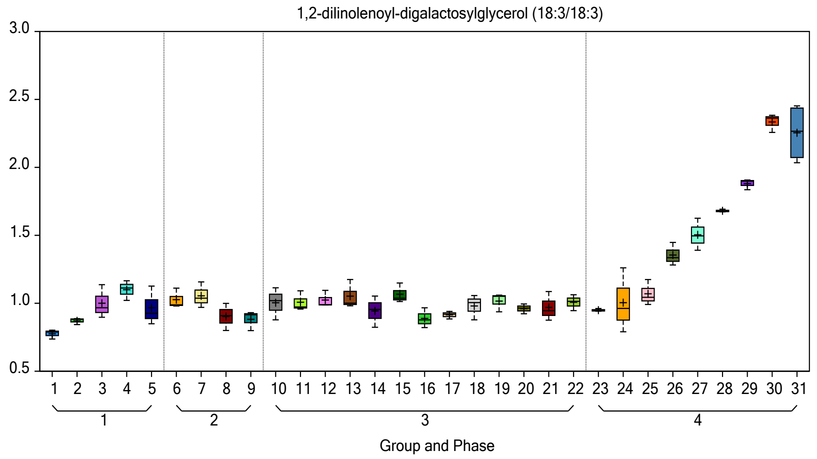
1,2-dilinolenoyl-digalactosylglycerol (18:3/18:3)
Group and Phase

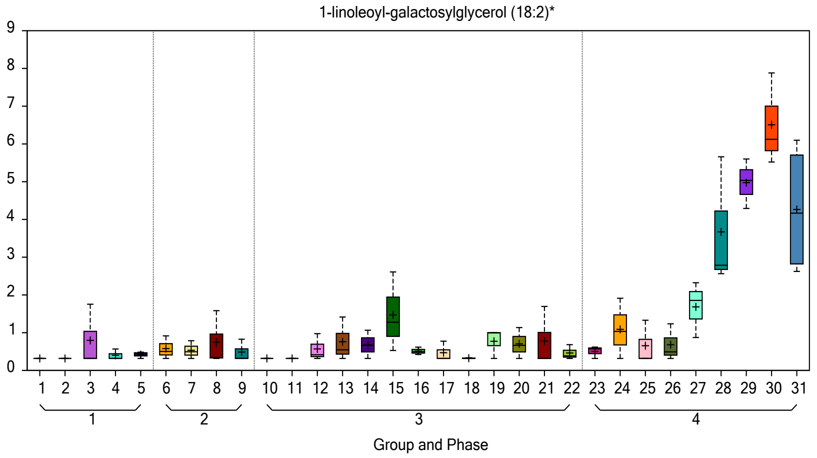
1-linoleoyl-galactosylglycerol (18:2)*
Group and Phase

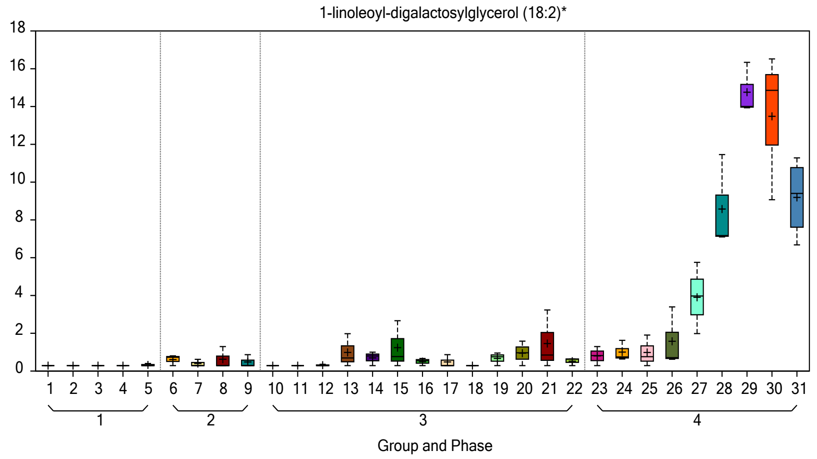
1-linoleoyl-digalactosylglycerol (18:2)*
Group and Phase

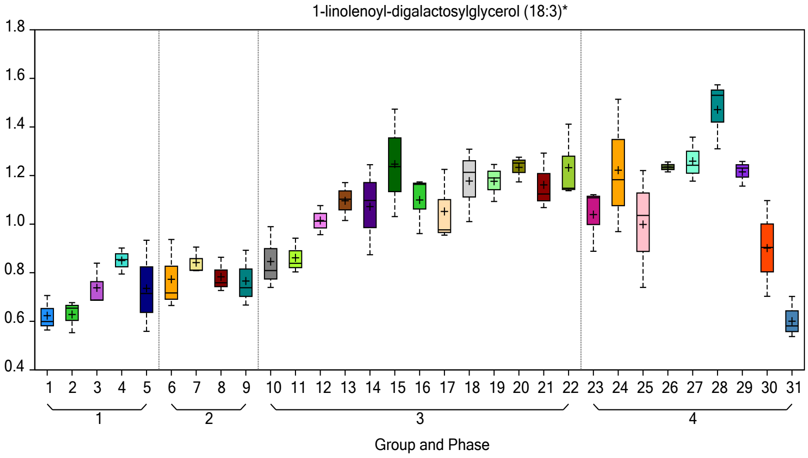
1-linolenoyl-digalactosylglycerol (18:3)*
Group and Phase

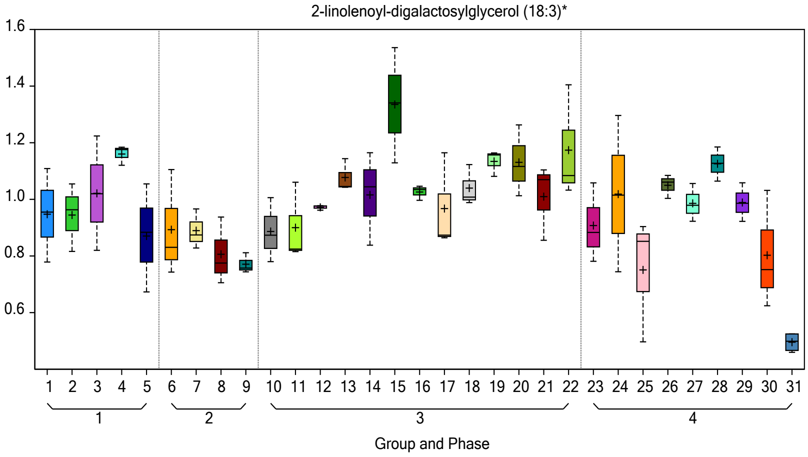
2-linolenoyl-digalactosylglycerol (18:3)*
Group and Phase

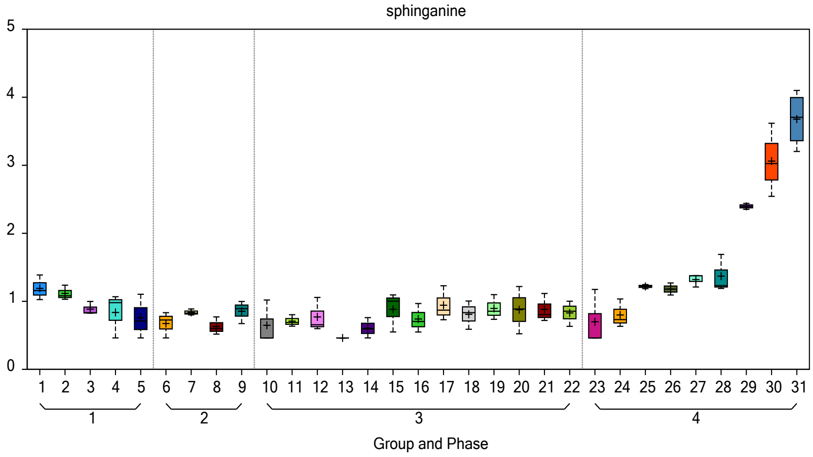
sphinganine
Group and Phase

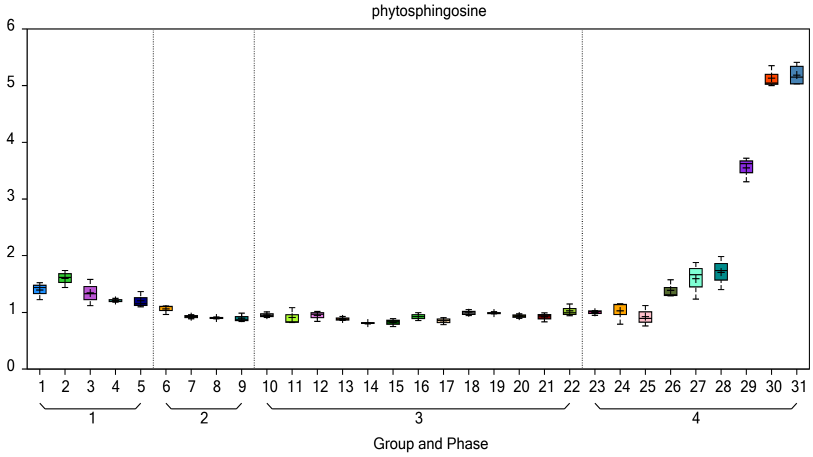
phytosphingosine
Group and Phase

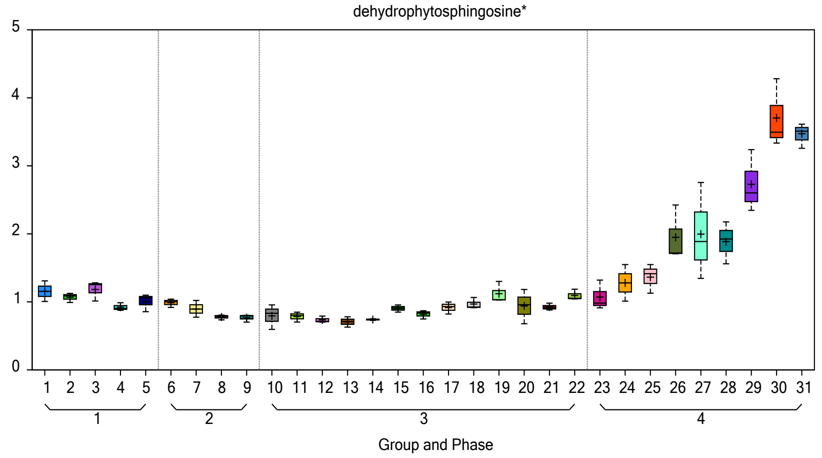
dehydrophytosphingosine*
0
1
2
3
4
5
1 2 3 4 5 6 7 8 9 10 11 12 13 14 15 16 17 18 19 20 21 22 23 24 25 26 27 28 29 30 31
1
2
3
4
Group and Phase

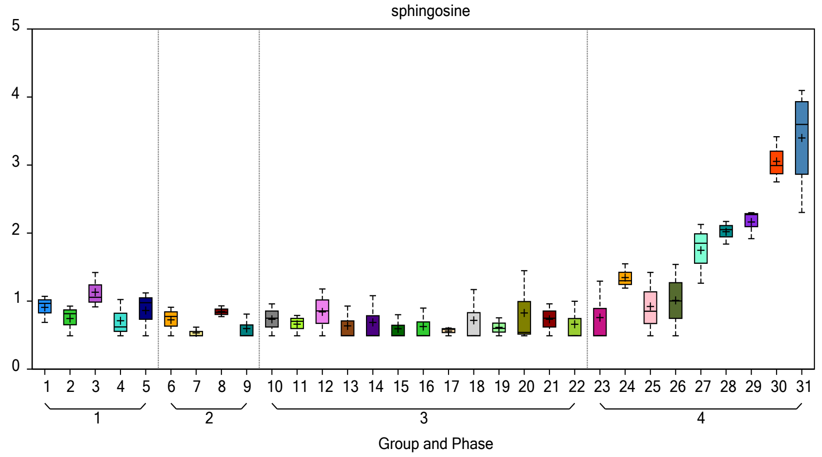
sphingosine
0
1
2
3
4
5
1 2 3 4 5 6 7 8 9 10 11 12 13 14 15 16 17 18 19 20 21 22 23 24 25 26 27 28 29 30 31
1
2
3
4
Group and Phase

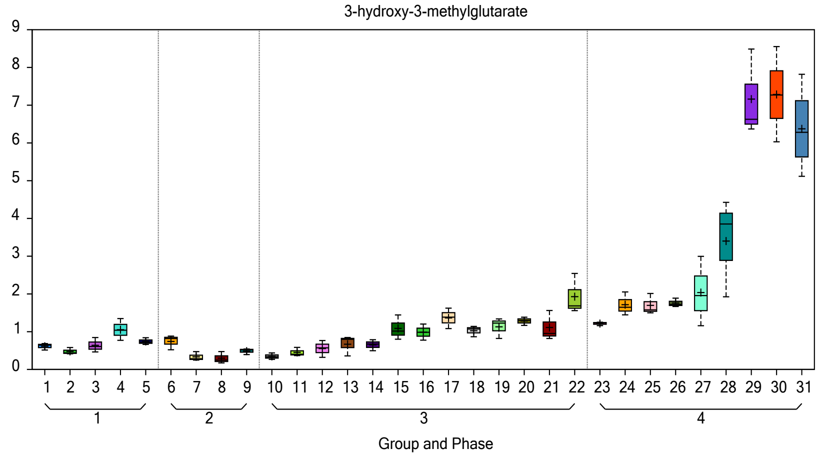
3-hydroxy-3-methylglutarate
0
1
2
3
4
5
6
7
8
9
1 2 3 4 5 6 7 8 9 10 11 12 13 14 15 16 17 18 19 20 21 22 23 24 25 26 27 28 29 30 31
1
2
3
4
Group and Phase

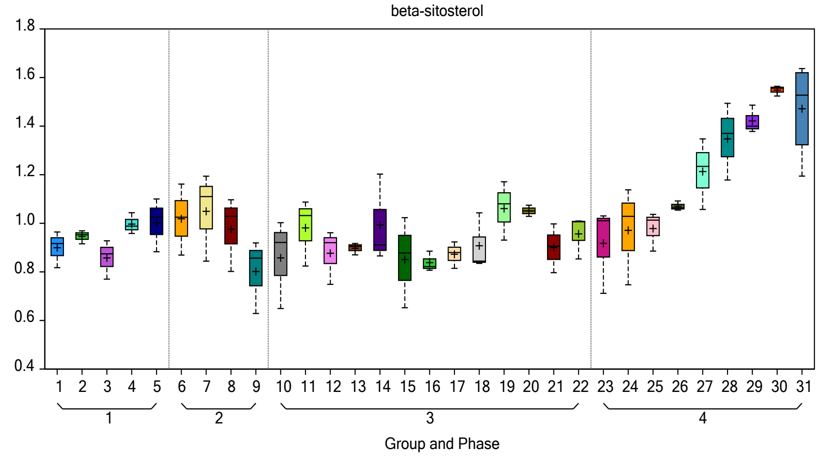
beta-sitosterol
0.4
0.6
0.8
1.0
1.2
1.4
1.6
1.8
1 2 3 4 5 6 7 8 9 10 11 12 13 14 15 16 17 18 19 20 21 22 23 24 25 26 27 28 29 30 31
1
2
3
4
Group and Phase

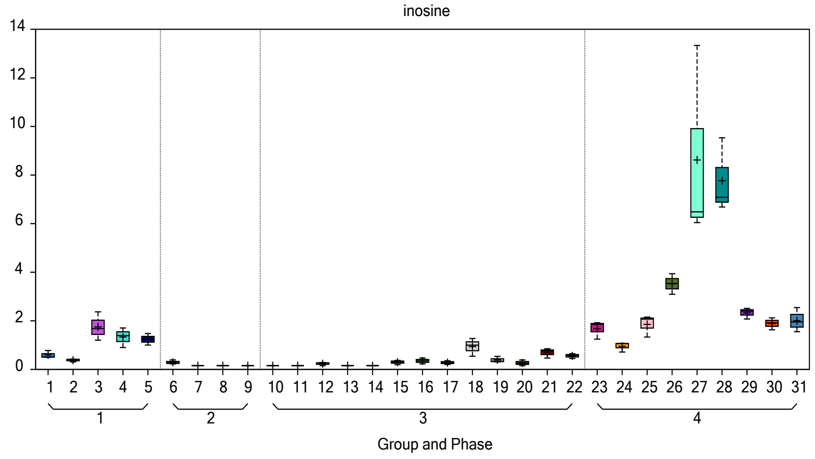
inosine
0 2 4 6 8 10 12 14
1 2 3 4 5 6 7 8 9 10 11 12 13 14 15 16 17 18 19 20 21 22 23 24 25 26 27 28 29 30 31
1 2 3 4
Group and Phase

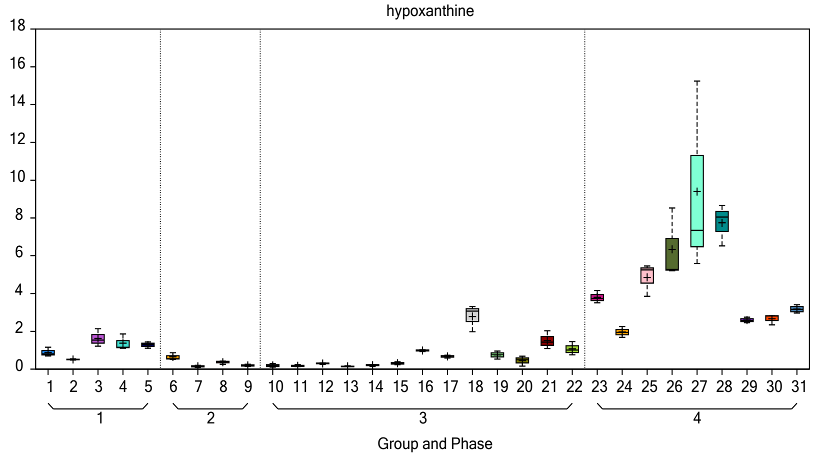
hypoxanthine
0 2 4 6 8 10 12 14 16 18
1 2 3 4 5 6 7 8 9 10 11 12 13 14 15 16 17 18 19 20 21 22 23 24 25 26 27 28 29 30 31
1 2 3 4
Group and Phase

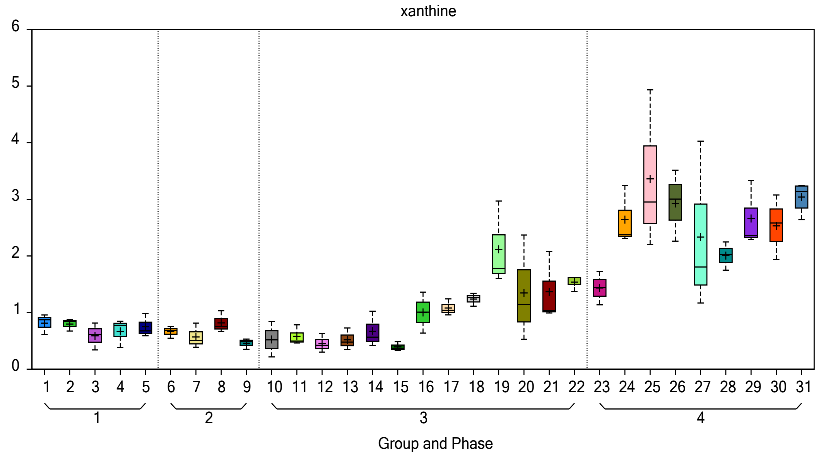
xanthine
0 1 2 3 4 5 6
1 2 3 4 5 6 7 8 9 10 11 12 13 14 15 16 17 18 19 20 21 22 23 24 25 26 27 28 29 30 31
1 2 3 4
Group and Phase

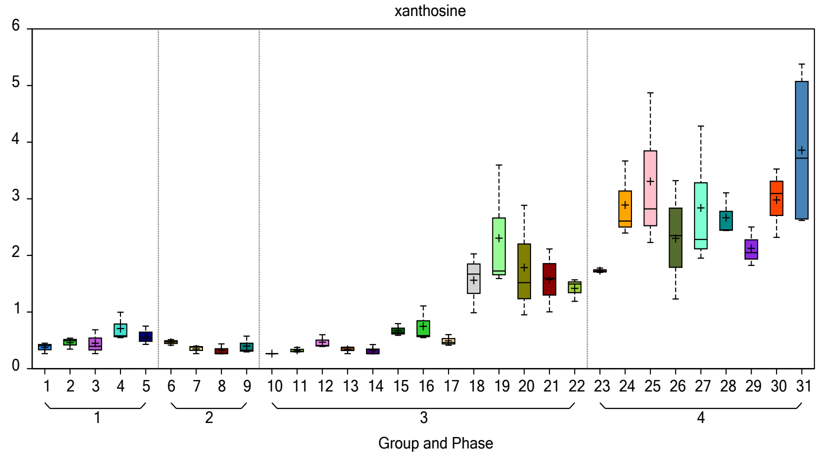
xanthosine
0 1 2 3 4 5 6
1 2 3 4 5 6 7 8 9 10 11 12 13 14 15 16 17 18 19 20 21 22 23 24 25 26 27 28 29 30 31
1 2 3 4
Group and Phase

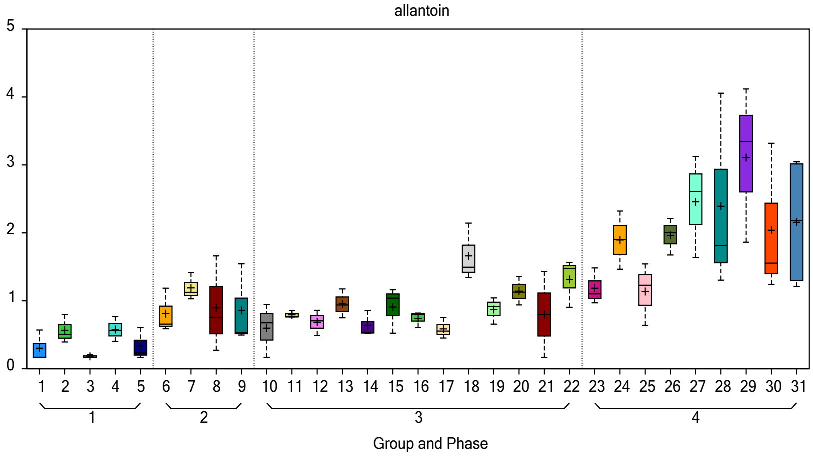
allantoin
Group and Phase

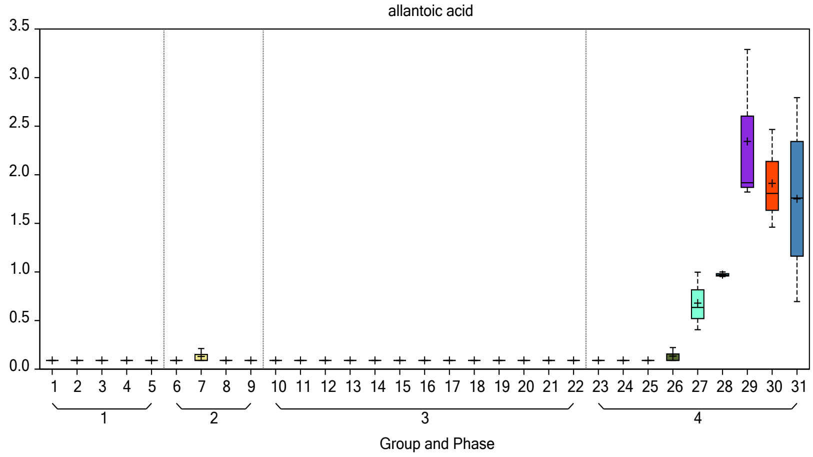
allantoic acid
Group and Phase

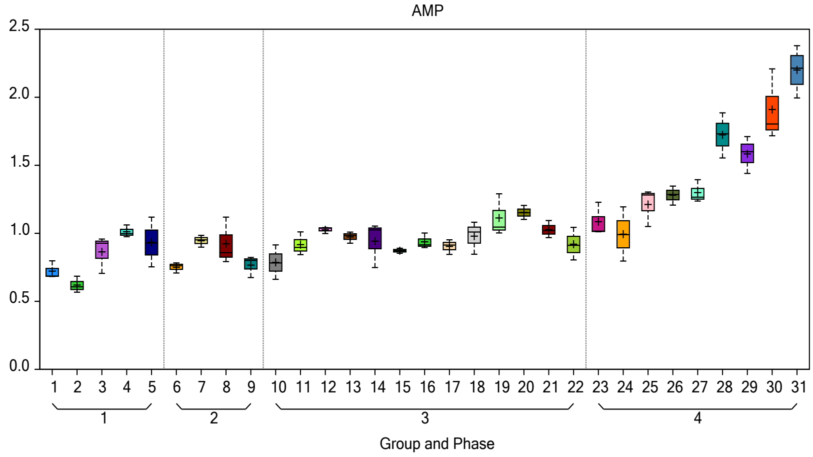
AMP
Group and Phase

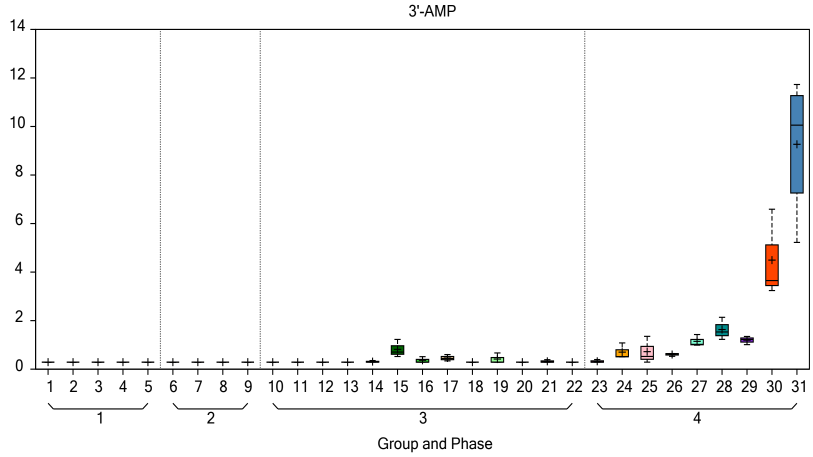
3'-AMP
Group and Phase

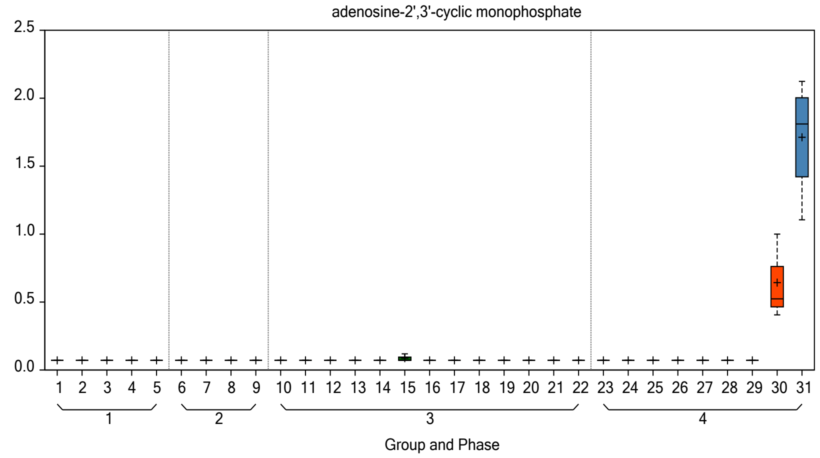
adenosine-2',3'-cyclic monophosphate
0.0
0.5
1.0
1.5
2.0
2.5
1 2 3 4 5 6 7 8 9 10 11 12 13 14 15 16 17 18 19 20 21 22 23 24 25 26 27 28 29 30 31
1
2
3
4
Group and Phase

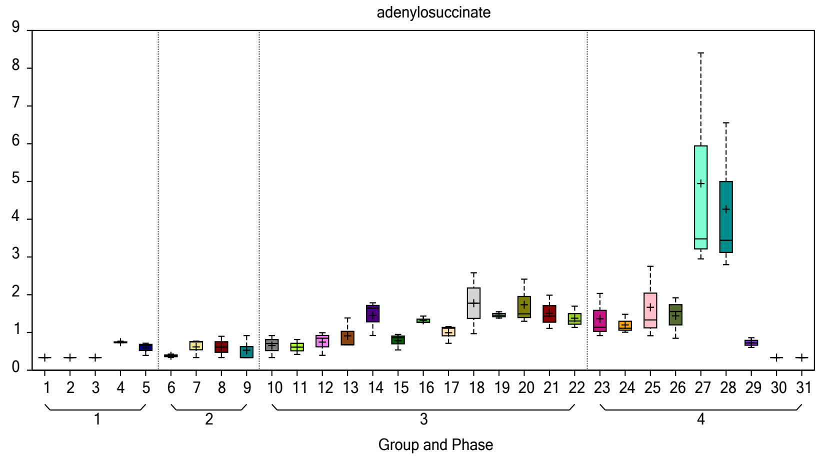
adenylosuccinate
0
1
2
3
4
5
6
7
8
9
1 2 3 4 5 6 7 8 9 10 11 12 13 14 15 16 17 18 19 20 21 22 23 24 25 26 27 28 29 30 31
1
2
3
4
Group and Phase

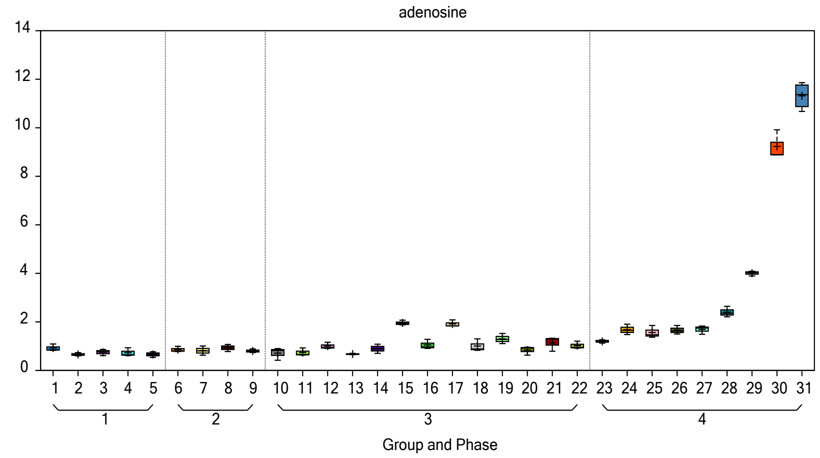
adenosine
0
2
4
6
8
10
12
14
1 2 3 4 5 6 7 8 9 10 11 12 13 14 15 16 17 18 19 20 21 22 23 24 25 26 27 28 29 30 31
1
2
3
4
Group and Phase

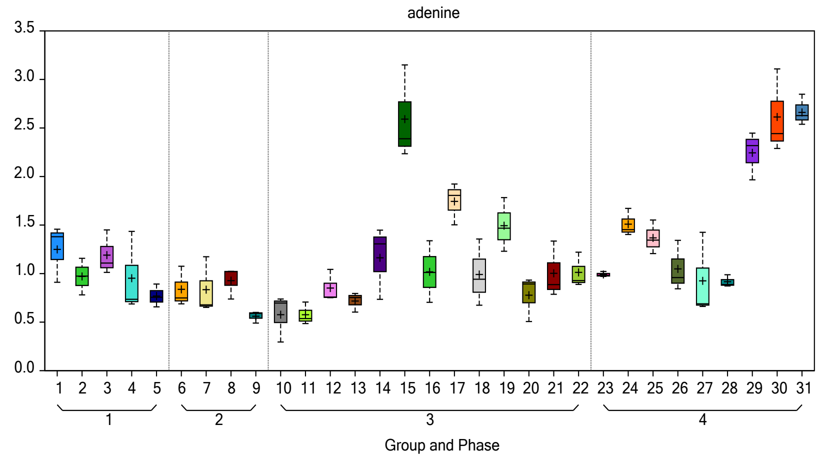
adenine
0.0
0.5
1.0
1.5
2.0
2.5
3.0
3.5
1 2 3 4 5 6 7 8 9 10 11 12 13 14 15 16 17 18 19 20 21 22 23 24 25 26 27 28 29 30 31
1
2
3
4
Group and Phase

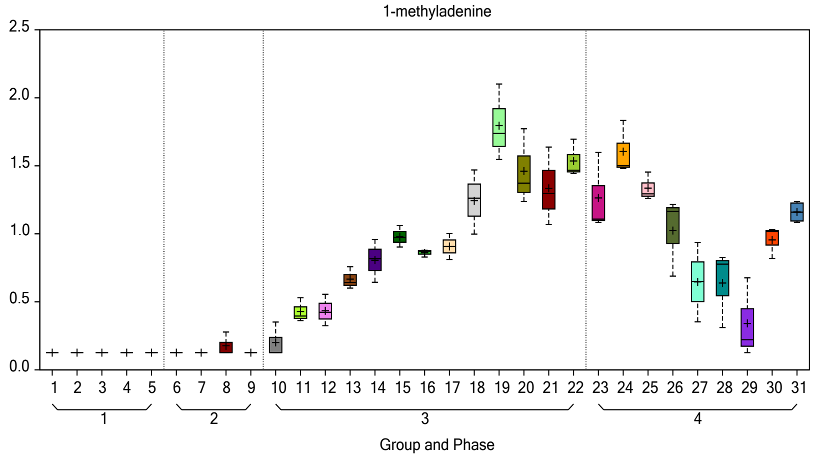
1-methyladenine
2.5
2.0
1.5
1.0
0.5
0.0
1 2 3 4 5 6 7 8 9 10 11 12 13 14 15 16 17 18 19 20 21 22 23 24 25 26 27 28 29 30 31
1
2
3
4
Group and Phase

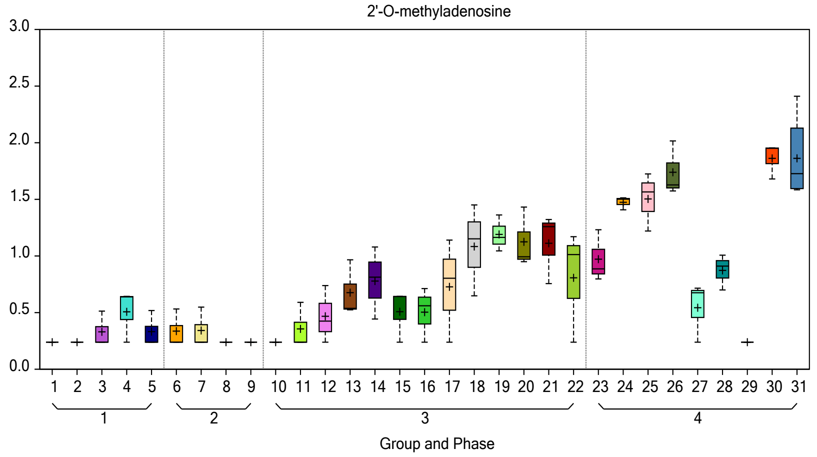
2'-O-methyladenosine
3.0
2.5
2.0
1.5
1.0
0.5
0.0
1 2 3 4 5 6 7 8 9 10 11 12 13 14 15 16 17 18 19 20 21 22 23 24 25 26 27 28 29 30 31
1
2
3
4
Group and Phase

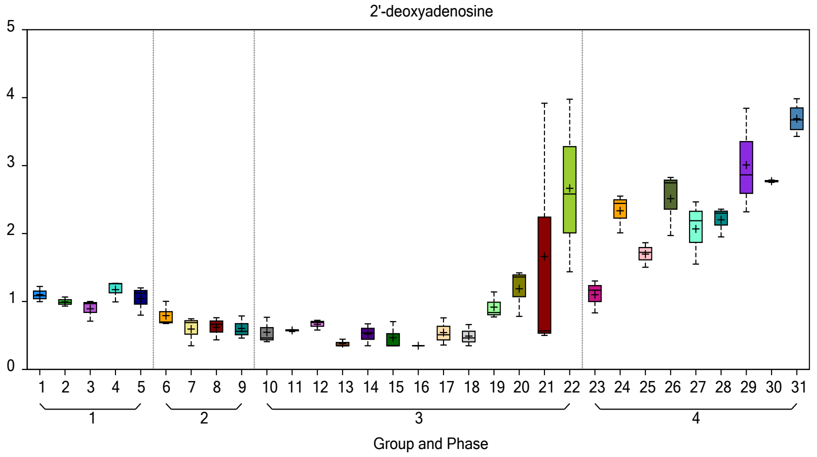
2'-deoxyadenosine
5
4
3
2
1
0
1 2 3 4 5 6 7 8 9 10 11 12 13 14 15 16 17 18 19 20 21 22 23 24 25 26 27 28 29 30 31
1
2
3
4
Group and Phase

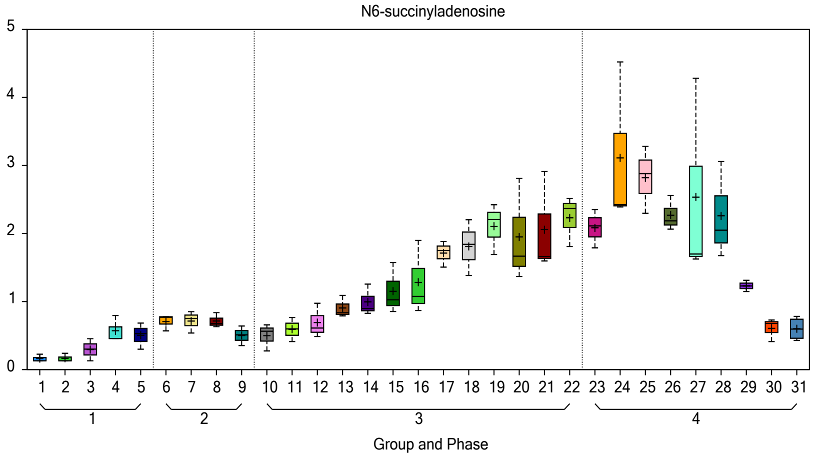
N6-succinyladenosine
5
4
3
2
1
0
1 2 3 4 5 6 7 8 9 10 11 12 13 14 15 16 17 18 19 20 21 22 23 24 25 26 27 28 29 30 31
1
2
3
4
Group and Phase

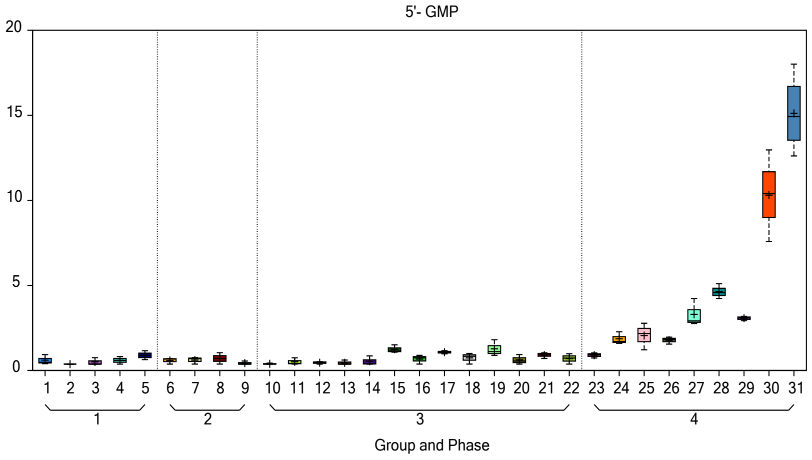
5'- GMP
0
5
10
15
20
1 2 3 4 5 6 7 8 9 10 11 12 13 14 15 16 17 18 19 20 21 22 23 24 25 26 27 28 29 30 31
1
2
3
4
Group and Phase

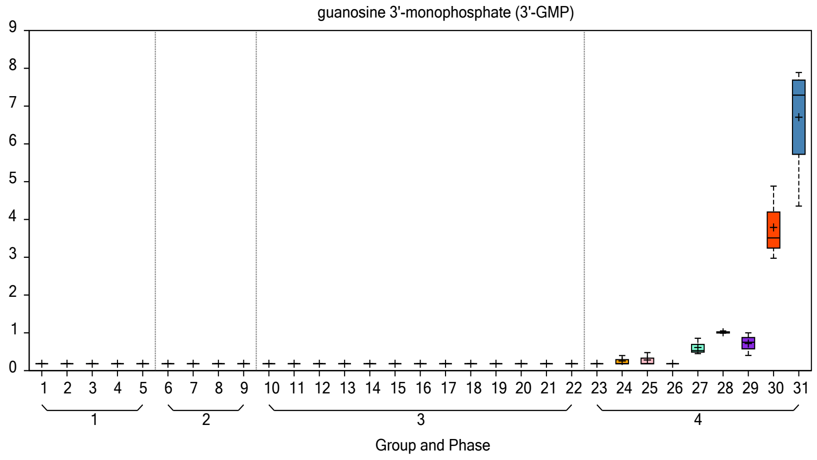
guanosine 3'-monophosphate (3'-GMP)
0
1
2
3
4
5
6
7
8
9
1 2 3 4 5 6 7 8 9 10 11 12 13 14 15 16 17 18 19 20 21 22 23 24 25 26 27 28 29 30 31
1
2
3
4
Group and Phase

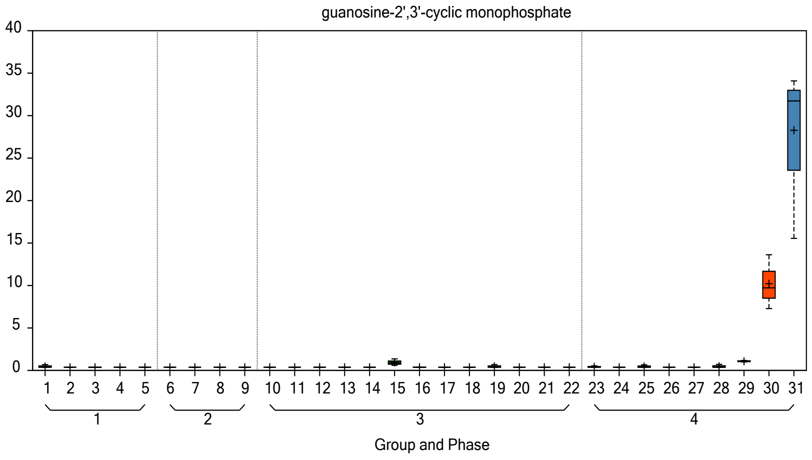
guanosine-2',3'-cyclic monophosphate
0
5
10
15
20
25
30
35
40
1 2 3 4 5 6 7 8 9 10 11 12 13 14 15 16 17 18 19 20 21 22 23 24 25 26 27 28 29 30 31
1
2
3
4
Group and Phase

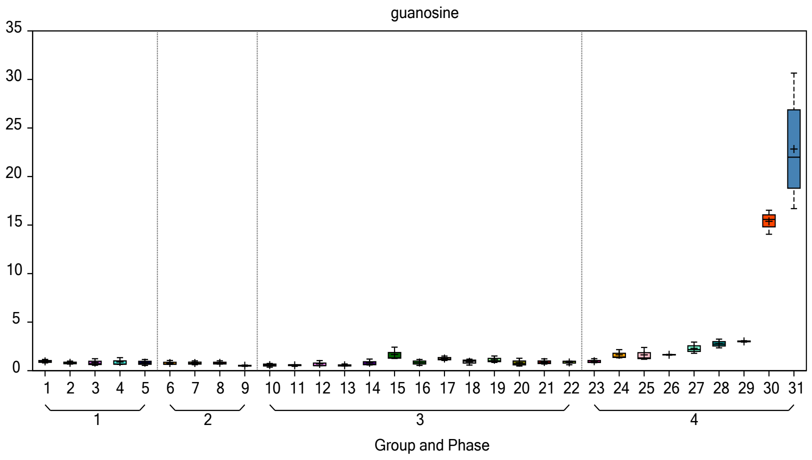
guanosine
0
5
10
15
20
25
30
35
1 2 3 4 5 6 7 8 9 10 11 12 13 14 15 16 17 18 19 20 21 22 23 24 25 26 27 28 29 30 31
1
2
3
4
Group and Phase

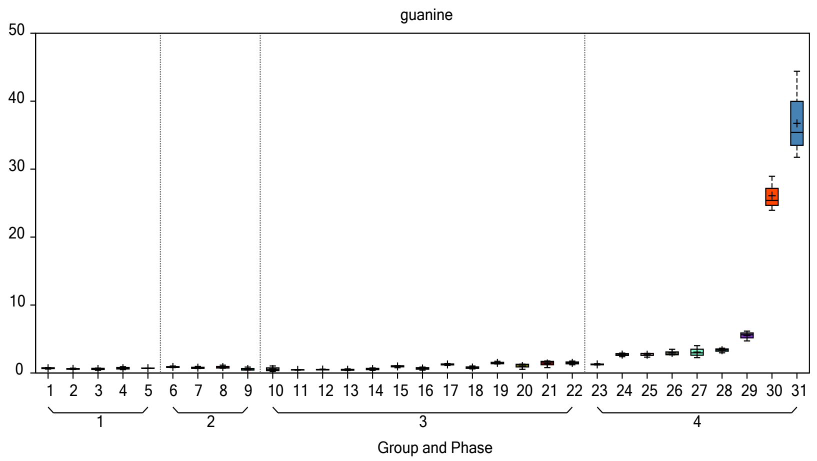
guanine
0
10
20
30
40
50
1 2 3 4 5 6 7 8 9 10 11 12 13 14 15 16 17 18 19 20 21 22 23 24 25 26 27 28 29 30 31
1
2
3
4
Group and Phase

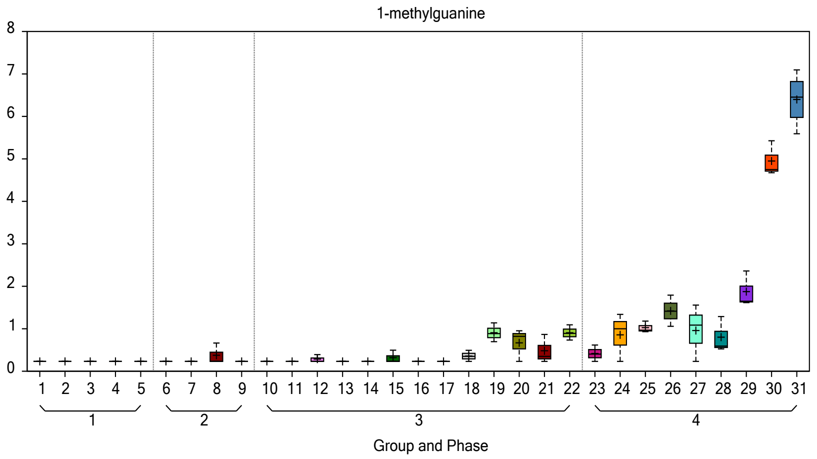
1-methylguanine
0
1
2
3
4
5
6
7
8
1 2 3 4 5 6 7 8 9 10 11 12 13 14 15 16 17 18 19 20 21 22 23 24 25 26 27 28 29 30 31
1
2
3
4
Group and Phase

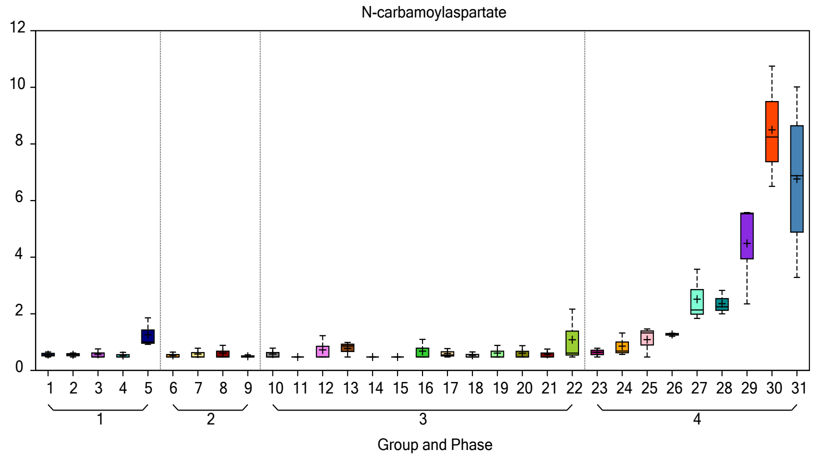
N-carbamoylaspartate
0
2
4
6
8
10
12
1 2 3 4 5 6 7 8 9 10 11 12 13 14 15 16 17 18 19 20 21 22 23 24 25 26 27 28 29 30 31
1
2
3
4
Group and Phase

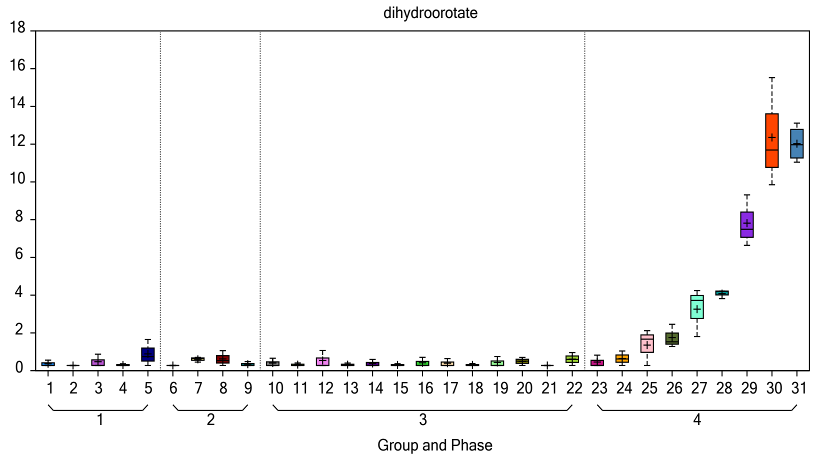
dihydroorotate
0
2
4
6
8
10
12
14
16
18
1 2 3 4 5 6 7 8 9 10 11 12 13 14 15 16 17 18 19 20 21 22 23 24 25 26 27 28 29 30 31
1
2
3
4
Group and Phase

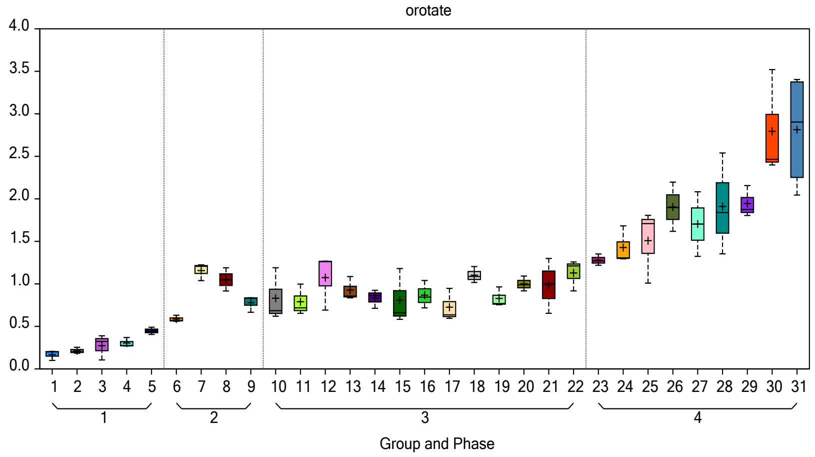
orotate
4.0
3.5
3.0
2.5
2.0
1.5
1.0
0.5
0.0
1 2 3 4 5 6 7 8 9 10 11 12 13 14 15 16 17 18 19 20 21 22 23 24 25 26 27 28 29 30 31
1
2
3
4
Group and Phase

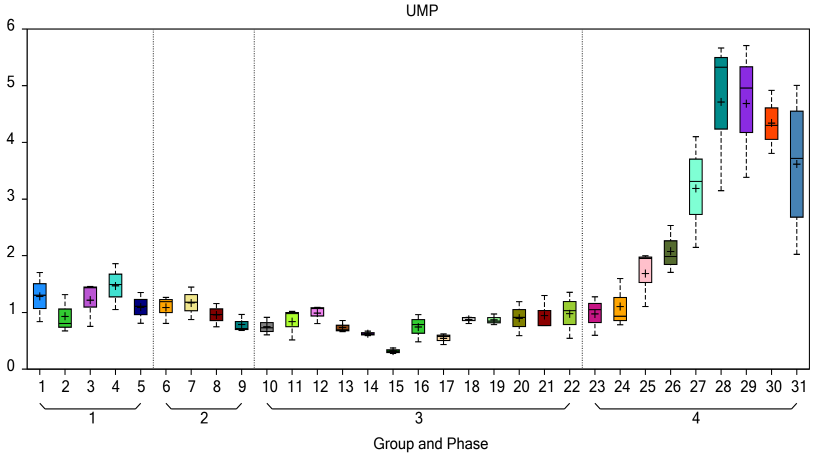
UMP
6
5
4
3
2
1
0
1 2 3 4 5 6 7 8 9 10 11 12 13 14 15 16 17 18 19 20 21 22 23 24 25 26 27 28 29 30 31
1
2
3
4
Group and Phase

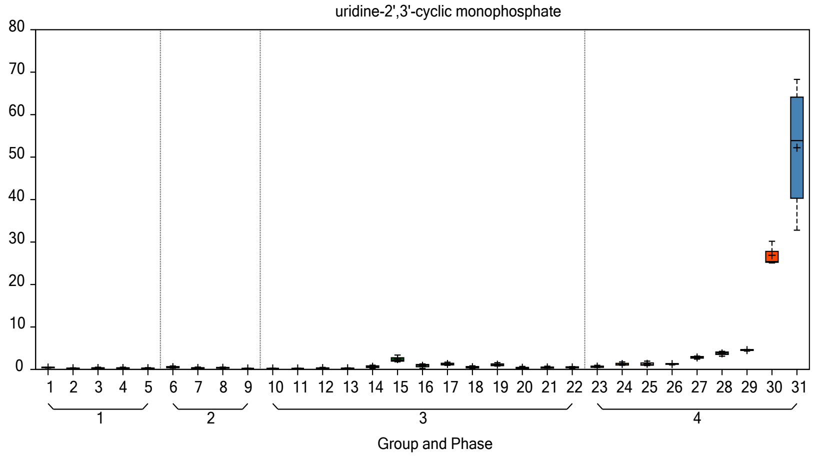
uridine-2',3'-cyclic monophosphate
80
70
60
50
40
30
20
10
0
1 2 3 4 5 6 7 8 9 10 11 12 13 14 15 16 17 18 19 20 21 22 23 24 25 26 27 28 29 30 31
1
2
3
4
Group and Phase

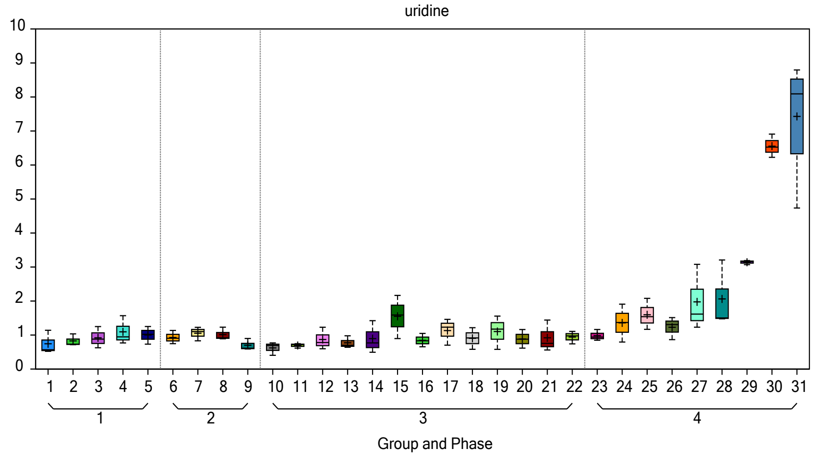
uridine
10
9
8
7
6
5
4
3
2
1
0
1 2 3 4 5 6 7 8 9 10 11 12 13 14 15 16 17 18 19 20 21 22 23 24 25 26 27 28 29 30 31
1
2
3
4
Group and Phase

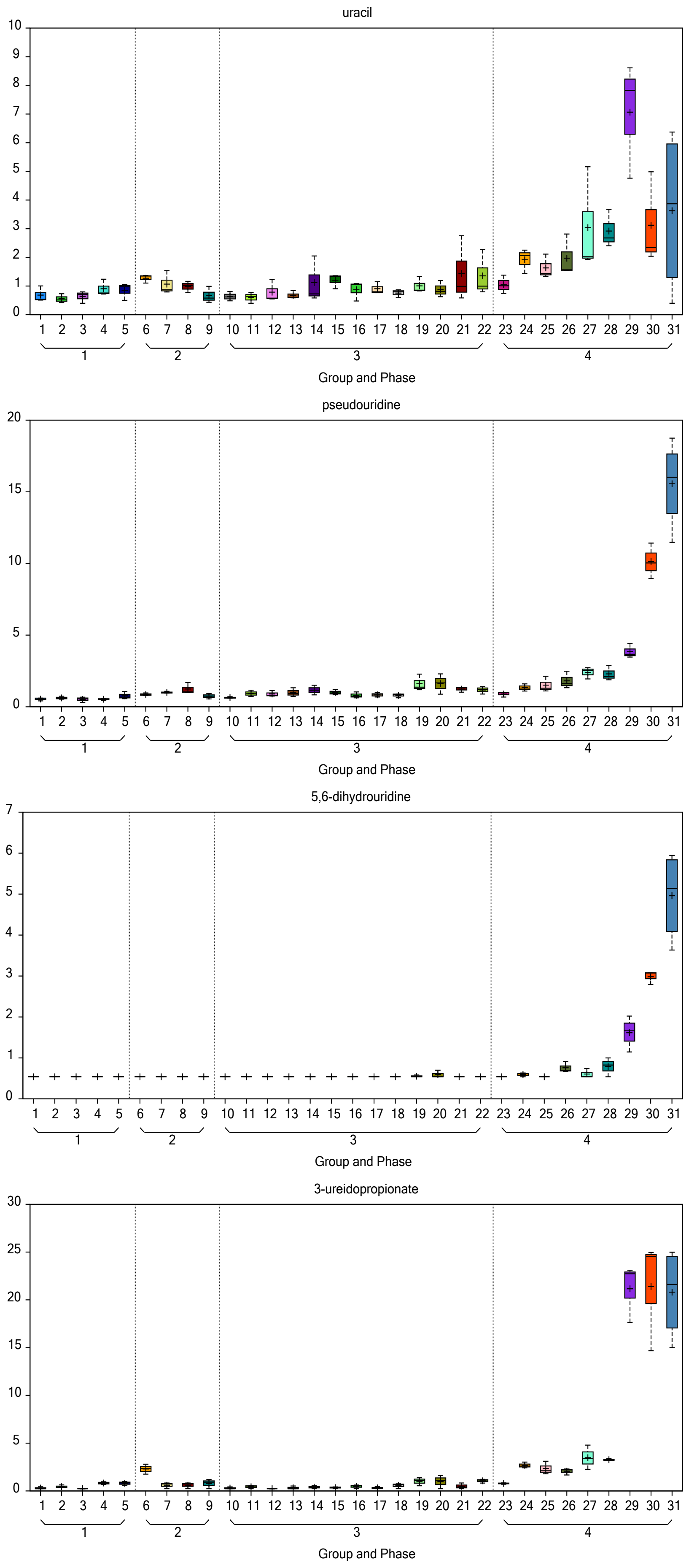

uracil
pseudouridine
5,6-dihydrouridine
3-ureidopropionate
Group and Phase

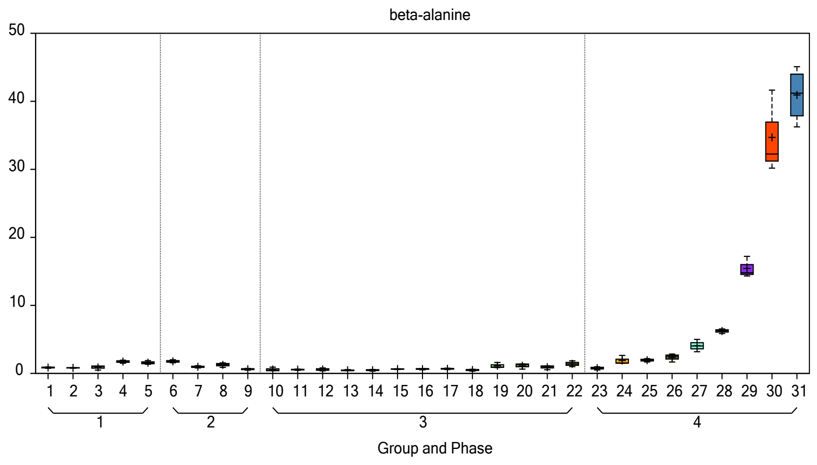
beta-alanine
Group and Phase

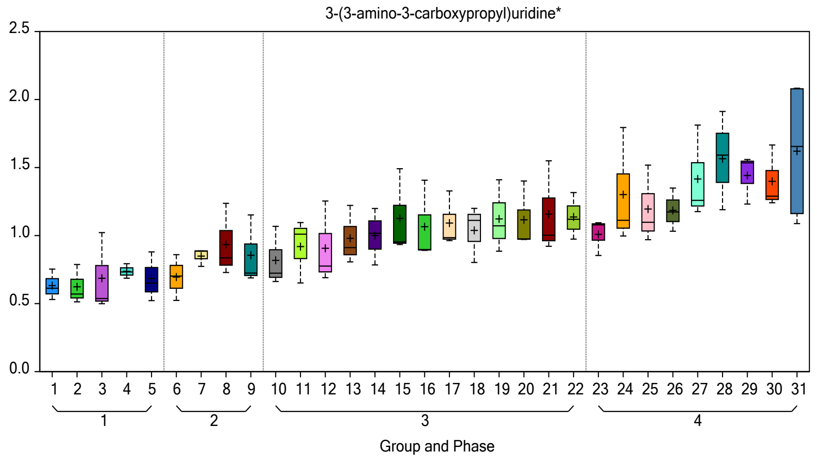
3-(3-amino-3-carboxypropyl)uridine*
Group and Phase

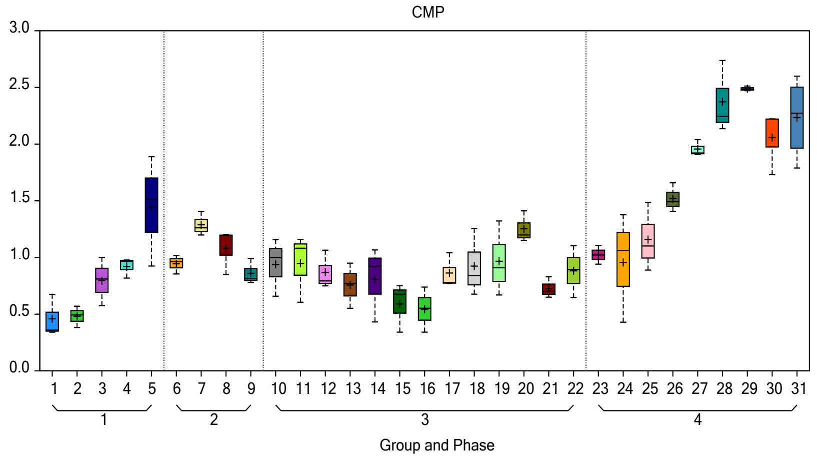
CMP
Group and Phase

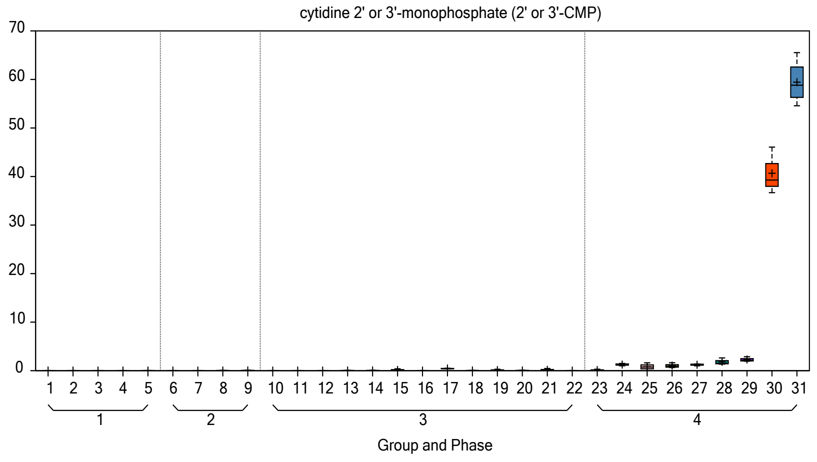
cytidine 2' or 3'-monophosphate (2' or 3'-CMP)
Group and Phase

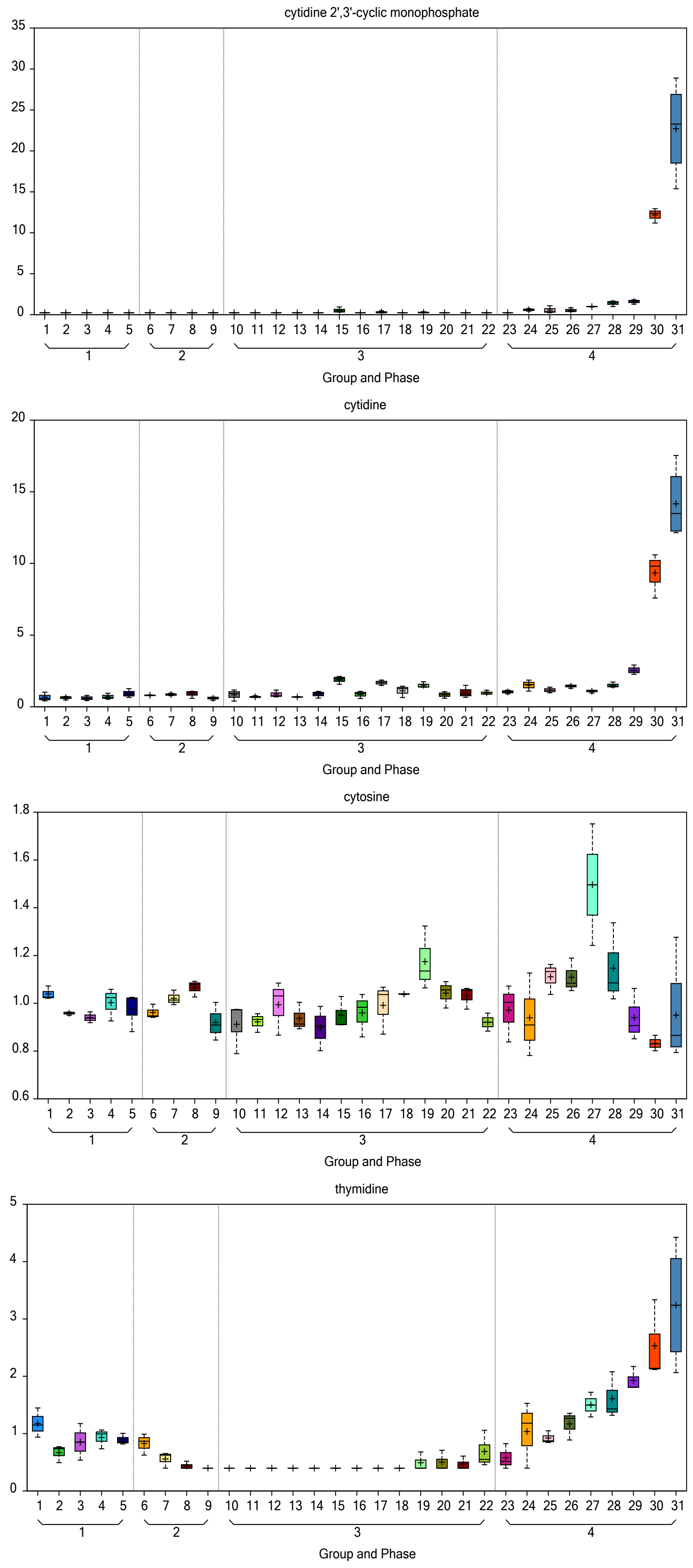

cytidine 2',3'-cyclic monophosphate
Group and Phase
cytidine
Group and Phase
cytosine
Group and Phase
thymidine
Group and Phase

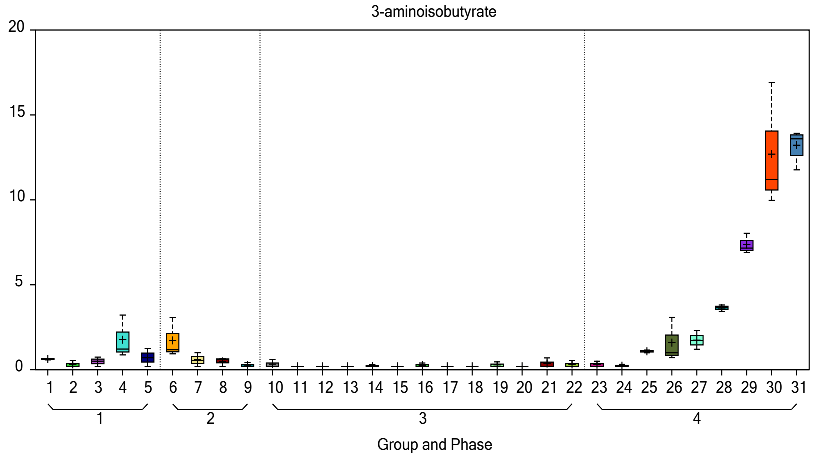

3-aminoisobutyrate
0
5
10
15
20
1 2 3 4 5 6 7 8 9 10 11 12 13 14 15 16 17 18 19 20 21 22 23 24 25 26 27 28 29 30 31
1
2
3
4
Group and Phase

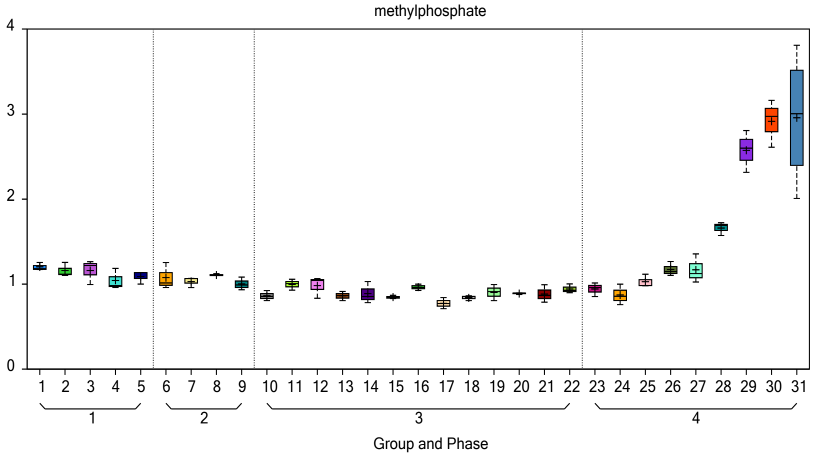

methylphosphate
0
1
2
3
4
1 2 3 4 5 6 7 8 9 10 11 12 13 14 15 16 17 18 19 20 21 22 23 24 25 26 27 28 29 30 31
1
2
3
4
Group and Phase

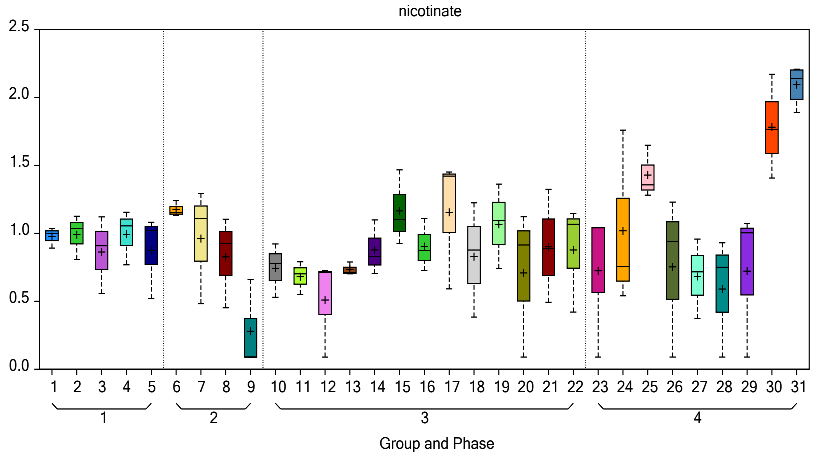

nicotinate
0.0
0.5
1.0
1.5
2.0
2.5
1 2 3 4 5 6 7 8 9 10 11 12 13 14 15 16 17 18 19 20 21 22 23 24 25 26 27 28 29 30 31
1
2
3
4
Group and Phase

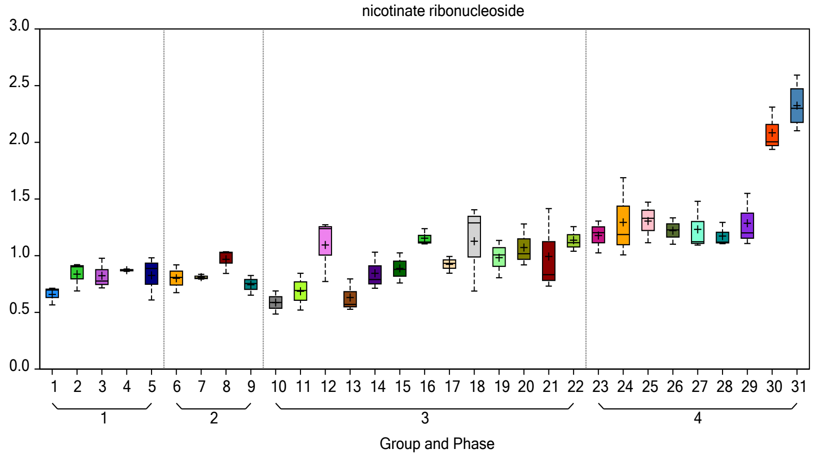

nicotinate ribonucleoside
0.0
0.5
1.0
1.5
2.0
2.5
3.0
1 2 3 4 5 6 7 8 9 10 11 12 13 14 15 16 17 18 19 20 21 22 23 24 25 26 27 28 29 30 31
1
2
3
4
Group and Phase

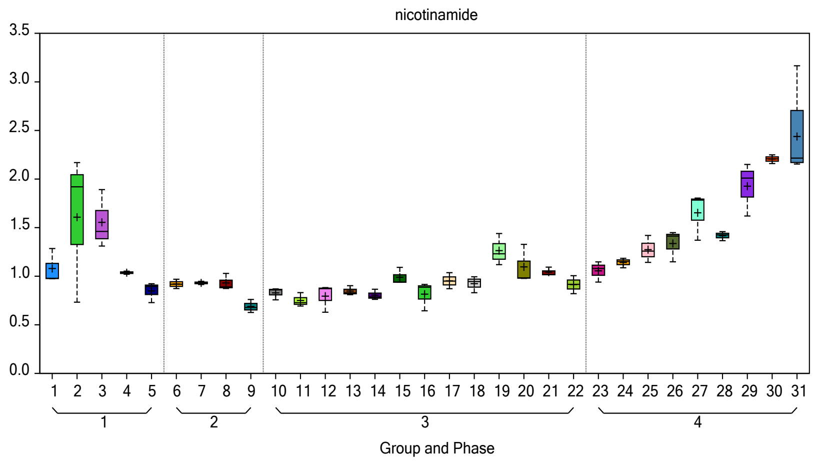
nicotinamide
Group and Phase

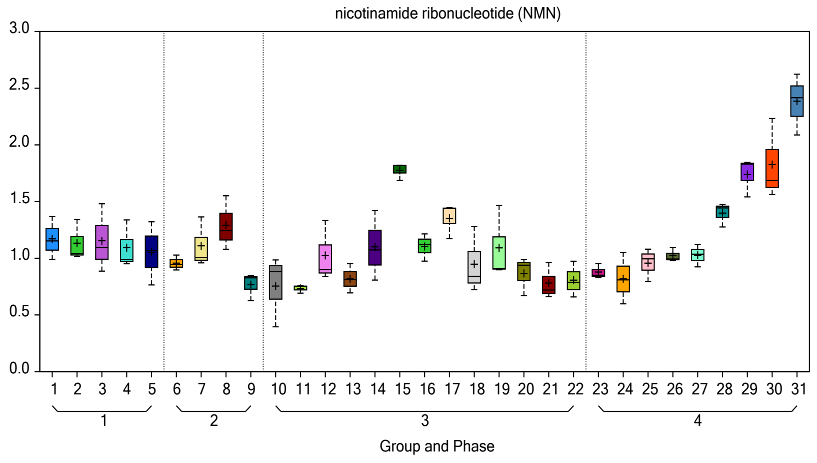
nicotinamide ribonucleotide (NMN)
Group and Phase

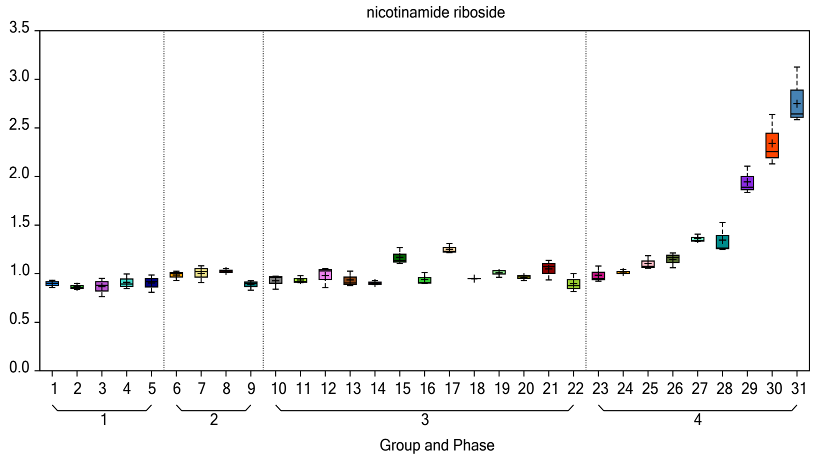
nicotinamide riboside
Group and Phase

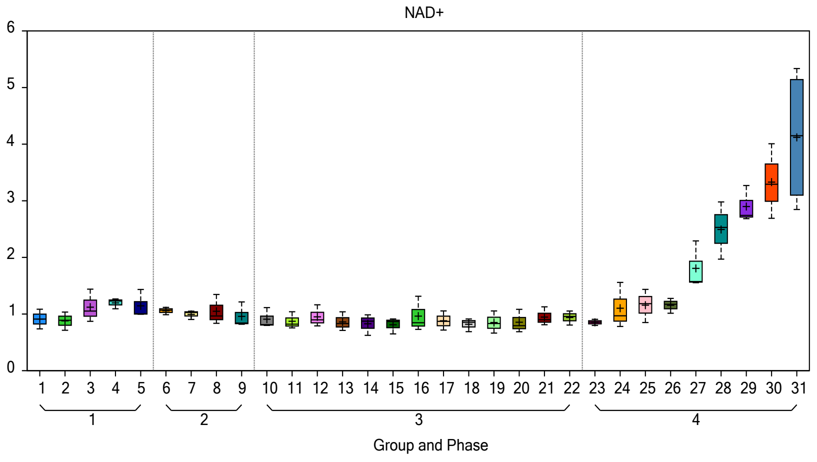
NAD+
Group and Phase

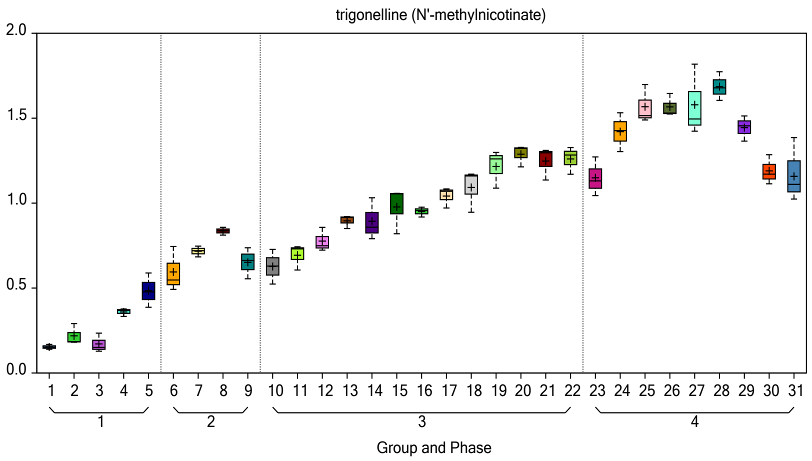
trigonelline (N'-methylnicotinate)
0.0
0.5
1.0
1.5
2.0
1 2 3 4 5 6 7 8 9 10 11 12 13 14 15 16 17 18 19 20 21 22 23 24 25 26 27 28 29 30 31
1
2
3
4
Group and Phase

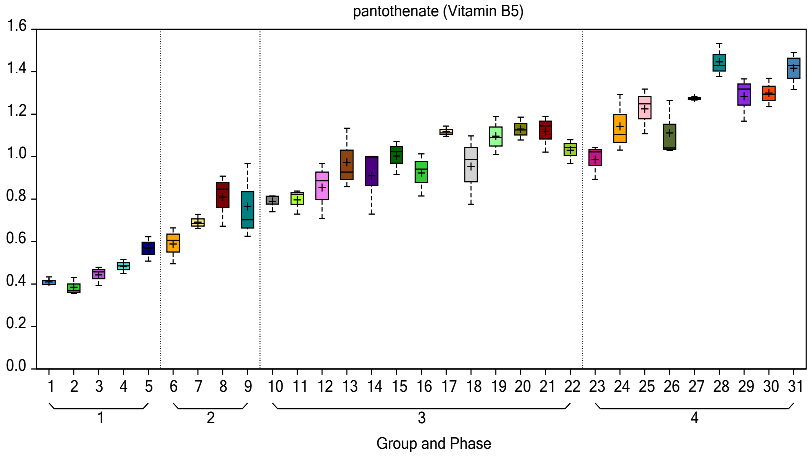
pantothenate (Vitamin B5)
0.0
0.2
0.4
0.6
0.8
1.0
1.2
1.4
1.6
1 2 3 4 5 6 7 8 9 10 11 12 13 14 15 16 17 18 19 20 21 22 23 24 25 26 27 28 29 30 31
1
2
3
4
Group and Phase

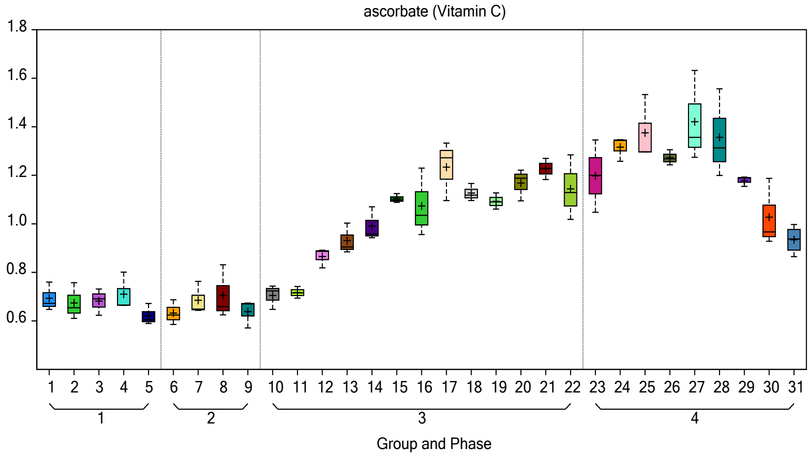
ascorbate (Vitamin C)
0.6
0.8
1.0
1.2
1.4
1.6
1.8
1 2 3 4 5 6 7 8 9 10 11 12 13 14 15 16 17 18 19 20 21 22 23 24 25 26 27 28 29 30 31
1
2
3
4
Group and Phase

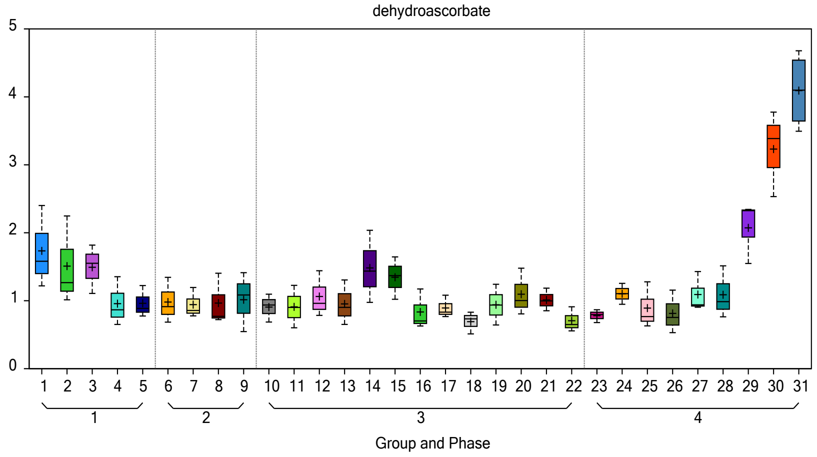
dehydroascorbate
0
1
2
3
4
5
1 2 3 4 5 6 7 8 9 10 11 12 13 14 15 16 17 18 19 20 21 22 23 24 25 26 27 28 29 30 31
1
2
3
4
Group and Phase

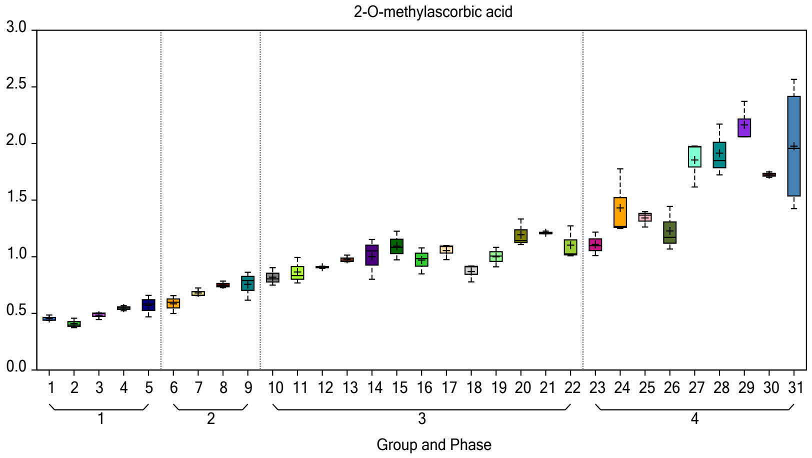
2-O-methylascorbic acid
3.0
2.5
2.0
1.5
1.0
0.5
0.0
1 2 3 4 5 6 7 8 9 10 11 12 13 14 15 16 17 18 19 20 21 22 23 24 25 26 27 28 29 30 31
1
2
3
4
Group and Phase

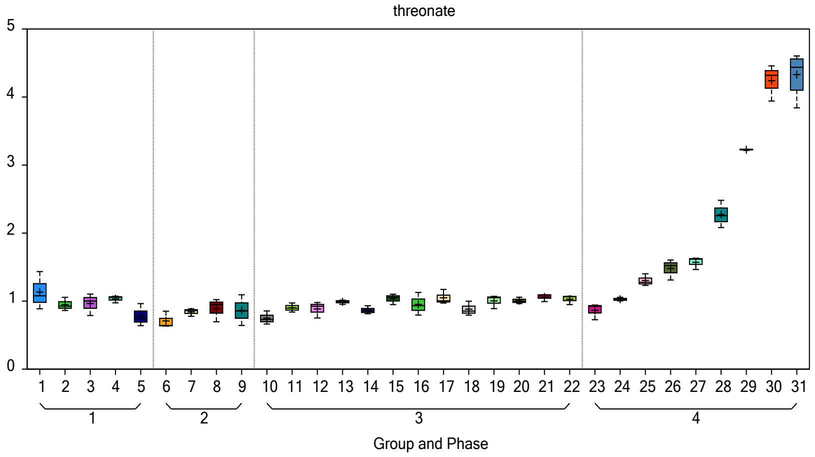
threonate
5
4
3
2
1
0
1 2 3 4 5 6 7 8 9 10 11 12 13 14 15 16 17 18 19 20 21 22 23 24 25 26 27 28 29 30 31
1
2
3
4
Group and Phase

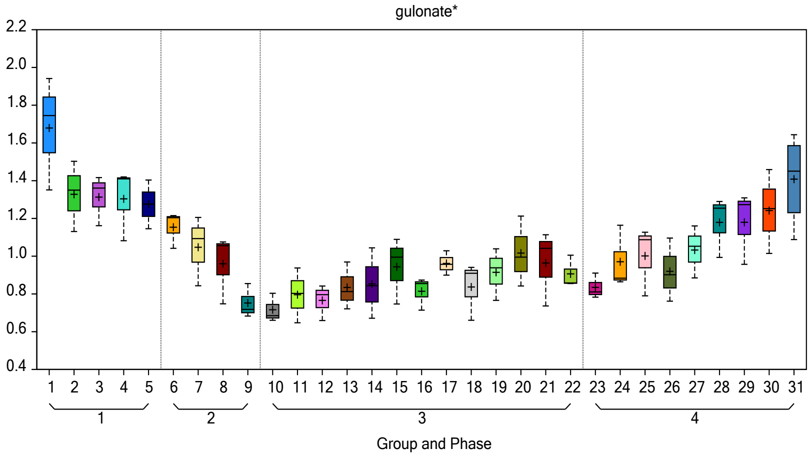
gulonate*
2.2
2.0
1.8
1.6
1.4
1.2
1.0
0.8
0.6
0.4
1 2 3 4 5 6 7 8 9 10 11 12 13 14 15 16 17 18 19 20 21 22 23 24 25 26 27 28 29 30 31
1
2
3
4
Group and Phase

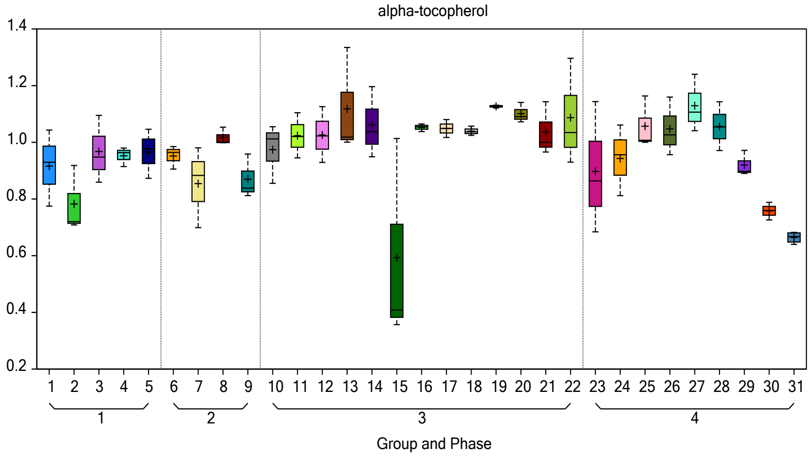
alpha-tocopherol
1.4
1.2
1.0
0.8
0.6
0.4
0.2
1 2 3 4 5 6 7 8 9 10 11 12 13 14 15 16 17 18 19 20 21 22 23 24 25 26 27 28 29 30 31
1
2
3
4
Group and Phase

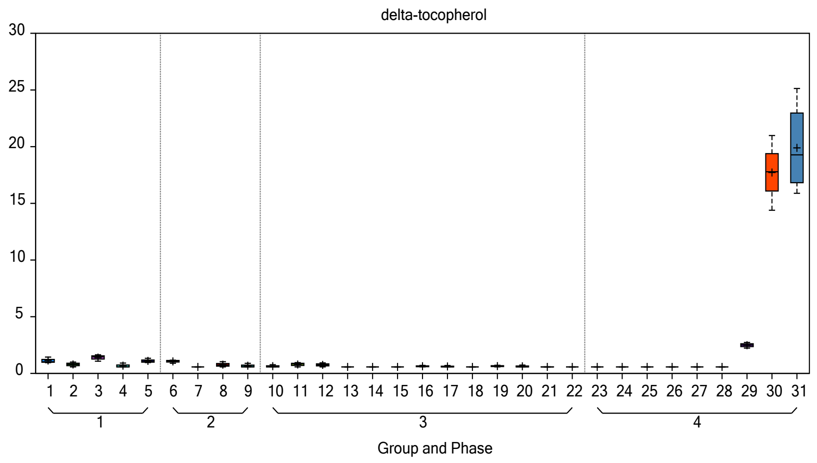
delta-tocopherol
30
25
20
15
10
5
0
1 2 3 4 5 6 7 8 9 10 11 12 13 14 15 16 17 18 19 20 21 22 23 24 25 26 27 28 29 30 31
1
2
3
4
Group and Phase

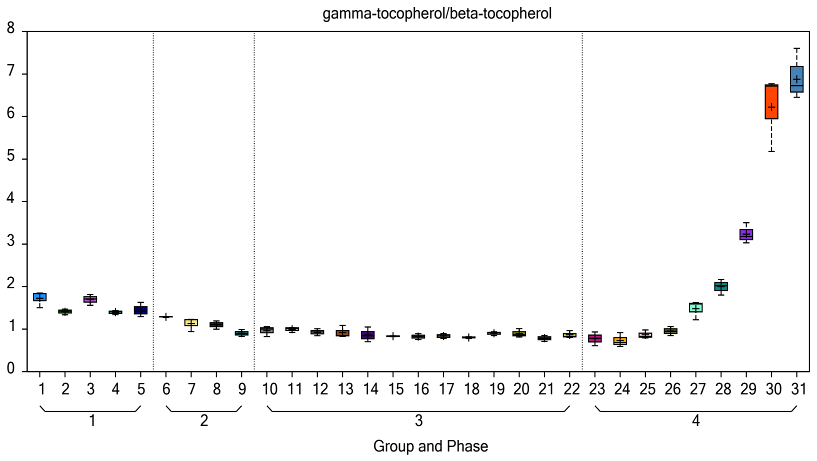
gamma-tocopherol/beta-tocopherol
8
7
6
5
4
3
2
1
0
1 2 3 4 5 6 7 8 9 10 11 12 13 14 15 16 17 18 19 20 21 22 23 24 25 26 27 28 29 30 31
1
2
3
4
Group and Phase

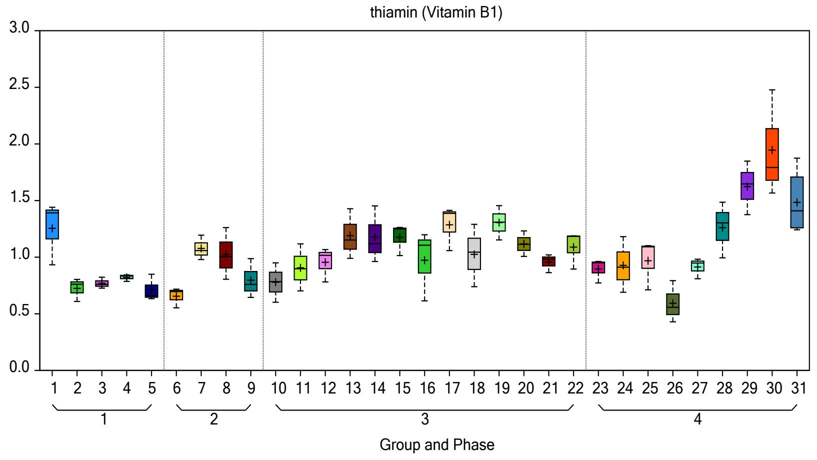
thiamin (Vitamin B1)
3.0
2.5
2.0
1.5
1.0
0.5
0.0
1 2 3 4 5 6 7 8 9 10 11 12 13 14 15 16 17 18 19 20 21 22 23 24 25 26 27 28 29 30 31
1
2
3
4
Group and Phase

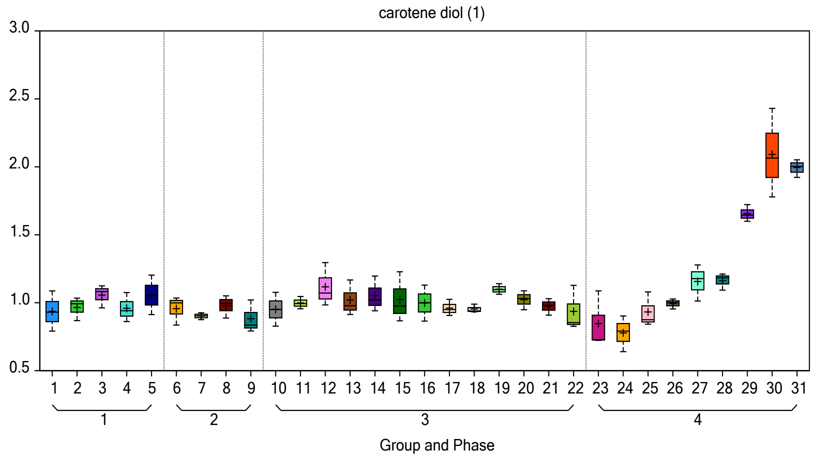
carotene diol (1)
3.0
2.5
2.0
1.5
1.0
0.5
1 2 3 4 5 6 7 8 9 10 11 12 13 14 15 16 17 18 19 20 21 22 23 24 25 26 27 28 29 30 31
1
2
3
4
Group and Phase

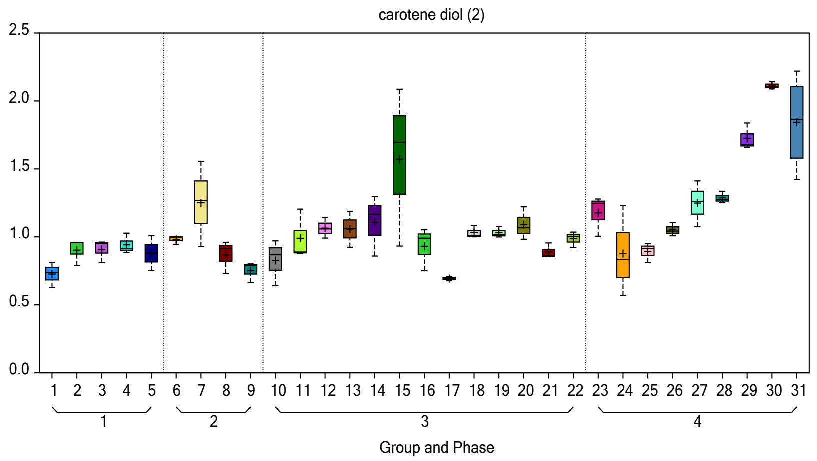
carotene diol (2)
2.5
2.0
1.5
1.0
0.5
0.0
1 2 3 4 5 6 7 8 9 10 11 12 13 14 15 16 17 18 19 20 21 22 23 24 25 26 27 28 29 30 31
1
2
3
4
Group and Phase

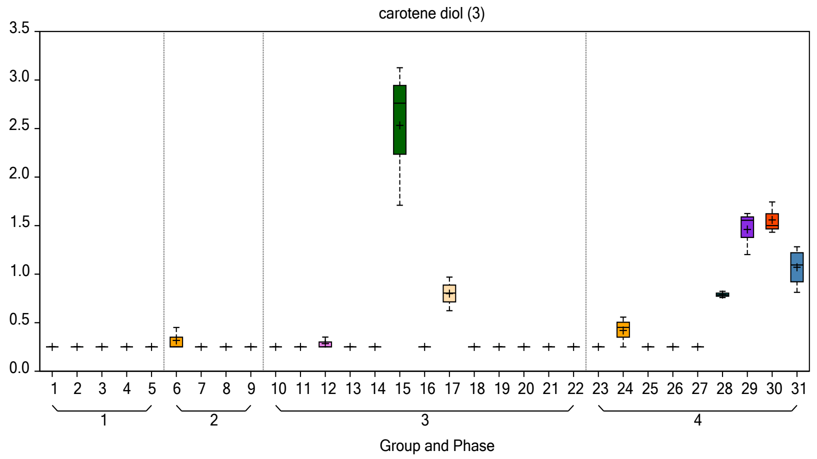
carotene diol (3)
3.5
3.0
2.5
2.0
1.5
1.0
0.5
0.0
1 2 3 4 5 6 7 8 9 10 11 12 13 14 15 16 17 18 19 20 21 22 23 24 25 26 27 28 29 30 31
1
2
3
4
Group and Phase

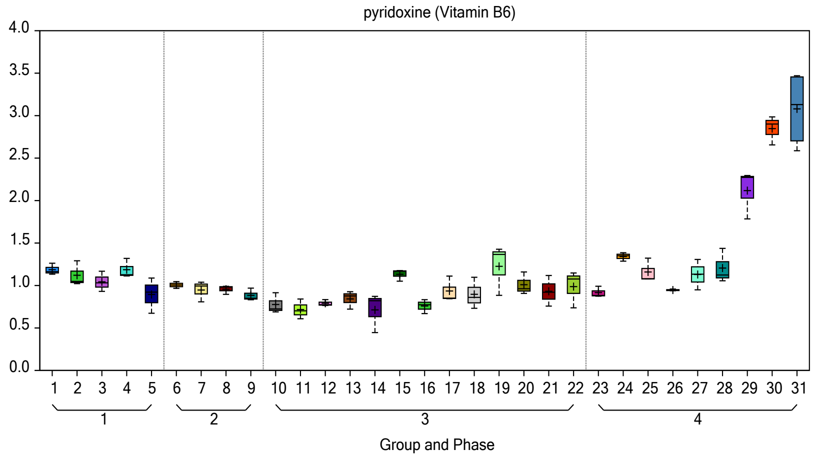
pyridoxine (Vitamin B6)
4.0
3.5
3.0
2.5
2.0
1.5
1.0
0.5
0.0
1 2 3 4 5 6 7 8 9 10 11 12 13 14 15 16 17 18 19 20 21 22 23 24 25 26 27 28 29 30 31
1
2
3
4
Group and Phase

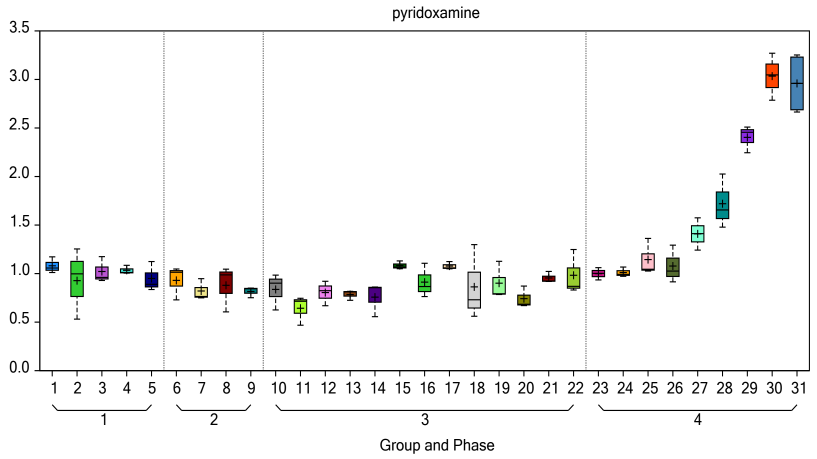
pyridoxamine
3.5
3.0
2.5
2.0
1.5
1.0
0.5
0.0
1 2 3 4 5 6 7 8 9 10 11 12 13 14 15 16 17 18 19 20 21 22 23 24 25 26 27 28 29 30 31
1
2
3
4
Group and Phase

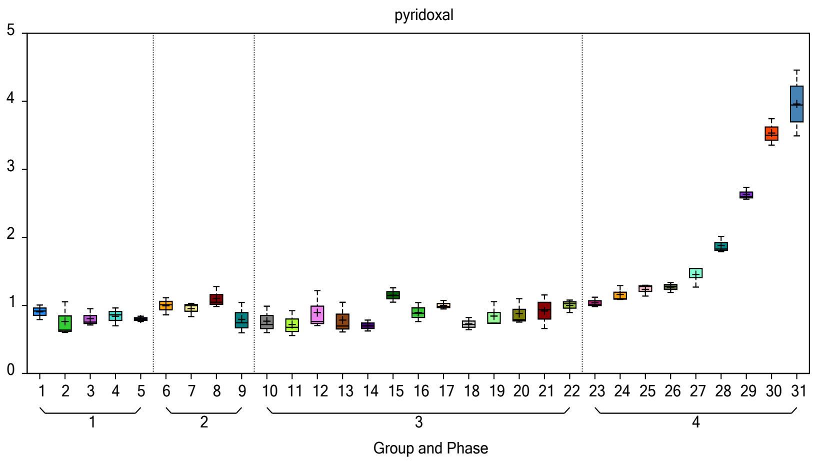

pyridoxal
0
1
2
3
4
5
1 2 3 4 5 6 7 8 9 10 11 12 13 14 15 16 17 18 19 20 21 22 23 24 25 26 27 28 29 30 31
1
2
3
4
Group and Phase

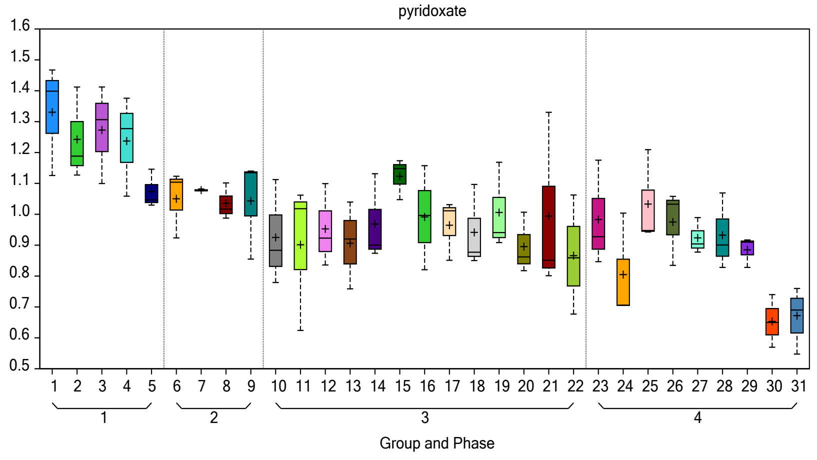

pyridoxate
0.5
0.6
0.7
0.8
0.9
1.0
1.1
1.2
1.3
1.4
1.5
1.6
1 2 3 4 5 6 7 8 9 10 11 12 13 14 15 16 17 18 19 20 21 22 23 24 25 26 27 28 29 30 31
1
2
3
4
Group and Phase

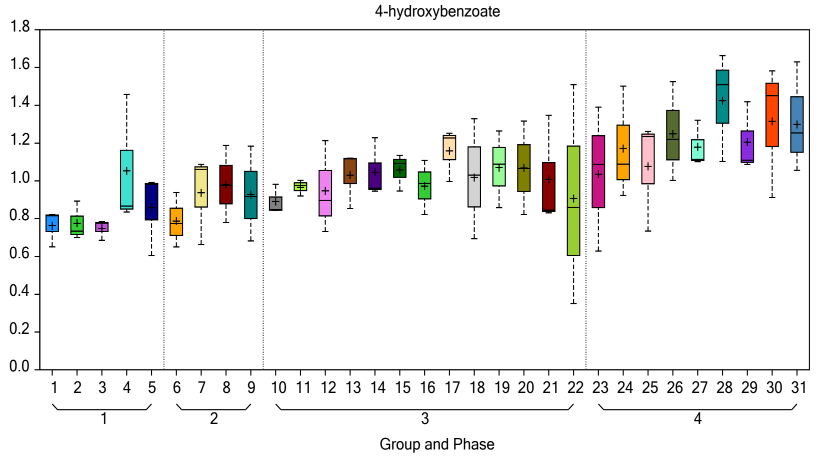

4-hydroxybenzoate
0.0
0.2
0.4
0.6
0.8
1.0
1.2
1.4
1.6
1.8
1 2 3 4 5 6 7 8 9 10 11 12 13 14 15 16 17 18 19 20 21 22 23 24 25 26 27 28 29 30 31
1
2
3
4
Group and Phase

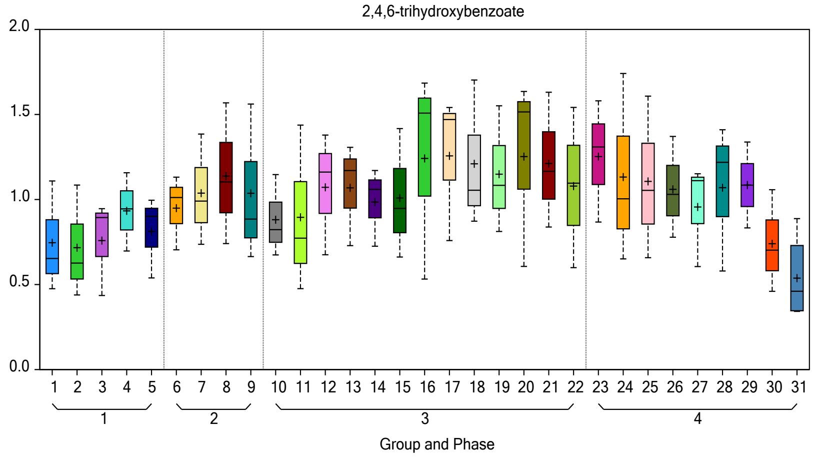

2,4,6-trihydroxybenzoate
0.0
0.5
1.0
1.5
2.0
1 2 3 4 5 6 7 8 9 10 11 12 13 14 15 16 17 18 19 20 21 22 23 24 25 26 27 28 29 30 31
1
2
3
4
Group and Phase

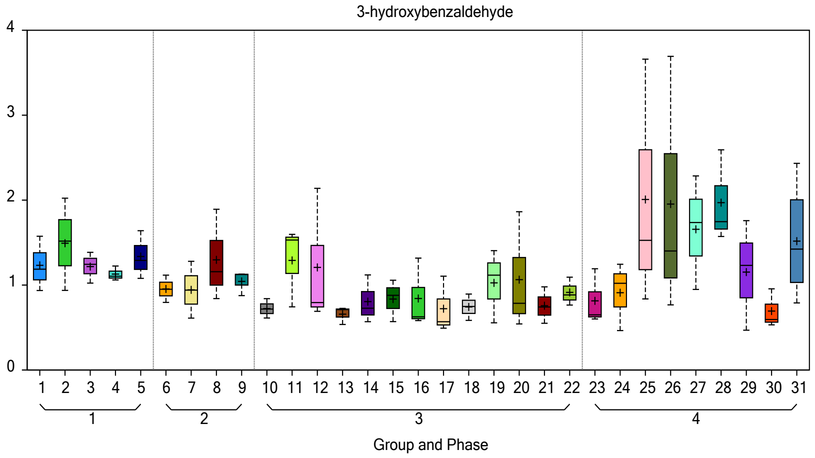
3-hydroxybenzaldehyde
Group and Phase

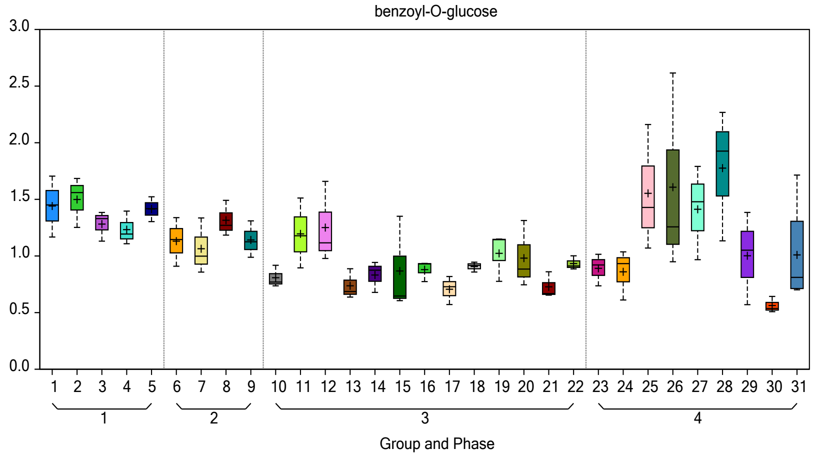
benzoyl-O-glucose
Group and Phase

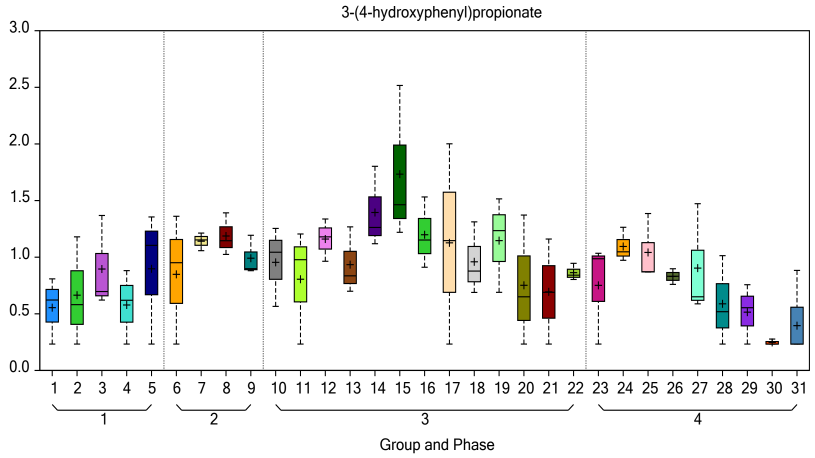
3-(4-hydroxyphenyl)propionate
Group and Phase

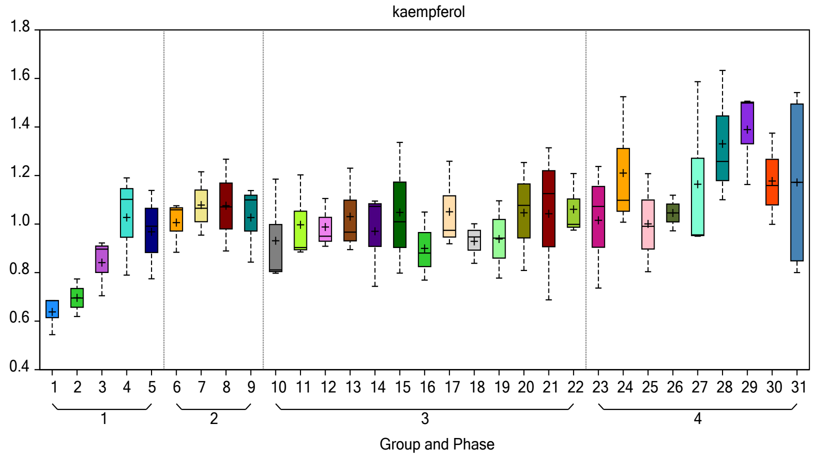
kaempferol
Group and Phase

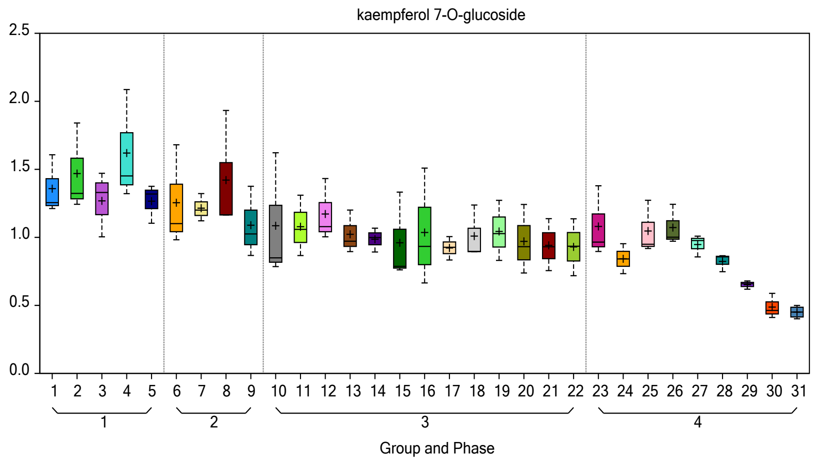
kaempferol 7-O-glucoside
2.5
2.0
1.5
1.0
0.5
0.0
1 2 3 4 5 6 7 8 9 10 11 12 13 14 15 16 17 18 19 20 21 22 23 24 25 26 27 28 29 30 31
1
2
3
4
Group and Phase

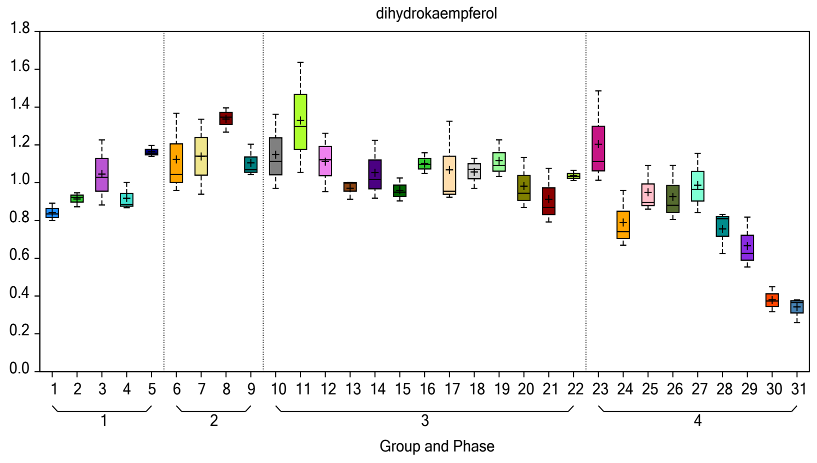
dihydrokaempferol
1.8
1.6
1.4
1.2
1.0
0.8
0.6
0.4
0.2
0.0
1 2 3 4 5 6 7 8 9 10 11 12 13 14 15 16 17 18 19 20 21 22 23 24 25 26 27 28 29 30 31
1
2
3
4
Group and Phase

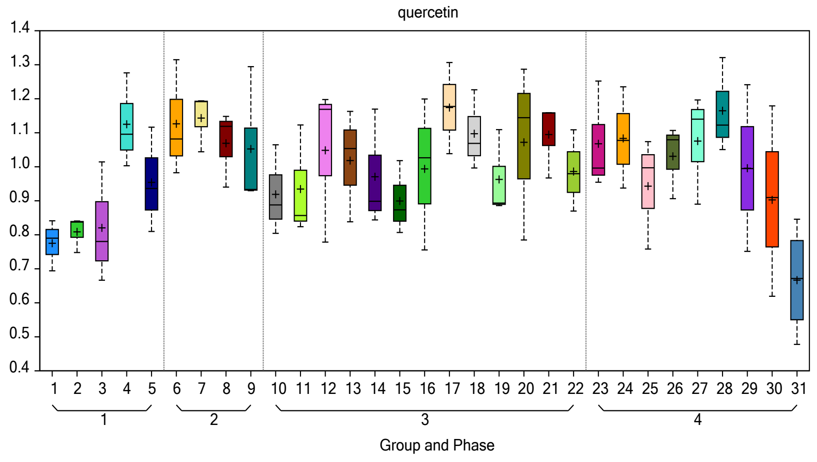
quercetin
1.4
1.3
1.2
1.1
1.0
0.9
0.8
0.7
0.6
0.5
0.4
1 2 3 4 5 6 7 8 9 10 11 12 13 14 15 16 17 18 19 20 21 22 23 24 25 26 27 28 29 30 31
1
2
3
4
Group and Phase

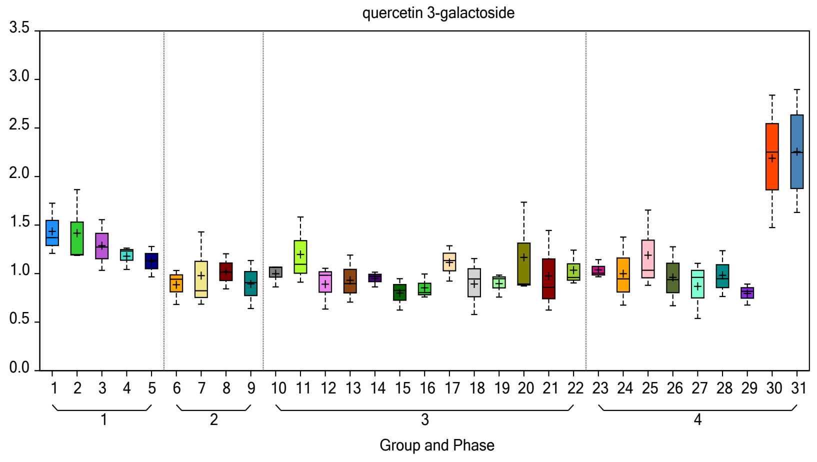
quercetin 3-galactoside
3.5
3.0
2.5
2.0
1.5
1.0
0.5
0.0
1 2 3 4 5 6 7 8 9 10 11 12 13 14 15 16 17 18 19 20 21 22 23 24 25 26 27 28 29 30 31
1
2
3
4
Group and Phase

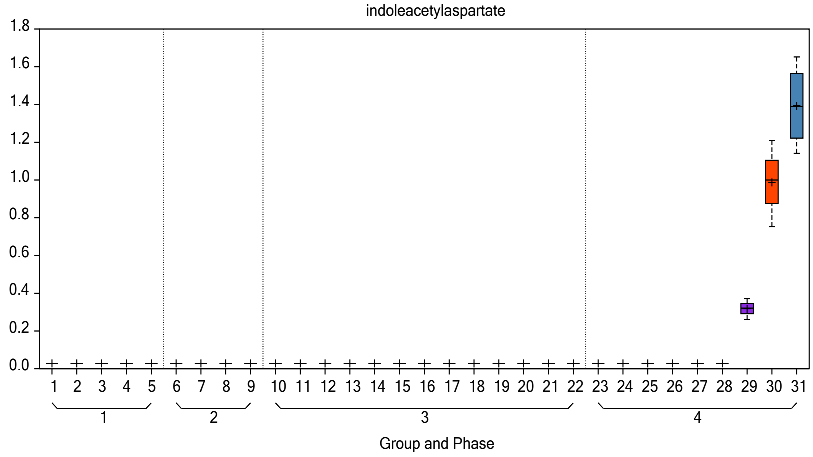
indoleacetylaspartate
1.8
1.6
1.4
1.2
1.0
0.8
0.6
0.4
0.2
0.0
1 2 3 4 5 6 7 8 9 10 11 12 13 14 15 16 17 18 19 20 21 22 23 24 25 26 27 28 29 30 31
1 2 3 4
Group and Phase

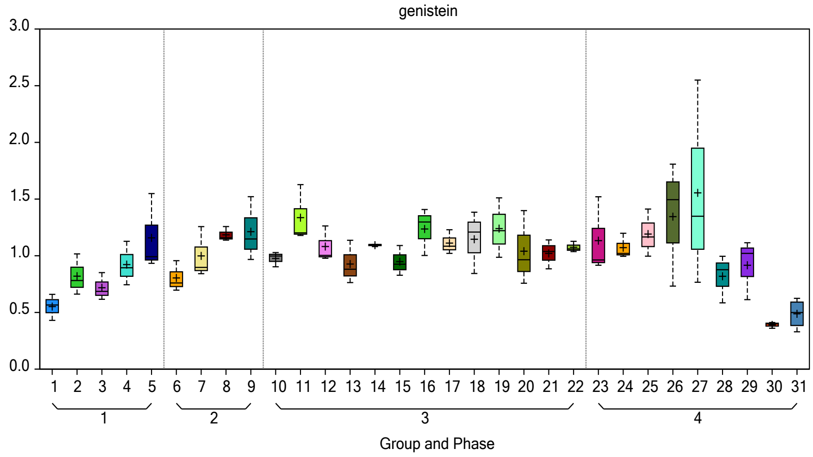
genistein
3.0
2.5
2.0
1.5
1.0
0.5
0.0
1 2 3 4 5 6 7 8 9 10 11 12 13 14 15 16 17 18 19 20 21 22 23 24 25 26 27 28 29 30 31
1 2 3 4
Group and Phase

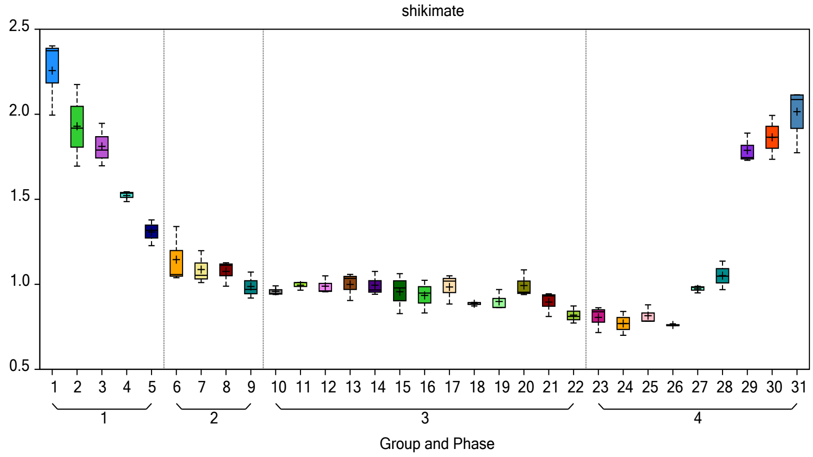
shikimate
2.5
2.0
1.5
1.0
0.5
1 2 3 4 5 6 7 8 9 10 11 12 13 14 15 16 17 18 19 20 21 22 23 24 25 26 27 28 29 30 31
1 2 3 4
Group and Phase

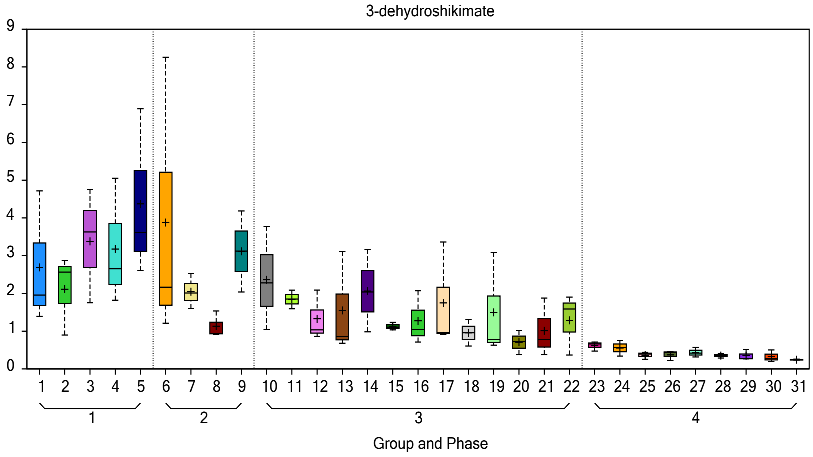
3-dehydroshikimate
9
8
7
6
5
4
3
2
1
0
1 2 3 4 5 6 7 8 9 10 11 12 13 14 15 16 17 18 19 20 21 22 23 24 25 26 27 28 29 30 31
1 2 3 4
Group and Phase

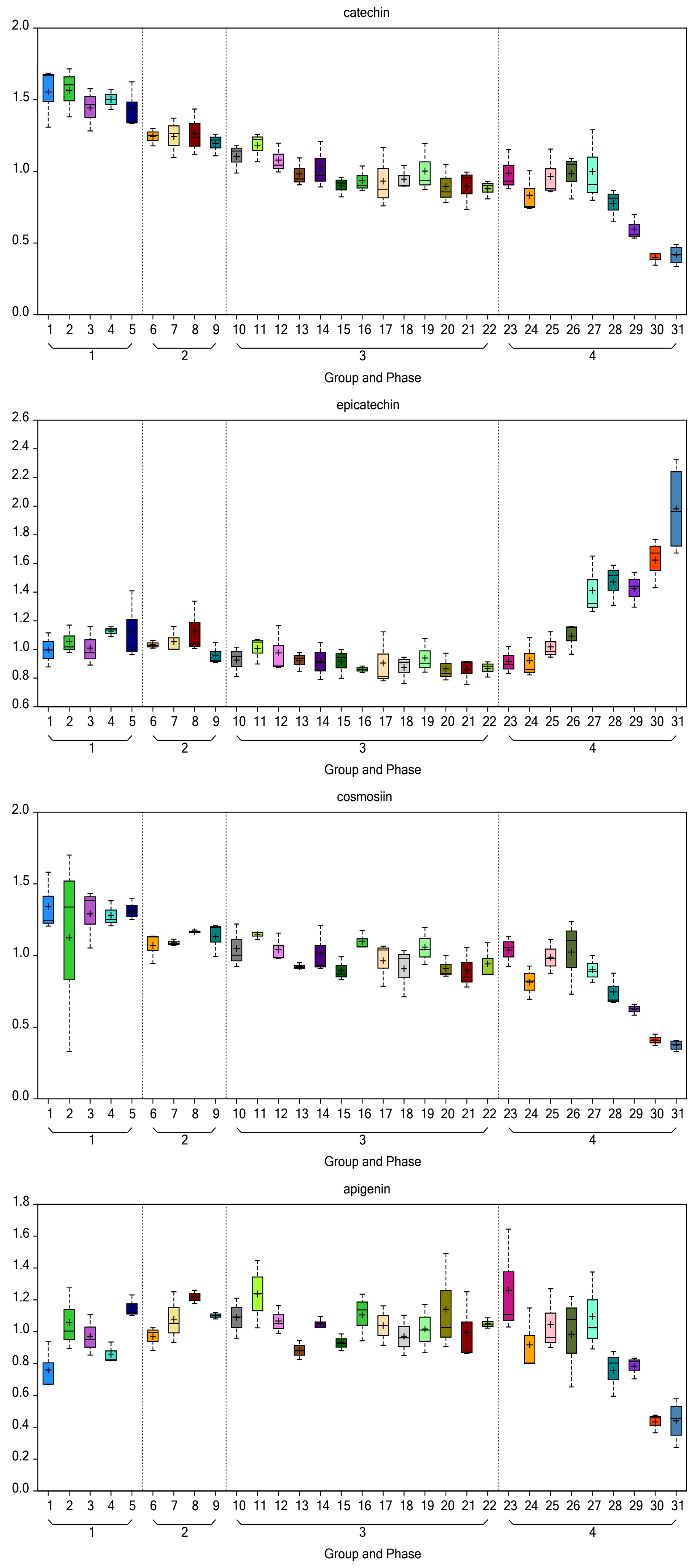
catechin
epicatechin
cosmosiin
apigenin
Group and Phase
1 2 3 4 5 6 7 8 9 10 11 12 13 14 15 16 17 18 19 20 21 22 23 24 25 26 27 28 29 30 31
1
2
3
4

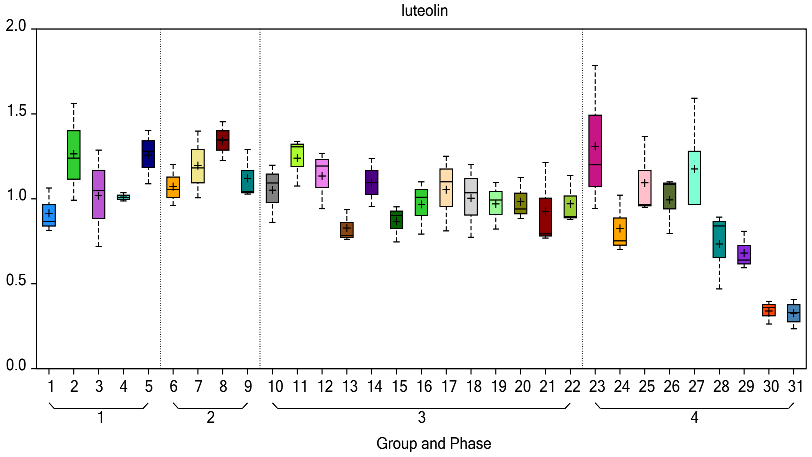
luteolin
2.0
1.5
1.0
0.5
0.0
1 2 3 4 5 6 7 8 9 10 11 12 13 14 15 16 17 18 19 20 21 22 23 24 25 26 27 28 29 30 31
1
2
3
4
Group and Phase

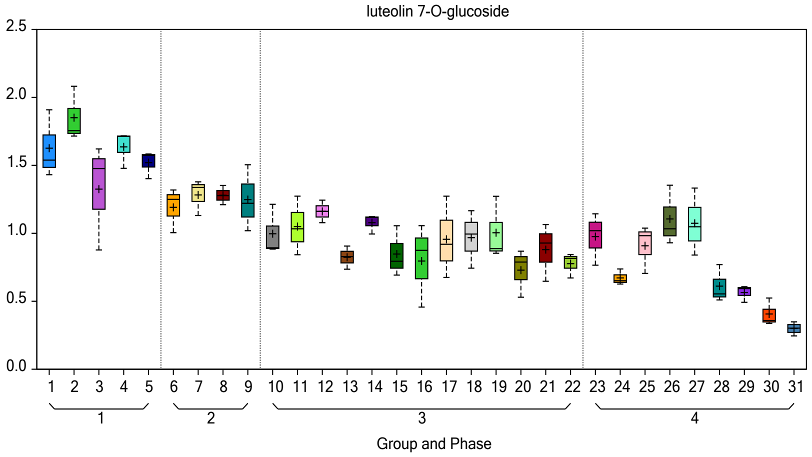
luteolin 7-O-glucoside
2.5
2.0
1.5
1.0
0.5
0.0
1 2 3 4 5 6 7 8 9 10 11 12 13 14 15 16 17 18 19 20 21 22 23 24 25 26 27 28 29 30 31
1
2
3
4
Group and Phase

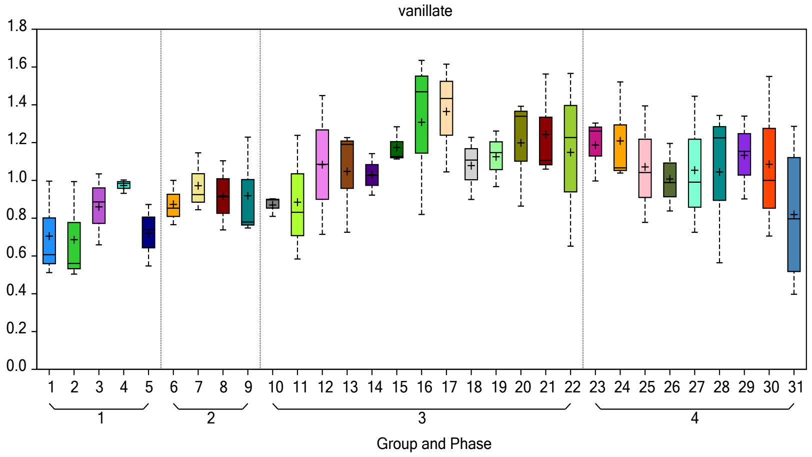
vanillate
1.8
1.6
1.4
1.2
1.0
0.8
0.6
0.4
0.2
0.0
1 2 3 4 5 6 7 8 9 10 11 12 13 14 15 16 17 18 19 20 21 22 23 24 25 26 27 28 29 30 31
1
2
3
4
Group and Phase

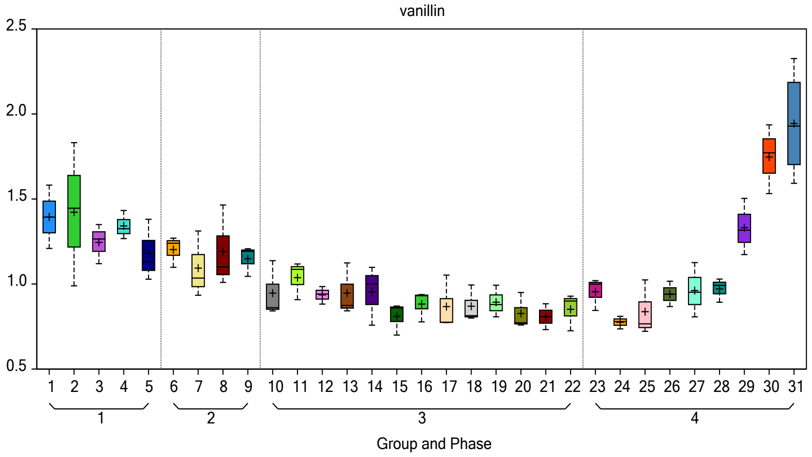
vanillin
2.5
2.0
1.5
1.0
0.5
1 2 3 4 5 6 7 8 9 10 11 12 13 14 15 16 17 18 19 20 21 22 23 24 25 26 27 28 29 30 31
1
2
3
4
Group and Phase

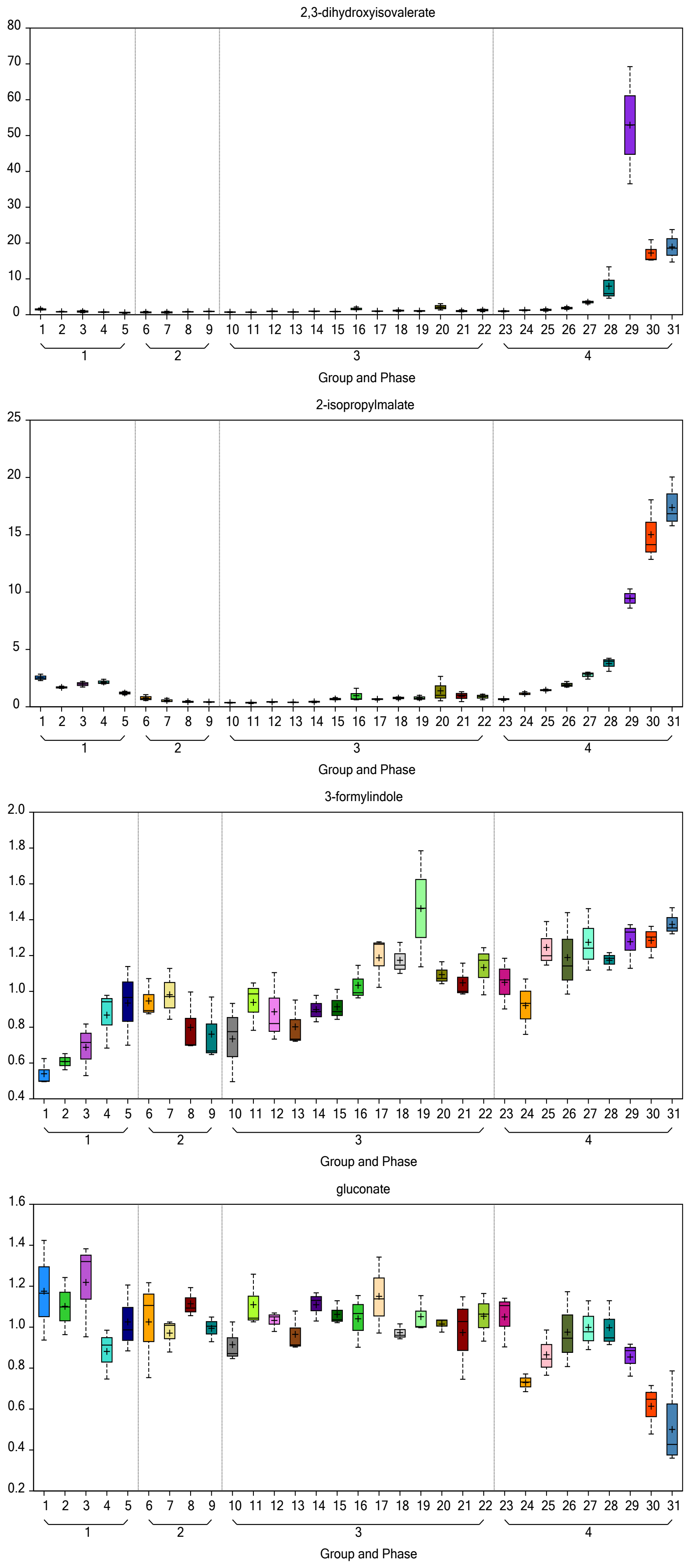
2,3-dihydroxyisovalerate
2-isopropylmalate
3-formylindole
gluconate
Group and Phase

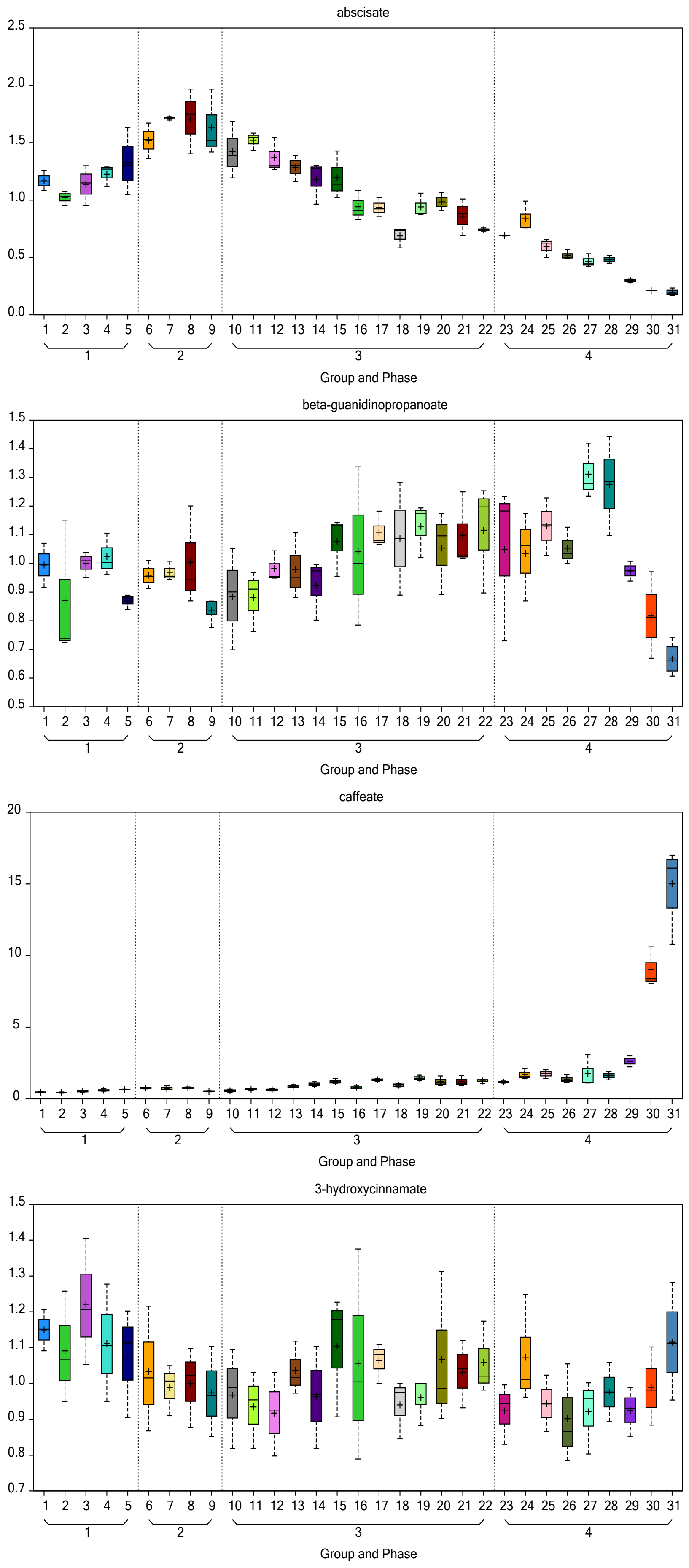
abscisate
Group and Phase
beta-guanidinopropanoate
Group and Phase
caffeate
Group and Phase
3-hydroxycinnamate
Group and Phase

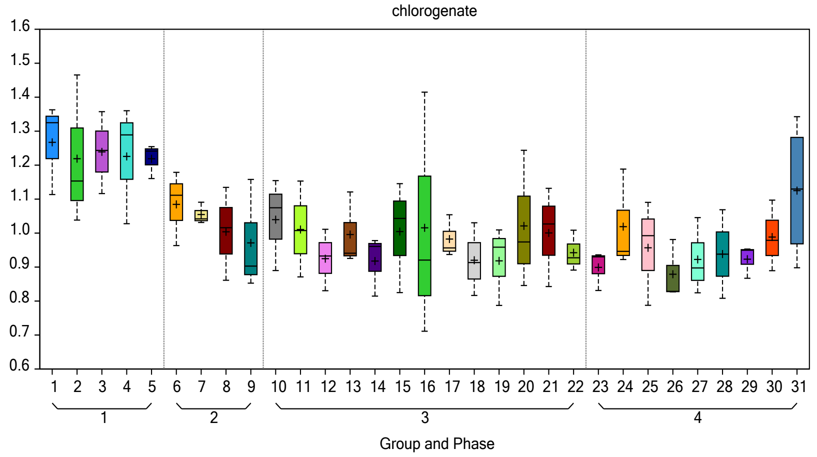
chlorogenate
Group and Phase

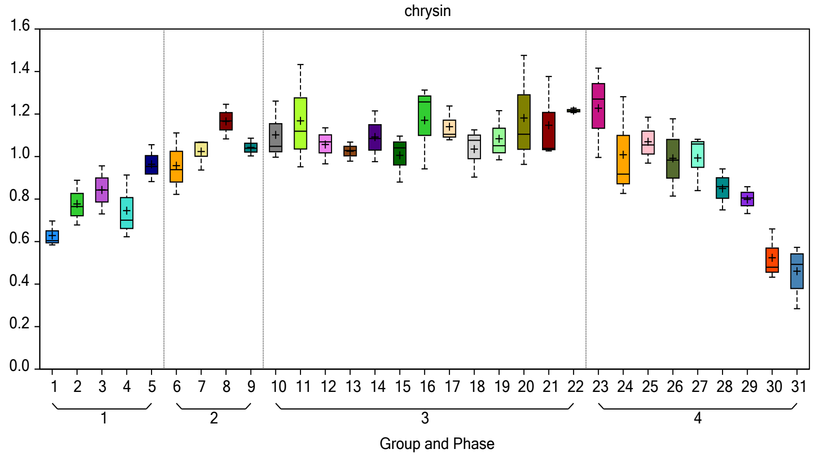
chrysin
Group and Phase

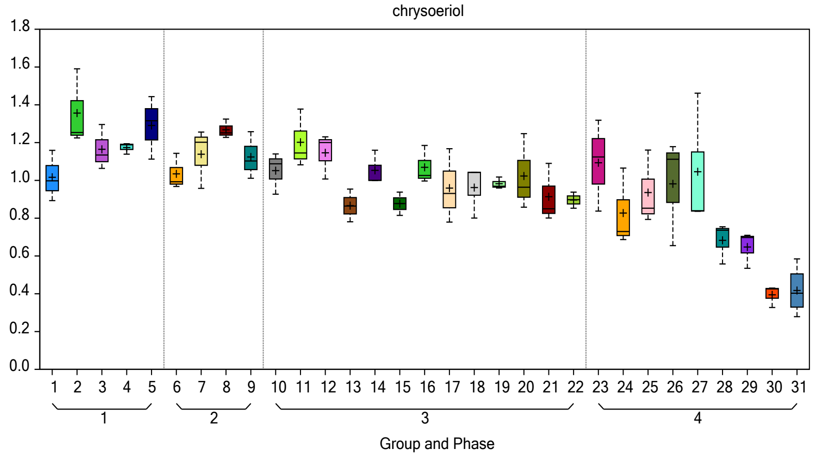
chrysoeriol
Group and Phase

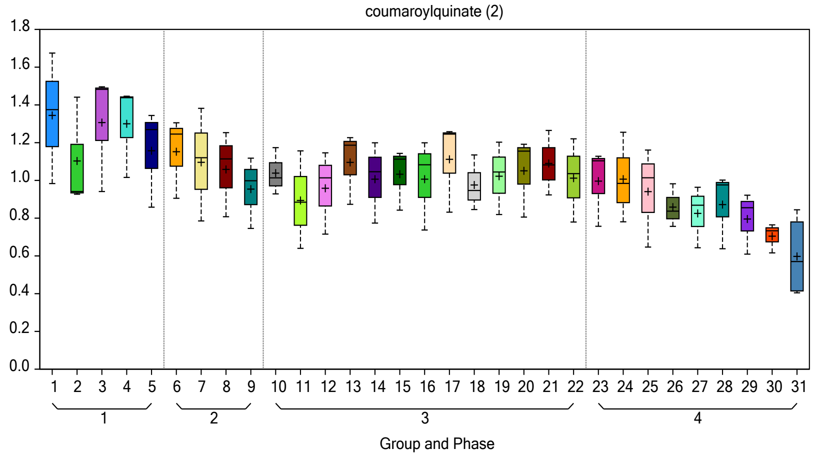
coumaroylquinate (2)
Group and Phase

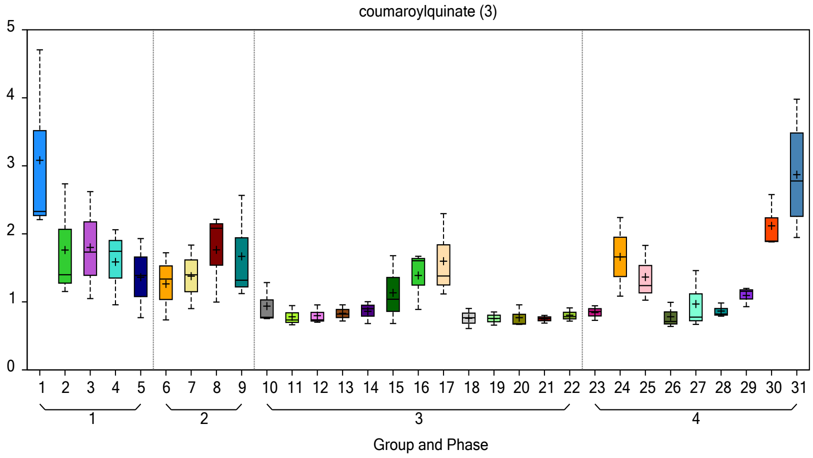
coumaroylquinate (3)
0 1 2 3 4 5
1 2 3 4 5 6 7 8 9 10 11 12 13 14 15 16 17 18 19 20 21 22 23 24 25 26 27 28 29 30 31
1 2 3 4
Group and Phase

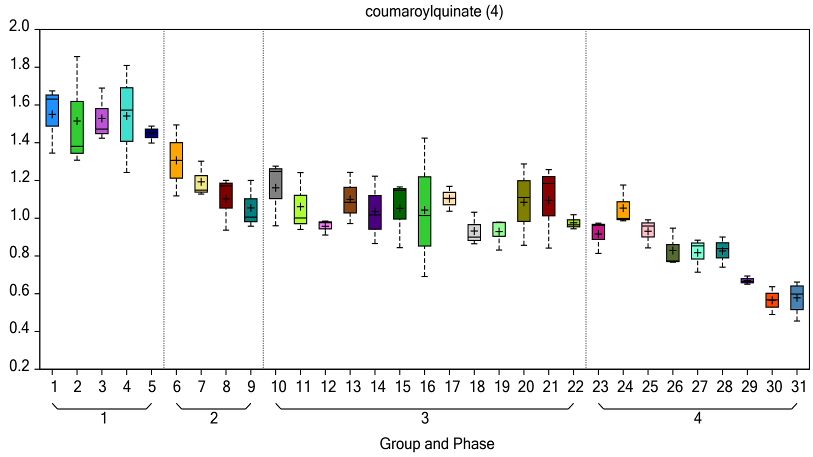
coumaroylquinate (4)
0.2 0.4 0.6 0.8 1.0 1.2 1.4 1.6 1.8 2.0
1 2 3 4 5 6 7 8 9 10 11 12 13 14 15 16 17 18 19 20 21 22 23 24 25 26 27 28 29 30 31
1 2 3 4
Group and Phase

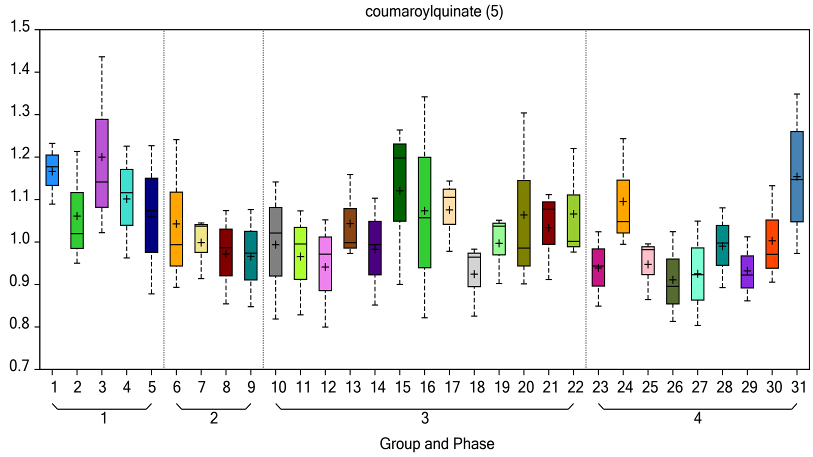
coumaroylquinate (5)
0.7 0.8 0.9 1.0 1.1 1.2 1.3 1.4 1.5
1 2 3 4 5 6 7 8 9 10 11 12 13 14 15 16 17 18 19 20 21 22 23 24 25 26 27 28 29 30 31
1 2 3 4
Group and Phase

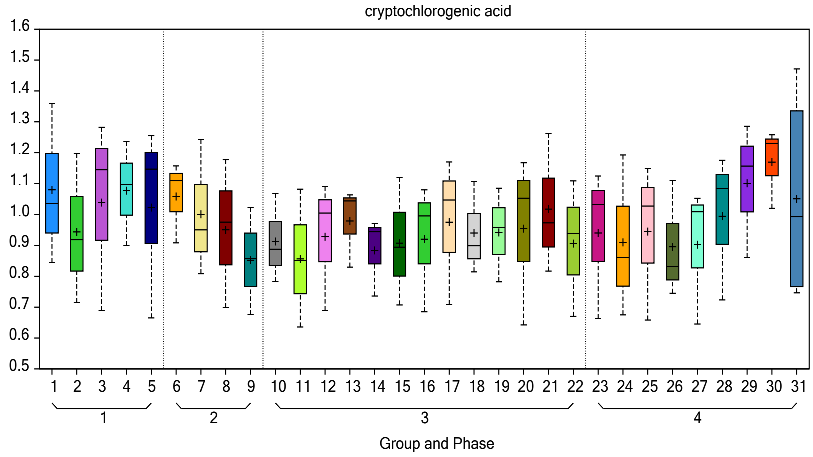
cryptochlorogenic acid
0.5 0.6 0.7 0.8 0.9 1.0 1.1 1.2 1.3 1.4 1.5 1.6
1 2 3 4 5 6 7 8 9 10 11 12 13 14 15 16 17 18 19 20 21 22 23 24 25 26 27 28 29 30 31
1 2 3 4
Group and Phase

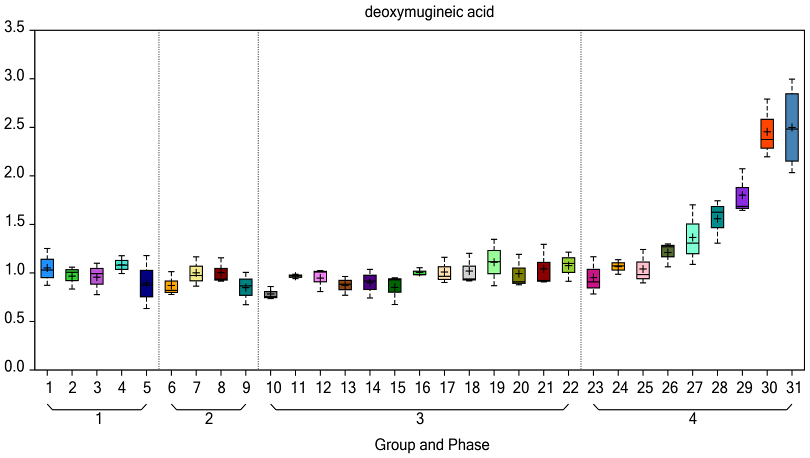
deoxymugineic acid
3.5
3.0
2.5
2.0
1.5
1.0
0.5
0.0
1 2 3 4 5 6 7 8 9 10 11 12 13 14 15 16 17 18 19 20 21 22 23 24 25 26 27 28 29 30 31
1
2
3
4
Group and Phase

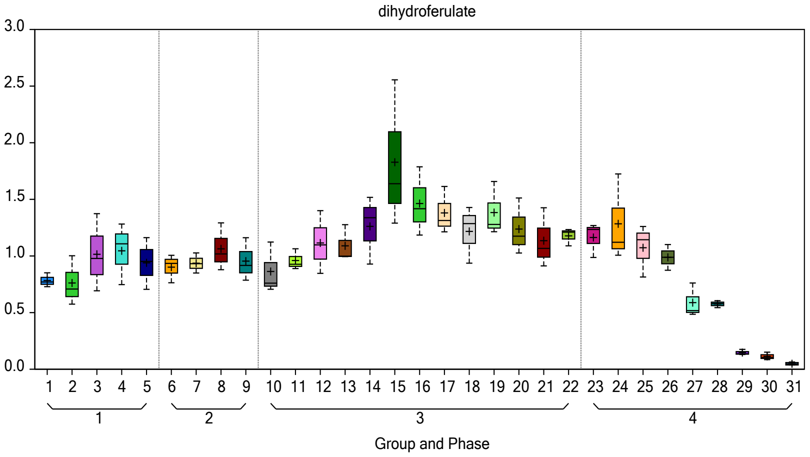
dihydroferulate
3.0
2.5
2.0
1.5
1.0
0.5
0.0
1 2 3 4 5 6 7 8 9 10 11 12 13 14 15 16 17 18 19 20 21 22 23 24 25 26 27 28 29 30 31
1
2
3
4
Group and Phase

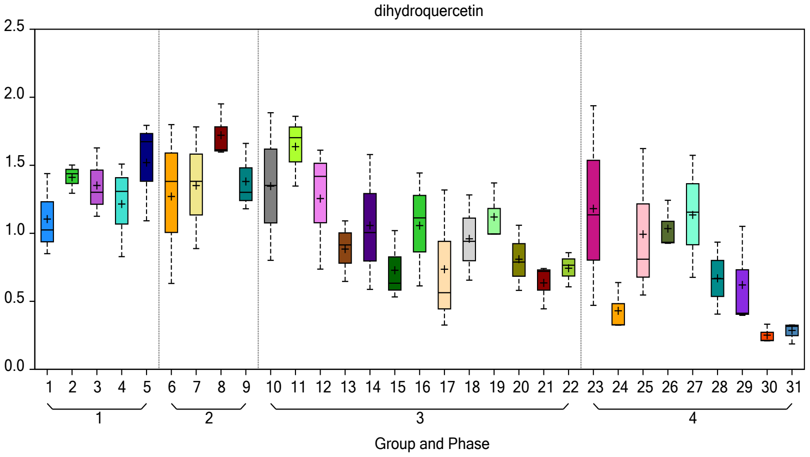
dihydroquercetin
2.5
2.0
1.5
1.0
0.5
0.0
1 2 3 4 5 6 7 8 9 10 11 12 13 14 15 16 17 18 19 20 21 22 23 24 25 26 27 28 29 30 31
1
2
3
4
Group and Phase

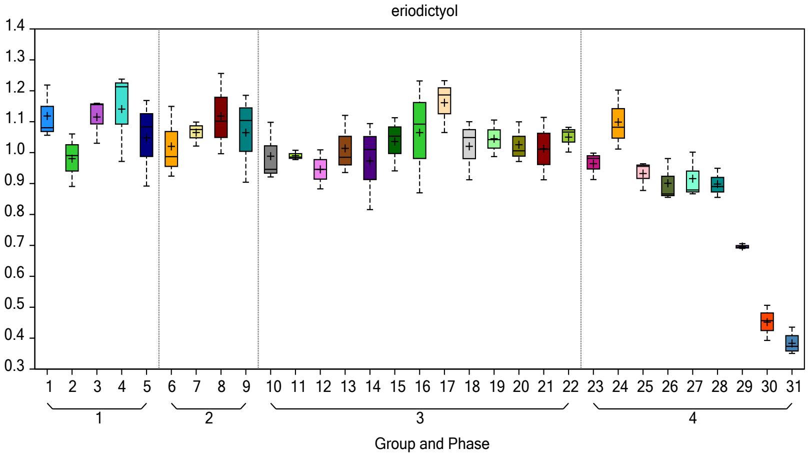
eriodictyol
1.4
1.3
1.2
1.1
1.0
0.9
0.8
0.7
0.6
0.5
0.4
0.3
1 2 3 4 5 6 7 8 9 10 11 12 13 14 15 16 17 18 19 20 21 22 23 24 25 26 27 28 29 30 31
1
2
3
4
Group and Phase

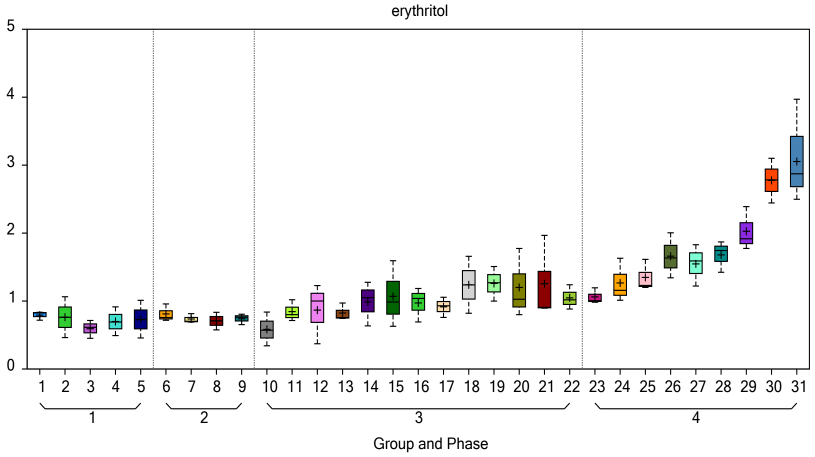
erythritol
5
4
3
2
1
0
1 2 3 4 5 6 7 8 9 10 11 12 13 14 15 16 17 18 19 20 21 22 23 24 25 26 27 28 29 30 31
1 2 3 4
Group and Phase

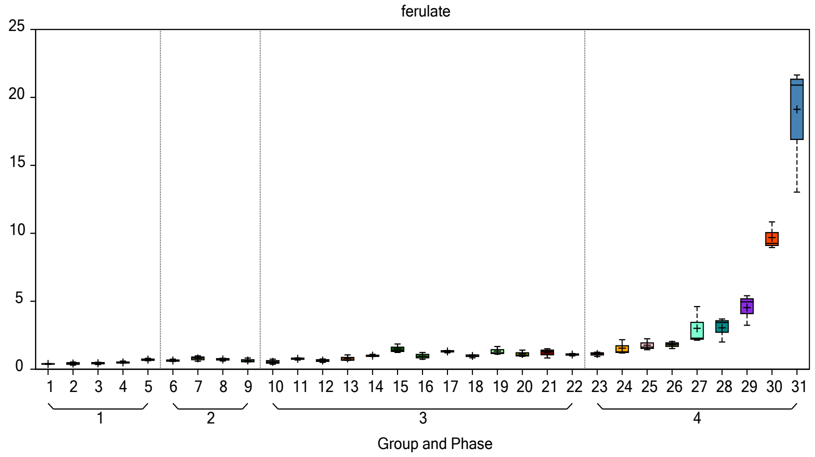
ferulate
25
20
15
10
5
0
1 2 3 4 5 6 7 8 9 10 11 12 13 14 15 16 17 18 19 20 21 22 23 24 25 26 27 28 29 30 31
1 2 3 4
Group and Phase

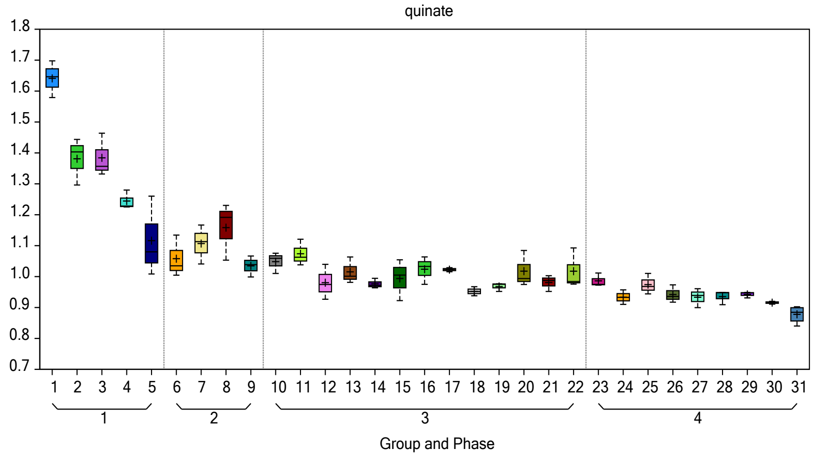
quinate
1.8
1.7
1.6
1.5
1.4
1.3
1.2
1.1
1.0
0.9
0.8
0.7
1 2 3 4 5 6 7 8 9 10 11 12 13 14 15 16 17 18 19 20 21 22 23 24 25 26 27 28 29 30 31
1 2 3 4
Group and Phase

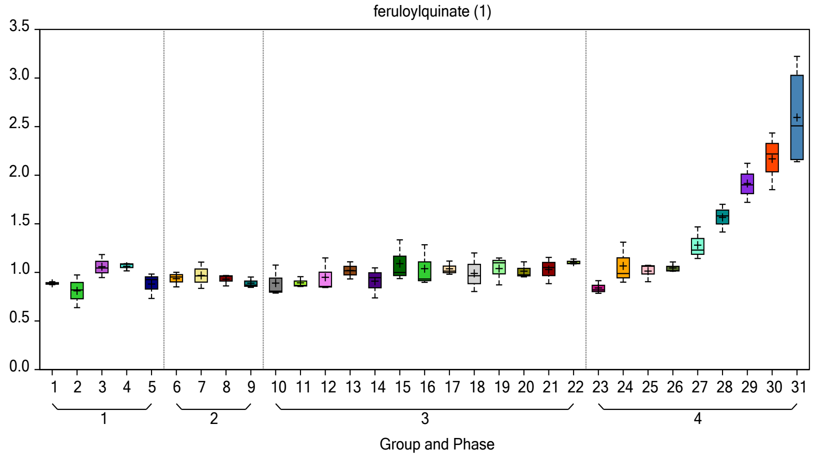
feruloylquinate (1)
3.5
3.0
2.5
2.0
1.5
1.0
0.5
0.0
1 2 3 4 5 6 7 8 9 10 11 12 13 14 15 16 17 18 19 20 21 22 23 24 25 26 27 28 29 30 31
1 2 3 4
Group and Phase

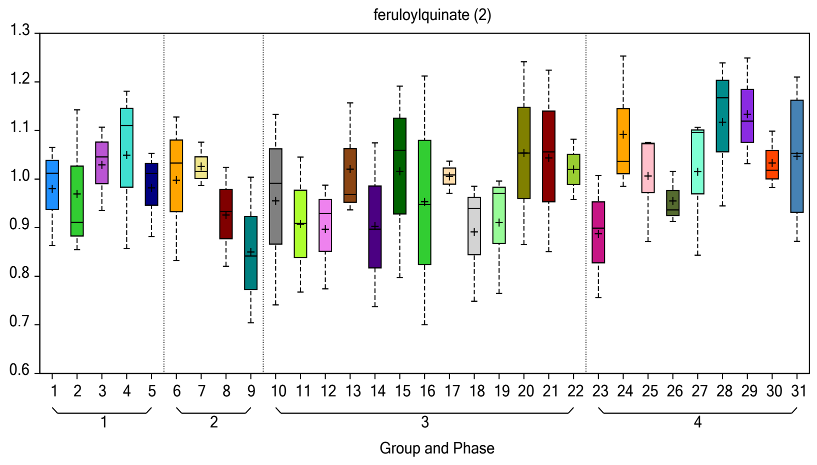
feruloylquinate (2)
Group and Phase

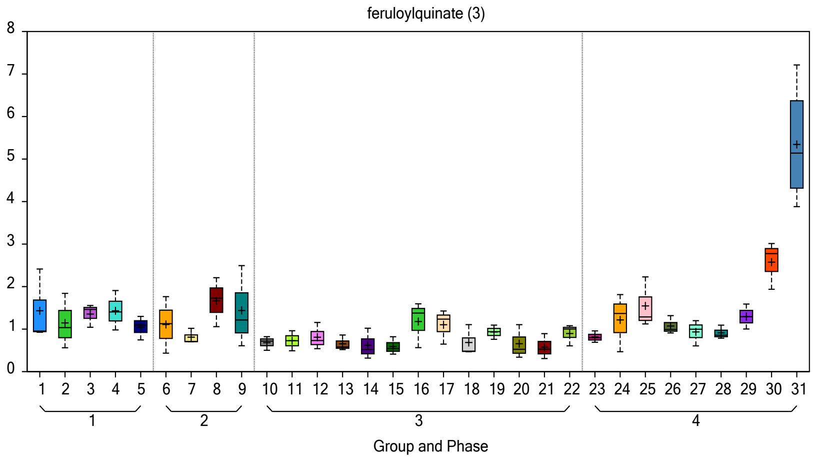
feruloylquinate (3)
Group and Phase

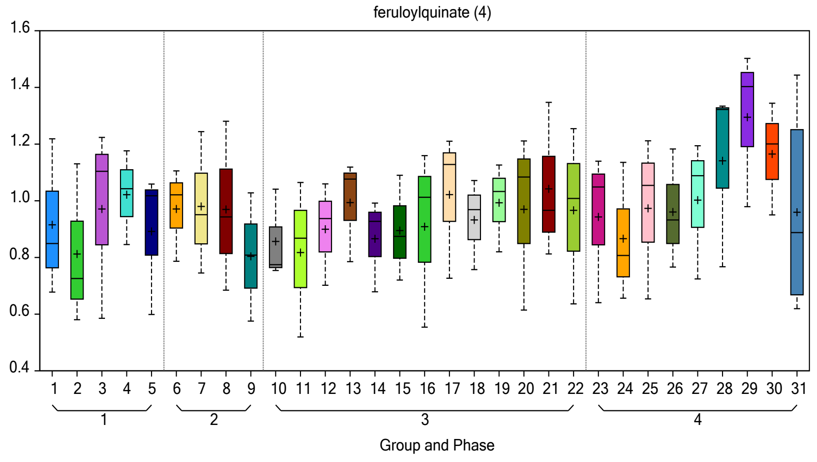
feruloylquinate (4)
Group and Phase

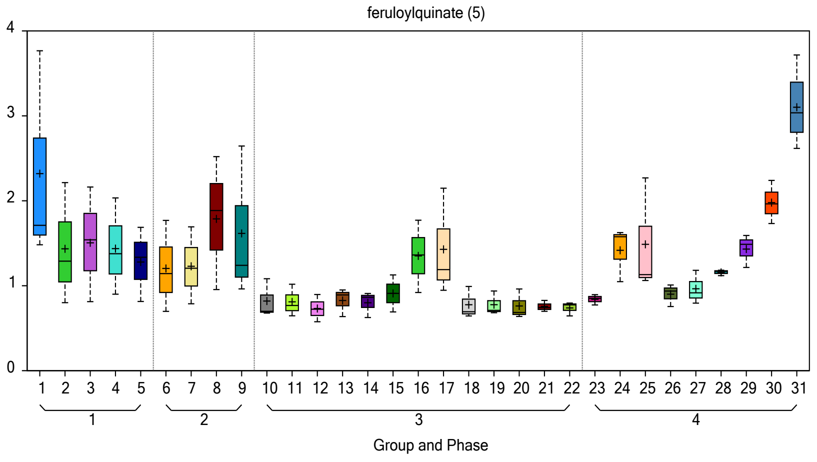
feruloylquinate (5)
Group and Phase

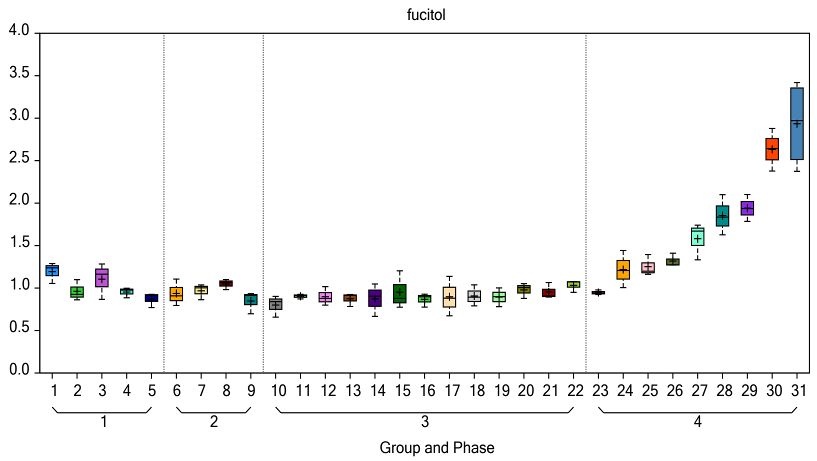
fucitol
4.0
3.5
3.0
2.5
2.0
1.5
1.0
0.5
0.0
1 2 3 4 5 6 7 8 9 10 11 12 13 14 15 16 17 18 19 20 21 22 23 24 25 26 27 28 29 30 31
1
2
3
4
Group and Phase

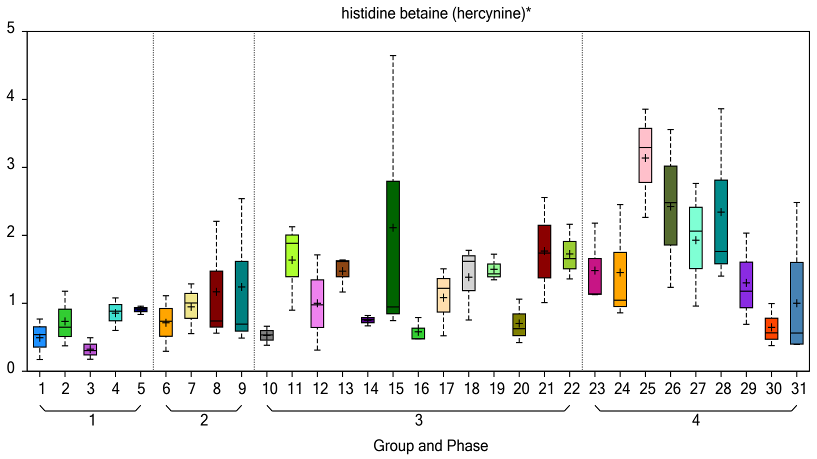
histidine betaine (hercynine)*
5
4
3
2
1
0
1 2 3 4 5 6 7 8 9 10 11 12 13 14 15 16 17 18 19 20 21 22 23 24 25 26 27 28 29 30 31
1
2
3
4
Group and Phase

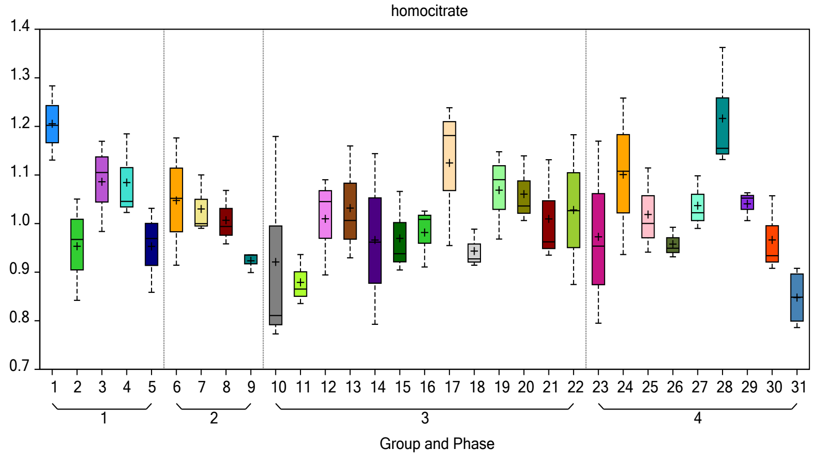
homocitrate
1.4
1.3
1.2
1.1
1.0
0.9
0.8
0.7
1 2 3 4 5 6 7 8 9 10 11 12 13 14 15 16 17 18 19 20 21 22 23 24 25 26 27 28 29 30 31
1
2
3
4
Group and Phase

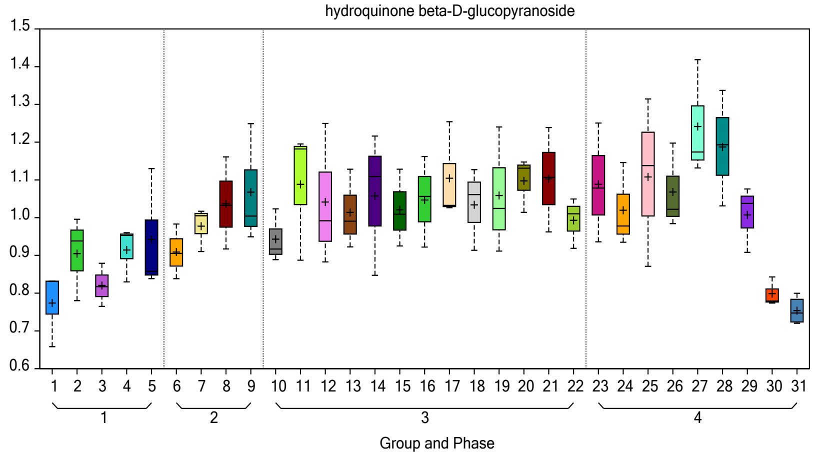
hydroquinone beta-D-glucopyranoside
1.5
1.4
1.3
1.2
1.1
1.0
0.9
0.8
0.7
0.6
1 2 3 4 5 6 7 8 9 10 11 12 13 14 15 16 17 18 19 20 21 22 23 24 25 26 27 28 29 30 31
1
2
3
4
Group and Phase

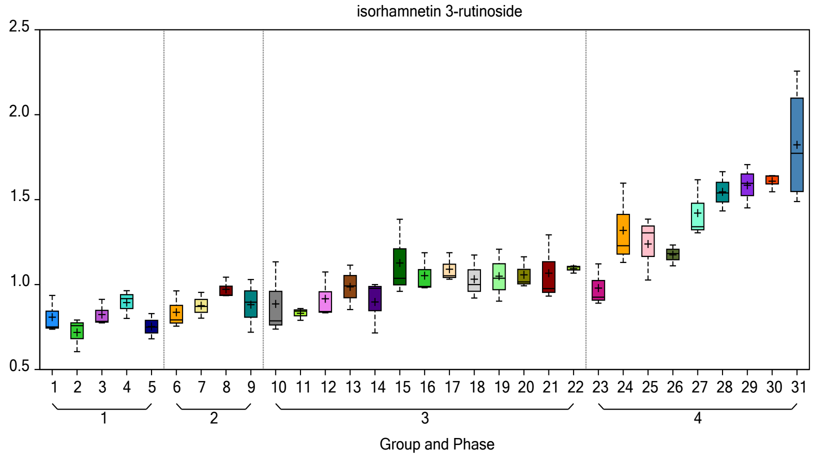
isorhamnetin 3-rutinoside
2.5
2.0
1.5
1.0
0.5
1 2 3 4 5 6 7 8 9 10 11 12 13 14 15 16 17 18 19 20 21 22 23 24 25 26 27 28 29 30 31
1
2
3
4
Group and Phase

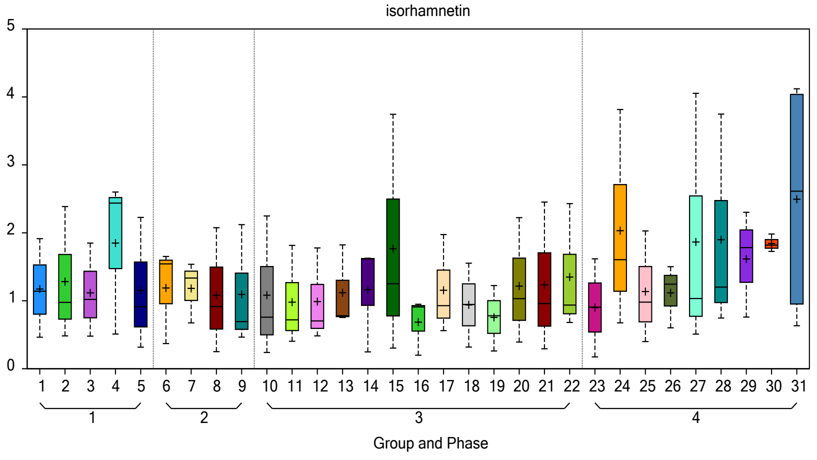
isorhamnetin
5
4
3
2
1
0
1 2 3 4 5 6 7 8 9 10 11 12 13 14 15 16 17 18 19 20 21 22 23 24 25 26 27 28 29 30 31
1
2
3
4
Group and Phase

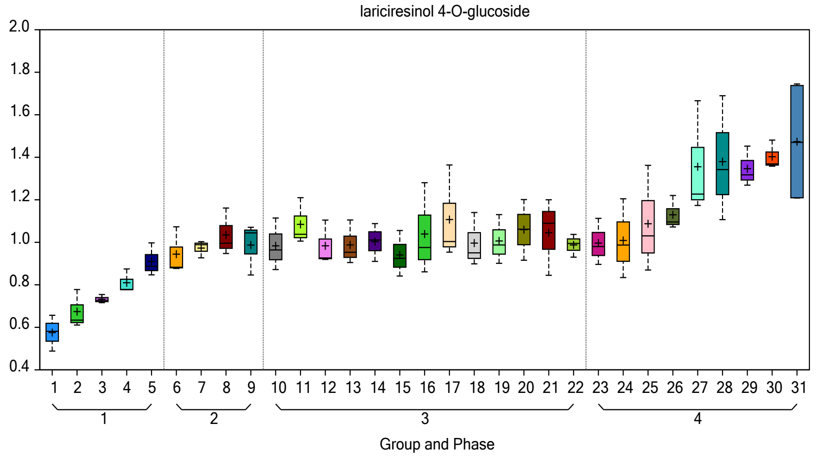
lariciresinol 4-O-glucoside
2.0
1.8
1.6
1.4
1.2
1.0
0.8
0.6
0.4
1 2 3 4 5 6 7 8 9 10 11 12 13 14 15 16 17 18 19 20 21 22 23 24 25 26 27 28 29 30 31
1
2
3
4
Group and Phase

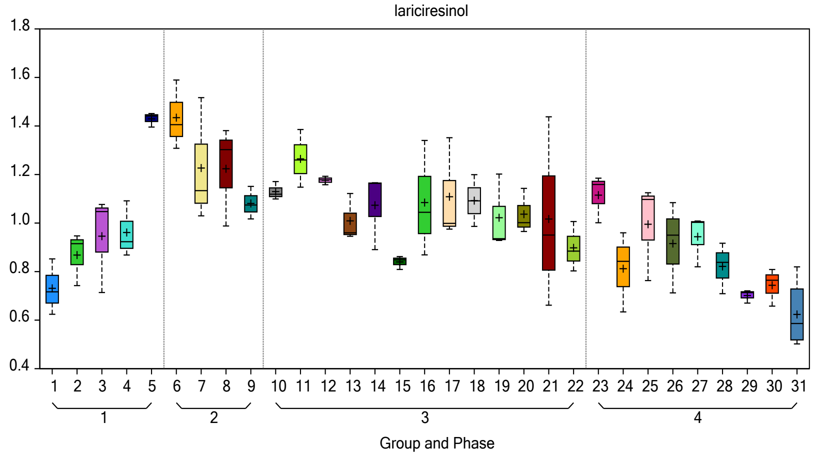
lariciresinol
1.8
1.6
1.4
1.2
1.0
0.8
0.6
0.4
1 2 3 4 5 6 7 8 9 10 11 12 13 14 15 16 17 18 19 20 21 22 23 24 25 26 27 28 29 30 31
1
2
3
4
Group and Phase

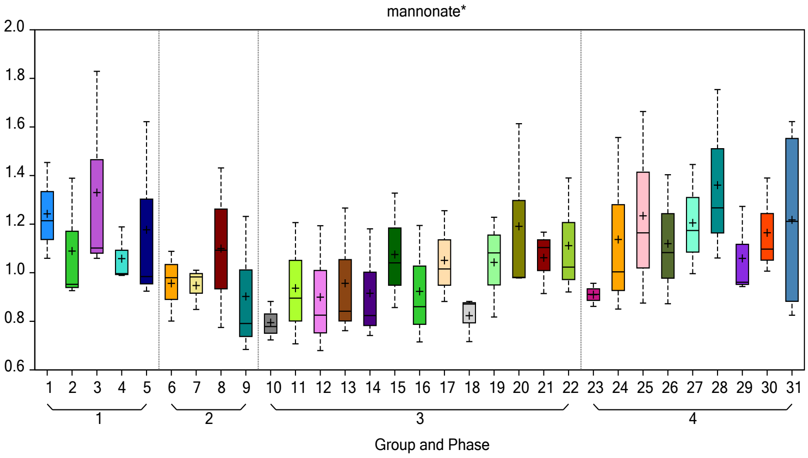
mannonate*
2.0
1.8
1.6
1.4
1.2
1.0
0.8
0.6
1 2 3 4 5 6 7 8 9 10 11 12 13 14 15 16 17 18 19 20 21 22 23 24 25 26 27 28 29 30 31
1
2
3
4
Group and Phase

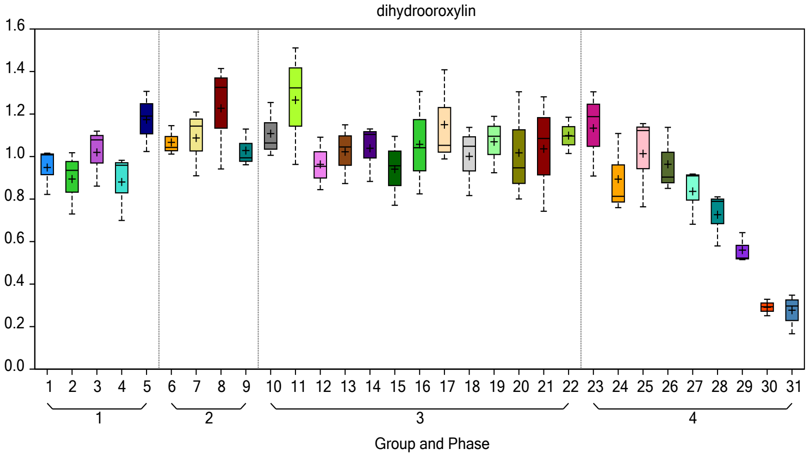
dihydrooroxylin
1.6
1.4
1.2
1.0
0.8
0.6
0.4
0.2
0.0
1 2 3 4 5 6 7 8 9 10 11 12 13 14 15 16 17 18 19 20 21 22 23 24 25 26 27 28 29 30 31
1
2
3
4
Group and Phase

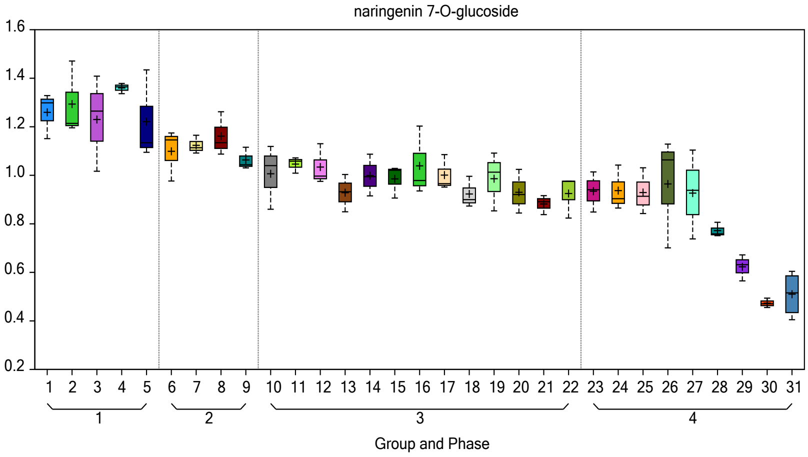
naringenin 7-O-glucoside
1.6
1.4
1.2
1.0
0.8
0.6
0.4
0.2
1 2 3 4 5 6 7 8 9 10 11 12 13 14 15 16 17 18 19 20 21 22 23 24 25 26 27 28 29 30 31
1
2
3
4
Group and Phase

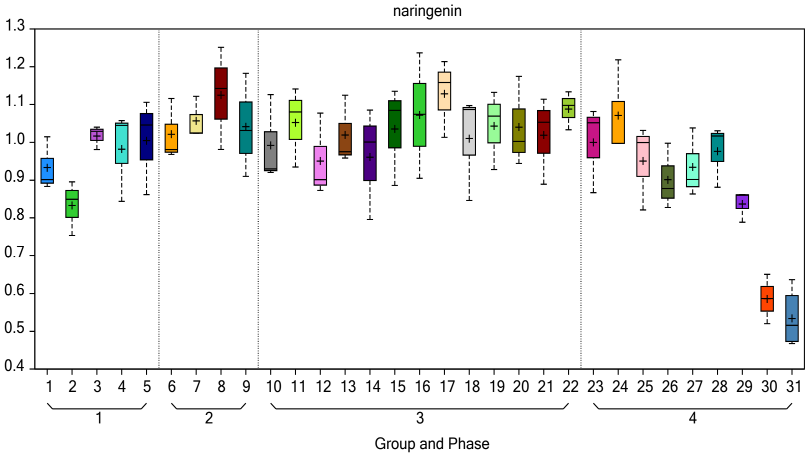
naringenin
1.3
1.2
1.1
1.0
0.9
0.8
0.7
0.6
0.5
0.4
1 2 3 4 5 6 7 8 9 10 11 12 13 14 15 16 17 18 19 20 21 22 23 24 25 26 27 28 29 30 31
1
2
3
4
Group and Phase

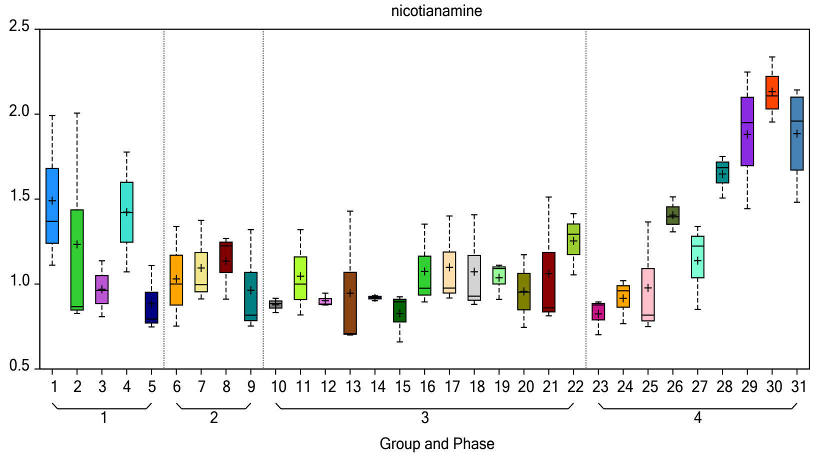
nicotianamine
2.5
2.0
1.5
1.0
0.5
1 2 3 4 5 6 7 8 9 10 11 12 13 14 15 16 17 18 19 20 21 22 23 24 25 26 27 28 29 30 31
1
2
3
4
Group and Phase

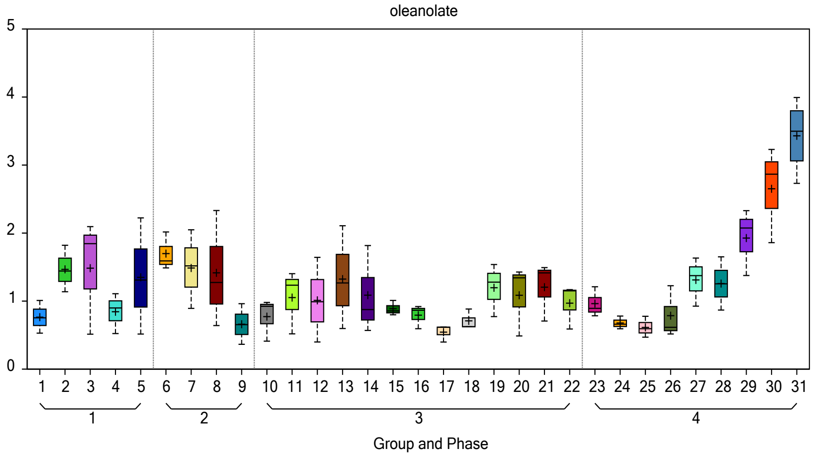
oleanolate
5
4
3
2
1
0
1 2 3 4 5 6 7 8 9 10 11 12 13 14 15 16 17 18 19 20 21 22 23 24 25 26 27 28 29 30 31
1
2
3
4
Group and Phase

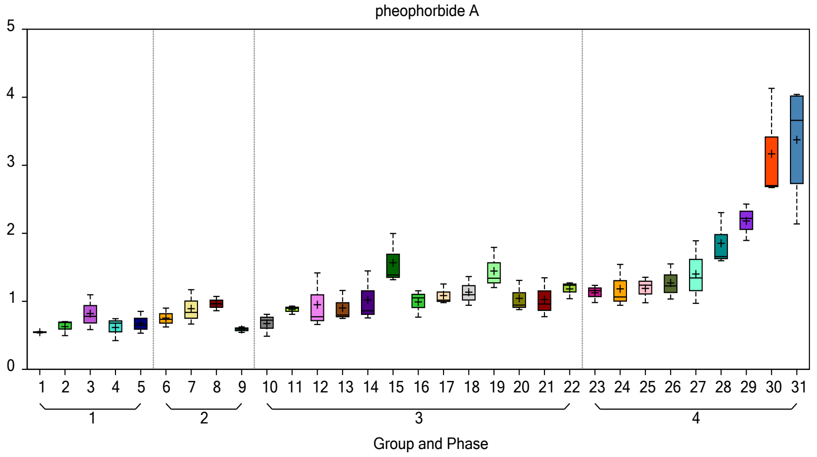
pheophorbide A
5
4
3
2
1
0
1 2 3 4 5 6 7 8 9 10 11 12 13 14 15 16 17 18 19 20 21 22 23 24 25 26 27 28 29 30 31
1
2
3
4
Group and Phase

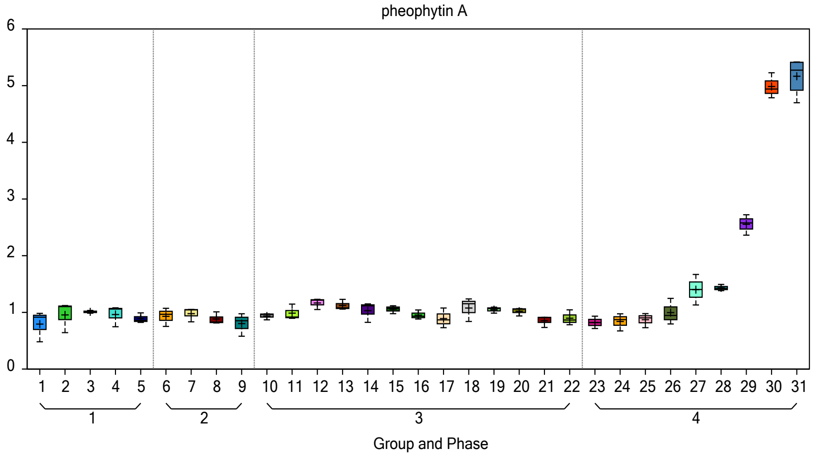
pheophytin A
6
5
4
3
2
1
0
1 2 3 4 5 6 7 8 9 10 11 12 13 14 15 16 17 18 19 20 21 22 23 24 25 26 27 28 29 30 31
1
2
3
4
Group and Phase

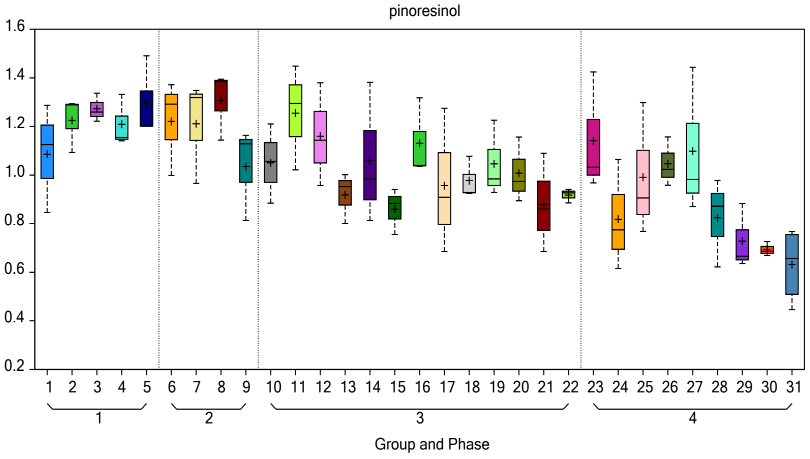
pinoresinol
1.6
1.4
1.2
1.0
0.8
0.6
0.4
0.2
1 2 3 4 5 6 7 8 9 10 11 12 13 14 15 16 17 18 19 20 21 22 23 24 25 26 27 28 29 30 31
1
2
3
4
Group and Phase

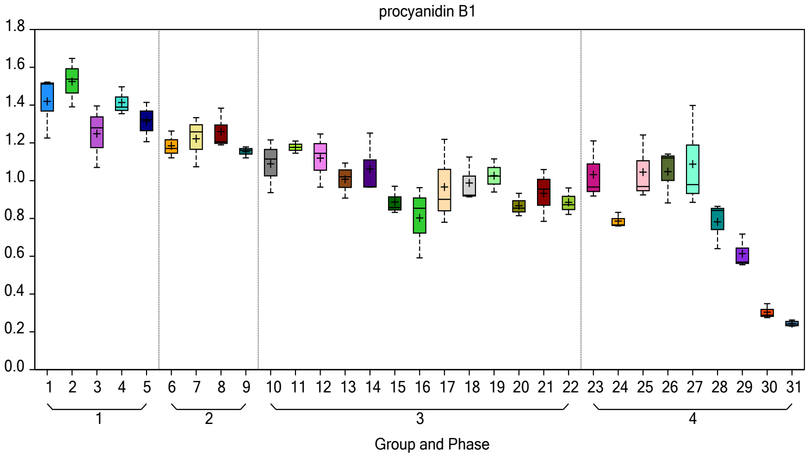
procyanidin B1
1.8
1.6
1.4
1.2
1.0
0.8
0.6
0.4
0.2
0.0
1 2 3 4 5 6 7 8 9 10 11 12 13 14 15 16 17 18 19 20 21 22 23 24 25 26 27 28 29 30 31
1
2
3
4
Group and Phase

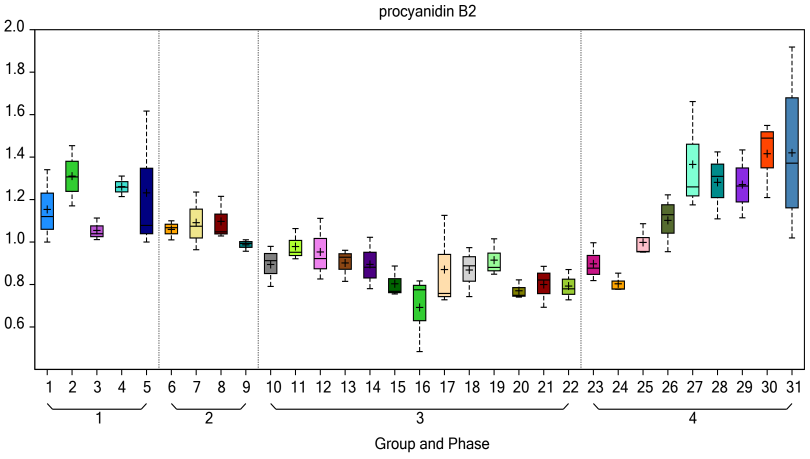
procyanidin B2
2.0
1.8
1.6
1.4
1.2
1.0
0.8
0.6
1 2 3 4 5 6 7 8 9 10 11 12 13 14 15 16 17 18 19 20 21 22 23 24 25 26 27 28 29 30 31
1
2
3
4
Group and Phase

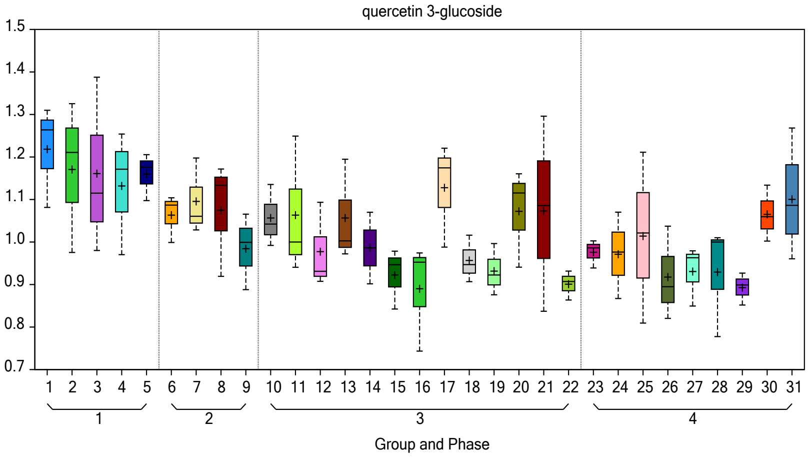
quercetin 3-glucoside
1.5
1.4
1.3
1.2
1.1
1.0
0.9
0.8
0.7
1 2 3 4 5 6 7 8 9 10 11 12 13 14 15 16 17 18 19 20 21 22 23 24 25 26 27 28 29 30 31
1
2
3
4
Group and Phase

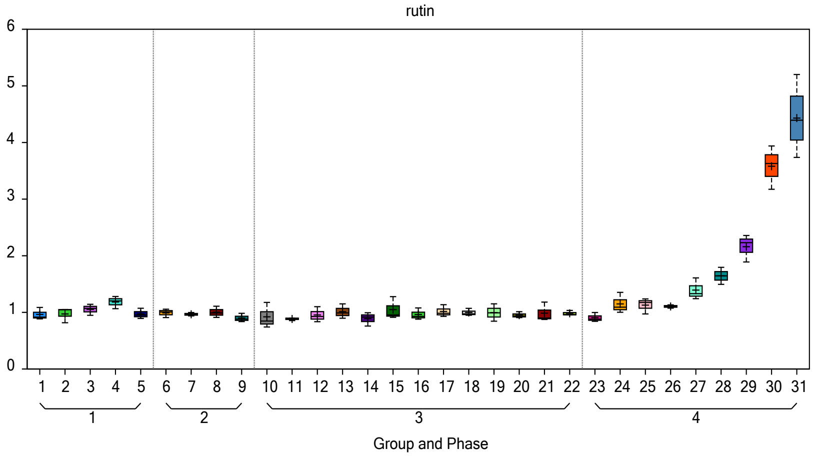
rutin
0 1 2 3 4 5 6
1 2 3 4 5 6 7 8 9 10 11 12 13 14 15 16 17 18 19 20 21 22 23 24 25 26 27 28 29 30 31
1 2 3 4
Group and Phase

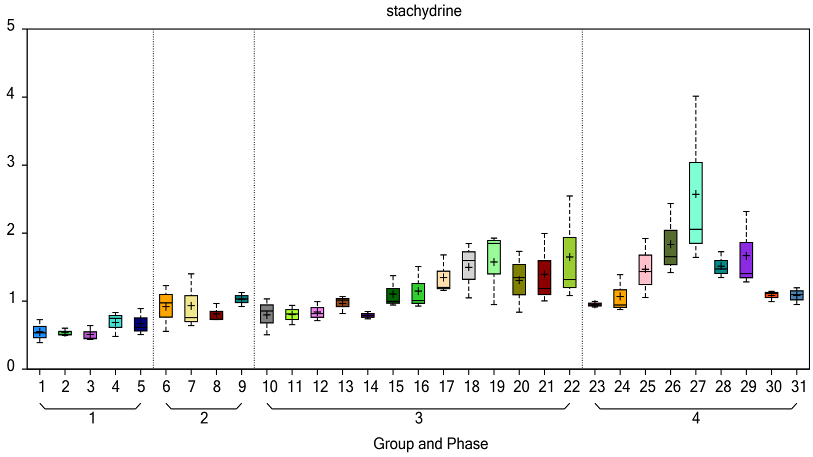
stachydrine
0 1 2 3 4 5
1 2 3 4 5 6 7 8 9 10 11 12 13 14 15 16 17 18 19 20 21 22 23 24 25 26 27 28 29 30 31
1 2 3 4
Group and Phase

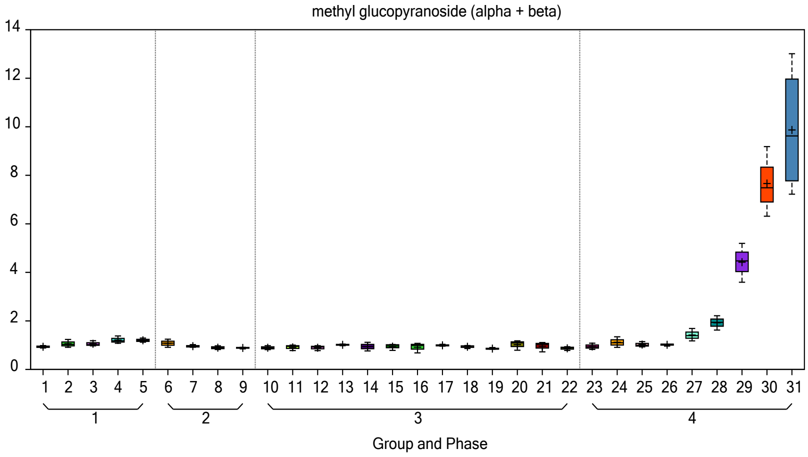
methyl glucopyranoside (alpha + beta)
0 2 4 6 8 10 12 14
1 2 3 4 5 6 7 8 9 10 11 12 13 14 15 16 17 18 19 20 21 22 23 24 25 26 27 28 29 30 31
1 2 3 4
Group and Phase

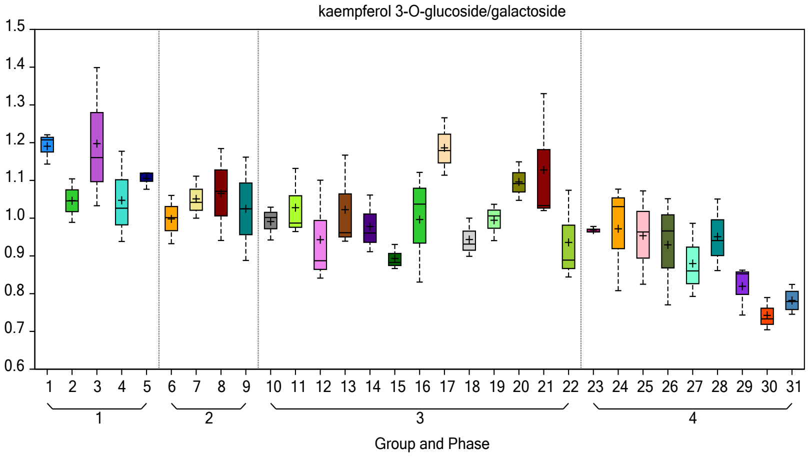
kaempferol 3-O-glucoside/galactoside
0.6 0.7 0.8 0.9 1.0 1.1 1.2 1.3 1.4 1.5
1 2 3 4 5 6 7 8 9 10 11 12 13 14 15 16 17 18 19 20 21 22 23 24 25 26 27 28 29 30 31
1 2 3 4
Group and Phase

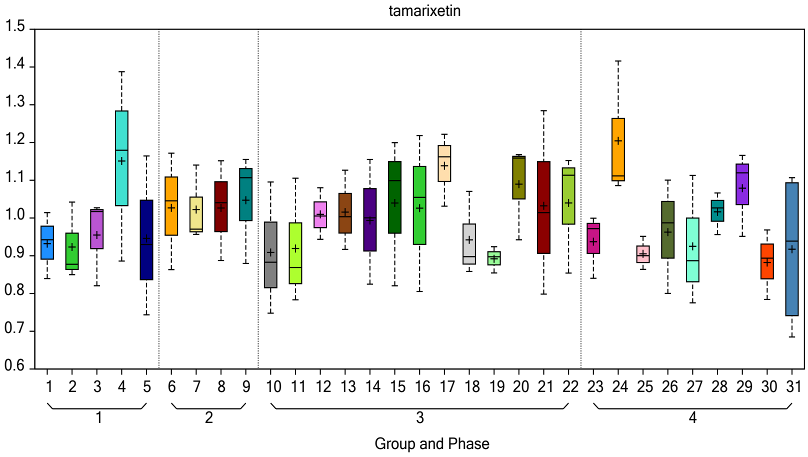
tamarixetin
1.5
1.4
1.3
1.2
1.1
1.0
0.9
0.8
0.7
0.6
1 2 3 4 5 6 7 8 9 10 11 12 13 14 15 16 17 18 19 20 21 22 23 24 25 26 27 28 29 30 31
1 2 3 4
Group and Phase

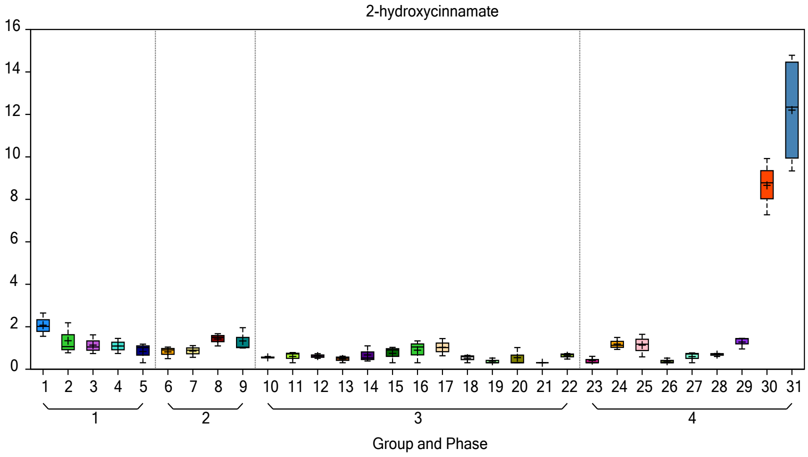
2-hydroxycinnamate
16
14
12
10
8
6
4
2
0
1 2 3 4 5 6 7 8 9 10 11 12 13 14 15 16 17 18 19 20 21 22 23 24 25 26 27 28 29 30 31
1 2 3 4
Group and Phase

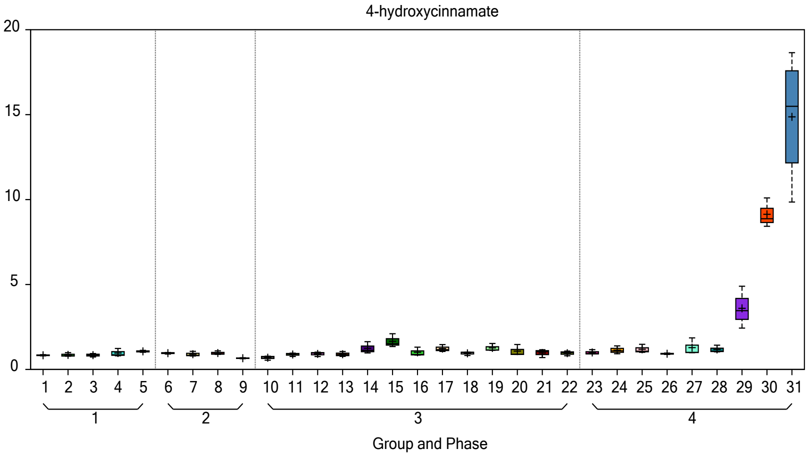
4-hydroxycinnamate
20
15
10
5
0
1 2 3 4 5 6 7 8 9 10 11 12 13 14 15 16 17 18 19 20 21 22 23 24 25 26 27 28 29 30 31
1 2 3 4
Group and Phase

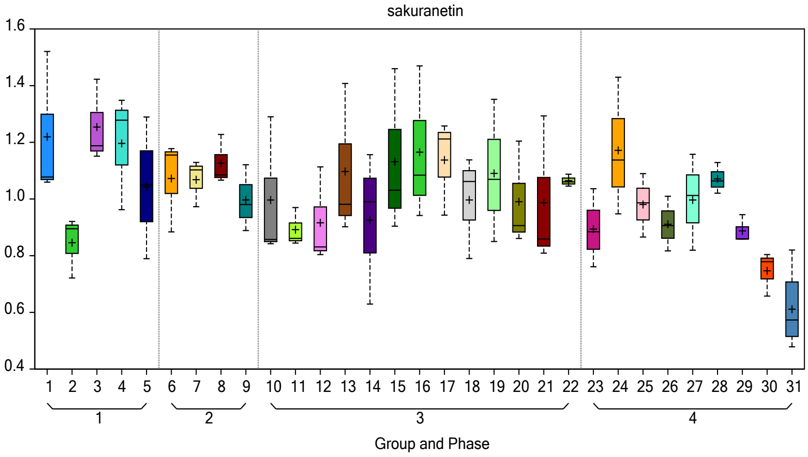
sakuranetin
1.6
1.4
1.2
1.0
0.8
0.6
0.4
1 2 3 4 5 6 7 8 9 10 11 12 13 14 15 16 17 18 19 20 21 22 23 24 25 26 27 28 29 30 31
1 2 3 4
Group and Phase

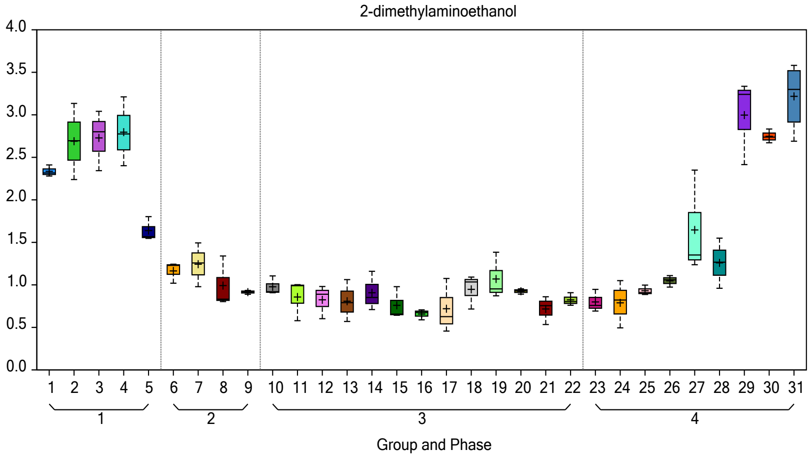
2-dimethylaminoethanol
4.0
3.5
3.0
2.5
2.0
1.5
1.0
0.5
0.0
1 2 3 4 5 6 7 8 9 10 11 12 13 14 15 16 17 18 19 20 21 22 23 24 25 26 27 28 29 30 31
1 2 3 4
Group and Phase

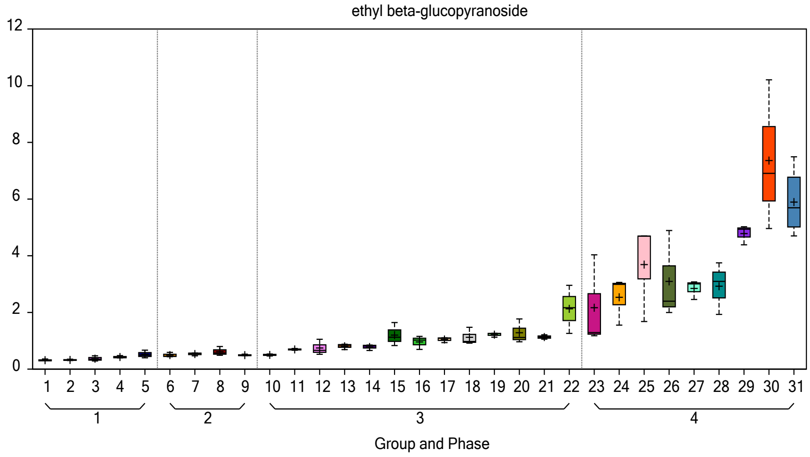
ethyl beta-glucopyranoside
12
10
8
6
4
2
0
1 2 3 4 5 6 7 8 9 10 11 12 13 14 15 16 17 18 19 20 21 22 23 24 25 26 27 28 29 30 31
1 2 3 4
Group and Phase

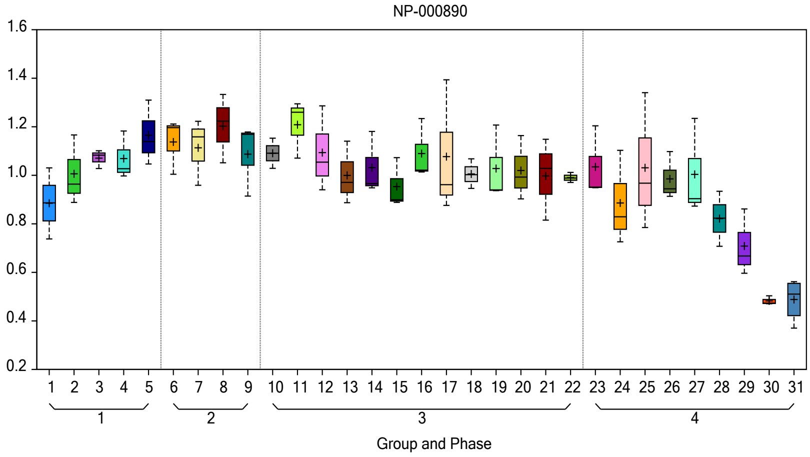
NP-000890
1.6
1.4
1.2
1.0
0.8
0.6
0.4
0.2
1 2 3 4 5 6 7 8 9 10 11 12 13 14 15 16 17 18 19 20 21 22 23 24 25 26 27 28 29 30 31
1 2 3 4
Group and Phase

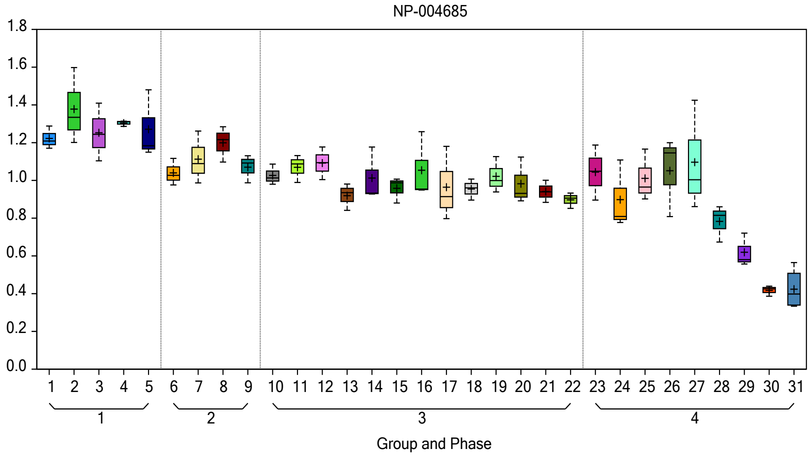
NP-004685
1.8
1.6
1.4
1.2
1.0
0.8
0.6
0.4
0.2
0.0
1 2 3 4 5 6 7 8 9 10 11 12 13 14 15 16 17 18 19 20 21 22 23 24 25 26 27 28 29 30 31
1 2 3 4
Group and Phase

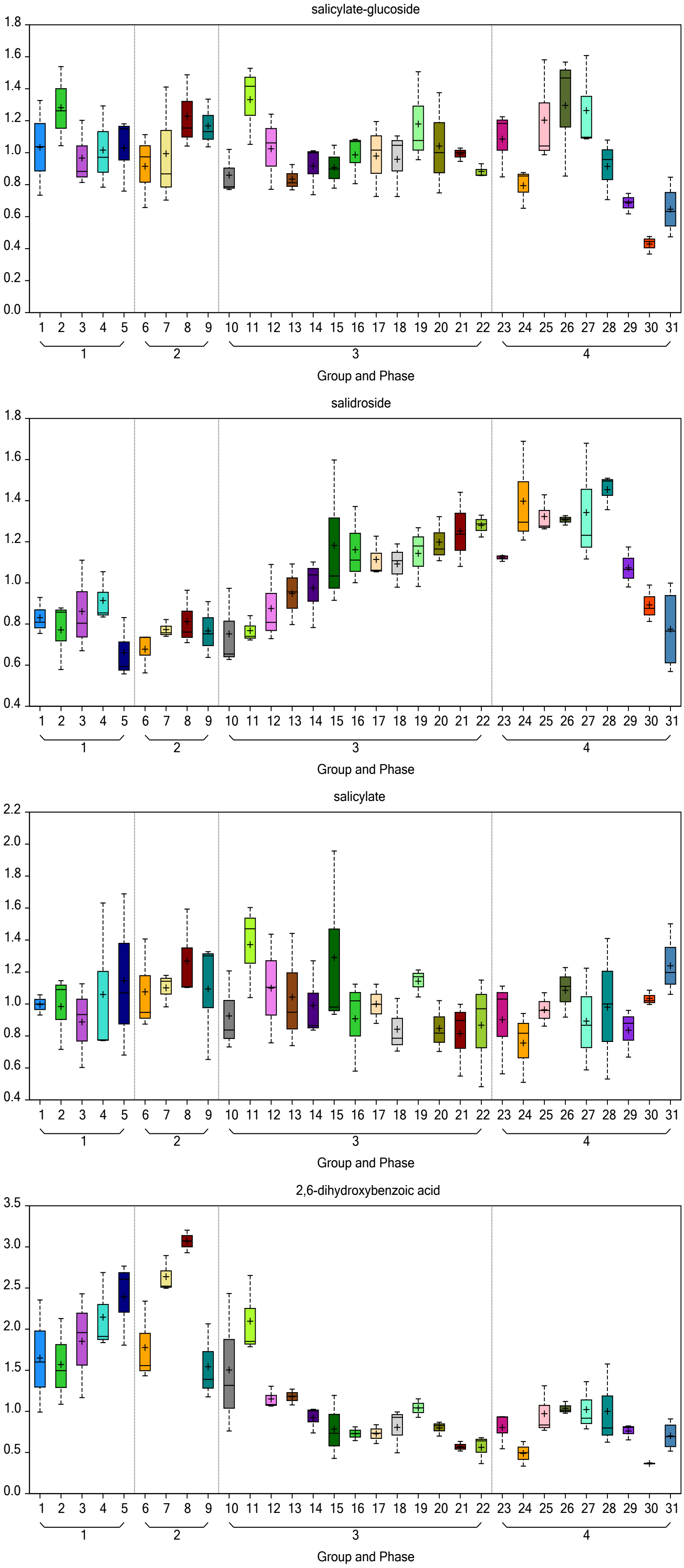
salicylate-glucoside
salidroside
salicylate
2,6-dihydroxybenzoic acid
1 2 3 4 5 6 7 8 9 10 11 12 13 14 15 16 17 18 19 20 21 22 23 24 25 26 27 28 29 30 31
1
2
3
4
Group and Phase

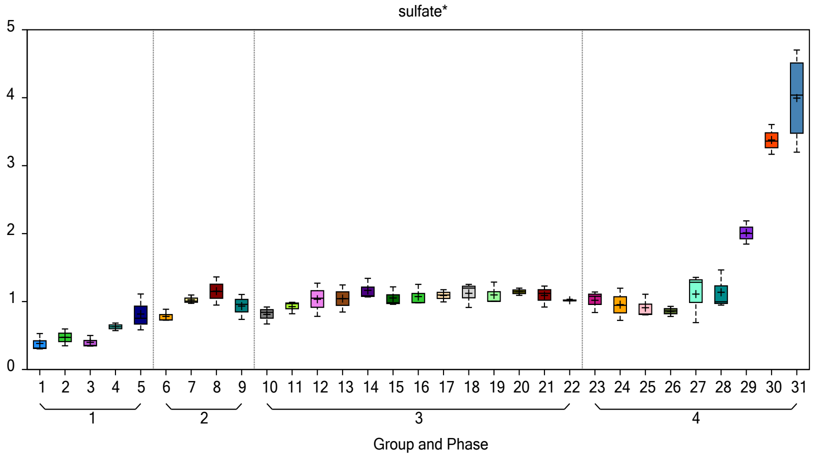
sulfate*
0
1
2
3
4
5
1 2 3 4 5 6 7 8 9 10 11 12 13 14 15 16 17 18 19 20 21 22 23 24 25 26 27 28 29 30 31
1
2
3
4
Group and Phase

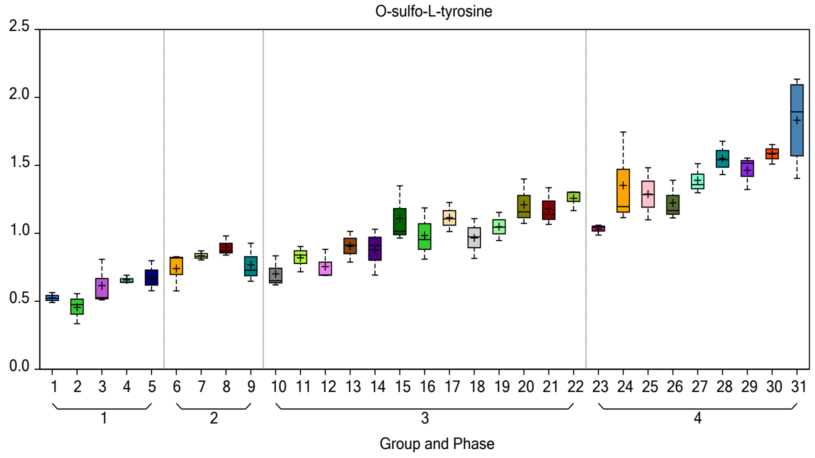
O-sulfo-L-tyrosine
0.0
0.5
1.0
1.5
2.0
2.5
1 2 3 4 5 6 7 8 9 10 11 12 13 14 15 16 17 18 19 20 21 22 23 24 25 26 27 28 29 30 31
1
2
3
4
Group and Phase

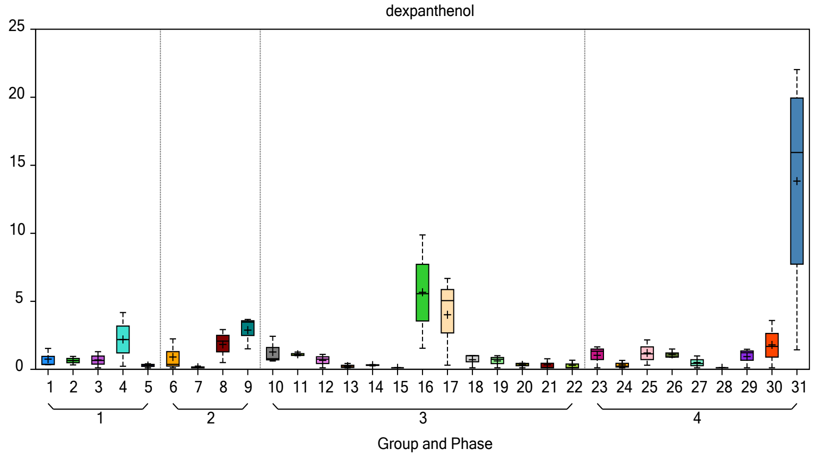
dexpanthenol
0
5
10
15
20
25
1 2 3 4 5 6 7 8 9 10 11 12 13 14 15 16 17 18 19 20 21 22 23 24 25 26 27 28 29 30 31
1
2
3
4
Group and Phase

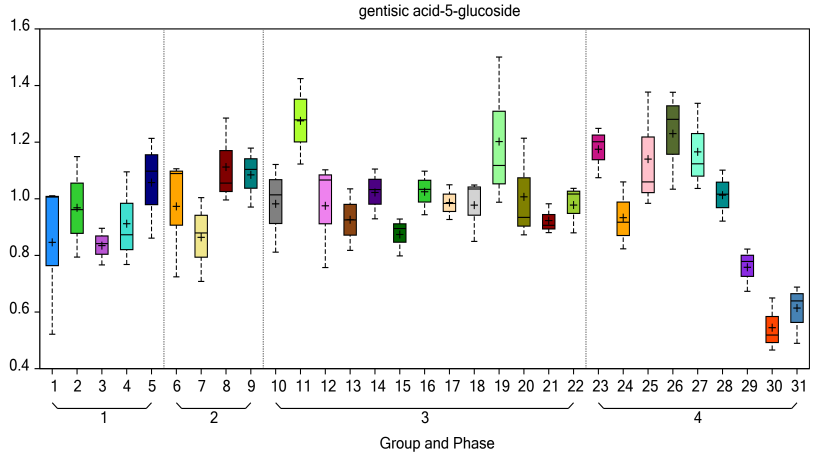
gentisic acid-5-glucoside
0.4
0.6
0.8
1.0
1.2
1.4
1.6
1 2 3 4 5 6 7 8 9 10 11 12 13 14 15 16 17 18 19 20 21 22 23 24 25 26 27 28 29 30 31
1
2
3
4
Group and Phase

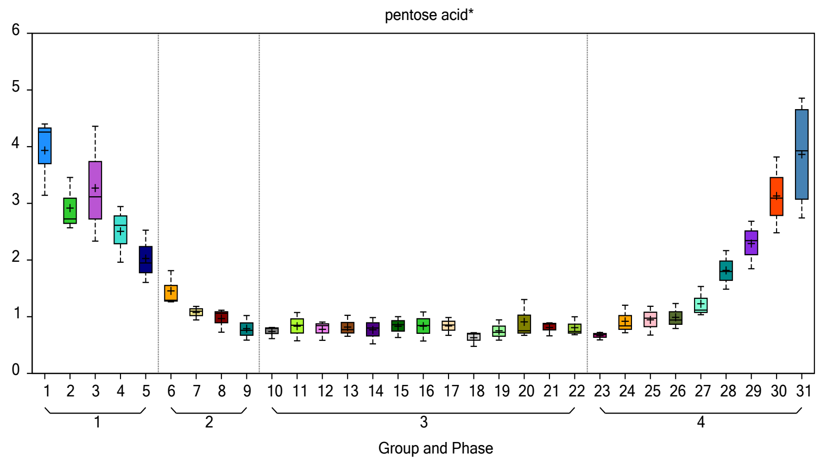
pentose acid*
0
1
2
3
4
5
6
1 2 3 4 5 6 7 8 9 10 11 12 13 14 15 16 17 18 19 20 21 22 23 24 25 26 27 28 29 30 31
1
2
3
4
Group and Phase
