# Supplementary material for: Metabolites in Cherry Buds to Detect Winter Dormancy
Source: Metabolites. 2022 Mar 16;12(3):247. doi: 10.3390/metabo12030247 (PMC8951522; doi:10.3390/metabo12030247)
Supplement: Supplementary file 1 [file metabolites-12-00247-s001.zip › metabolites-1609763-supplementary/Table S1.pdf]

Table S1: List of 445 metabolites found in sweet cherry buds (cv. 'Summit') in the 2015/16 season

Number	Metabolite	Super Pathway	Sub-Pathway
1	glycine	Amino Acid	Glycine, Serine and Threonine Metabolism
2	dimethylglycine	Amino Acid	Glycine, Serine and Threonine Metabolism
3	betaine	Amino Acid	Glycine, Serine and Threonine Metabolism
4	serine	Amino Acid	Glycine, Serine and Threonine Metabolism
5	N-acetylserine	Amino Acid	Glycine, Serine and Threonine Metabolism
6	threonine	Amino Acid	Glycine, Serine and Threonine Metabolism
7	alanine	Amino Acid	Alanine and Aspartate Metabolism
8	N-acetylalanine	Amino Acid	Alanine and Aspartate Metabolism
9	aspartate	Amino Acid	Alanine and Aspartate Metabolism
10	asparagine	Amino Acid	Alanine and Aspartate Metabolism
11	N-acetylasparagine	Amino Acid	Alanine and Aspartate Metabolism
12	glutamate	Amino Acid	Glutamate Metabolism
13	glutamine	Amino Acid	Glutamate Metabolism
14	alpha-ketoglutaramate*	Amino Acid	Glutamate Metabolism
15	N-acetylglutamate	Amino Acid	Glutamate Metabolism
16	N-acetylglutamine	Amino Acid	Glutamate Metabolism
17	glutamate, gamma-methyl ester	Amino Acid	Glutamate Metabolism
18	pyroglutamine*	Amino Acid	Glutamate Metabolism
19	gamma-aminobutyrate (GABA)	Amino Acid	Glutamate Metabolism
20	carboxyethyl-GABA	Amino Acid	Glutamate Metabolism
21	histidine	Amino Acid	Histidine Metabolism
22	1-methylhistidine	Amino Acid	Histidine Metabolism
23	3-methylhistidine	Amino Acid	Histidine Metabolism
24	N-acetylhistidine	Amino Acid	Histidine Metabolism
25	1-methyl-4-imidazoleacetate	Amino Acid	Histidine Metabolism
26	1-methyl-5-imidazoleacetate	Amino Acid	Histidine Metabolism
27	4-imidazoleacetate	Amino Acid	Histidine Metabolism
28	lysine	Amino Acid	Lysine Metabolism
29	N6-methyllysine	Amino Acid	Lysine Metabolism
30	N6,N6,N6-trimethyllysine	Amino Acid	Lysine Metabolism
31	fructosyllysine	Amino Acid	Lysine Metabolism
32	2-aminoadipate	Amino Acid	Lysine Metabolism
33	pipecolate	Amino Acid	Lysine Metabolism
34	6-oxopiperidine-2-carboxylate	Amino Acid	Lysine Metabolism
35	N,N,N-trimethyl-5-aminovalerate	Amino Acid	Lysine Metabolism
36	phenylalanine	Amino Acid	Phenylalanine Metabolism
37	phenylpyruvate	Amino Acid	Phenylalanine Metabolism
38	phenethylamine	Amino Acid	Phenylalanine Metabolism
39	tyrosine	Amino Acid	Tyrosine Metabolism
40	4-hydroxyphenylpyruvate	Amino Acid	Tyrosine Metabolism
41	tyrosol	Amino Acid	Tyrosine Metabolism
42	3-(4-hydroxyphenyl)lactate (HPLA)	Amino Acid	Tyrosine Metabolism
43	o-Tyrosine	Amino Acid	Tyrosine Metabolism
44	gentisate	Amino Acid	Tyrosine Metabolism
45	tryptophan	Amino Acid	Tryptophan Metabolism
46	kynurenine	Amino Acid	Tryptophan Metabolism
47	kynurenate	Amino Acid	Tryptophan Metabolism
48	xanthurenate	Amino Acid	Tryptophan Metabolism
49	leucine	Amino Acid	Leucine, Isoleucine and Valine Metabolism
50	3-methylglutaconate	Amino Acid	Leucine, Isoleucine and Valine Metabolism
51	isoleucine	Amino Acid	Leucine, Isoleucine and Valine Metabolism
52	methylsuccinate	Amino Acid	Leucine, Isoleucine and Valine Metabolism
53	valine	Amino Acid	Leucine, Isoleucine and Valine Metabolism
54	2,3-dihydroxy-2-methylbutyrate	Amino Acid	Leucine, Isoleucine and Valine Metabolism
55	methionine	Amino Acid	Methionine, Cysteine, SAM and Taurine Metabolism
56	N-acetylmethionine	Amino Acid	Methionine, Cysteine, SAM and Taurine Metabolism
57	S-methylmethionine	Amino Acid	Methionine, Cysteine, SAM and Taurine Metabolism
58	methionine sulfone	Amino Acid	Methionine, Cysteine, SAM and Taurine Metabolism
59	methionine sulfoxide	Amino Acid	Methionine, Cysteine, SAM and Taurine Metabolism
60	N-acetylmethionine sulfoxide	Amino Acid	Methionine, Cysteine, SAM and Taurine Metabolism
61	S-adenosylhomocysteine (SAH)	Amino Acid	Methionine, Cysteine, SAM and Taurine Metabolism
62	cysteine	Amino Acid	Methionine, Cysteine, SAM and Taurine Metabolism
63	cysteine sulfinic acid	Amino Acid	Methionine, Cysteine, SAM and Taurine Metabolism
64	N-acetyltaurine	Amino Acid	Methionine, Cysteine, SAM and Taurine Metabolism
65	3-sulfo-L-alanine	Amino Acid	Methionine, Cysteine, SAM and Taurine Metabolism
66	arginine	Amino Acid	Urea cycle; Arginine and Proline Metabolism
67	argininosuccinate	Amino Acid	Urea cycle; Arginine and Proline Metabolism
68	3-amino-2-piperidone	Amino Acid	Urea cycle; Arginine and Proline Metabolism

69	2-oxoarginine*	Amino Acid	Urea cycle; Arginine and Proline Metabolism
70	citrulline	Amino Acid	Urea cycle; Arginine and Proline Metabolism
71	homocitrulline	Amino Acid	Urea cycle; Arginine and Proline Metabolism
72	proline	Amino Acid	Urea cycle; Arginine and Proline Metabolism
73	dimethylarginine (ADMA + SDMA)	Amino Acid	Urea cycle; Arginine and Proline Metabolism
74	N-acetylarginine	Amino Acid	Urea cycle; Arginine and Proline Metabolism
75	N-acetylproline	Amino Acid	Urea cycle; Arginine and Proline Metabolism
76	N-delta-acetylornithine	Amino Acid	Urea cycle; Arginine and Proline Metabolism
77	hydroxyproline	Amino Acid	Urea cycle; Arginine and Proline Metabolism
78	N-methylproline	Amino Acid	Urea cycle; Arginine and Proline Metabolism
79	N,N,N-trimethyl-alanylproline betaine (TMAP)	Amino Acid	Urea cycle; Arginine and Proline Metabolism
80	N-monomethylarginine	Amino Acid	Urea cycle; Arginine and Proline Metabolism
81	guanidinoacetate	Amino Acid	Creatine Metabolism
82	putrescine	Amino Acid	Polyamine Metabolism
83	N-acetylputrescine	Amino Acid	Polyamine Metabolism
84	N-carbamoylputrescine	Amino Acid	Polyamine Metabolism
85	spermidine	Amino Acid	Polyamine Metabolism
86	5-methylthioadenosine (MTA)	Amino Acid	Polyamine Metabolism
87	4-guanidinobutanoate	Amino Acid	Guanidino and Acetamido Metabolism
88	glutathione, reduced (GSH)	Amino Acid	Glutathione Metabolism
89	glutathione, oxidized (GSSG)	Amino Acid	Glutathione Metabolism
90	cysteinylglycine	Amino Acid	Glutathione Metabolism
91	5-oxoproline	Amino Acid	Glutathione Metabolism
92	ophthalmate	Amino Acid	Glutathione Metabolism
93	gamma-glutamylglutamate	Peptide	Gamma-glutamyl Amino Acid
94	gamma-glutamylglutamine	Peptide	Gamma-glutamyl Amino Acid
95	gamma-glutamylhistidine	Peptide	Gamma-glutamyl Amino Acid
96	gamma-glutamylvaline	Peptide	Gamma-glutamyl Amino Acid
97	alanylleucine	Peptide	Dipeptide
98	glycylleucine	Peptide	Dipeptide
99	glycylvaline	Peptide	Dipeptide
100	isoleucylglycine	Peptide	Dipeptide
101	leucylalanine	Peptide	Dipeptide
102	leucylglycine	Peptide	Dipeptide
103	lysylleucine	Peptide	Dipeptide
104	phenylalanylalanine	Peptide	Dipeptide
105	phenylalanylglycine	Peptide	Dipeptide
106	prolylglycine	Peptide	Dipeptide
107	tryptophylglycine	Peptide	Dipeptide
108	tyrosylglycine	Peptide	Dipeptide
109	valylglutamine	Peptide	Dipeptide
110	valylglycine	Peptide	Dipeptide
111	valylleucine	Peptide	Dipeptide
112	leucylglutamine*	Peptide	Dipeptide
113	N,N-dimethyl-pro-pro	Peptide	Modified Peptides
114	glucose	Carbohydrate	Glycolysis, Gluconeogenesis, and Pyruvate Metabolism
115	glucose 6-phosphate	Carbohydrate	Glycolysis, Gluconeogenesis, and Pyruvate Metabolism
116	Isobar: hexose diphosphates	Carbohydrate	Glycolysis, Gluconeogenesis, and Pyruvate Metabolism
117	3-phosphoglycerate	Carbohydrate	Glycolysis, Gluconeogenesis, and Pyruvate Metabolism
118	pyruvate	Carbohydrate	Glycolysis, Gluconeogenesis, and Pyruvate Metabolism
119	glycerate	Carbohydrate	Glycolysis, Gluconeogenesis, and Pyruvate Metabolism
120	ribose	Carbohydrate	Pentose Metabolism
121	ribitol	Carbohydrate	Pentose Metabolism
122	ribonate	Carbohydrate	Pentose Metabolism
123	ribulose/xylulose	Carbohydrate	Pentose Metabolism
124	xylose	Carbohydrate	Pentose Metabolism
125	arabinose	Carbohydrate	Pentose Metabolism
126	arabitol/xylitol	Carbohydrate	Pentose Metabolism
127	arabonate/xylonate	Carbohydrate	Pentose Metabolism
128	sedoheptulose	Carbohydrate	Pentose Metabolism
129	ribulonate/xylulonate/lyxonate*	Carbohydrate	Pentose Metabolism
130	maltose	Carbohydrate	Glycogen Metabolism
131	stachyose	Carbohydrate	Disaccharides and Oligosaccharides
132	sucrose	Carbohydrate	Disaccharides and Oligosaccharides
133	raffinose	Carbohydrate	Disaccharides and Oligosaccharides
134	maltitol/lactitol/cellobiotol/palatinol	Carbohydrate	Disaccharides and Oligosaccharides
135	fructose	Carbohydrate	Fructose, Mannose and Galactose Metabolism
136	mannitol/sorbitol	Carbohydrate	Fructose, Mannose and Galactose Metabolism
137	mannose	Carbohydrate	Fructose, Mannose and Galactose Metabolism

138	galactonate	Carbohydrate	Fructose, Mannose and Galactose Metabolism
139	UDP-glucose	Carbohydrate	Nucleotide Sugar
140	UDP-galactose	Carbohydrate	Nucleotide Sugar
141	UDP-N-acetylglucosamine/galactosamine	Carbohydrate	Nucleotide Sugar
142	glucuronate	Carbohydrate	Aminosugar Metabolism
143	N-acetylglucosamine 6-phosphate	Carbohydrate	Aminosugar Metabolism
144	N-acetylglucosaminylasparagine	Carbohydrate	Aminosugar Metabolism
145	erythronate*	Carbohydrate	Aminosugar Metabolism
146	N-acetylglucosamine/N-acetylgalactosamine	Carbohydrate	Aminosugar Metabolism
147	N6-carboxymethyllysine	Carbohydrate	Advanced Glycation End-product
148	citrate	Energy	TCA Cycle
149	aconitate [cis or trans]	Energy	TCA Cycle
150	isocitrate	Energy	TCA Cycle
151	isocitric lactone	Energy	TCA Cycle
152	alpha-ketoglutarate	Energy	TCA Cycle
153	succinate	Energy	TCA Cycle
154	fumarate	Energy	TCA Cycle
155	malate	Energy	TCA Cycle
156	tricarballylate	Energy	TCA Cycle
157	2-methylcitrate	Energy	TCA Cycle
158	citraconate/glutaconate	Energy	TCA Cycle
159	phosphate	Energy	Oxidative Phosphorylation
160	malonate	Lipid	Fatty Acid Synthesis
161	arachidate (20:0)	Lipid	Long Chain Saturated Fatty Acid
162	hexadecatrienoate (16:3n3)	Lipid	Long Chain Polyunsaturated Fatty Acid (n3 and n6)
163	linoleate (18:2n6)	Lipid	Long Chain Polyunsaturated Fatty Acid (n3 and n6)
164	linolenate (18:3n3 or 3n6)	Lipid	Long Chain Polyunsaturated Fatty Acid (n3 and n6)
165	glutarate (C5-DC)	Lipid	Fatty Acid, Dicarboxylate
166	2-hydroxyglutarate	Lipid	Fatty Acid, Dicarboxylate
167	3-hydroxyadipate	Lipid	Fatty Acid, Dicarboxylate
168	maleate	Lipid	Fatty Acid, Dicarboxylate
169	dodecenedioate (C12:1-DC)*	Lipid	Fatty Acid, Dicarboxylate
170	hexadecanedioate (C16)	Lipid	Fatty Acid, Dicarboxylate
171	octadecanedioate (C18)	Lipid	Fatty Acid, Dicarboxylate
172	octadecenedioate (C18:1-DC)	Lipid	Fatty Acid, Dicarboxylate
173	octadecadienedioate (C18:2-DC)*	Lipid	Fatty Acid, Dicarboxylate
174	eicosanedioate (C20-DC)	Lipid	Fatty Acid, Dicarboxylate
175	docosadioate (C22-DC)	Lipid	Fatty Acid, Dicarboxylate
176	2-hydroxysebacate	Lipid	Fatty Acid, Dicarboxylate
177	linoleoylcholine*	Lipid	Fatty Acid Metabolism (Acyl Choline)
178	2-hydroxyarachidate*	Lipid	Fatty Acid, Monohydroxy
179	2-hydroxybehenate	Lipid	Fatty Acid, Monohydroxy
180	3-hydroxysebacate	Lipid	Fatty Acid, Monohydroxy
181	3-hydroxypalmitate	Lipid	Fatty Acid, Monohydroxy
182	3-hydroxystearate	Lipid	Fatty Acid, Monohydroxy
183	3-hydroxyarachidate*	Lipid	Fatty Acid, Monohydroxy
184	3-hydroxybehenate*	Lipid	Fatty Acid, Monohydroxy
185	16-hydroxypalmitate	Lipid	Fatty Acid, Monohydroxy
186	13-HODE + 9-HODE	Lipid	Fatty Acid, Monohydroxy
187	2S,3R-dihydroxybutyrate	Lipid	Fatty Acid, Dihydroxy
188	2R,3R-dihydroxybutyrate	Lipid	Fatty Acid, Dihydroxy
189	2,4-dihydroxybutyrate	Lipid	Fatty Acid, Dihydroxy
190	3,4-dihydroxybutyrate	Lipid	Fatty Acid, Dihydroxy
191	oleoyl ethanolamide	Lipid	Endocannabinoid
192	linoleoyl ethanolamide	Lipid	Endocannabinoid
193	linolenoyl ethanolamide	Lipid	Endocannabinoid
194	myo-inositol	Lipid	Inositol Metabolism
195	inositol 1-phosphate (I1P)	Lipid	Inositol Metabolism
196	inositol trisphosphate	Lipid	Inositol Metabolism
197	choline	Lipid	Phospholipid Metabolism
198	phosphocholine	Lipid	Phospholipid Metabolism
199	glycerophosphorylcholine (GPC)	Lipid	Phospholipid Metabolism
200	phosphoethanolamine (PE)	Lipid	Phospholipid Metabolism
201	glycerophosphoethanolamine	Lipid	Phospholipid Metabolism
202	glycerophosphoserine*	Lipid	Phospholipid Metabolism
203	glycerophosphoinositol*	Lipid	Phospholipid Metabolism
204	1-palmitoyl-2-linoleoyl-GPA (16:0/18:2)*	Lipid	Phosphatidic acid (PA)
205	1-palmitoyl-2-linolenoyl-GPA (16:0/18:3)*	Lipid	Phosphatidic acid (PA)
206	1-oleoyl-2-linoleoyl-GPA (18:1/18:2)*	Lipid	Phosphatidic acid (PA)

207	1,2-dilinoleoyl-GPA (18:2/18:2)*	Lipid	Phosphatidic acid (PA)
208	1-linoleoyl-2-linolenoyl-GPA (18:2/18:3)*	Lipid	Phosphatidic acid (PA)
209	1,2-dilinolenoyl-GPA (18:3/18:3)*	Lipid	Phosphatidic acid (PA)
210	1-palmitoyl-2-oleoyl-GPC (16:0/18:1)	Lipid	Phosphatidylcholine (PC)
211	1-palmitoyl-2-linoleoyl-GPC (16:0/18:2)	Lipid	Phosphatidylcholine (PC)
212	1-palmitoyl-2-alpha-linolenoyl-GPC (16:0/18:3n3)*	Lipid	Phosphatidylcholine (PC)
213	1-palmitoleoyl-2-linolenoyl-GPC (16:1/18:3)*	Lipid	Phosphatidylcholine (PC)
214	1-stearoyl-2-oleoyl-GPC (18:0/18:1)	Lipid	Phosphatidylcholine (PC)
215	1-stearoyl-2-linoleoyl-GPC (18:0/18:2)*	Lipid	Phosphatidylcholine (PC)
216	1-oleoyl-2-linoleoyl-GPC (18:1/18:2)*	Lipid	Phosphatidylcholine (PC)
217	1,2-dilinoleoyl-GPC (18:2/18:2)	Lipid	Phosphatidylcholine (PC)
218	1-linoleoyl-2-linolenoyl-GPC (18:2/18:3)*	Lipid	Phosphatidylcholine (PC)
219	1,2-dilinolenoyl-GPC (18:3/18:3)*	Lipid	Phosphatidylcholine (PC)
220	1-palmitoyl-2-oleoyl-GPE (16:0/18:1)	Lipid	Phosphatidylethanolamine (PE)
221	1-palmitoyl-2-linoleoyl-GPE (16:0/18:2)	Lipid	Phosphatidylethanolamine (PE)
222	1-stearoyl-2-linoleoyl-GPE (18:0/18:2)*	Lipid	Phosphatidylethanolamine (PE)
223	1-oleoyl-2-linoleoyl-GPE (18:1/18:2)*	Lipid	Phosphatidylethanolamine (PE)
224	1,2-dilinoleoyl-GPE (18:2/18:2)*	Lipid	Phosphatidylethanolamine (PE)
225	1,2-dipalmitoyl-GPG (16:0/16:0)	Lipid	Phosphatidylglycerol (PG)
226	1-palmitoyl-2-oleoyl-GPG (16:0/18:1)	Lipid	Phosphatidylglycerol (PG)
227	1-palmitoyl-2-linoleoyl-GPG (16:0/18:2)	Lipid	Phosphatidylglycerol (PG)
228	1-palmitoyl-2-linolenoyl-GPG (16:0/18:3)*	Lipid	Phosphatidylglycerol (PG)
229	1-palmitoyl-2-linoleoyl-GPI (16:0/18:2)	Lipid	Phosphatidylinositol (PI)
230	1-stearoyl-2-linoleoyl-GPI (18:0/18:2)	Lipid	Phosphatidylinositol (PI)
231	1-palmitoyl-GPA (16:0)	Lipid	Lysophospholipid
232	1-linoleoyl-GPA (18:2)*	Lipid	Lysophospholipid
233	1-linolenoyl-GPA (18:3)*	Lipid	Lysophospholipid
234	1-linolenoyl-GPG (18:3)*	Lipid	Lysophospholipid
235	1-linoleoyl-GPC (18:2)	Lipid	Lysophospholipid
236	1-linolenoyl-GPC (18:3)*	Lipid	Lysophospholipid
237	1-palmitoyl-GPE (16:0)	Lipid	Lysophospholipid
238	1-stearoyl-GPE (18:0)	Lipid	Lysophospholipid
239	1-linoleoyl-GPE (18:2)*	Lipid	Lysophospholipid
240	1-palmitoyl-GPG (16:0)*	Lipid	Lysophospholipid
241	1-linoleoyl-GPG (18:2)*	Lipid	Lysophospholipid
242	1-palmitoyl-GPI* (16:0)	Lipid	Lysophospholipid
243	1-stearoyl-GPI (18:0)	Lipid	Lysophospholipid
244	1-linoleoyl-GPI* (18:2)*	Lipid	Lysophospholipid
245	glycerol 3-phosphate	Lipid	Glycerolipid Metabolism
246	glycerophosphoglycerol	Lipid	Glycerolipid Metabolism
247	1-palmitoylglycerol (16:0)	Lipid	Monoacylglycerol
248	1-palmitoleoylglycerol (16:1)*	Lipid	Monoacylglycerol
249	1-oleoylglycerol (18:1)	Lipid	Monoacylglycerol
250	1-linoleoylglycerol (18:2)	Lipid	Monoacylglycerol
251	1-linolenoylglycerol (18:3)	Lipid	Monoacylglycerol
252	2-linoleoylglycerol (18:2)	Lipid	Monoacylglycerol
253	diacylglycerol (16:1/18:2 [2], 16:0/18:3 [1])*	Lipid	Diacylglycerol
254	palmitoyl-linoleoyl-glycerol (16:0/18:2) [1]*	Lipid	Diacylglycerol
255	palmitoyl-linoleoyl-glycerol (16:0/18:2) [2]*	Lipid	Diacylglycerol
256	palmitoyl-linolenoyl-glycerol (16:0/18:3) [2]*	Lipid	Diacylglycerol
257	oleoyl-linoleoyl-glycerol (18:1/18:2) [2]	Lipid	Diacylglycerol
258	oleoyl-linolenoyl-glycerol (18:1/18:3) [2]*	Lipid	Diacylglycerol
259	linoleoyl-linoleoyl-glycerol (18:2/18:2) [1]*	Lipid	Diacylglycerol
260	linoleoyl-linoleoyl-glycerol (18:2/18:2) [2]*	Lipid	Diacylglycerol
261	linoleoyl-linolenoyl-glycerol (18:2/18:3) [1]*	Lipid	Diacylglycerol
262	linoleoyl-linolenoyl-glycerol (18:2/18:3) [2]*	Lipid	Diacylglycerol
263	linolenoyl-linolenoyl-glycerol (18:3/18:3) [1]*	Lipid	Diacylglycerol
264	linolenoyl-linolenoyl-glycerol (18:3/18:3) [2]*	Lipid	Diacylglycerol
265	SQDG (18:3/16:0)	Lipid	Diacylglycerol
266	galactosylglycerol	Lipid	Galactosyl Glycerolipids
267	digalactosylglycerol*	Lipid	Galactosyl Glycerolipids
268	1-palmitoyl-2-linoleoyl-digalactosylglycerol (16:0/18:2)	Lipid	Galactosyl Glycerolipids
269	1-palmitoyl-2-linoleoyl-galactosylglycerol (16:0/18:2)*	Lipid	Galactosyl Glycerolipids
270	1-palmitoyl-2-linolenoyl-galactosylglycerol (16:0/18:3)	Lipid	Galactosyl Glycerolipids
271	1-palmitoyl-2-linolenoyl-digalactosylglycerol (16:0/18:	Lipid	Galactosyl Glycerolipids
272	1-linolenoyl-2-hexadecatrienoyl-galactosylglycerol (18	Lipid	Galactosyl Glycerolipids
273	1,2-dilinoleoyl-digalactosylglycerol (18:2/18:2)*	Lipid	Galactosyl Glycerolipids
274	1,2-dilinoleoyl-galactosylglycerol (18:2/18:2)*	Lipid	Galactosyl Glycerolipids
275	1-linoleoyl-2-linolenoyl-galactosylglycerol (18:2/18:3)*	Lipid	Galactosyl Glycerolipids

276	1-linoleoyl-2-linolenoyl-digalactosylglycerol (18:2/18:3	Lipid	Galactosyl Glycerolipids
277	1,2-dilinolenoyl-galactosylglycerol (18:3/18:3)*	Lipid	Galactosyl Glycerolipids
278	1,2-dilinolenoyl-digalactosylglycerol (18:3/18:3)	Lipid	Galactosyl Glycerolipids
279	1-linoleoyl-galactosylglycerol (18:2)*	Lipid	Galactosyl Glycerolipids
280	1-linoleoyl-digalactosylglycerol (18:2)*	Lipid	Galactosyl Glycerolipids
281	1-linolenoyl-digalactosylglycerol (18:3)*	Lipid	Galactosyl Glycerolipids
282	2-linolenoyl-digalactosylglycerol (18:3)*	Lipid	Galactosyl Glycerolipids
283	sphinganine	Lipid	Sphingolipid Synthesis
284	phytosphingosine	Lipid	Sphingolipid Synthesis
285	dehydrophytosphingosine*	Lipid	Sphingolipid Synthesis
286	sphingosine	Lipid	Sphingosines
287	3-hydroxy-3-methylglutarate	Lipid	Mevalonate Metabolism
288	beta-sitosterol	Lipid	Sterol
289	inosine	Nucleotide	Purine Metabolism, (Hypo)Xanthine/Inosine containing
290	hypoxanthine	Nucleotide	Purine Metabolism, (Hypo)Xanthine/Inosine containing
291	xanthine	Nucleotide	Purine Metabolism, (Hypo)Xanthine/Inosine containing
292	xanthosine	Nucleotide	Purine Metabolism, (Hypo)Xanthine/Inosine containing
293	allantoin	Nucleotide	Purine Metabolism, (Hypo)Xanthine/Inosine containing
294	allantoic acid	Nucleotide	Purine Metabolism, (Hypo)Xanthine/Inosine containing
295	AMP	Nucleotide	Purine Metabolism, Adenine containing
296	3'-AMP	Nucleotide	Purine Metabolism, Adenine containing
297	adenosine-2',3'-cyclic monophosphate	Nucleotide	Purine Metabolism, Adenine containing
298	adenylosuccinate	Nucleotide	Purine Metabolism, Adenine containing
299	adenosine	Nucleotide	Purine Metabolism, Adenine containing
300	adenine	Nucleotide	Purine Metabolism, Adenine containing
301	1-methyladenine	Nucleotide	Purine Metabolism, Adenine containing
302	2'-O-methyladenosine	Nucleotide	Purine Metabolism, Adenine containing
303	2'-deoxyadenosine	Nucleotide	Purine Metabolism, Adenine containing
304	N6-succinyladenosine	Nucleotide	Purine Metabolism, Adenine containing
305	5'- GMP	Nucleotide	Purine Metabolism, Guanine containing
306	guanosine 3'-monophosphate (3'-GMP)	Nucleotide	Purine Metabolism, Guanine containing
307	guanosine-2',3'-cyclic monophosphate	Nucleotide	Purine Metabolism, Guanine containing
308	guanosine	Nucleotide	Purine Metabolism, Guanine containing
309	guanine	Nucleotide	Purine Metabolism, Guanine containing
310	1-methylguanine	Nucleotide	Purine Metabolism, Guanine containing
311	N-carbamoylaspartate	Nucleotide	Pyrimidine Metabolism, Orotate containing
312	dihydroorotate	Nucleotide	Pyrimidine Metabolism, Orotate containing
313	orotate	Nucleotide	Pyrimidine Metabolism, Orotate containing
314	UMP	Nucleotide	Pyrimidine Metabolism, Uracil containing
315	uridine-2',3'-cyclic monophosphate	Nucleotide	Pyrimidine Metabolism, Uracil containing
316	uridine	Nucleotide	Pyrimidine Metabolism, Uracil containing
317	uracil	Nucleotide	Pyrimidine Metabolism, Uracil containing
318	pseudouridine	Nucleotide	Pyrimidine Metabolism, Uracil containing
319	5,6-dihydrouridine	Nucleotide	Pyrimidine Metabolism, Uracil containing
320	3-ureidopropionate	Nucleotide	Pyrimidine Metabolism, Uracil containing
321	beta-alanine	Nucleotide	Pyrimidine Metabolism, Uracil containing
322	3-(3-amino-3-carboxypropyl)uridine*	Nucleotide	Pyrimidine Metabolism, Uracil containing
323	CMP	Nucleotide	Pyrimidine Metabolism, Cytidine containing
324	cytidine 2' or 3'-monophosphate (2' or 3'-CMP)	Nucleotide	Pyrimidine Metabolism, Cytidine containing
325	cytidine 2',3'-cyclic monophosphate	Nucleotide	Pyrimidine Metabolism, Cytidine containing
326	cytidine	Nucleotide	Pyrimidine Metabolism, Cytidine containing
327	cytosine	Nucleotide	Pyrimidine Metabolism, Cytidine containing
328	thymidine	Nucleotide	Pyrimidine Metabolism, Thymine containing
329	3-aminoisobutyrate	Nucleotide	Pyrimidine Metabolism, Thymine containing
330	methylphosphate	Nucleotide	Purine and Pyrimidine Metabolism
331	nicotinate	Cofactors and Vitar	Nicotinate and Nicotinamide Metabolism
332	nicotinate ribonucleoside	Cofactors and Vitar	Nicotinate and Nicotinamide Metabolism
333	nicotinamide	Cofactors and Vitar	Nicotinate and Nicotinamide Metabolism
334	nicotinamide ribonucleotide (NMN)	Cofactors and Vitar	Nicotinate and Nicotinamide Metabolism
335	nicotinamide riboside	Cofactors and Vitar	Nicotinate and Nicotinamide Metabolism
336	NAD+	Cofactors and Vitar	Nicotinate and Nicotinamide Metabolism
337	trigonelline (N'-methylnicotinate)	Cofactors and Vitar	Nicotinate and Nicotinamide Metabolism
338	pantothenate (Vitamin B5)	Cofactors and Vitar	Pantothenate and CoA Metabolism
339	ascorbate (Vitamin C)	Cofactors and Vitar	Ascorbate and Aldarate Metabolism
340	dehydroascorbate	Cofactors and Vitar	Ascorbate and Aldarate Metabolism
341	2-O-methylascorbic acid	Cofactors and Vitar	Ascorbate and Aldarate Metabolism
342	threonate	Cofactors and Vitar	Ascorbate and Aldarate Metabolism
343	gulonate*	Cofactors and Vitar	Ascorbate and Aldarate Metabolism
344	alpha-tocopherol	Cofactors and Vitar	Tocopherol Metabolism

345	delta-tocopherol	Cofactors and Vitar	Tocopherol Metabolism
346	gamma-tocopherol/beta-tocopherol	Cofactors and Vitar	Tocopherol Metabolism
347	thiamin (Vitamin B1)	Cofactors and Vitar	Thiamine Metabolism
348	carotene diol (1)	Cofactors and Vitar	Vitamin A Metabolism
349	carotene diol (2)	Cofactors and Vitar	Vitamin A Metabolism
350	carotene diol (3)	Cofactors and Vitar	Vitamin A Metabolism
351	pyridoxine (Vitamin B6)	Cofactors and Vitar	Vitamin B6 Metabolism
352	pyridoxamine	Cofactors and Vitar	Vitamin B6 Metabolism
353	pyridoxal	Cofactors and Vitar	Vitamin B6 Metabolism
354	pyridoxate	Cofactors and Vitar	Vitamin B6 Metabolism
355	4-hydroxybenzoate	Xenobiotics	Benzoate Metabolism
356	2,4,6-trihydroxybenzoate	Xenobiotics	Benzoate Metabolism
357	3-hydroxybenzaldehyde	Xenobiotics	Benzoate Metabolism
358	benzoyl-O-glucose	Xenobiotics	Benzoate Metabolism
359	3-(4-hydroxyphenyl)propionate	Xenobiotics	Benzoate Metabolism
360	kaempferol	Xenobiotics	Food Component/Plant
361	kaempferol 7-O-glucoside	Xenobiotics	Food Component/Plant
362	dihydrokaempferol	Xenobiotics	Food Component/Plant
363	quercetin	Xenobiotics	Food Component/Plant
364	quercetin 3-galactoside	Xenobiotics	Food Component/Plant
365	indoleacetylaspartate	Xenobiotics	Food Component/Plant
366	genistein	Xenobiotics	Food Component/Plant
367	shikimate	Xenobiotics	Food Component/Plant
368	3-dehydroshikimate	Xenobiotics	Food Component/Plant
369	catechin	Xenobiotics	Food Component/Plant
370	epicatechin	Xenobiotics	Food Component/Plant
371	cosmosiin	Xenobiotics	Food Component/Plant
372	apigenin	Xenobiotics	Food Component/Plant
373	luteolin	Xenobiotics	Food Component/Plant
374	luteolin 7-O-glucoside	Xenobiotics	Food Component/Plant
375	vanillate	Xenobiotics	Food Component/Plant
376	vanillin	Xenobiotics	Food Component/Plant
377	2,3-dihydroxyisovalerate	Xenobiotics	Food Component/Plant
378	2-isopropylmalate	Xenobiotics	Food Component/Plant
379	3-formylindole	Xenobiotics	Food Component/Plant
380	gluconate	Xenobiotics	Food Component/Plant
381	abscisate	Xenobiotics	Food Component/Plant
382	beta-guanidinopropanoate	Xenobiotics	Food Component/Plant
383	caffeate	Xenobiotics	Food Component/Plant
384	3-hydroxycinnamate	Xenobiotics	Food Component/Plant
385	chlorogenate	Xenobiotics	Food Component/Plant
386	chrysin	Xenobiotics	Food Component/Plant
387	chrysoeriol	Xenobiotics	Food Component/Plant
388	coumaroylquinate (2)	Xenobiotics	Food Component/Plant
389	coumaroylquinate (3)	Xenobiotics	Food Component/Plant
390	coumaroylquinate (4)	Xenobiotics	Food Component/Plant
391	coumaroylquinate (5)	Xenobiotics	Food Component/Plant
392	cryptochlorogenic acid	Xenobiotics	Food Component/Plant
393	deoxymugineic acid	Xenobiotics	Food Component/Plant
394	dihydroferulate	Xenobiotics	Food Component/Plant
395	dihydroquercetin	Xenobiotics	Food Component/Plant
396	eriodictyol	Xenobiotics	Food Component/Plant
397	erythritol	Xenobiotics	Food Component/Plant
398	ferulate	Xenobiotics	Food Component/Plant
399	quinate	Xenobiotics	Food Component/Plant
400	feruloylquinate (1)	Xenobiotics	Food Component/Plant
401	feruloylquinate (2)	Xenobiotics	Food Component/Plant
402	feruloylquinate (3)	Xenobiotics	Food Component/Plant
403	feruloylquinate (4)	Xenobiotics	Food Component/Plant
404	feruloylquinate (5)	Xenobiotics	Food Component/Plant
405	fucitol	Xenobiotics	Food Component/Plant
406	histidine betaine (hercynine)*	Xenobiotics	Food Component/Plant
407	homocitrate	Xenobiotics	Food Component/Plant
408	hydroquinone beta-D-glucopyranoside	Xenobiotics	Food Component/Plant
409	isorhamnetin 3-rutinoside	Xenobiotics	Food Component/Plant
410	isorhamnetin	Xenobiotics	Food Component/Plant
411	lariciresinol 4-O-glucoside	Xenobiotics	Food Component/Plant
412	lariciresinol	Xenobiotics	Food Component/Plant
413	mannonate*	Xenobiotics	Food Component/Plant

414	dihydrooroxylin	Xenobiotics	Food Component/Plant
415	naringenin 7-O-glucoside	Xenobiotics	Food Component/Plant
416	naringenin	Xenobiotics	Food Component/Plant
417	nicotianamine	Xenobiotics	Food Component/Plant
418	oleanolate	Xenobiotics	Food Component/Plant
419	pheophorbide A	Xenobiotics	Food Component/Plant
420	pheophytin A	Xenobiotics	Food Component/Plant
421	pinoresinol	Xenobiotics	Food Component/Plant
422	procyanidin B1	Xenobiotics	Food Component/Plant
423	procyanidin B2	Xenobiotics	Food Component/Plant
424	quercetin 3-glucoside	Xenobiotics	Food Component/Plant
425	rutin	Xenobiotics	Food Component/Plant
426	stachydrine	Xenobiotics	Food Component/Plant
427	methyl glucopyranoside (alpha + beta)	Xenobiotics	Food Component/Plant
428	kaempferol 3-O-glucoside/galactoside	Xenobiotics	Food Component/Plant
429	tamarixetin	Xenobiotics	Food Component/Plant
430	2-hydroxycinnamate	Xenobiotics	Food Component/Plant
431	4-hydroxycinnamate	Xenobiotics	Food Component/Plant
432	sakuranetin	Xenobiotics	Food Component/Plant
433	2-dimethylaminoethanol	Xenobiotics	Food Component/Plant
434	ethyl beta-glucopyranoside	Xenobiotics	Food Component/Plant
435	NP-000890	Xenobiotics	Food Component/Plant
436	NP-004685	Xenobiotics	Food Component/Plant
437	salicylate-glucoside	Xenobiotics	Drug - Analgesics, Anesthetics
438	salidroside	Xenobiotics	Drug - Psychoactive
439	salicylate	Xenobiotics	Drug - Topical Agents
440	2,6-dihydroxybenzoic acid	Xenobiotics	Drug - Topical Agents
441	sulfate*	Xenobiotics	Chemical
442	O-sulfo-L-tyrosine	Xenobiotics	Chemical
443	dexpanthenol	Xenobiotics	Chemical
444	gentisic acid-5-glucoside	Xenobiotics	Chemical
445	pentose acid*	Partially Characteri	Partially Characterized Molecules
